# Identification and evaluation of midbrain specific longevity-related genes in exceptionally long-lived but healthy mice

**DOI:** 10.3389/fnagi.2022.1030807

**Published:** 2023-01-11

**Authors:** Hyojung Kim, Yu-Jin Huh, Ji Hun Kim, Minkyung Jo, Joo-Heon Shin, Sang Chul Park, Jee-Yin Ahn, Yun-Il Lee, Yunjong Lee

**Affiliations:** ^1^Department of Pharmacology, Sungkyunkwan University School of Medicine, Suwon, Republic of Korea; ^2^Division of Biotechnology, Department of Interdisciplinary Studies, Well Aging Research Center, DGIST, Daegu, Republic of Korea; ^3^Department of New Biology, DGIST, Daegu, Republic of Korea; ^4^Lieber Institute for Brain Development, Johns Hopkins Medical Campus, Baltimore, MD, United States; ^5^Department of Neurology, Johns Hopkins School of Medicine, Baltimore, MD, United States; ^6^The Future Life & Society Research Center, Advanced Institute of Aging Science, Chonnam National University, Gwangju, Republic of Korea; ^7^Department of Molecular Cell Biology, Sungkyunkwan University School of Medicine, Suwon, Republic of Korea

**Keywords:** longevity, motor function, dopaminergic neurites, gene prioritization, parkin, AIMP2, NRF1, Akt

## Abstract

Brain aging is a complex biological process that is affected by both genetic background and environment. The transcriptomic analysis of aged human and rodent brains has been applied to identify age-associated molecular and cellular processes for which intervention could possibly restore declining brain functions induced by aging. However, whether these age-associated genetic alterations are indeed involved in the healthy aging of the brain remains unclear. We herein characterized a naturally occurring, extremely long-lived (34 months of age) but healthy mouse group retaining well-preserved motor functions. Strikingly, these long-lived mice maintained tyrosine hydroxylase expression and dopaminergic fiber densities, even in the presence of persistent neuroinflammation and expression of aging markers. Combined with Endeavor gene prioritization, we identified the following midbrain-specific longevity-associated genes in the midbrain of these mice: *aimp2, hexb, cacybp, akt2, nrf1, axin1, wwp2, sp2, dnajb9, notch, traf7*, and *lrp1*. A detailed biochemical analysis of the midbrain of these long-lived mice confirmed the increased expression of Nrf1 and the activation of Akt1 and 2. Interestingly, dopaminergic neuroprotective and age-associated E3 ubiquitin ligase parkin expression was retained at high levels in the aforementioned midbrains, possibly supporting the suppression of its toxic substrates AIMP2 and PARIS. In contrast, the 24-month-old mice with dopaminergic neurite deficits failed to maintain parkin expression in the midbrain. AIMP2-induced cytotoxicity, mitochondrial stress, and neurite toxicity can be prevented by overexpression of parkin, Akt1, and Nrf1 in SH-SY5Y and PC12 cells, and basal expression of parkin, Akt1, and Nrf1 is required for maintenance of mitochondrial function and neurite integrity in PC12 cells. Taken together, this longevity-associated pathway could be a potential target of intervention to maintain nigrostriatal dopaminergic fibers and motor ability to ensure healthy longevity.

## Introduction

Aging is a complicated biological process that is influenced by genetic background and environment (de Magalhaes et al., 2012). Accordingly, different strains of mice exhibit a wide range of lifespan discrepancies, but even the same mouse strain can manifest quite different lifespans depending on diet, crowding, differences in breeding history, and viral infection in mouse housing facilities (Green, 1966; Yuan et al., 2009). During aging, it has become evident that cellular and organ functions tend to decline, and this functional decline is associated with aging hallmarks, including the dysregulation of proteostasis, accumulation of oxidative stress, DNA damage, telomere attrition, epigenome alteration, inflammation, and metabolic alteration (Lopez-Otin et al., 2013). Among the diverse organs affected during aging, brain aging is relatively challenging owing to the complexity of cellular components and the distinct neuronal composition in brain subregions. Understanding the molecular mechanisms of brain aging could be critically important, partly because it is the strongest risk factor for neurodegenerative diseases, such as Parkinson’s disease. The transcriptome analysis of aged human or rodent brains has revealed age-associated genes and relevant biological processes (Loerch et al., 2008; Wood et al., 2013; Glaab and Schneider, 2015). However, whether targeting these age-associated genes could reverse or prevent the functional decline of the brain during aging remains unclear. Accordingly, as an alternative strategy, the molecular signatures in naturally occurring extremely long-lived mice (~34 months of age, equivalent to a human centenarian) without severe aged phenotypes and physiological dysfunction can be characterized since these outlier age groups somehow develop genetic programs to adapt to accumulating aging stresses. Indeed, naturally occurring, extremely long-lived mice retain preserved immune function and controlled Nuclear Factor Kappa B activity compared to aged old mice (Arranz et al., 2010). Therefore, it would be more effective to study molecular alterations in healthy, extremely long-lived mice with relatively preserved brain function to identify longevity and anti-aging genes.

Parkin is an E3 ubiquitin ligase whose activity is critical for maintaining the survival and function of midbrain dopaminergic neurons (Ko et al., 2010). Dysfunctional parkin activity due to aging (Lee et al., 2016) and Parkinson’s disease (PD) pathogenesis contribute to nigrostriatal deficits and motor impairment. It has been shown that the accumulation of parkin substrates is responsible for midbrain dopamine cell toxicity downstream of parkin deficits (Ko et al., 2010). Specifically, the accumulation of the parkin substrate Aminoacyl tRNA synthetase complex-interacting multifunctional protein 2 (AIMP2) can aggregate α-synuclein and induce progressive dopaminergic neurodegeneration in the midbrain (Lee et al., 2013). Moreover, another pathogenic parkin substrate, the parkin-interacting substrate (PARIS, also known as ZNF746), impairs dopamine cell survival and antioxidant defense *via* the transcriptional repression of nuclear respiratory factor 1 (NRF1; Shin et al., 2011). However, it is unclear whether the pathogenic parkin substrates AIMP2 and PARIS are involved in dopaminergic integrity during normal aging and longevity.

Akt (also known as protein kinase B) is a serine/threonine protein kinase involved in cell survival, proliferation, and insulin signaling, and its polymorphism is associated with human longevity. It is involved in the survival of midbrain dopamine neurons, and the suppression of Akt1 expression and phosphorylation has been observed in the striatal postmortem brains of patients with PD (Kim et al., 2020). Interestingly, another study showed a maintained Akt1 expression in surviving neurons in the postmortem human brains of the aforementioned patients (Timmons et al., 2009). Akt1 activating small compounds can provide dopaminergic neuroprotection against α-synuclein aggregation and oxidative stress (Kim et al., 2020). It is important to note that preconditioning brief and mild stresses can activate Akt1, which is responsible for conferring resistance to subsequent cytotoxic insults (Kim et al., 2020). Moreover, Akt1 can regulate neurite outgrowth and proper growth cone functions (Cheng et al., 2011; Jin et al., 2018). However, similar to the pathogenic substrates of parkin, the role of Akt1 in midbrain aging and healthy longevity remains largely unexplored.

We herein employed an Endeavor prioritization approach (Tranchevent et al., 2016) to obtain age-associated and potentially midbrain-specific gene candidates. Several genes, including parkin, AIMP2, NRF1, Akt1, and Akt2, showed specific and significant alterations in the midbrains of exceptionally long-lived but healthy mice compared to adult mice, suggesting their roles in dopaminergic neuron longevity. The expression of the survival genes parkin and Akt1 was well maintained in the ventral midbrain of long-lived mice, while their expression was compromised in 24-month-old mice demonstrating normal aging. Significantly, our study has demonstrated that AIMP2-induced cytotoxicity and neurite toxicity could be efficiently prevented through the expression of Akt1, NRF1, and parkin.

## Results

### Long-lived healthy mice have a preserved dopaminergic system

**Figure 1:**
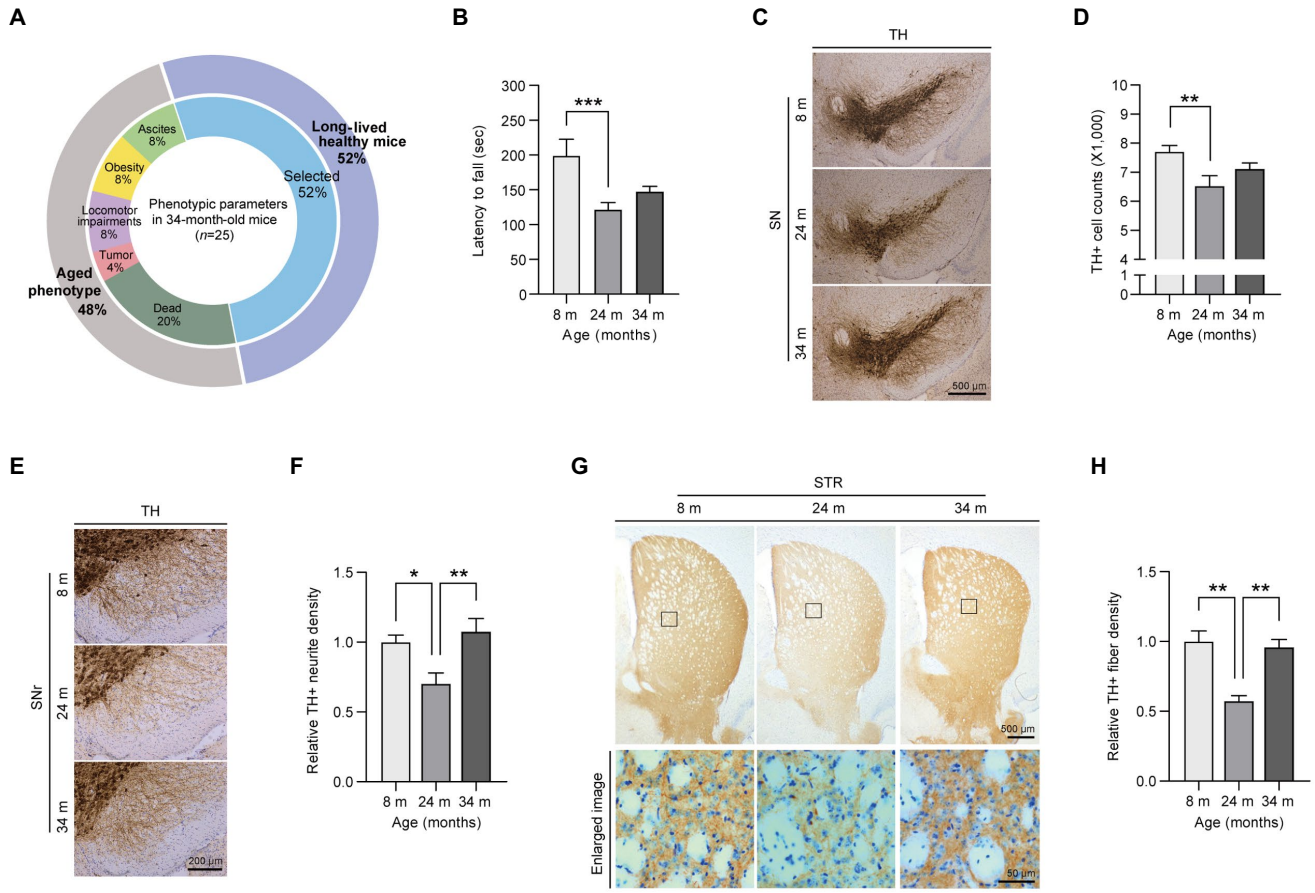
Preserved motor functions and dopaminergic fiber density in exceptionally long-lived mice. **(A)** Phenotypic parameters of long-lived 34-month-old mice. The selected healthy mice (52% of the total long-lived mouse cohort) are used in this study as exceptionally long-lived mice. **(B)** Motorcoordination of 8-, 24-, and 34-month-old mice as determined by measuring latency time on accelerating rotarod tests (*n* = 9 for 8 months; *n* = 13 for 24 months; *n* = 13 for 34 months). **(C)** Representative anti-TH immunohistochemistry images of ventral midbrain sections from 8-, 24-, and 34-month-old mice. Scale bar, 500 μm. **(D)** Stereological assessment of TH-stained dopaminergic neurons in the substantia nigra pars compacta of the mouse groups presented in the left panel (*n* = 13 mice per group). **(E)** Representative anti-TH immunohistochemistry images showing dopaminergic nerve fibers in the substantia nigra reticulata from mice of the indicated age groups. Scale bar, 200 μm. **(F)** Quantification of TH-positive fiber densities in the SN reticularis of the indicated age groups (*n* = 6 mice per group). **(G)** Representative anti-TH immunohistochemistry of the striatum from 8-, 24-, and 34-month-old mice. The lower panel indicates enlarged images of the striatal region (black box in the upper panel). Scale bar, 500 and 50 μm, respectively. **(H)** Quantification of relative dopaminergic axon terminal densities in the striatum of different age groups of mice as determined by anti-TH immunohistochemistry (*n* = 4 for 8 months; *n* = 3 for 24 months; *n* = 4 for 34 months). Quantified data are expressed as the mean ± SEM; *p < 0.05, **p < 0.01, and ***p < 0.001, ANOVA test followed by Tukey’s HSD *post hoc* analysis.

To characterize the long-lived healthy mice, the 34-month-old mice were first grouped into subcategories based on their overt pathogenic phenotypes. Of 25 exceptionally long-lived mice, ~48% exhibited severe aged phenotypes, such as a tumor, locomotor impairments, ascites, and obesity, and 20% died due to aging (Figure 1A). We excluded mice that displayed the abovementioned aged phenotype and subsequently selected 13 healthy individuals for this health-longevity study. Moreover, we analyzed the body weights of mice at three different age stages (Supplementary Figure 1A). As previously described, all male and female mice gained body weight during aging (Yang et al., 2014). Additionally, we also identified the increased expression of the age-and senescence-related markers, poly ubiquitinated proteins and p21 in the midbrains of 24 month and 34 month-old mice (Supplementary Figures 1B,C). But, there was no significant difference in the aging marker expression levels between aged and long-lived mice (Supplementary Figures 1B,C).

The first step in this study was to assess the differences in motor function during aging (Figure 1B). Through locomotor activity measurements using a rota-rod assay, 24-month-old adult mice showed significantly decreased behavioral activity during normal aging compared to young mice, which was consistent with a previous report (Lee et al., 2016; Figure 1B). In contrast, long-lived healthy mice showed a slightly increased pattern without any further decline in locomotor activity compared to aged 24-month-old mice (Figure 1B). To explore the underlying mechanisms of this preserved locomotor activity in long-lived healthy mice, we investigated dopaminergic neuronal integrity, which has been shown to be critically important for fine motor control. A reduction of ~15.3% of dopaminergic neurons was observed in aged 24-month-old mice compared to that in young 8-month-old mice (Figures 1C,D). Surprisingly, long-lived healthy mice showed a trend of increase in dopaminergic neurons without a further decline in dopaminergic neuron survival compared to aged 24-month-old mice (Figures 1C,D). Consistent with dopamine neuron loss, the aged mice exhibited a remarkable 30% decrease in dopaminergic nerve fiber density in the substantia nigra pars reticulata (SNr) regions compared to young mice (Figures 1E,F). Remarkably, dopaminergic neuritic density levels in the SNr of 34-month-old mice were almost equivalent to those in 8-month-old mice (Figures 1E,F). Dopaminergic nerve terminals in the striatum exhibited patterns similar to tyrosine hydroxylase (TH)-positive neurite densities in the SNr of mice of different ages (Figures 1G,H). There was a marked loss of ~50% in dopamine axon terminals in the striatum of 24-month-old mice, while 34-month-old mice maintained almost equivalent dopaminergic terminal densities in the striatum compared to those in 8-month-old mice (Figures 1G,H).

Next, we sought to determine neuropathological alterations in each mouse age group. We observed an increase in neuroinflammatory signatures, including microgliosis and astrogliosis, as reflected by the increased ionized calcium-binding adapter molecule 1 (IBA1) -positive cell soma diameter and enhanced glial fibrillary acidic protein (GFAP)-positive signals in the ventral midbrain of 24-month-old aged mice compared to young 8-month-old mice (Supplementary Figures 1D–G). There was no difference in GFAP neuroinflammatory responses between adult and long-lived healthy mice, while microglial activation was further exacerbated in 34-month-old mice compared to that in 24-month-old mice (Supplementary Figures 1D–G). Taken together, our data demonstrated that long-lived healthy mice exhibited a preserved tendency (pattern) of dopamine neuron survival, enhancement of dopamine neurite densities, and delayed motor decline, even in the presence of a neuroinflammatory environment.

### Midbrain specific potential longevity genes identified by the Endeavor algorithm

**Figure 2:**
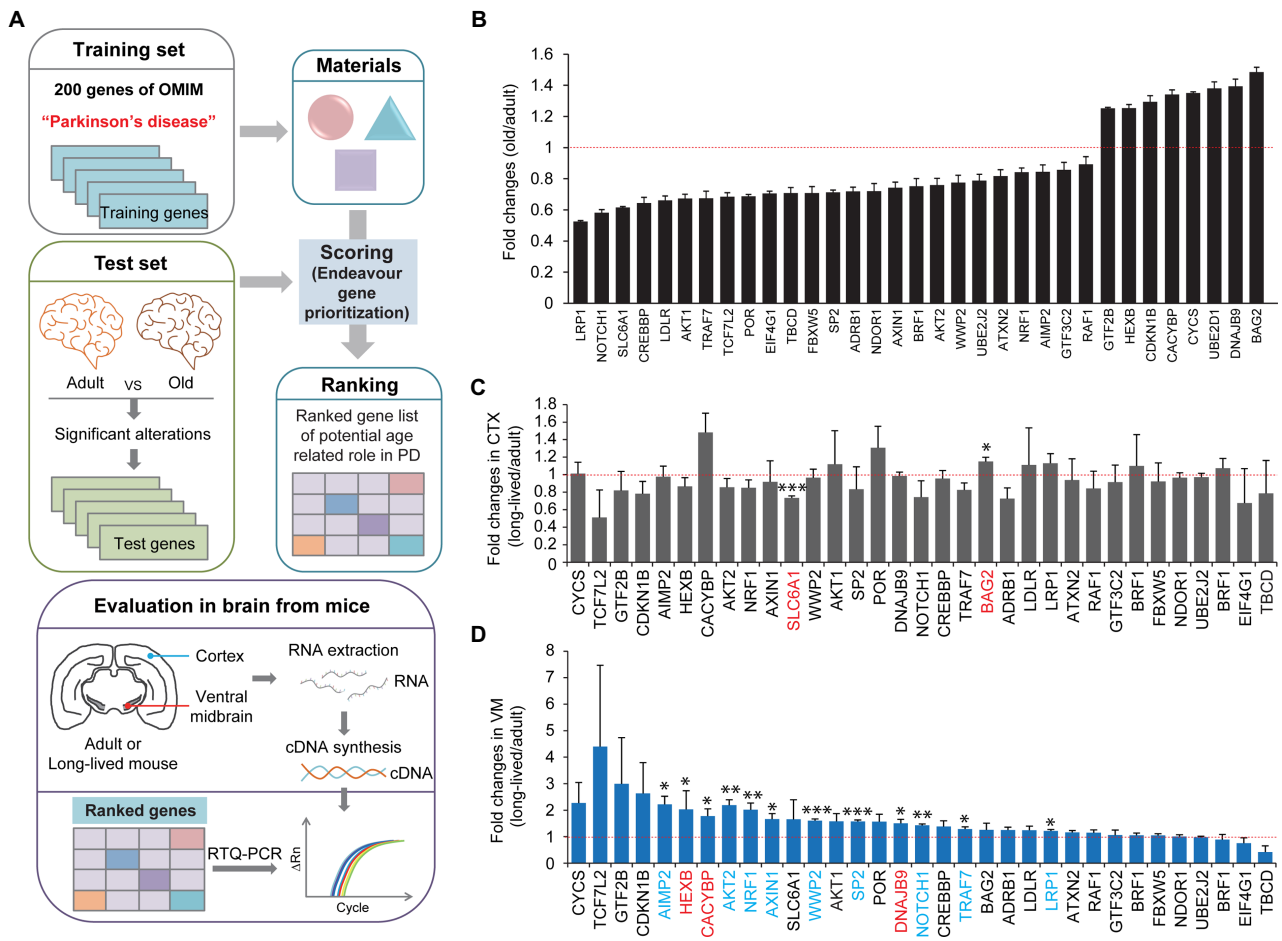
Midbrain specific longevity gene priority analysis. **(A)** A schematic flow chart showing the strategies applied to identify longevity-related genes responsible for motor maintenance in exceptionally long-lived healthy mice. A list of genes with significant alteration in the prefrontal cortex during human aging (group with an average age of 40 years vs. group with an average age of 70 years, *n* = 6 per group) was obtained by a reanalysis of the previous report (Jaffe et al., 2015; Supplementary Table 1). The list of genes altered significantly during human brain aging process was analyzed and ranked by the Endeavor gene prioritization method (Tranchevent et al., 2016), which was pretrained using 200 genes of OMIM obtained using the term “Parkinson’s disease.” High ranked genes were further evaluated by rt-qPCR in the brain subregions (CTX and ventral midbrain) of 8-month-old and 34-month-old healthy mice. **(B)** List of high-ranked genes from the Endeavor prioritization of these age-associated genes with significant alterations in prefrontal cortex human brain regions, ranging from adult to old age groups (*n* = 6 per group, reanalyzed using data set published in Jaffe et al. (2015)). **(C)** Quantification of relative messenger RNA fold changes for the selected genes in the cortex subregions from 8-month-old to 34-month-old mice (*n* = 3 per group). **(D)** Quantification of relative messenger RNA fold changes for the selected genes in the midbrain subregions from 8-month-old to 34-month-old mice (*n* = 3 per group). Quantified data are expressed as mean ± SEM *p < 0.05, **p < 0.01, and ***p < 0.001, unpaired two-tailed Student’s *t*-test.

We screened and evaluated the potential longevity genes responsible for preserved motor function in extremely long-lived mice. To obtain candidate genes for evaluation, we first employed the Endeavor algorithm to prioritize age-related genes associated with midbrain function (Figure 2A). Endeavor models in several processes of interest (gene ontology, interactions, text mining, and so on) were generated by entering 200 genes selected from online Mendelian inheritance in man (OMIM) with the term “Parkinson’s disease.” We chose PD because midbrain function is relatively and selectively impaired in this brain disorder. A list of age-related genes was obtained from the reanalysis of the publicly available human brain RNA-seq database (Jaffe et al., 2015; Supplementary Table 1). By comparing adult (average age, 40 years) and old (average age, 70 years) human brain transcriptomes, genes with significant alterations were selected as age-associated candidate genes (Figure 2B; Supplementary Table 1). A Cytoscape functional analysis of these age-associated genes revealed gene functional clusters with gene enrichment in several pathways, including neurodegenerative disease, signaling pathways regulating the pluripotency of stem cells, melanogenesis, ribosome biogenesis, and so on (Supplementary Figure 2; Supplementary Table 2). The age-associated candidate genes were applied to the Endeavor models, scored, and ranked in each process of interest. A total of 33 high ranked genes were shown with their fold changes in mRNA expression between adult and old human brains (Figure 2B; Supplementary Table 3).

We subsequently investigated which genes were indeed selectively altered in the cortex and ventral midbrain of extremely long-lived mice (34-month-old but healthy mice) compared to 8-month-old mice. We assumed that the *in silico* analysis of PD-associated age-related genes might be differentially regulated in extremely long-lived mice to counteract the normal aging process. Therefore, we extracted total RNA from the ventral midbrain and cortex of adult and long-lived mice and analyzed the relative abundance of the selected target genes using a real-time quantitative polymerase chain reaction (PCR) analysis (Figure 2A). We only found two significantly altered genes, that is, *SLC6A1*, and *BAG2,* in the cortex of long-lived mice compared to adult mice (Figure 2C). There was a modest increase in *BAG2*, whereas *SLC6A1* decreased by ~30% in the long-lived mouse cortex compared to that in the adult cortex (Figure 2C). Intriguingly, there were 12 genes with a significant increase in their expression in the ventral midbrain of long-lived mice compared to that in adult mice (Figure 2D). Interestingly, several significantly altered genes in the ventral midbrain of long-lived mice (i.e., *AIMP2, AKT2, NRF1, AXIN1, WWP2, SP2, NOTCH1, TRAF7*, and *LRP1* [colored in blue]) were changed in the opposite directions of the pattern of gene expression changes in human cortex normal aging (Figure 2B). Notably, *AIMP2, AKT2*, and *NRF1* displayed the most robust changes in expression, indicating a potentially strong contribution to counteraging function.

### Profiling of potential neuro-survival gene expressions in the ventral midbrain of long-lived mice

**Figure 3:**
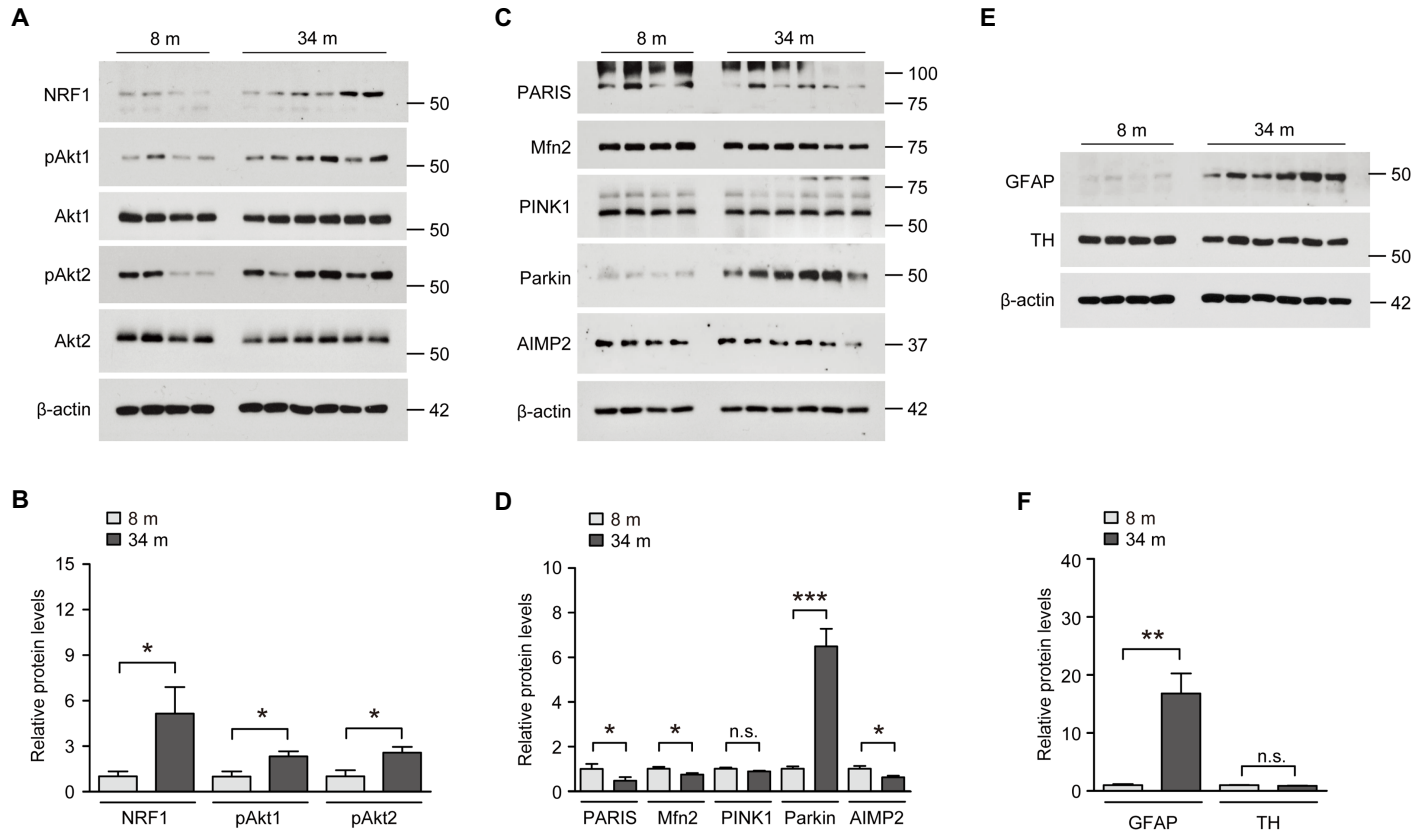
Upregulation of neuroprotective NRF1, parkin, and phosphorylated Akt in the midbrain of exceptionally long-lived healthy mice. **(A)** Representative western blots of NRF1, pAkt1, Akt1, pAkt2, and Akt2 in the midbrain of mice at 8 and 34 months of age using the indicated antibodies. β-actin was used as an internal loading control. **(B)** Quantification of the relative expression levels of the indicated proteins in the midbrain of mice aged 8 and 34 months normalized to β-actin (*n* = 4 mice for 8 months; *n* = 6 mice for 34 months). **(C)** Representative western blots of PARIS, Mfn2, PINK1, parkin, and AIMP2 in the midbrain of mice aged 8 and 34 months using the indicated antibodies. β-actin was used as an internal loading control. **(D)** Quantification of the relative expression levels of the indicated proteins in the midbrain of 8-month-old and 34-month-old mice normalized to β-actin (*n* = 4 mice for 8 months; *n* = 6 mice for 34 months). **(E)** Representative western blots of GFAP and TH in the midbrain of mice aged 8 and 34 months using the indicated antibodies. β-actin was used as an internal loading control. **(F)** Quantification of the relative expression levels of the indicated proteins in the midbrain of mice aged 8 and 34 months normalized to β-actin (*n* = 4 mice for 8 months; *n* = 6 mice for 34 months). Quantified data are expressed as mean ± SEM; *p < 0.05, **p < 0.01, and ***p < 0.001, unpaired two-tailed Student’s *t*-test.

We examined the actual protein expression of these potential midbrain-specific longevity genes that displayed the most robust alterations, indicating a counteraging function. First, the protein expression of NRF1, which is known to function in antioxidant defense and neuronal survival, was markedly increased in the ventral midbrain of long-lived mice compared to that in adult 8-month-old mice (Figures 3A,B). This result is consistent with NRF1 mRNA expression, as determined by a realtime quantitative PCR (rt-qPCR) analysis. Since Akt2 mRNA expression was increased in the ventral midbrain of long-lived mice, we subsequently examined protein expression and phosphorylation as indicators of the kinase activation of Akt1 and Akt2. The protein expression levels were not altered, but the phosphorylation of both Akt1 and Akt2 were increased by more than two times in the ventral midbrain of long-lived mice compared to those in adult 8-month-old mice (Figures 3A,B). The alteration of NRF1 and Akt phosphorylation is ventral midbrain-specific because the cortex failed to show any robust expression changes in these proteins (Supplementary Figure 3A). Compared to the increased AIMP2 mRNA levels in the long-lived mouse ventral midbrain, there was a reduction of ~38% in AIMP2 protein expression, as determined by a western blot analysis (Figures 3C,D). To address the discrepancy in AIMP2 mRNA and protein expression, we monitored the upstream pathways that regulate AIMP2 stability (Supplementary Figure 3B). AIMP2 is a pathological substrate of the E3 ubiquitin ligase, parkin. Interestingly, parkin expression was markedly enhanced in the ventral midbrain of long-lived mice, which was consistent with the suppression of AIMP2 protein expression despite increased AIMP2 transcription. Since parkin and PINK1 are reported to regulate common substrates (Supplementary Figure 3B), such as PARIS and mitofusin-2 (Mfn2), these protein expressions were also examined. They showed a marked reduction of PARIS and a modest reduction of Mfn2 in the absence of changes in PINK1 expression (Figures 3C,D). In contrast, parkin expression appeared to be reduced in the cortex of 34-month-old mice, with no remarkable alterations in the expression of its substrates AIMP2 and PARIS compared to 8-month-old mice (Supplementary Figure 3C). Consistent with the enhanced expression of neuro-survival genes (parkin, phospho-Akt, and NRF1) and fairly preserved dopamine neurons and fiber density in long-lived mouse brains, the protein expression of the dopamine neuron marker TH was comparable between the brains of long-lived and 8-months-old mice, even in the presence of an increase in GFAP expression, indicative of astrogliosis (Figures 3E,F).

### Parkin inactivation and pathologic substrate accumulation in normally aged ventral midbrains

**Figure 4:**
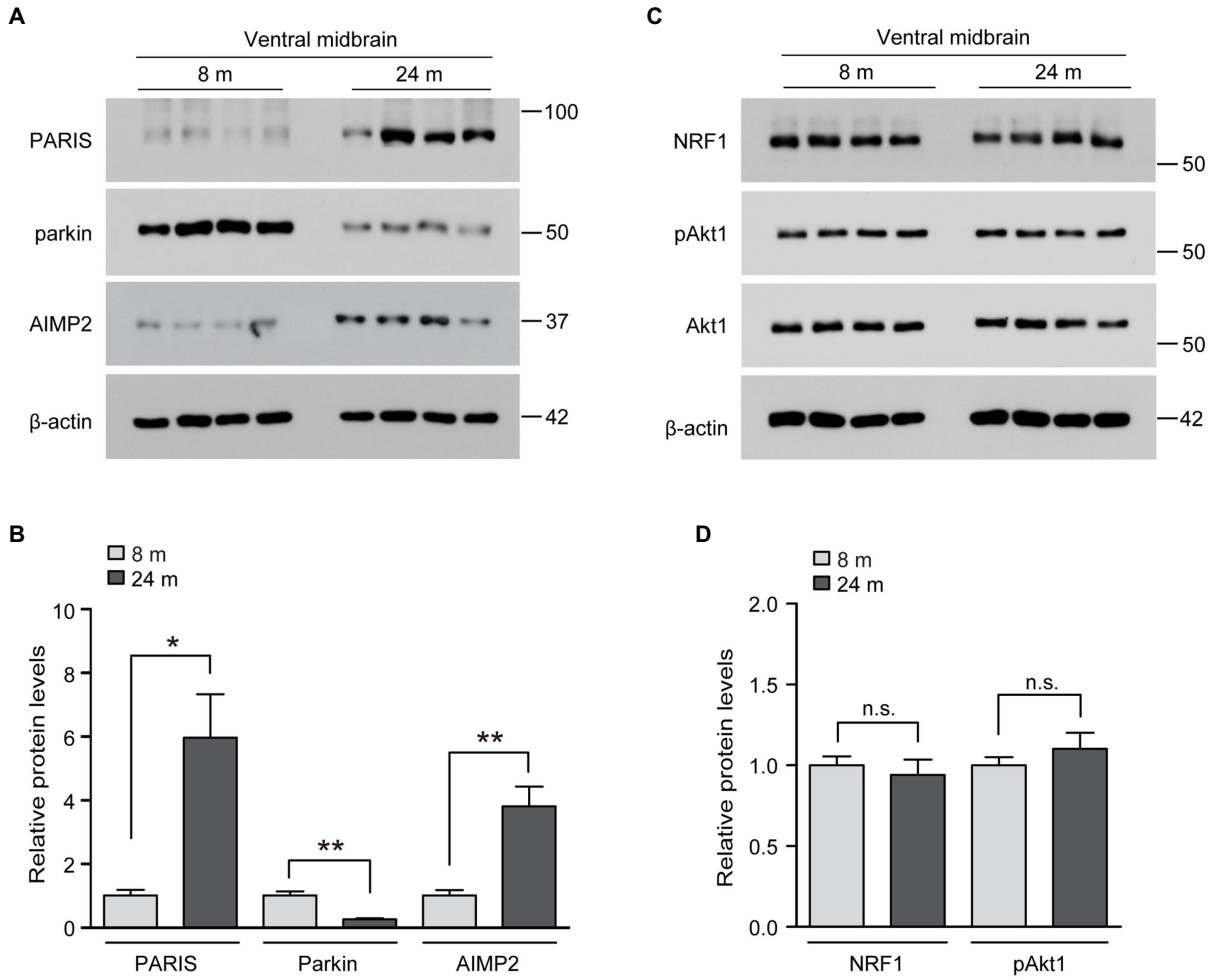
Regulation of longevity-related genes in the midbrain of aged mouse brains. **(A)** Representative western blots of PARIS, parkin and AIMP2 in the midbrain of mice aged 8 and 24 months using the indicated antibodies. β-actin was used as an internal loading control. **(B)** Quantification of the relative expression levels of the indicated proteins in the midbrain of mice aged 8 and 24 months normalized to β-actin (*n* = 4 mice per group). **(C)** Representative western blots of NRF1, pAkt1 and Akt1 in the midbrain of mice aged 8 and 24 months using the indicated antibodies. β-actin was used as an internal loading control. **(D)** Quantification of the relative expression levels of the indicated proteins in the midbrain of 8-month-old and 24-month-old mice normalized to β-actin (*n* = 4 mice per group). Quantified data are expressed as mean ± SEM *p < 0.05 and **p < 0.01, unpaired two-tailed Student’s *t*-test; n.s., non-significant.

We wondered whether these unexpected inductions of several neuro-survival pathways in the brains of long-lived mice are distinct and differentiated from the brains of normally aged mice. Indeed, parkin expression has been reported to decrease in the ventral midbrain of aged mice, with a mild loss of dopaminergic neurons (Lee et al., 2016). Consistent with this previous report, parkin expression was markedly reduced in the ventral midbrain of aged mice in the present study (Figures 4A,B). Correlating with reduced parkin expression, the pathologic parkin substrates AIMP2 and PARIS accumulated during the aging process of the ventral midbrain (Figures 4A,B). The neuro-survival genes elevated in the ventral midbrain of long-lived mice (NRF1, Akt phosphorylation) did not change in normally aged 24-month-old mice compared to those in 8-month-old mice (Figures 4C,D). Taken together, the parkin pathway, NRF1, and Akt phosphorylation, which are critical for dopamine neuron survival, are differentially regulated during normal aging and longevity.

### Parkin, NRF1, and Akt1 prevent AIMP2-induced cytotoxicity and neurite toxicity

**Figure 5:**
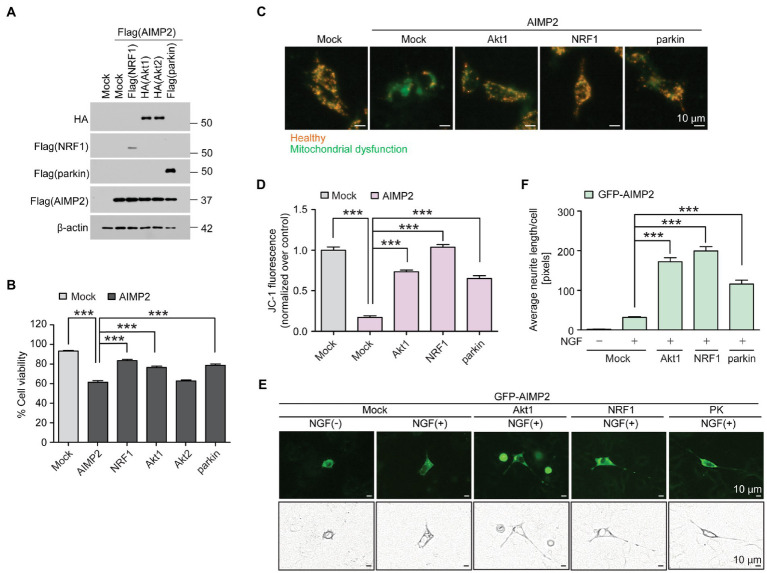
AIMP2 toxicity is inhibited by longevity-related and neuroprotective Akt1, NRF1, and parkin expression. **(A)** Confirmation of protein expression in SH-SY5Y cells transiently transfected (48 h) with Flag-tagged NRF1, parkin, AIMP2, HA-tagged Akt1, and Akt2 determined by a western blot using the indicated antibodies. **(B)** Cell viability assessment for SH-SY5Y cells expressing AIMP2 alone or in combination with NRF1, Akt1, Akt2, and parkin (48 h) determined by a trypan blue exclusion assay (*n* = 6 per group). **(C)** Functional assessment of mitochondrial membrane potential for SH-SY5Y cells transiently transfected with the indicated constructs (48 h) determined by fluorescence reading using a JC-1 dye. Red and green fluorescences reflect healthy and damaged mitochondria, respectively. Scale bar = 10 μm. **(D)** Relative mitochondrial red/green fluorescence ratio in SH-SY5Y cells transiently transfected with the indicated constructs (48 h) and stained with a JC-1 dye (*n* = 13 images from 3 experiments per group). **(E)** Representative fluorescence images, paths of cell body, and neurites of the differentiated (+NGF, 42 h) PC12 cells expressing GFP-tagged AIMP2 in combination with constitutively active Akt1, NRF1, or parkin (48 h). Scale bar, 10 μm. **(F)** Quantification of the relative average lengths of the neurites from differentiated PC12 cells expressing the indicated combination of proteins (*n* = 30 cells per group in 5 experiments per group). Scale bar = 10 μm. Quantified data are expressed as mean ± SEM ***p < 0.001, ANOVA test followed by Tukey’s HSD *post hoc* analysis.

We sought to determine whether there was a functional interaction between previously known neuroprotective genes (NRF1, parkin, and Akt) and pathologic AIMP2. SH-SY5Y cells were transfected with FLAG-AIMP2, with or without co-transfection with FLAG-NRF1, HA-Akt1, HA-Akt2, and FLAG-parkin (Figure 5A; Supplementary Figure 4A). The exogenous expression of each transfected construct was confirmed by western blotting using antibodies against FLAG (AIMP2, NRF1, parkin) or HA (Akt1 and Akt2; Figure 5A). Western blot analysis using antibodies specific for each protein (Akt1, Akt2, NRF1, parkin, and AIMP2) revealed robust overexpression levels of the indicated exogenous proteins in SH-SY5Y cells by the corresponding construct transfection (Supplementary Figure 4A). A trypan blue exclusion cell viability analysis revealed a cytotoxicity rate of ~40% induced by AIMP2 expression in SH-SY5Y cells (Figure 5B). AIMP2-induced cytotoxicity was markedly prevented by the co-expression of NRF1, Akt1, and parkin (Figure 5B). However, Akt2 expression failed to prevent AIMP2-induced cell death (Figure 5B). As another indicator of cytotoxicity, JC-1 imaging was employed to monitor the mitochondrial membrane potential in SH-SY5Y cells with the transient expression of AIMP2, together with longevity-associated neuroprotective genes. AIMP2 expression resulted in the impairment of mitochondrial potential, whereas Akt1, NRF1, and parkin co-expression prevented this AIMP2-induced mitochondrial toxicity (Figures 5C,D).

The characteristic feature found in long-lived mice with relatively well-sustained motor function at that age was the surprising preservation of dopaminergic nerve terminals. Therefore, we investigated whether AIMP2 accumulation could lead to neurite toxicity and whether the longevity of genes could counteract this. PC12 cells transfected with the GFP construct were differentiated using nerve growth factor (NGF) treatment to produce extensive neurite outgrowth (Supplementary Figures 4B,C). GFP-AIMP2 expression almost completely blocked neurite outgrowth (Figures 5E,F), indicating a potential axonopathy induced by AIMP2 expression. The co-expression of Akt1, NRF1, and parkin substantially recovered neurite outgrowth in PC12 cells, even in the presence of AIMP2 expression. Particularly, Akt1 and NRF1 showed greater protective effects in preventing AIMP2-induced neurite toxicity, even when compared to the previously known AIMP2 regulator, parkin (Figures 5E,F). Taken together, we have shown that longevity-associated genes, such as Akt1, NRF1, and parkin, functionally suppress AIMP2-induced mitochondrial dysfunction, neurite impairment, and cytotoxicity.

The biological role of these longevity-associated genes was further investigated by using lentiCRISPR-mediated gene ablation in PC12 cells. Transfection of lentiCRISPR constructs targeting those longevity-associated genes (gAkt1, gNRF1, and gparkin) led to a marked suppression of the target protein expression (Supplementary Figures 4D–I). The genetic ablation of Akt1, NRF1, or parkin in PC12 cells resulted in an ~70% impairment of mitochondrial membrane potential as compared to gControl-transfected PC12 (Supplementary Figures 4J,K). Moreover, deletion of these longevity-associated genes in PC12 cells substantially hampered the neurite outgrowth from PC12 cells during NGF-induced differentiation (Supplementary Figures 4L,M). These gRNA-mediated gene knockout experiments support the role of longevity-associated proteins in the maintenance of mitochondrial function and neurite structure in PC12 cells.

## Discussion

In this study, we first found that exceptionally long-lived healthy mice showed sustained locomotor activity compared to 24-month-old aged mice and had a conserved dopaminergic system. Many studies have examined motor function deficiency in neurodegenerative disease models, including PD and Huntington’s disease models (Bates et al., 1997; Lee et al., 2002), but there has been little research on the alteration of motor function during normal aging and the underlying cellular and molecular mechanisms. Previous studies have shown that motor impairment might be correlated with dopaminergic neuronal loss in movement disorder models (Lee et al., 2013), and a decrease in dopaminergic neuron and dopamine transporter levels during normal aging has also been reported previously (Noda et al., 2020). Consistent with this notion, normal aging for up to 24 months reduced nigral dopamine neurons and dopamine neurite densities in both SNr and striatum. Strikingly, the exceptionally long-lived healthy mice somehow preserved their motor functions with maintained TH expression, dopamine neuron survival, and enhanced TH fiber arborization. It is even more surprising that there was sustained neuroinflammation in the ventral midbrain of these exceptionally long-lived healthy mice, while neurite densities were maintained at levels comparable to those of 8-month-old mice. This finding suggests that the mouse group of healthy longevity somehow adapted to the aging-associated neuroinflammatory environments and as such preserved dopaminergic viability and function. Our finding also suggests that behavioral phenotypes other than motor function could be also preserved in these exceptionally long-lived but healthy mice. Further studies in other brain regions and organs of the long-lived mice would expand our understanding of the longevity-associated genetic alterations in multiple organ systems. It would be also informative to compare gender difference in aging and longevity.

Our approach to screen midbrain longevity-associated genes was based on Endeavor gene prioritization, enabling profiling and ranking across multiple genomic data sources related to the 200 training genes obtained from the OMIM term “Parkinson’s disease.” Indeed, according to previous studies, there is a remarkable overlap between cellular aging hallmarks (i.e., proteostasis, oxidative stress, DNA damage, epigenome, metabolism, and inflammation) and genetic alterations in both aging and neurodegenerative diseases (Glaab and Schneider, 2015). Despite some sample variations possibly attributable to brain subregion dissection errors or 34 month-long maintenance of mice, several high-ranked genes in our Endeavor gene prioritization analysis were still markedly altered at the transcript level, specifically in the midbrains of extremely long-lived mice, again supporting the notion that genetic alterations in the brain are region-specific. Although we narrowed down our subsequent molecular analysis to the candidate genes associated with cell survival, it would provide molecular insights into midbrain longevity and functional preservation to study longevity candidate genes, including AXIN1, WWP2, SP2, DNAJB9, NOTCH1, TRAF7, and LRP1, whose expressions were significantly elevated in our analysis. It is also interesting to note that *SLC6A1* and *BAG2* were differentially expressed specifically in the cortex but not in the midbrain. Since these extremely long-lived mice displayed a healthy appearance, these genes are possibly associated with functional modulation in the cortex regions.

AIMP2 has been extensively studied in the context of the pathogenesis of PD. AIMP2 accumulation due to parkin inactivation contributes to dopaminergic neuron loss *via* PARP1 activation and α-synuclein aggregation *via* self-amyloid-like oligomerization and interaction with α-synuclein (Ham et al., 2020). A recent exome data analysis for dominant late-onset PD families and validation analysis of 1,542 independent patients with PD identified numerous AIMP2 variants associated with PD (Gialluisi et al., 2021), first supporting the clinical genetic association of AIMP2 with PD. In this study, we first identified the parkin substrate, AIMP2, as a longevity-associated gene. Interestingly, the AIMP2 transcript levels increased in the ventral midbrain of extremely long-lived mice. The transcriptional regulation of the promoter of AIMP2 during aging needs to be further investigated because this signaling pathway could be a potential target to inhibit AIMP2 toxicity on cell survival, mitochondrial function, and neurite structure. AIMP2 accumulation in the midbrain correlates well with dopaminergic neurite density in both SNr and striatum. Preventing AIMP2 protein accumulation in the midbrain of extremely long-lived mice, even in the presence of higher AIMP2 transcripts, could be mainly due to its cognate E3 ligase parkin expression, whose levels were markedly reduced in the brain of aged 24-month-old mice. Consistent with this notion, the age-associated downregulation of parkin in 24-month-old mice was accompanied by AIMP2 accumulation. Supporting the functional activation of these elevated levels of parkin, the levels of another pathogenic parkin substrate, PARIS, also decreased with the corresponding elevation of the PARIS target gene, NRF1. Although our molecular analysis focused on AIMP2 toxicity, it is still possible that there are changes in the expression of numerous genes with potential adverse effects on dopamine neurons. This is definitely true owing to a strong and persistent inflammatory signature in the ventral midbrains of extremely long-lived mice. There would be a tug of war between the cell survival gene network and the age-associated cytotoxic pathways. Since the pathological roles of glial inflammation and aging in neurodegeneration has been revealed previously (Bussian et al., 2018; Yun et al., 2018), detailed analysis of molecular signatures of both glial inflammation and glial senescence would be instructive to better understanding of glia–neuron interaction during aging and longevity.

Dopaminergic axonopathy occurs during both normal brain aging and PD pathogenesis, especially at an early stage prior to substantial dopaminergic neuron death (Burke and O'Malley, 2013). Proper functional circuit preservation of the nigrostriatal pathway should rely on dopaminergic nerve terminals that produce neurotransmitter dopamine and innervate striatal medium spiny neurons. When we assume that the ongoing aging process was continuously imposed, even in extremely long-lived mice who lived up to 34 months of age, it is surprising seeing that these mice preserved a motor function slightly better than the 24-month-old mice. This preservation of motor ability is likely due to extensive dopaminergic axonal branching and growth, and TH protein expression, which is required for dopamine synthesis in extremely long-lived healthy mice. It is uncertain which molecular pathway contributes to the prevention or restoration of axonal loss, which is associated with aging. Notably, we have shown in a functional interaction study in differentiated PC12 cells that AIMP2 accumulation is sufficient to induce neurite shortening. AIMP2 has been shown to increase α-synuclein aggregation (Ham et al., 2020), and this α-synucleinopathy may partly contribute to neurite toxicity in PC12 cells. A functional interaction study in SH-SY5Y cells and PC12 cells revealed that the midbrain longevity-associated gene NRF1 exerted the most potent protection against AIMP2 toxicity compared to Akt1 and parkin. NRF1 is a neuroprotective transcription factor with antioxidant defense functions. A reduction in NRF1 expression has been observed during normal aging. The increase in NRF1 transcription in the midbrain of extremely long-lived healthy mice may contribute to adaptive defense against accumulating oxidative stress during aging and inflammation. Our cell toxicity study also supports the functional role of NRF1 in antagonizing AIMP2 toxicity. It remains to be determined why Akt2, compared to Akt1, has no effect on AIMP2 toxicity. Indeed, Akt2 expression was found to be elevated in the initial RTQ PCR analysis of midbrain tissues from exceptionally long-lived mice. Akt2 may have other protective functions that are irrelevant to the inhibition of AIMP2. Although it has been suggested that enhancing Akt1, NRF1, or parkin could efficiently suppress potential age-associated AIMP2 accumulation and dopaminergic toxicity, this needs to be validated in the brains of aged mice *in vivo* to determine whether axonal loss and age-associated severe phenotypes can be reversed by boosting these genes in a similar manner to those in extremely long-lived mice. Besides, the basal expression of longevity-associated proteins, Akt1, NRF1, and parkin seems to be required for maintenance of normal functioning of mitochondria and neurite integrity. Genetic ablation of these genes in PC12 cells produced cellular aging-associated phenotypes, thereby suggesting that altered expression of these genes could potentially contribute to midbrain aging and motor impairments. Our findings provide new significant molecular pathways that might influence normal aging and exceptionally long-lived healthy aging processes. Based on the longevity-related genes (i.e., Akt1, NRF1, parkin, and AIMP2), potentially effective therapeutics might be exploited for the rejuvenation and cure of age-associated neurodegenerative diseases.

## Materials and methods

### Chemicals, plasmids, and antibodies

The following primary antibodies were used: rabbit antibodies for TH (#NB300-109, 1:5,000 for WB, 1:1,000 for IHC, Novus Biologicals), rabbit antibodies for NRF1 (#ab34682, 1:5,000, Abcam), rabbit antibodies for GFAP (#ab7260, 1:5,000 for WB, 1:2,500 for IHC, Abcam), rabbit antibody for AIMP2 (#10424-1-AP, 1:5,000, Proteintech), rabbit antibody for PARIS (#24543-1-AP, 1:5,000, Proteintech), rabbit antibody for Mfn2 (#9482S, 1:5,000, Cell Signaling Technology), rabbit antibody for PINK1 (#BC100-494, 1:3,000, Novus Biologicals), rabbit antibody for phospho-Akt1(S473) (#9018S, 1:5,000, Cell Signaling Technology), rabbit antibody for phospho-Akt2 (S474) (#8599S, 1:5,000, Cell Signaling Technology), rabbit antibody for IBA1 (#019-19,741, 1:500, Wako), mouse antibody for ubiquitin (#sc-8017, 1:1,000, Santa Cruz), mouse antibody for p21 (#sc-6246, 1:500, Santa Cruz), mouse antibody for parkin (#4211S, 1:5,000, Cell Signaling Technology), mouse antibody for Akt1 (#2967S, 1:5,000, Cell Signaling Technology), mouse antibody for Akt2 (#5239S, 1:5,000, Cell Signaling Technology), horse radish peroxidase (HRP)-conjugated mouse antibody for HA (#2999S, 1:5,000, Cell Signaling Technology), and HRP-conjugated mouse antibody for FLAG (#8592, 1:5,000, Sigma).

The following secondary antibodies were used: HRP-conjugated goat antibody for mouse immunoglobulin (Ig)G (#GTX-213111-01, 1:5,000, Genetex), HRP-conjugated goat antibody for rabbit IgG (#GTX-213110-01, 1:5,000, Genetex), and HRP-conjugated mouse antibody for β-actin (cat# A3854, 1:10,000, Sigma-Aldrich).

### Animal experiments

All animal experiments conducted in this study were approved by the ethical committee of the Daegu Gyeongbuk Institute of Science and Technology (DGIST-IACUC-18040408-02) in accordance with international guidelines. Male and female C57BL/6J mice at the age of 6–8 weeks were obtained from Hyochang Science (South Korea) and housed for up to 34 months in the specific pathogen free (SPF)-animal care facility of DGIST. Experimental mice were randomly grouped, and a maximum of 5 mice have been raised in individually ventilated cages in a barrier room. The housing condition was maintained in a 12:12 h light/dark cycle with controlled temperature (20°C–26°C) and humidity (40%–60%) environments. The gamma-irradiated food and autoclaved water were provided *ad libitum*, and disinfected cages were changed weekly. All mice were categorized as per ages as follows: young (8 months), adult (24 months), and long-lived (34 months).

### Tissue immunohistochemistry

Mice were anesthetized with avertin (250 mg/kg body weight, intraperitoneal injection), a mixture of 2,2,2-tribromoethanol and 2-methyl-2-butanol (Sigma-Aldrich, MO, United States), and perfused with phosphate-buffered saline (PBS). Collected brain tissues were subsequently fixed with 4% paraformaldehyde. Following post-fixation with 4% paraformaldehyde, tissues were cryoprotected with 30% sucrose solution. They were cross-sectioned to a thickness of 40 μm and prepared as free-floating sections. For histological analysis, the primary antibodies used were TH, GFAP, and IBA1. Brain sections were incubated with primary antibodies at 4°C overnight, followed by incubation with biotinylated goat anti-rabbit IgG. Using an ABC kit (Vector Laboratories, CA, United States), the avidin/biotin-based enzymatic reaction was amplified, and the target protein was visualized using 3,3′-diaminobenzidine (DAB, Sigma-Aldrich, MO, United States). Some sections were counterstained with a solution of 0.1% cresyl violet (IHC World, MD, United States) and coverslipped in permanent mounting medium (Biosesang, South Korea). All images of the stained brain samples were acquired using a Zeiss Axio Imager M2 (Carl Zeiss, Germany).

### Quantitative histological analysis

#### Stereological counting of dopaminergic neuron

The total number of TH-positive cells in the substantia nigra pars compacta (SNpc) region was counted using an optical fractionator probe in the Stereo Investigator software (MicroBright-Field, VT, United States). Counting was conducted under ×40 objective and 40 × 40 μm counting frames. All stereological assessments were performed in an unbiased manner.

#### Histological analysis

For TH-positive dopaminergic neuron fiber density assessments, two different brain regions (STR and SNr) were used. In STR, sections between bregma +0.98 and +0.50 mm were used. The substantia nigra (SN) region was used for the GFAP, IBA1-positive cell analysis. IBA1-positive microglia cells were used to measure soma diameter using ImageJ under ×20 objectives. All presented data were normalized to those of young mice for comparison. All images were quantified by optical density using ImageJ software (National Institutes of Health, MD, United States).

### Behavior test

For behavioral assessment, a rotarod test (Ugo Basile, Italy) was performed as previously described, with some modifications (Lee et al., 2016). All mice were trained for adaptation before the test and underwent three trials thereafter. For adaptation, each mouse was placed on a rod that rotated at 5 rpm. Subsequently, the mice were subjected to the rotating rod accelerating from 5 rpm to 40 rpm in 300 s. There was a minimum rest period of 15 min between trials. The assessment was performed once a week and repeated for 3 weeks.

### Gene prioritization analysis

Age-related genes associated with PD were prioritized using the Endeavor algorithm (Tranchevent et al., 2016). First, the training gene set was entered by searching 200 genes in OMIM using the term “Parkinson’s disease.” The process of interest in model generation includes annotation (GeneOntology, Kegg, and Swissprot), blast, interaction (Bind, BioGrid, Hprd, InNetDb, Intact, Mint, and String), and text. Age-related candidate genes were selected from the reanalysis of significantly altered genes in the human prefrontal cortex between the adult and aged groups (Jaffe et al., 2015). All age-related candidate genes were applied to the generated models and ranked in each process of interest. The global ranking of candidate genes was also provided through the integration of each gene score. In the subsequent selection of target genes for the rt-qPCR analysis of mouse brains, we included the top 33 genes in global prioritization.

### Preparation of tissue for immunoblot

After dissecting the subregions of the mouse brain, the ventral midbrain (VM) and cortex regions were used for the following procedures. The dissected mouse brain tissues were homogenized in lysis buffer [1% Nonidet P40 and 0.5% sodium deoxycholic acid in PBS (pH 7.4) and protease/phosphatase inhibitors] using a Diax 900 homogenizer. The homogenized brain lysates were incubated on ice for 30 min, with vortexing every 10 min, and subsequently centrifuged at 14,000×*g* for 30 min.

After centrifugation, the supernatants were collected into new tubes, and the protein levels were quantified using the BCA Protein Assay Kit (Pierce) with bovine serum albumin standards. The proteins obtained through preparation were mixed with a 2X Laemmli buffer (Bio-rad) supplemented with β–mercaptoethanol (Bio-rad) and boiled for 5 min at 95°C. Thereafter, the protein samples were separated by SDS-PAGE and transferred onto nitrocellulose membranes (0.45 μm, #162–0115, Bio-rad) for immunoblotting with the indicated antibodies. The bands on the nitrocellulose membrane were visualized using the SuperSignal West Pico PLUS chemiluminescent substrate (#34579, Thermo Fisher Scientific).

### Plasmids

FLAG-AIMP2, pEGFP-AIMP2, FLAG-Parkin, and pEGFP-N1 plasmids have been previously described (Ko et al., 2010; Lee et al., 2013). The plasmids pMSCV-hyg-FLAG-NRF1 (#34707), pECE-HA-Akt1 (#10841), and pcDNA3-HA-Akt2 (#9016) were purchased from Addgene. Guide RNAs (gRNA) targeting rat Akt1, NRF1, and parkin were cloned into LentiCRISPR v2 vector (Addgene, Plasmid #52961) and validated by sequencing following the protocol provided by the Prof. Zhang’s lab (Shalem et al., 2014). The targeting sequences for each gene of the cloned gRNAs are as follows: gAkt1 = tgatgaagacagagcggccg; gNRF1 = atctatccgaaagagacagc; gparkin = agtggttgctaagcgacagg. lentiCRISPR-EGFP sgRNA1 (Addgene, Plasmid #51760) was used as a gRNA control. pcDNA3-YFP (Addgene, Plasmid #13033) was used to visualize transfected PC12 cells in gRNA-mediated gene knockout experiments.

### RNA extraction from brain tissue

Mouse brain subregion tissues (VM, CTX) were homogenized with 1 ml of a QIAzol Lysis Reagent (cat# 79306, QIAGEN) and subsequently transferred to a new tube. Homogenized samples were incubated for 5 min at room temperature, added to 0.2 ml of chloroform, and vortexed for 15 s. After vortexing, the homogenized samples were incubated for 2–3 min at room temperature and centrifuged at 12,000×*g* for 15 min at 4°C. After centrifugation, the supernatants were transferred to a new tube to isolate the RNA phase and add equal amounts of 100% isopropanol to the aqueous phase. These tubes were incubated for 10 min at room temperature, and the aqueous samples were subsequently centrifuged at 12,000×*g* for 10 min at 4°C. After centrifugation, the supernatant was removed from each tube to leave the RNA pellet. We subsequently washed the pellet with 1 ml of 75% ethanol per tube and discarded the ethanol from the tube. We proceeded to air dry the pellet to completely remove ethanol and re-suspend the RNA pellet in 50 μl of DNase and RNase-free water (#19077-015, Invitrogen).

### rt-qPCR analysis

cDNA was synthesized from the total RNA (1 μg) obtained from the abovementioned procedure using a first-strand cDNA synthesis kit (#170-8891, iScript cDNA synthesis kit, Bio-Rad). The relative abundance of the target mRNA expression was analyzed using real-time PCR (QuantStudio 6 flex Real-Time PCR System, Applied Biosystems) with a SYBR Green PCR master mix (#4309155, Applied Biosystems). The relative mRNA expression levels of the target genes were calculated using the ΔΔCt method, with Glyceraldehyde 3-phosphate dehydrogenase (GAPDH) as an internal loading control. TH primer sequences used in this study are listed in Supplementary Table 4.

### Cell culture and transfection

Human neuroblastoma SH-SY5Y cells (ATCC) were grown in Dulbecco’s modified Eagle’s medium (DMEM, #11995073, GIBCO) with 10% fetal bovine serum (FBS, vol/vol, #26140079, GIBCO) and a penicillin–streptomycin antibiotic solution (100 U/mL, #P4333, Sigma-Aldrich). PC12 cells, noradrenergic clonal cells obtained from the adrenal medulla of Rattus norvegicus (ATCC), were grown in DMEM (#11995073, GIBCO) containing 10% FBS (vol/vol, #26140079, GIBCO), 5% Horse serum (vol/vol, #26050-088, GIBCO), 2 mM Glutamine (#G7513, Sigma-Aldrich), and penicillin–streptomycin antibiotic solution (100 U/mL, #P4333, Sigma-Aldrich). Both types of cells were grown at 37°C in a humidified atmosphere consisting of 5% CO_2_/95% air. X-tremeGENE HP transfection reagents (#6366546001, Roche) were used for transient transfection, following the manufacturer’s instructions.

### Cell viability assay

After plating SH-SY5Y cells in 12-well plates at a density of 0.2 × 10^6^, the cells were transiently transfected with the indicated constructs and grown for 48 h. The cells were harvested and washed twice with PBS, and subsequently centrifuged at 400*g* for 5 min. After centrifugation, the cells were resuspended in PBS, mixed with an equal volume of 0.4% trypan blue (wt/vol), and incubated for 2 min at room temperature. A Countess II Automated Cell Counter (Life Technologies) was used to analyze the proportion of live and dead cells. Alternatively, intracellular differences in mitochondrial membrane potential were measured using a JC-1 mitochondrial membrane potential assay kit (#1009172, Cayman). Fluorescent images from the JC-1 assay were obtained using a fluorescence microscope (Axio Imager M2, Zeiss).

### PC12 neurite length analysis

To differentiate PC12 cells, we removed the complete culture medium and replaced them with a differentiation medium consisting of DMEM (#11995073, GIBCO) with 10% horse serum (vol/vol, #26050-088, GIBCO), 50 ng/mL NGF (#13257-019, Invitrogen), and penicillin–streptomycin antibiotic solution (100 U/mL, #P4333, Sigma-Aldrich). After transient transfection, the cells were incubated for 42 h in a differentiation medium, and fluorescence images were obtained using a fluorescence microscope (Axio Imager M2, Zeiss). For lentiCRISPR-mediated gene ablation experiments, puromycin (Sigma-Aldrich, P8833, 48 h, 1.5 μg/mL) selection and NGF treatment (48 h) were carried out for PC12 cells following gRNA transfection (96 h). The Neuron J software was used to measure the neurite length of each cell.

### Statistics

Quantitative data are presented as the mean ± standard error of means (SEM). Statistical significance was evaluated by an unpaired two-tailed Student’s *t*-test for two-group comparisons and analysis of variance (ANOVA) with Tukey’s HSD *post hoc* analysis for comparisons of more than three groups. A *p*-value < 0.05 was considered statistically significant. GraphPad Prism software version 9.1.0 was used to prepare all data plots and for statistical analyses.

## Data availability statement

The datasets presented in this study can be found in online repositories. The names of the repository/repositories and accession number(s) can be found in the article/Supplementary material.

## Ethics statement

The animal study was reviewed and approved by the ethical committee of the Daegu Gyeongbuk Institute of Science and Technology.

## Author contributions

YL and Y-IL: conceptualization, resources, writing—review and editing, supervision, and funding acquisition. HK, JK, MJ, and Y-JH: methodology and investigation. J-HS, HK, JK, MJ, and Y-JH: formal analysis. HK and JK: data curation. SP and J-YA: resources. HK, Y-JH, Y-IL, and YL: writing—original draft preparation. All authors contributed to the article and approved the submitted version.

## Funding

This study was supported by the Sunken Research Fund, Sungkyunkwan University, 2015 and by grants from the National Research Foundation (NRF-2018R1D1A1B07046762) and ICT (2021M3H4A4079521), which is funded by the Korean Ministry of Science, ICT, and Future Planning (MSIP). This research was also supported by the Bio & Medical Technology Development Program of the National Research Foundation (NRF) funded by the Korean government (MSIT) (2020M3A9D8038653).

## Conflict of interest

The authors declare that the research was conducted in the absence of any commercial or financial relationships that could be construed as a potential conflict of interest.

## Publisher’s note

All claims expressed in this article are solely those of the authors and do not necessarily represent those of their affiliated organizations, or those of the publisher, the editors and the reviewers. Any product that may be evaluated in this article, or claim that may be made by its manufacturer, is not guaranteed or endorsed by the publisher.

## Supplementary material

The Supplementary material for this article can be found online at: https://www.frontiersin.org/articles/10.3389/fnagi.2022.1030807/full#supplementary-material
