# Supplementary material for: Identification and evaluation of midbrain specific longevity-related genes in exceptionally long-lived but healthy mice
Source: Front Aging Neurosci. 2023 Jan 11;14:1030807. doi: 10.3389/fnagi.2022.1030807 (PMC9874112; doi:10.3389/fnagi.2022.1030807)
Supplement: Supplementary file 1 [file Data_Sheet_1.docx]

**Supplementary figures and tables**

**Identification and evaluation of midbrain specific longevity-related genes in exceptionally long-lived but healthy mice**

Hyojung Kim, Yu-Jin Huh, Ji Hun Kim, Minkyung Jo, Joo-Heon Shin, Sang Chul Park, Jee-Yin Ahn, Yun-Il Lee, and Yunjong Lee

**
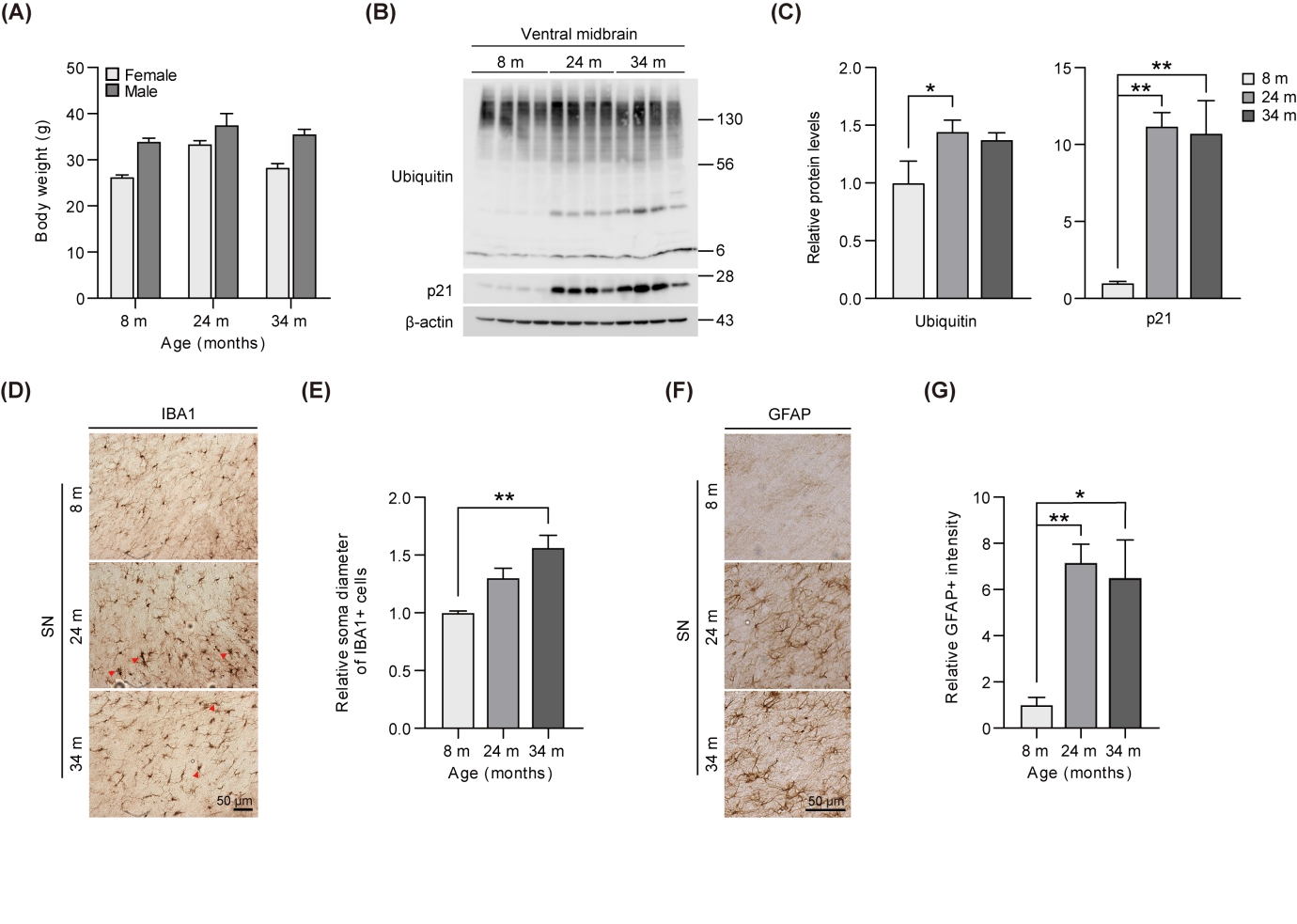
**

**Supplementary figure S1. Phenotypic and neuropathological assessments of aged and long-lived healthy mice**

(A) Body weight of female and male mice at the indicated ages (*n* = 9 mice for 8 months; *n* = 13 mice for 24 months; *n* = 13 mice for 34 months).

(B) Representative western blots of aging- and senescence-related proteins (ubiquitin and p21) in the midbrains from 8-, 24-, and 34-month-old mice. β-actin was used as an internal loading control.

(C) Quantification of relative expression levels of the indicated proteins (ubiquitin and p21) in the midbrains from 8-, 24- and 34-month-old mice normalized to β-actin (*n* = 4 mice per group).

(D) Representative anti-IBA1 immunohistochemistry images of the substantia nigra from 8-, 24-, and 34-month-old mice. Arrowheads indicate activated microglia. Scale bar, 50 μm.

(E) Quantification of the relative soma diameter of the IBA1-positive nigral microglia from 8-, 24-, and 34-month-old mice (*n* = 3 mice per group).

(F) Representative anti-GFAP immunohistochemistry images of SN from 8-, 24-, 34-month-old mice. Scale bar, 50 μm.

(G) Quantification of GFAP-positive cell intensities of indicated age (*n* = 4 mice per group).

Quantified data are expressed as mean ± SEM; **P* < 0.05, and ***P* < 0.01, ANOVA test followed by Tukey’s HSD post hoc analysis.

**
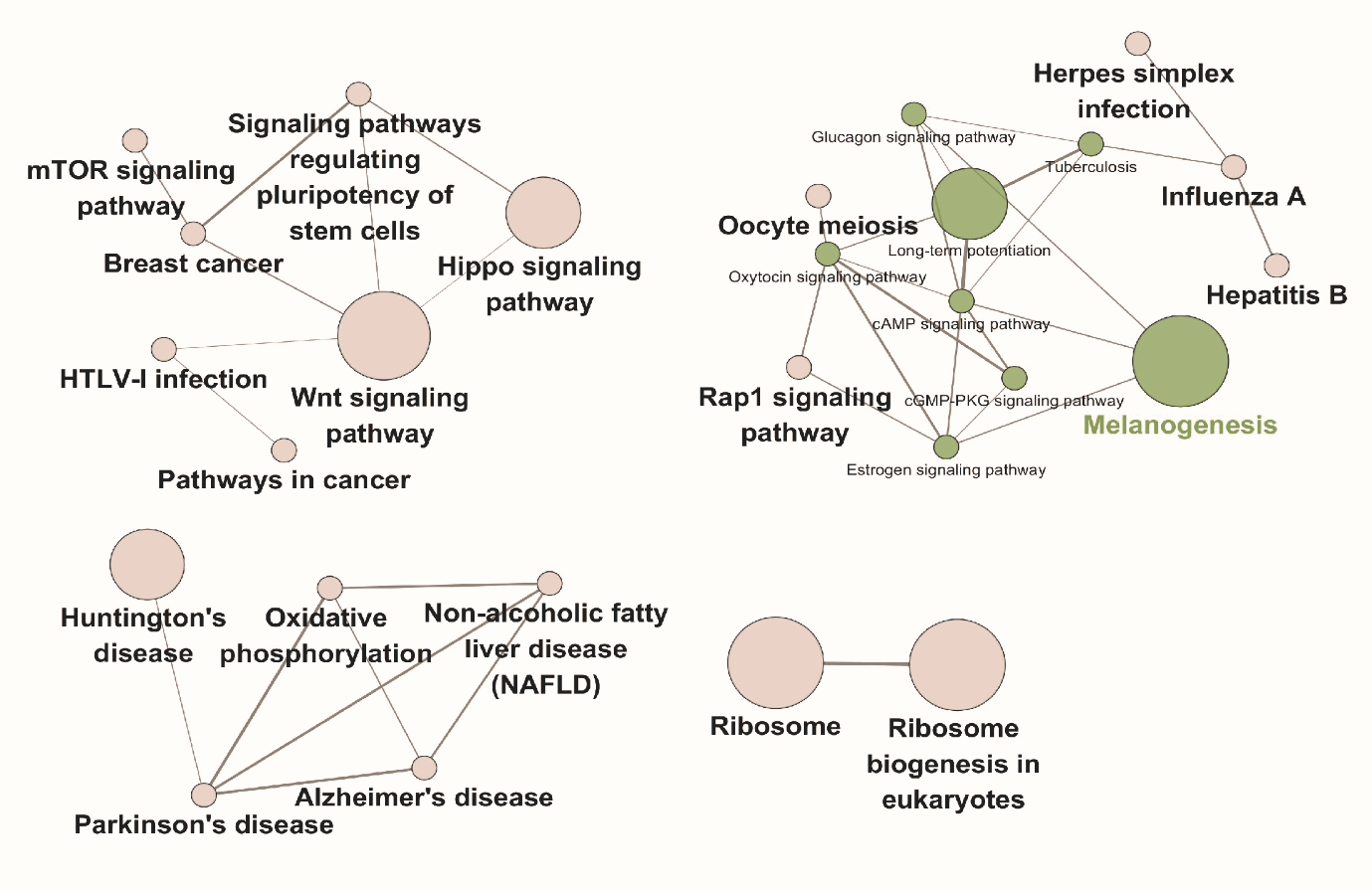
**

**Supplementary figure S2. Functional pathway analysis of age-associated genes during human brain aging**

Cytoscape functional clustering (minimum number of genes for functional grouping = 7) of significantly (p < 0.05) altered genes (1,015 genes) in the human prefrontal cortex between the middle- and old-aged groups (detailed list of functional pathways in Supplementary table 2).

**
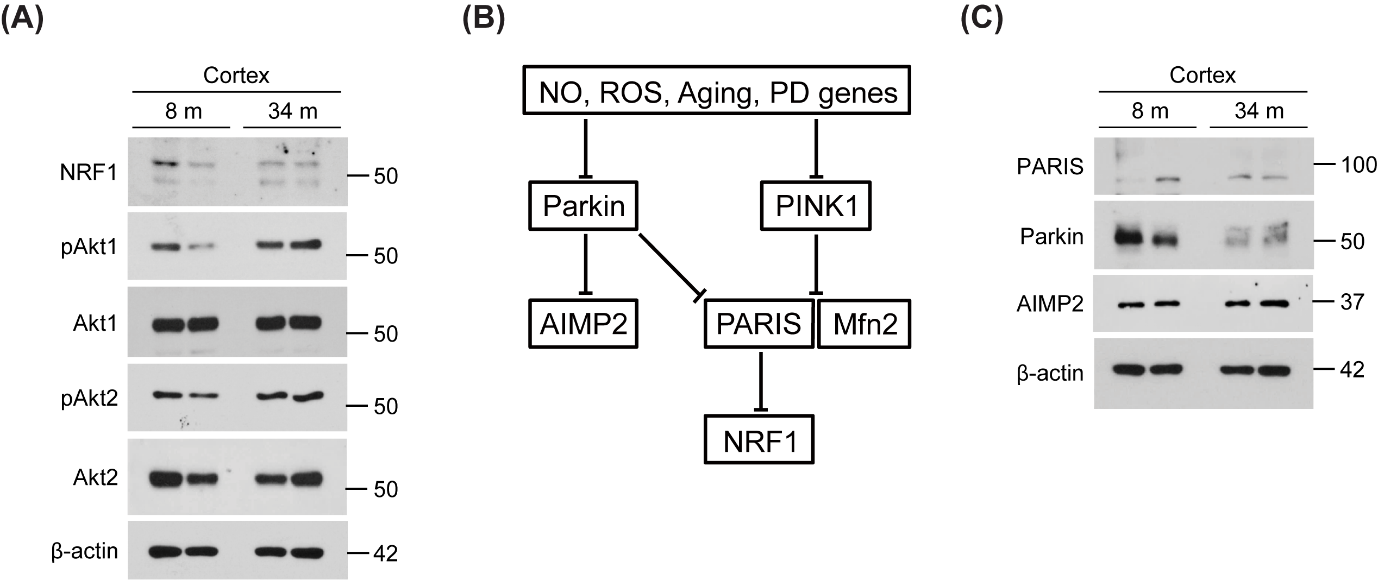
**

**Supplementary figure S3. Expression of selected genes in the cortex of long-lived mouse brains**

(A) Representative western blots of NRF1, pAkt1, pAkt2, total Akt1 and Akt2 expressed in the cortex of 8- and 34-month-old mice using the indicated antibodies. β-actin was used as an internal loading control.

(B) Simplified summary of previously identified parkin and PINK1-related pathological signaling pathways. Catalytic functions of parkin and PINK1 can be compromised by PD-linked mutations or PD associated diverse stresses including nitric oxide (NO), reactive oxygen species (ROS), or aging. Dysfunctional parkin or PINK1 can lead to the accumulation of their target protein substrates such as AIMP2, PARIS, and Mfn2. Too much PARIS can then suppress the expression of NRF1 of antioxidant function.

(C) Representative western blots of PARIS, parkin and AIMP2 expressed in the cortex of 8- and 34-month-old mice using the indicated antibodies. β-actin was used as an internal loading control.

**
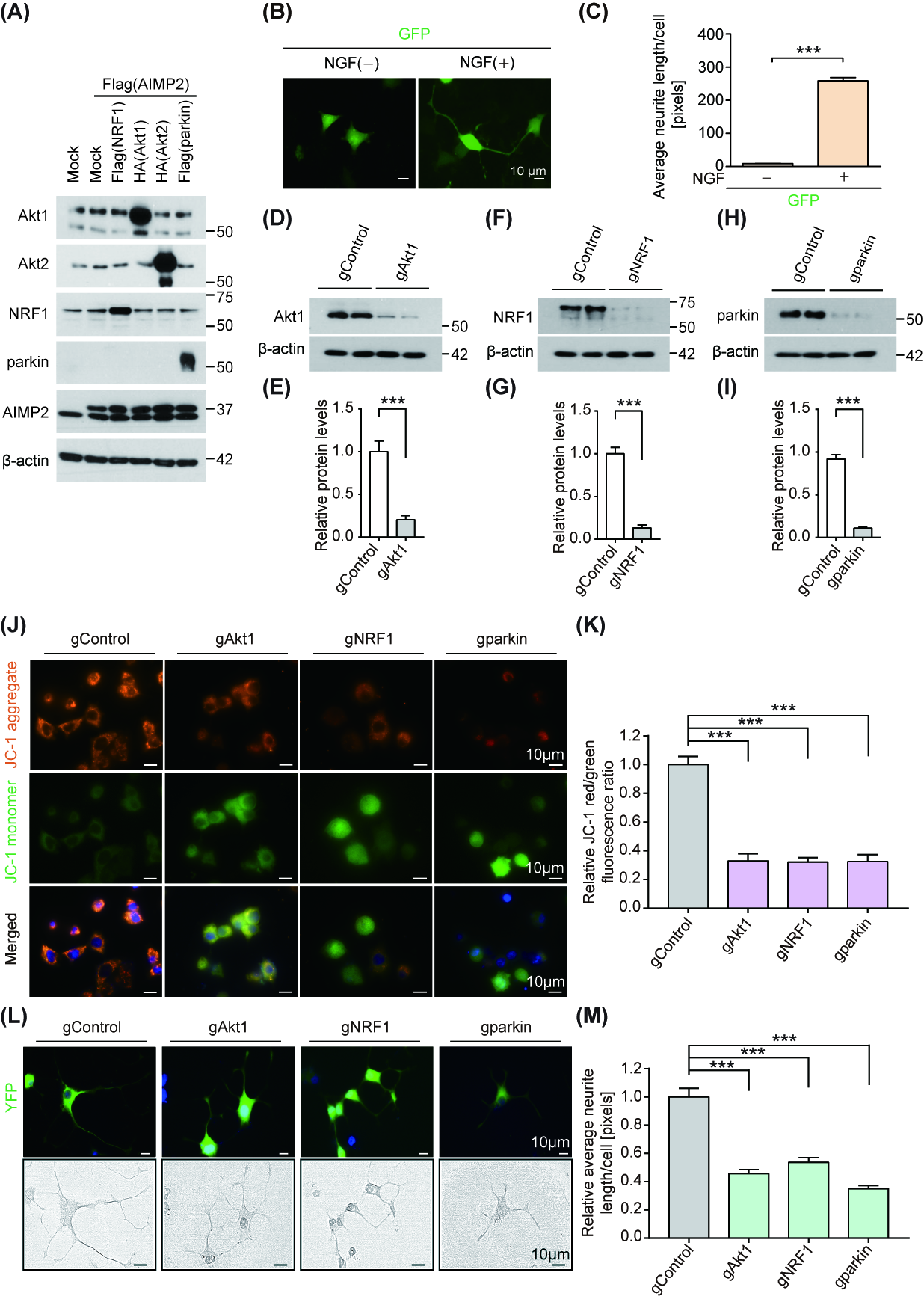
**

**Supplementary figure S4. Evaluation of biological functions of longevity-related proteins in PC12 cells**

(A) Assessment of protein expression in SH-SY5Y cells transiently transfected (48 h) with Flag-tagged NRF1, parkin, AIMP2, HA-tagged Akt1, and Akt2 determined by a western blot using the indicated antibodies.

(B) Representative fluorescence images of undifferentiated (- NGF) or differentiated (+ NGF, 2 days) PC12 cells expressing GFP. Scale bar, 10 μm.

(C) Quantification of neurite lengths for PC12 cells treated with or without NGF (2 days, *n* = 30 cells per group, five experiments).

(D) Expression of Akt1 in PC12 cells transiently transfected (96 h) with LentiCRISPR-cas9 targeting Akt1 (gAkt1) or control gRNA plasmid (gControl) and selected with puromycin (1.5 μg/ml, 48 h) monitored by western blots using anti-Akt1 antibody. β-actin was used as an internal loading control.

(E) Quantification of relative Akt1 expression in the indicated experimental groups normalized to β-actin (*n* = 4 experiments per group).

(F) Expression of NRF1 in PC12 cells transiently transfected (96 h) with LentiCRISPR-cas9 targeting NRF1 (gNRF1) or control gRNA plasmid (gControl) and selected with puromycin (1.5 μg/ml, 48 h) monitored by western blots using anti-NRF1 antibody. β-actin was used as an internal loading control.

(G) Quantification of relative NRF1 expression in the indicated experimental groups normalized to β-actin (*n* = 4 experiments per group).

(H) Expression of parkin in PC12 cells transiently transfected (96 h) with LentiCRISPR-cas9 targeting parkin (gparkin) or control gRNA plasmid (gControl) and selected with puromycin (1.5 μg/ml, 48 h) monitored by western blots using anti-parkin antibody. β-actin was used as an internal loading control.

(I) Quantification of relative parkin expression in the indicated experimental groups normalized to β-actin (*n* = 4 experiments per group).

(J) Functional assessment of mitochondrial membrane potential for PC12 cells transiently transfected with the indicated gRNA constructs (96 h) and selected with puromycin (1.5 μg/ml, 48 h) determined by fluorescence imaging using a JC-1 dye. Red and green fluorescences reflect healthy and damaged mitochondria, respectively. Scale bar = 10 μm.

(K) Relative mitochondrial JC-1 red/green fluorescence ratio in PC12 cells with the indicated treatments (*n* = 6 images from 3 experiments per group).

(L) Representative fluorescence and bright-field images of cell body, and neurites of the differentiated (+ NGF, 48 h) PC12 cells expressing YFP in combination with the indicated gRNA (96 h transfection and 48 h puromycin selection). Scale bar, 10 μm.

(M) Quantification of the normalized relative average lengths of neurites from differentiated PC12 cells with the indicated transfections (*n* = 20 cells from 3 experiments per group). Scale bar = 10 μm.

Quantified data are expressed as mean ± SEM. ****P* < 0.001, unpaired two-tailed Student’s *t*-test for two group comparison or ANOVA test followed by Tukey’s HSD post hoc analysis for three or more group comparison.

.

**Supplementary table 1.** Differentially expressed genes in the human prefrontal cortex during aging

| **Gene symbol** | **Gene Name** | **adult** | **old** | **ratio** | **ttest** |
| --- | --- | --- | --- | --- | --- |
| LOC100132153 | similar to tubulin T beta15 | 10.4454 | 2.5743 | 0.2465 | 0.0315 |
| WNT7B | "wingless-type MMTV integration site family, member 7B" | 2.5660 | 0.6690 | 0.2607 | 0.0188 |
| PCDHGA12 | "protocadherin gamma subfamily C, 3; protocadherin gamma subfamily C, 5; protocadherin gamma subfamily C, 4; protocadherin gamma subfamily A, 12" | 1.0359 | 0.3321 | 0.3206 | 0.0003 |
| PCDHGC5 | "protocadherin gamma subfamily C, 3; protocadherin gamma subfamily C, 5; protocadherin gamma subfamily C, 4; protocadherin gamma subfamily A, 12" | 1.0359 | 0.3321 | 0.3206 | 0.0003 |
| PCDHGC3 | "protocadherin gamma subfamily C, 3; protocadherin gamma subfamily C, 5; protocadherin gamma subfamily C, 4; protocadherin gamma subfamily A, 12" | 1.0359 | 0.3321 | 0.3206 | 0.0003 |
| PCDHGC4 | "protocadherin gamma subfamily C, 3; protocadherin gamma subfamily C, 5; protocadherin gamma subfamily C, 4; protocadherin gamma subfamily A, 12" | 1.0359 | 0.3321 | 0.3206 | 0.0003 |
| SLC6A11 | "solute carrier family 6 (neurotransmitter transporter, GABA), member 11" | 7.8033 | 3.0209 | 0.3871 | 0.0423 |
| SLC29A4 | "solute carrier family 29 (nucleoside transporters), member 4; similar to solute carrier family 29 (nucleoside transporters), member 4" | 12.4424 | 4.8762 | 0.3919 | 0.0233 |
| LOC100132308 | "solute carrier family 29 (nucleoside transporters), member 4; similar to solute carrier family 29 (nucleoside transporters), member 4" | 12.4424 | 4.8762 | 0.3919 | 0.0233 |
| LOC402509 | "solute carrier family 29 (nucleoside transporters), member 4; similar to solute carrier family 29 (nucleoside transporters), member 4" | 12.4424 | 4.8762 | 0.3919 | 0.0233 |
| RNU6-2 | "RNA, U6 small nuclear 2; RNA, U6 small nuclear 1" | 2.2388 | 0.8852 | 0.3954 | 0.0145 |
| RNU6-1 | "RNA, U6 small nuclear 2; RNA, U6 small nuclear 1" | 2.2388 | 0.8852 | 0.3954 | 0.0145 |
| P2RY1 | "purinergic receptor P2Y, G-protein coupled, 1" | 1.4092 | 0.5625 | 0.3991 | 0.0274 |
| NCAN | neurocan | 38.4210 | 15.4501 | 0.4021 | 0.0045 |
| SHISA6 | FLJ45455 protein | 1.1456 | 0.4684 | 0.4089 | 0.0025 |
| Sorcs2 | sortilin-related VPS10 domain containing receptor 2 | 8.4566 | 3.5354 | 0.4181 | 0.0005 |
| rhpn2 | "rhophilin, Rho GTPase binding protein 2; similar to rhophilin, Rho GTPase binding protein 2" | 2.0030 | 0.8628 | 0.4307 | 0.0471 |
| RHPN2P1 | "rhophilin, Rho GTPase binding protein 2; similar to rhophilin, Rho GTPase binding protein 2" | 2.0030 | 0.8628 | 0.4307 | 0.0471 |
| Fat1 | FAT tumor suppressor homolog 1 (Drosophila) | 3.7743 | 1.6331 | 0.4327 | 0.0194 |
| rgma | "RGM domain family, member A" | 17.9974 | 7.9215 | 0.4401 | 0.0089 |
| Plcxd1 | "phosphatidylinositol-specific phospholipase C, X domain containing 1" | 6.9548 | 3.1017 | 0.4460 | 0.0472 |
| ephb1 | EPH receptor B1 | 4.4325 | 1.9781 | 0.4463 | 0.0066 |
| GRIN2D | "glutamate receptor, ionotropic, N-methyl D-aspartate 2D" | 2.1841 | 1.0176 | 0.4659 | 0.0101 |
| Prdm16 | PR domain containing 16 | 2.0348 | 0.9714 | 0.4774 | 0.0423 |
| sdc3 | syndecan 3 | 49.1572 | 23.7673 | 0.4835 | 0.0128 |
| RADIL | Ras association and DIL domains | 1.4385 | 0.7022 | 0.4881 | 0.0440 |
| Ttyh3 | tweety homolog 3 (Drosophila) | 33.0055 | 16.1513 | 0.4894 | 0.0162 |
| LRP5 | low density lipoprotein receptor-related protein 5 | 2.3584 | 1.1611 | 0.4923 | 0.0316 |
| mir126 | microRNA 126 | 1.3909 | 0.6855 | 0.4929 | 0.0276 |
| DCHS1 | dachsous 1 (Drosophila) | 2.5035 | 1.2351 | 0.4934 | 0.0055 |
| ZNF710 | zinc finger protein 710 | 5.9894 | 2.9916 | 0.4995 | 0.0046 |
| SEPN1 | "selenoprotein N, 1" | 14.7191 | 7.3847 | 0.5017 | 0.0078 |
| Ptchd2 | patched domain containing 2 | 2.0549 | 1.0310 | 0.5018 | 0.0032 |
| BAI1 | brain-specific angiogenesis inhibitor 1 | 31.3867 | 15.9550 | 0.5083 | 0.0122 |
| BCL9L | B-cell CLL/lymphoma 9-like | 2.9379 | 1.5002 | 0.5106 | 0.0279 |
| MAPK4 | mitogen-activated protein kinase 4 | 15.2305 | 7.7987 | 0.5120 | 0.0081 |
| LRRC8A | "leucine rich repeat containing 8 family, member A" | 29.6998 | 15.2697 | 0.5141 | 0.0279 |
| Sprn | shadow of prion protein homolog (zebrafish) | 15.7481 | 8.1602 | 0.5182 | 0.0406 |
| KIAA0195 | KIAA0195 | 17.1415 | 8.9091 | 0.5197 | 0.0144 |
| APC2 | adenomatosis polyposis coli 2 | 29.8771 | 15.5930 | 0.5219 | 0.0312 |
| Fam83h | "family with sequence similarity 83, member H" | 2.8450 | 1.4903 | 0.5238 | 0.0252 |
| lrp1 | low density lipoprotein-related protein 1 (alpha-2-macroglobulin receptor) | 23.0753 | 12.1032 | 0.5245 | 0.0062 |
| Slc12a4 | "solute carrier family 12 (potassium/chloride transporters), member 4" | 3.1270 | 1.6413 | 0.5249 | 0.0490 |
| Mir324 | microRNA 324 | 2.4629 | 1.2945 | 0.5256 | 0.0350 |
| DAG1 | dystroglycan 1 (dystrophin-associated glycoprotein 1) | 11.9971 | 6.3648 | 0.5305 | 0.0112 |
| TNRC18B | trinucleotide repeat containing 18B | 9.8015 | 5.2050 | 0.5310 | 0.0484 |
| ECE1 | endothelin converting enzyme 1 | 8.3630 | 4.4485 | 0.5319 | 0.0115 |
| wfs1 | Wolfram syndrome 1 (wolframin) | 18.1636 | 9.7767 | 0.5383 | 0.0185 |
| RN5S17 | "RNA, 5S ribosomal 9; RNA, 5S ribosomal 13; RNA, 5S ribosomal 12; RNA, 5S ribosomal 11; RNA, 5S ribosomal 10; RNA, 5S ribosomal 17; RNA, 5S ribosomal 16; RNA, 5S ribosomal 15; RNA, 5S ribosomal 14; RNA, 5S ribosomal 1; RNA, 5S ribosomal 2; RNA, 5S ribosom | 3.4166 | 1.8427 | 0.5393 | 0.0303 |
| RN5S16 | "RNA, 5S ribosomal 9; RNA, 5S ribosomal 13; RNA, 5S ribosomal 12; RNA, 5S ribosomal 11; RNA, 5S ribosomal 10; RNA, 5S ribosomal 17; RNA, 5S ribosomal 16; RNA, 5S ribosomal 15; RNA, 5S ribosomal 14; RNA, 5S ribosomal 1; RNA, 5S ribosomal 2; RNA, 5S ribosom | 3.4166 | 1.8427 | 0.5393 | 0.0303 |
| RN5S6 | "RNA, 5S ribosomal 9; RNA, 5S ribosomal 13; RNA, 5S ribosomal 12; RNA, 5S ribosomal 11; RNA, 5S ribosomal 10; RNA, 5S ribosomal 17; RNA, 5S ribosomal 16; RNA, 5S ribosomal 15; RNA, 5S ribosomal 14; RNA, 5S ribosomal 1; RNA, 5S ribosomal 2; RNA, 5S ribosom | 3.4166 | 1.8427 | 0.5393 | 0.0303 |
| RN5S7 | "RNA, 5S ribosomal 9; RNA, 5S ribosomal 13; RNA, 5S ribosomal 12; RNA, 5S ribosomal 11; RNA, 5S ribosomal 10; RNA, 5S ribosomal 17; RNA, 5S ribosomal 16; RNA, 5S ribosomal 15; RNA, 5S ribosomal 14; RNA, 5S ribosomal 1; RNA, 5S ribosomal 2; RNA, 5S ribosom | 3.4166 | 1.8427 | 0.5393 | 0.0303 |
| RN5S9 | "RNA, 5S ribosomal 9; RNA, 5S ribosomal 13; RNA, 5S ribosomal 12; RNA, 5S ribosomal 11; RNA, 5S ribosomal 10; RNA, 5S ribosomal 17; RNA, 5S ribosomal 16; RNA, 5S ribosomal 15; RNA, 5S ribosomal 14; RNA, 5S ribosomal 1; RNA, 5S ribosomal 2; RNA, 5S ribosom | 3.4166 | 1.8427 | 0.5393 | 0.0303 |
| RN5S3 | "RNA, 5S ribosomal 9; RNA, 5S ribosomal 13; RNA, 5S ribosomal 12; RNA, 5S ribosomal 11; RNA, 5S ribosomal 10; RNA, 5S ribosomal 17; RNA, 5S ribosomal 16; RNA, 5S ribosomal 15; RNA, 5S ribosomal 14; RNA, 5S ribosomal 1; RNA, 5S ribosomal 2; RNA, 5S ribosom | 3.4166 | 1.8427 | 0.5393 | 0.0303 |
| RN5S4 | "RNA, 5S ribosomal 9; RNA, 5S ribosomal 13; RNA, 5S ribosomal 12; RNA, 5S ribosomal 11; RNA, 5S ribosomal 10; RNA, 5S ribosomal 17; RNA, 5S ribosomal 16; RNA, 5S ribosomal 15; RNA, 5S ribosomal 14; RNA, 5S ribosomal 1; RNA, 5S ribosomal 2; RNA, 5S ribosom | 3.4166 | 1.8427 | 0.5393 | 0.0303 |
| RN5S1 | "RNA, 5S ribosomal 9; RNA, 5S ribosomal 13; RNA, 5S ribosomal 12; RNA, 5S ribosomal 11; RNA, 5S ribosomal 10; RNA, 5S ribosomal 17; RNA, 5S ribosomal 16; RNA, 5S ribosomal 15; RNA, 5S ribosomal 14; RNA, 5S ribosomal 1; RNA, 5S ribosomal 2; RNA, 5S ribosom | 3.4166 | 1.8427 | 0.5393 | 0.0303 |
| RN5S15 | "RNA, 5S ribosomal 9; RNA, 5S ribosomal 13; RNA, 5S ribosomal 12; RNA, 5S ribosomal 11; RNA, 5S ribosomal 10; RNA, 5S ribosomal 17; RNA, 5S ribosomal 16; RNA, 5S ribosomal 15; RNA, 5S ribosomal 14; RNA, 5S ribosomal 1; RNA, 5S ribosomal 2; RNA, 5S ribosom | 3.4166 | 1.8427 | 0.5393 | 0.0303 |
| RN5S8 | "RNA, 5S ribosomal 9; RNA, 5S ribosomal 13; RNA, 5S ribosomal 12; RNA, 5S ribosomal 11; RNA, 5S ribosomal 10; RNA, 5S ribosomal 17; RNA, 5S ribosomal 16; RNA, 5S ribosomal 15; RNA, 5S ribosomal 14; RNA, 5S ribosomal 1; RNA, 5S ribosomal 2; RNA, 5S ribosom | 3.4166 | 1.8427 | 0.5393 | 0.0303 |
| RN5S5 | "RNA, 5S ribosomal 9; RNA, 5S ribosomal 13; RNA, 5S ribosomal 12; RNA, 5S ribosomal 11; RNA, 5S ribosomal 10; RNA, 5S ribosomal 17; RNA, 5S ribosomal 16; RNA, 5S ribosomal 15; RNA, 5S ribosomal 14; RNA, 5S ribosomal 1; RNA, 5S ribosomal 2; RNA, 5S ribosom | 3.4166 | 1.8427 | 0.5393 | 0.0303 |
| RN5S13 | "RNA, 5S ribosomal 9; RNA, 5S ribosomal 13; RNA, 5S ribosomal 12; RNA, 5S ribosomal 11; RNA, 5S ribosomal 10; RNA, 5S ribosomal 17; RNA, 5S ribosomal 16; RNA, 5S ribosomal 15; RNA, 5S ribosomal 14; RNA, 5S ribosomal 1; RNA, 5S ribosomal 2; RNA, 5S ribosom | 3.4166 | 1.8427 | 0.5393 | 0.0303 |
| RN5S12 | "RNA, 5S ribosomal 9; RNA, 5S ribosomal 13; RNA, 5S ribosomal 12; RNA, 5S ribosomal 11; RNA, 5S ribosomal 10; RNA, 5S ribosomal 17; RNA, 5S ribosomal 16; RNA, 5S ribosomal 15; RNA, 5S ribosomal 14; RNA, 5S ribosomal 1; RNA, 5S ribosomal 2; RNA, 5S ribosom | 3.4166 | 1.8427 | 0.5393 | 0.0303 |
| RN5S11 | "RNA, 5S ribosomal 9; RNA, 5S ribosomal 13; RNA, 5S ribosomal 12; RNA, 5S ribosomal 11; RNA, 5S ribosomal 10; RNA, 5S ribosomal 17; RNA, 5S ribosomal 16; RNA, 5S ribosomal 15; RNA, 5S ribosomal 14; RNA, 5S ribosomal 1; RNA, 5S ribosomal 2; RNA, 5S ribosom | 3.4166 | 1.8427 | 0.5393 | 0.0303 |
| RN5S2 | "RNA, 5S ribosomal 9; RNA, 5S ribosomal 13; RNA, 5S ribosomal 12; RNA, 5S ribosomal 11; RNA, 5S ribosomal 10; RNA, 5S ribosomal 17; RNA, 5S ribosomal 16; RNA, 5S ribosomal 15; RNA, 5S ribosomal 14; RNA, 5S ribosomal 1; RNA, 5S ribosomal 2; RNA, 5S ribosom | 3.4166 | 1.8427 | 0.5393 | 0.0303 |
| RN5S10 | "RNA, 5S ribosomal 9; RNA, 5S ribosomal 13; RNA, 5S ribosomal 12; RNA, 5S ribosomal 11; RNA, 5S ribosomal 10; RNA, 5S ribosomal 17; RNA, 5S ribosomal 16; RNA, 5S ribosomal 15; RNA, 5S ribosomal 14; RNA, 5S ribosomal 1; RNA, 5S ribosomal 2; RNA, 5S ribosom | 3.4166 | 1.8427 | 0.5393 | 0.0303 |
| RN5S14 | "RNA, 5S ribosomal 9; RNA, 5S ribosomal 13; RNA, 5S ribosomal 12; RNA, 5S ribosomal 11; RNA, 5S ribosomal 10; RNA, 5S ribosomal 17; RNA, 5S ribosomal 16; RNA, 5S ribosomal 15; RNA, 5S ribosomal 14; RNA, 5S ribosomal 1; RNA, 5S ribosomal 2; RNA, 5S ribosom | 3.4166 | 1.8427 | 0.5393 | 0.0303 |
| caskin2 | CASK interacting protein 2 | 8.3258 | 4.5285 | 0.5439 | 0.0119 |
| Cacng4 | "calcium channel, voltage-dependent, gamma subunit 4" | 5.8744 | 3.1963 | 0.5441 | 0.0322 |
| Gpr75 | G protein-coupled receptor 75 | 3.7713 | 2.0579 | 0.5457 | 0.0106 |
| tp53i11 | tumor protein p53 inducible protein 11 | 5.3294 | 2.9133 | 0.5466 | 0.0157 |
| Gnl3l | guanine nucleotide binding protein-like 3 (nucleolar)-like | 2.0849 | 1.1472 | 0.5502 | 0.0130 |
| CCDC85C | coiled-coil domain containing 85C | 4.3245 | 2.3940 | 0.5536 | 0.0401 |
| Pskh1 | protein serine kinase H1 | 4.1123 | 2.2917 | 0.5573 | 0.0500 |
| mn1 | meningioma (disrupted in balanced translocation) 1 | 2.8469 | 1.5917 | 0.5591 | 0.0035 |
| FAM38A | "family with sequence similarity 38, member A" | 2.6074 | 1.4603 | 0.5601 | 0.0385 |
| TMEM132A | transmembrane protein 132A | 29.8723 | 16.7974 | 0.5623 | 0.0266 |
| WDR86 | WD repeat domain 86 | 1.9269 | 1.0890 | 0.5652 | 0.0454 |
| Tpcn1 | two pore segment channel 1 | 10.8609 | 6.1412 | 0.5654 | 0.0165 |
| GATA2 | GATA binding protein 2 | 1.0725 | 0.6092 | 0.5680 | 0.0469 |
| SLC7A5 | "solute carrier family 7 (cationic amino acid transporter, y+ system), member 5" | 23.4529 | 13.3761 | 0.5703 | 0.0115 |
| TMEM104 | transmembrane protein 104 | 3.6113 | 2.0602 | 0.5705 | 0.0163 |
| ZNF628 | zinc finger protein 628 | 1.7187 | 0.9826 | 0.5717 | 0.0185 |
| Per1 | period homolog 1 (Drosophila) | 10.2926 | 5.8948 | 0.5727 | 0.0210 |
| JMJD8 | jumonji domain containing 8 | 4.6810 | 2.6853 | 0.5737 | 0.0231 |
| plxnb2 | plexin B2 | 9.6950 | 5.5651 | 0.5740 | 0.0252 |
| RAVER1 | "ribonucleoprotein, PTB-binding 1" | 1.9665 | 1.1367 | 0.5781 | 0.0068 |
| SHROOM3 | shroom family member 3 | 1.5664 | 0.9065 | 0.5787 | 0.0170 |
| notch1 | "Notch homolog 1, translocation-associated (Drosophila)" | 5.1118 | 2.9665 | 0.5803 | 0.0188 |
| CHPF | chondroitin polymerizing factor | 21.1567 | 12.2989 | 0.5813 | 0.0043 |
| Tie1 | tyrosine kinase with immunoglobulin-like and EGF-like domains 1 | 2.8841 | 1.6920 | 0.5867 | 0.0011 |
| BOC | Boc homolog (mouse) | 1.0452 | 0.6146 | 0.5881 | 0.0252 |
| C2orf85 | chromosome 2 open reading frame 85 | 5.9640 | 3.5077 | 0.5882 | 0.0245 |
| kiaa0664 | KIAA0664 | 13.6272 | 8.0371 | 0.5898 | 0.0254 |
| sufU | suppressor of fused homolog (Drosophila) | 2.6063 | 1.5378 | 0.5900 | 0.0039 |
| AGRN | agrin | 17.7114 | 10.4845 | 0.5920 | 0.0137 |
| Hcfc1 | host cell factor C1 (VP16-accessory protein) | 10.1541 | 6.0227 | 0.5931 | 0.0411 |
| rgs12 | regulator of G-protein signaling 12 | 7.7069 | 4.5719 | 0.5932 | 0.0220 |
| PLEC | "similar to Plectin 1 (PLTN) (PCN) (Hemidesmosomal protein 1) (HD1); plectin 1, intermediate filament binding protein 500kDa" | 21.2382 | 12.5996 | 0.5933 | 0.0269 |
| LOC652460 | "similar to Plectin 1 (PLTN) (PCN) (Hemidesmosomal protein 1) (HD1); plectin 1, intermediate filament binding protein 500kDa" | 21.2382 | 12.5996 | 0.5933 | 0.0269 |
| KCNN3 | "potassium intermediate/small conductance calcium-activated channel, subfamily N, member 3" | 1.6254 | 0.9663 | 0.5945 | 0.0441 |
| MIDN | midnolin | 10.0463 | 5.9727 | 0.5945 | 0.0241 |
| LRFN3 | leucine rich repeat and fibronectin type III domain containing 3 | 6.7473 | 4.0120 | 0.5946 | 0.0440 |
| Grik5 | "glutamate receptor, ionotropic, kainate 5" | 15.3848 | 9.1520 | 0.5949 | 0.0389 |
| mmp15 | matrix metallopeptidase 15 (membrane-inserted) | 3.4520 | 2.0562 | 0.5956 | 0.0144 |
| Klhl26 | kelch-like 26 (Drosophila) | 10.4873 | 6.2515 | 0.5961 | 0.0066 |
| ST5 | suppression of tumorigenicity 5 | 2.0260 | 1.2086 | 0.5966 | 0.0170 |
| TNKS1BP1 | "tankyrase 1 binding protein 1, 182kDa" | 8.6296 | 5.1509 | 0.5969 | 0.0048 |
| TLE3 | "transducin-like enhancer of split 3 (E(sp1) homolog, Drosophila)" | 2.6320 | 1.5723 | 0.5974 | 0.0201 |
| FKBP9 | "FK506 binding protein 9, 63 kDa" | 10.4672 | 6.2542 | 0.5975 | 0.0136 |
| Foxk1 | forkhead box K1 | 8.0211 | 4.7941 | 0.5977 | 0.0468 |
| SPATA2L | spermatogenesis associated 2-like | 7.6727 | 4.5873 | 0.5979 | 0.0315 |
| GAS2L1 | growth arrest-specific 2 like 1 | 20.0175 | 12.0066 | 0.5998 | 0.0474 |
| Ell | elongation factor RNA polymerase II | 1.9753 | 1.1858 | 0.6003 | 0.0235 |
| WDR81 | WD repeat domain 81 | 2.8008 | 1.6864 | 0.6021 | 0.0059 |
| SLC24A3 | "solute carrier family 24 (sodium/potassium/calcium exchanger), member 3" | 5.0675 | 3.0570 | 0.6033 | 0.0071 |
| tmem198 | transmembrane protein 198 | 8.7396 | 5.2979 | 0.6062 | 0.0239 |
| SLC27A1 | "solute carrier family 27 (fatty acid transporter), member 1" | 7.5732 | 4.5938 | 0.6066 | 0.0417 |
| ATP10A | "ATPase, class V, type 10A" | 1.9660 | 1.1938 | 0.6072 | 0.0126 |
| TMEM164 | transmembrane protein 164 | 3.0995 | 1.8883 | 0.6092 | 0.0431 |
| NPRL3 | chromosome 16 open reading frame 35 | 7.1767 | 4.3850 | 0.6110 | 0.0032 |
| SALL2 | sal-like 2 (Drosophila) | 8.5473 | 5.2303 | 0.6119 | 0.0145 |
| Abcd1 | "ATP-binding cassette, sub-family D (ALD), member 1" | 1.3126 | 0.8038 | 0.6124 | 0.0165 |
| NEURL1B | neuralized homolog 1B (Drosophila) | 4.5540 | 2.7895 | 0.6125 | 0.0393 |
| SLC6A1 | "solute carrier family 6 (neurotransmitter transporter, GABA), member 1" | 81.2451 | 49.8214 | 0.6132 | 0.0053 |
| clpB | ClpB caseinolytic peptidase B homolog (E. coli) | 5.5668 | 3.4167 | 0.6138 | 0.0431 |
| znf687 | zinc finger protein 687 | 4.8208 | 2.9644 | 0.6149 | 0.0020 |
| NLGN3 | neuroligin 3 | 27.4399 | 16.9052 | 0.6161 | 0.0023 |
| Med16 | mediator complex subunit 16 | 14.1849 | 8.7639 | 0.6178 | 0.0227 |
| MDGA1 | MAM domain containing glycosylphosphatidylinositol anchor 1 | 1.8634 | 1.1531 | 0.6188 | 0.0159 |
| AP1B1 | "adaptor-related protein complex 1, beta 1 subunit" | 21.0360 | 13.0316 | 0.6195 | 0.0136 |
| Atg2a | ATG2 autophagy related 2 homolog A (S. cerevisiae) | 6.0947 | 3.7759 | 0.6195 | 0.0155 |
| LRP3 | low density lipoprotein receptor-related protein 3 | 39.1814 | 24.2818 | 0.6197 | 0.0379 |
| Col5a1 | "collagen, type V, alpha 1" | 1.0236 | 0.6346 | 0.6200 | 0.0257 |
| sdk2 | sidekick homolog 2 (chicken) | 1.4975 | 0.9299 | 0.6210 | 0.0436 |
| sema3f | "sema domain, immunoglobulin domain (Ig), short basic domain, secreted, (semaphorin) 3F" | 1.1414 | 0.7104 | 0.6224 | 0.0407 |
| Zc3h4 | zinc finger CCCH-type containing 4 | 6.2162 | 3.8701 | 0.6226 | 0.0090 |
| RXRA | "retinoid X receptor, alpha" | 7.1832 | 4.4792 | 0.6236 | 0.0377 |
| CDH4 | "cadherin 4, type 1, R-cadherin (retinal)" | 6.5634 | 4.0928 | 0.6236 | 0.0274 |
| CSDC2 | "cold shock domain containing C2, RNA binding" | 11.6521 | 7.2747 | 0.6243 | 0.0246 |
| bcr | breakpoint cluster region | 12.6294 | 7.9011 | 0.6256 | 0.0198 |
| ATN1 | atrophin 1 | 82.8166 | 51.8512 | 0.6261 | 0.0465 |
| Sel1l3 | KIAA0746 protein | 3.5851 | 2.2502 | 0.6277 | 0.0339 |
| vps37b | vacuolar protein sorting 37 homolog B (S. cerevisiae) | 4.2051 | 2.6423 | 0.6284 | 0.0171 |
| WIZ | widely interspaced zinc finger motifs | 8.0758 | 5.0746 | 0.6284 | 0.0262 |
| BEGAIN | brain-enriched guanylate kinase-associated homolog (rat) | 8.7029 | 5.4768 | 0.6293 | 0.0233 |
| CCDC9 | coiled-coil domain containing 9 | 6.6880 | 4.2100 | 0.6295 | 0.0405 |
| WWC1 | WW and C2 domain containing 1 | 9.0736 | 5.7350 | 0.6321 | 0.0432 |
| ZNF70 | zinc finger protein 70 | 1.1551 | 0.7314 | 0.6332 | 0.0473 |
| Fam5b | "family with sequence similarity 5, member B" | 19.8083 | 12.5476 | 0.6334 | 0.0021 |
| LRRC56 | leucine rich repeat containing 56 | 1.7978 | 1.1394 | 0.6338 | 0.0309 |
| sox1 | SRY (sex determining region Y)-box 1 | 1.4607 | 0.9268 | 0.6345 | 0.0191 |
| speG | SPEG complex locus | 5.3228 | 3.3804 | 0.6351 | 0.0324 |
| GRAMD4 | "TSC22 domain family, member 3; GRAM domain containing 4" | 11.9012 | 7.5684 | 0.6359 | 0.0164 |
| TSC22D3 | "TSC22 domain family, member 3; GRAM domain containing 4" | 11.9012 | 7.5684 | 0.6359 | 0.0164 |
| Znf516 | zinc finger protein 516 | 1.7427 | 1.1094 | 0.6366 | 0.0429 |
| slc1a4 | "solute carrier family 1 (glutamate/neutral amino acid transporter), member 4" | 14.4544 | 9.2020 | 0.6366 | 0.0276 |
| MAVS | mitochondrial antiviral signaling protein | 4.2655 | 2.7159 | 0.6367 | 0.0338 |
| Nlgn2 | neuroligin 2 | 39.0978 | 24.9020 | 0.6369 | 0.0376 |
| PCNXL3 | pecanex-like 3 (Drosophila) | 5.4681 | 3.4946 | 0.6391 | 0.0039 |
| ZNF282 | zinc finger protein 282 | 6.2300 | 3.9819 | 0.6392 | 0.0106 |
| CREB3L1 | cAMP responsive element binding protein 3-like 1 | 2.7431 | 1.7539 | 0.6394 | 0.0171 |
| Srcap | Snf2-related CREBBP activator protein | 9.3053 | 5.9587 | 0.6404 | 0.0343 |
| CREBBP | CREB binding protein | 6.6923 | 4.2879 | 0.6407 | 0.0372 |
| ZBTB45 | zinc finger and BTB domain containing 45 | 7.2703 | 4.6644 | 0.6416 | 0.0484 |
| Nacc1 | "nucleus accumbens associated 1, BEN and BTB (POZ) domain containing" | 21.5839 | 13.8551 | 0.6419 | 0.0275 |
| CABIN1 | calcineurin binding protein 1 | 10.7648 | 6.9103 | 0.6419 | 0.0395 |
| C15orf59 | chromosome 15 open reading frame 59 | 41.8528 | 26.8811 | 0.6423 | 0.0418 |
| KIAA0649 | KIAA0649 | 11.3902 | 7.3183 | 0.6425 | 0.0180 |
| Hyal1 | hyaluronoglucosaminidase 1 | 1.1700 | 0.7527 | 0.6433 | 0.0113 |
| ptch1 | patched homolog 1 (Drosophila) | 2.0593 | 1.3250 | 0.6434 | 0.0313 |
| EHD1 | EH-domain containing 1 | 5.9121 | 3.8057 | 0.6437 | 0.0138 |
| SH3GL1 | SH3-domain GRB2-like 1 | 16.4607 | 10.5986 | 0.6439 | 0.0266 |
| Meis3 | Meis homeobox 3; Meis homeobox 3 pseudogene 2 | 20.1277 | 12.9769 | 0.6447 | 0.0396 |
| MEIS3P2 | Meis homeobox 3; Meis homeobox 3 pseudogene 2 | 20.1277 | 12.9769 | 0.6447 | 0.0396 |
| Pc | pyruvate carboxylase | 17.7166 | 11.4315 | 0.6452 | 0.0361 |
| MYH9 | "myosin, heavy chain 9, non-muscle" | 23.9754 | 15.4855 | 0.6459 | 0.0217 |
| SCAP | SREBF chaperone | 15.2742 | 9.8832 | 0.6471 | 0.0053 |
| b4galt2 | "UDP-Gal:betaGlcNAc beta 1,4- galactosyltransferase, polypeptide 2" | 23.5979 | 15.2731 | 0.6472 | 0.0075 |
| PRKD2 | protein kinase D2 | 2.2135 | 1.4346 | 0.6481 | 0.0345 |
| CRB2 | crumbs homolog 2 (Drosophila) | 4.1039 | 2.6610 | 0.6484 | 0.0464 |
| mfi2 | antigen p97 (melanoma associated) identified by monoclonal antibodies 133.2 and 96.5 | 1.5728 | 1.0212 | 0.6493 | 0.0380 |
| RGS9 | regulator of G-protein signaling 9 | 1.1230 | 0.7294 | 0.6495 | 0.0494 |
| ARHGEF10L | Rho guanine nucleotide exchange factor (GEF) 10-like | 5.7591 | 3.7423 | 0.6498 | 0.0265 |
| SETD1A | SET domain containing 1A | 5.0950 | 3.3194 | 0.6515 | 0.0358 |
| Crat | carnitine acetyltransferase | 15.7618 | 10.2966 | 0.6533 | 0.0207 |
| Ptbp1 | polypyrimidine tract binding protein 1 | 5.9044 | 3.8591 | 0.6536 | 0.0457 |
| Alg12 | "asparagine-linked glycosylation 12, alpha-1,6-mannosyltransferase homolog (S. cerevisiae)" | 4.9756 | 3.2574 | 0.6547 | 0.0082 |
| Fjx1 | four jointed box 1 (Drosophila) | 7.8300 | 5.1273 | 0.6548 | 0.0349 |
| NLRX1 | NLR family member X1 | 1.9300 | 1.2646 | 0.6552 | 0.0203 |
| MINK1 | misshapen-like kinase 1 (zebrafish) | 33.8796 | 22.2199 | 0.6558 | 0.0436 |
| Mier2 | "mesoderm induction early response 1, family member 2" | 5.2335 | 3.4334 | 0.6560 | 0.0288 |
| ZNF777 | zinc finger protein 777 | 6.8514 | 4.4994 | 0.6567 | 0.0382 |
| Plxnd1 | plexin D1 | 8.3438 | 5.4822 | 0.6570 | 0.0080 |
| GALNT2 | UDP-N-acetyl-alpha-D-galactosamine:polypeptide N-acetylgalactosaminyltransferase 2 (GalNAc-T2) | 6.5009 | 4.2751 | 0.6576 | 0.0074 |
| Ldlr | low density lipoprotein receptor | 3.0424 | 2.0028 | 0.6583 | 0.0274 |
| Mapk7 | mitogen-activated protein kinase 7 | 2.1983 | 1.4484 | 0.6589 | 0.0205 |
| ZNF362 | zinc finger protein 362 | 6.1694 | 4.0677 | 0.6593 | 0.0396 |
| Ehd4 | EH-domain containing 4 | 1.4515 | 0.9577 | 0.6598 | 0.0387 |
| actn4 | "actinin, alpha 4" | 40.2279 | 26.5645 | 0.6604 | 0.0101 |
| cyp46a1 | "cytochrome P450, family 46, subfamily A, polypeptide 1" | 13.6795 | 9.0371 | 0.6606 | 0.0270 |
| ABR | active BCR-related gene | 56.4049 | 37.2734 | 0.6608 | 0.0498 |
| ARHGAP33 | sorting nexin 26 | 18.4753 | 12.2420 | 0.6626 | 0.0465 |
| ephb2 | EPH receptor B2 | 2.7745 | 1.8390 | 0.6628 | 0.0293 |
| ncln | nicalin homolog (zebrafish) | 11.8861 | 7.8812 | 0.6631 | 0.0417 |
| SNX8 | sorting nexin 8 | 6.4651 | 4.2904 | 0.6636 | 0.0434 |
| PFKL | "phosphofructokinase, liver" | 24.3672 | 16.1929 | 0.6645 | 0.0055 |
| zbtb46 | zinc finger and BTB domain containing 46 | 2.9935 | 1.9909 | 0.6651 | 0.0405 |
| GYS1 | glycogen synthase 1 (muscle) | 7.2942 | 4.8544 | 0.6655 | 0.0088 |
| LPPR2 | lipid phosphate phosphatase-related protein type 2 | 36.7002 | 24.4556 | 0.6664 | 0.0368 |
| GABBR1 | "gamma-aminobutyric acid (GABA) B receptor, 1" | 76.1743 | 50.8778 | 0.6679 | 0.0143 |
| TMEM8B | transmembrane protein 8B | 10.0936 | 6.7505 | 0.6688 | 0.0223 |
| plk3 | polo-like kinase 3 (Drosophila) | 1.8518 | 1.2386 | 0.6688 | 0.0168 |
| calhm2 | calcium homeostasis modulator 2 | 2.9240 | 1.9598 | 0.6703 | 0.0313 |
| akt1 | v-akt murine thymoma viral oncogene homolog 1 | 7.0057 | 4.6971 | 0.6705 | 0.0273 |
| STRN4 | "striatin, calmodulin binding protein 4" | 12.4723 | 8.3639 | 0.6706 | 0.0377 |
| TRPM4 | "transient receptor potential cation channel, subfamily M, member 4" | 2.0168 | 1.3536 | 0.6712 | 0.0476 |
| TRAF7 | TNF receptor-associated factor 7 | 8.8980 | 5.9746 | 0.6714 | 0.0451 |
| ANO8 | anoctamin 8 | 10.8354 | 7.2820 | 0.6721 | 0.0158 |
| Arvcf | armadillo repeat gene deletes in velocardiofacial syndrome | 6.5503 | 4.4027 | 0.6721 | 0.0252 |
| Rasip1 | Ras interacting protein 1 | 2.4521 | 1.6483 | 0.6722 | 0.0466 |
| STAG3L1 | stromal antigen 3-like 2; stromal antigen 3-like 1 | 2.0466 | 1.3770 | 0.6728 | 0.0365 |
| STAG3L2 | stromal antigen 3-like 2; stromal antigen 3-like 1 | 2.0466 | 1.3770 | 0.6728 | 0.0365 |
| Tab1 | mitogen-activated protein kinase kinase kinase 7 interacting protein 1 | 9.9706 | 6.7174 | 0.6737 | 0.0058 |
| Prkca | "protein kinase C, alpha" | 18.9411 | 12.7663 | 0.6740 | 0.0137 |
| PTK7 | PTK7 protein tyrosine kinase 7 | 1.1310 | 0.7638 | 0.6753 | 0.0124 |
| RNF40 | ring finger protein 40 | 12.8496 | 8.6799 | 0.6755 | 0.0172 |
| ltbp3 | latent transforming growth factor beta binding protein 3 | 13.6522 | 9.2256 | 0.6758 | 0.0374 |
| pygb | "phosphorylase, glycogen; brain" | 52.3591 | 35.3825 | 0.6758 | 0.0191 |
| SCARB1 | "scavenger receptor class B, member 1" | 7.0364 | 4.7550 | 0.6758 | 0.0113 |
| Mta1 | metastasis associated 1 | 7.6375 | 5.1615 | 0.6758 | 0.0257 |
| Slc43a2 | "solute carrier family 43, member 2" | 7.2461 | 4.8996 | 0.6762 | 0.0489 |
| xrcc3 | X-ray repair complementing defective repair in Chinese hamster cells 3 | 1.4821 | 1.0029 | 0.6767 | 0.0469 |
| ZC3H3 | zinc finger CCCH-type containing 3 | 5.0437 | 3.4140 | 0.6769 | 0.0184 |
| EPHB3 | EPH receptor B3 | 3.0338 | 2.0538 | 0.6770 | 0.0092 |
| PAK4 | p21 protein (Cdc42/Rac)-activated kinase 4 | 3.6253 | 2.4547 | 0.6771 | 0.0161 |
| GREB1 | GREB1 protein | 2.7210 | 1.8452 | 0.6781 | 0.0136 |
| Zmiz1 | "zinc finger, MIZ-type containing 1" | 13.3092 | 9.0503 | 0.6800 | 0.0490 |
| Ptpn23 | "protein tyrosine phosphatase, non-receptor type 23" | 13.2609 | 9.0245 | 0.6805 | 0.0299 |
| Ogdh | oxoglutarate (alpha-ketoglutarate) dehydrogenase (lipoamide) | 31.7670 | 21.6295 | 0.6809 | 0.0158 |
| Stk32c | serine/threonine kinase 32C | 18.9868 | 12.9388 | 0.6815 | 0.0340 |
| Tcf7l2 | "transcription factor 7-like 2 (T-cell specific, HMG-box)" | 4.7032 | 3.2059 | 0.6816 | 0.0256 |
| FOXRED2 | FAD-dependent oxidoreductase domain containing 2 | 10.6099 | 7.2334 | 0.6818 | 0.0186 |
| TMEM184B | transmembrane protein 184B | 18.9301 | 12.9089 | 0.6819 | 0.0043 |
| SIDT2 | "SID1 transmembrane family, member 2" | 6.4622 | 4.4076 | 0.6821 | 0.0045 |
| MOBKL2A | "MOB1, Mps One Binder kinase activator-like 2A (yeast)" | 4.6356 | 3.1620 | 0.6821 | 0.0326 |
| LETM1 | leucine zipper-EF-hand containing transmembrane protein 1 | 7.4379 | 5.0776 | 0.6827 | 0.0073 |
| VPS18 | vacuolar protein sorting 18 homolog (S. cerevisiae) | 9.6564 | 6.5968 | 0.6832 | 0.0472 |
| OSBPL5 | oxysterol binding protein-like 5 | 4.0906 | 2.7957 | 0.6834 | 0.0363 |
| ercc2 | "excision repair cross-complementing rodent repair deficiency, complementation group 2" | 4.6399 | 3.1718 | 0.6836 | 0.0213 |
| nfix | nuclear factor I/X (CCAAT-binding transcription factor) | 55.7459 | 38.1076 | 0.6836 | 0.0427 |
| por | P450 (cytochrome) oxidoreductase | 7.7847 | 5.3227 | 0.6837 | 0.0121 |
| UPF1 | UPF1 regulator of nonsense transcripts homolog (yeast) | 10.8764 | 7.4384 | 0.6839 | 0.0254 |
| dtx1 | deltex homolog 1 (Drosophila) | 11.6639 | 7.9898 | 0.6850 | 0.0241 |
| PXDN | peroxidasin homolog (Drosophila) | 1.3189 | 0.9050 | 0.6862 | 0.0271 |
| Xylt2 | xylosyltransferase II | 4.0958 | 2.8122 | 0.6866 | 0.0194 |
| Pofut1 | protein O-fucosyltransferase 1 | 2.7134 | 1.8643 | 0.6871 | 0.0194 |
| GTPBP1 | GTP binding protein 1 | 5.5833 | 3.8378 | 0.6874 | 0.0078 |
| MYH14 | "myosin, heavy chain 14" | 7.8348 | 5.3856 | 0.6874 | 0.0307 |
| tcf3 | transcription factor 3 (E2A immunoglobulin enhancer binding factors E12/E47) | 3.7732 | 2.5937 | 0.6874 | 0.0395 |
| SEMA4G | "sema domain, immunoglobulin domain (Ig), transmembrane domain (TM) and short cytoplasmic domain, (semaphorin) 4G" | 1.9902 | 1.3683 | 0.6875 | 0.0194 |
| EHMT1 | euchromatic histone-lysine N-methyltransferase 1 | 2.5419 | 1.7477 | 0.6876 | 0.0165 |
| C17orf103 | chromosome 17 open reading frame 103 | 2.5344 | 1.7433 | 0.6879 | 0.0085 |
| PNPLA7 | patatin-like phospholipase domain containing 7 | 4.1373 | 2.8460 | 0.6879 | 0.0397 |
| NXPH3 | neurexophilin 3 | 7.1953 | 4.9518 | 0.6882 | 0.0406 |
| khsrp | KH-type splicing regulatory protein | 28.9518 | 19.9442 | 0.6889 | 0.0367 |
| PDZRN3 | PDZ domain containing ring finger 3 | 5.8300 | 4.0172 | 0.6891 | 0.0087 |
| Trim65 | tripartite motif-containing 65 | 5.1876 | 3.5754 | 0.6892 | 0.0158 |
| tmem8a | transmembrane protein 8A | 6.5148 | 4.4912 | 0.6894 | 0.0470 |
| ARID1A | AT rich interactive domain 1A (SWI-like) | 8.6099 | 5.9365 | 0.6895 | 0.0255 |
| c19orf6 | chromosome 19 open reading frame 6 | 34.2619 | 23.6315 | 0.6897 | 0.0487 |
| LOC152845 | pleiomorphic adenoma gene-like 2; similar to pleiomorphic adenoma gene-like 2 | 1.9426 | 1.3414 | 0.6905 | 0.0016 |
| PLAGL2 | pleiomorphic adenoma gene-like 2; similar to pleiomorphic adenoma gene-like 2 | 1.9426 | 1.3414 | 0.6905 | 0.0016 |
| BRPF1 | "bromodomain and PHD finger containing, 1" | 3.3802 | 2.3369 | 0.6914 | 0.0105 |
| MEX3B | mex-3 homolog B (C. elegans) | 1.6832 | 1.1645 | 0.6919 | 0.0099 |
| fgd1 | "FYVE, RhoGEF and PH domain containing 1" | 5.5612 | 3.8492 | 0.6922 | 0.0465 |
| YBX2 | Y box binding protein 2 | 1.0651 | 0.7374 | 0.6923 | 0.0442 |
| Dot1l | "DOT1-like, histone H3 methyltransferase (S. cerevisiae)" | 2.1378 | 1.4811 | 0.6928 | 0.0493 |
| TBC1D14 | "TBC1 domain family, member 14" | 5.8234 | 4.0369 | 0.6932 | 0.0477 |
| SLC41A1 | "solute carrier family 41, member 1" | 8.9258 | 6.1884 | 0.6933 | 0.0125 |
| RFX1 | "regulatory factor X, 1 (influences HLA class II expression)" | 2.4034 | 1.6664 | 0.6934 | 0.0331 |
| BCL9 | B-cell CLL/lymphoma 9 | 5.0097 | 3.4748 | 0.6936 | 0.0173 |
| BAHD1 | bromo adjacent homology domain containing 1 | 5.8269 | 4.0521 | 0.6954 | 0.0231 |
| CCDC120 | coiled-coil domain containing 120 | 1.7626 | 1.2261 | 0.6956 | 0.0458 |
| TOM1L2 | target of myb1-like 2 (chicken) | 36.9437 | 25.7301 | 0.6965 | 0.0196 |
| GMEB2 | glucocorticoid modulatory element binding protein 2 | 4.0831 | 2.8440 | 0.6965 | 0.0271 |
| KDM4B | lysine (K)-specific demethylase 4B | 4.6069 | 3.2093 | 0.6966 | 0.0094 |
| FBXW8 | F-box and WD repeat domain containing 8 | 1.6581 | 1.1557 | 0.6970 | 0.0093 |
| TECPR2 | tectonin beta-propeller repeat containing 2 | 10.6621 | 7.4375 | 0.6976 | 0.0114 |
| PXN | paxillin | 2.8819 | 2.0106 | 0.6977 | 0.0314 |
| DNAJB5 | "DnaJ (Hsp40) homolog, subfamily B, member 5" | 5.5846 | 3.8991 | 0.6982 | 0.0055 |
| C17orf70 | chromosome 17 open reading frame 70 | 4.7205 | 3.2985 | 0.6988 | 0.0235 |
| IFT140 | intraflagellar transport 140 homolog (Chlamydomonas) | 3.8782 | 2.7114 | 0.6992 | 0.0318 |
| Ctdp1 | "CTD (carboxy-terminal domain, RNA polymerase II, polypeptide A) phosphatase, subunit 1" | 3.1220 | 2.1881 | 0.7009 | 0.0279 |
| MDC1 | mediator of DNA-damage checkpoint 1 | 3.6798 | 2.5804 | 0.7012 | 0.0104 |
| PAQR7 | progestin and adipoQ receptor family member VII | 3.4291 | 2.4078 | 0.7022 | 0.0109 |
| CYFIP1 | cytoplasmic FMR1 interacting protein 1 | 4.8136 | 3.3813 | 0.7025 | 0.0081 |
| Axin2 | axin 2 | 3.4034 | 2.3909 | 0.7025 | 0.0336 |
| Eif4g1 | "eukaryotic translation initiation factor 4 gamma, 1" | 24.2225 | 17.0172 | 0.7025 | 0.0135 |
| pprc1 | "peroxisome proliferator-activated receptor gamma, coactivator-related 1" | 3.9640 | 2.7855 | 0.7027 | 0.0445 |
| GTF3C1 | "general transcription factor IIIC, polypeptide 1, alpha 220kDa" | 8.3648 | 5.8799 | 0.7029 | 0.0446 |
| PHF12 | PHD finger protein 12 | 3.1612 | 2.2222 | 0.7030 | 0.0007 |
| SMAD3 | SMAD family member 3 | 6.7728 | 4.7634 | 0.7033 | 0.0278 |
| Acox3 | "acyl-Coenzyme A oxidase 3, pristanoyl" | 3.1833 | 2.2394 | 0.7035 | 0.0006 |
| TMEM201 | transmembrane protein 201 | 3.4430 | 2.4221 | 0.7035 | 0.0225 |
| Tbcd | tubulin folding cofactor D | 7.7094 | 5.4278 | 0.7041 | 0.0360 |
| Sirpa | signal-regulatory protein alpha | 109.7370 | 77.2938 | 0.7044 | 0.0383 |
| FBXW5 | F-box and WD repeat domain containing 5 | 34.5686 | 24.3556 | 0.7046 | 0.0415 |
| TSPAN14 | tetraspanin 14 | 7.5003 | 5.2896 | 0.7053 | 0.0099 |
| ZNF408 | zinc finger protein 408 | 2.4910 | 1.7585 | 0.7060 | 0.0172 |
| RASSF7 | Ras association (RalGDS/AF-6) domain family (N-terminal) member 7 | 3.6305 | 2.5644 | 0.7064 | 0.0386 |
| RBMS2P1 | "RNA binding motif, single stranded interacting protein 2; RNA binding motif, single stranded interacting protein 2 pseudogene" | 1.9836 | 1.4014 | 0.7065 | 0.0491 |
| rbms2 | "RNA binding motif, single stranded interacting protein 2; RNA binding motif, single stranded interacting protein 2 pseudogene" | 1.9836 | 1.4014 | 0.7065 | 0.0491 |
| GTPBP5 | GTP binding protein 5 (putative) | 4.8850 | 3.4561 | 0.7075 | 0.0292 |
| kiaa0556 | KIAA0556 | 2.7289 | 1.9319 | 0.7080 | 0.0047 |
| MVD | mevalonate (diphospho) decarboxylase | 13.4266 | 9.5141 | 0.7086 | 0.0304 |
| Kifc3 | kinesin family member C3 | 6.7475 | 4.7816 | 0.7086 | 0.0248 |
| ZNF646 | zinc finger protein 646 | 2.3409 | 1.6595 | 0.7089 | 0.0310 |
| mtmr3 | myotubularin related protein 3 | 6.0156 | 4.2666 | 0.7093 | 0.0076 |
| sp2 | Sp2 transcription factor | 7.4173 | 5.2627 | 0.7095 | 0.0150 |
| p4hb | "prolyl 4-hydroxylase, beta polypeptide" | 17.4134 | 12.3594 | 0.7098 | 0.0040 |
| PDK2 | "pyruvate dehydrogenase kinase, isozyme 2" | 10.5770 | 7.5126 | 0.7103 | 0.0323 |
| fes | feline sarcoma oncogene | 2.0138 | 1.4316 | 0.7109 | 0.0381 |
| EP300 | E1A binding protein p300 | 7.7356 | 5.4992 | 0.7109 | 0.0257 |
| c19orf22 | chromosome 19 open reading frame 22 | 14.0561 | 9.9981 | 0.7113 | 0.0300 |
| NAV2 | neuron navigator 2 | 2.9725 | 2.1153 | 0.7116 | 0.0064 |
| ZNF764 | zinc finger protein 764 | 2.0308 | 1.4460 | 0.7120 | 0.0068 |
| traF2 | TNF receptor-associated factor 2 | 3.5728 | 2.5448 | 0.7123 | 0.0308 |
| MGAT4B | "mannosyl (alpha-1,3-)-glycoprotein beta-1,4-N-acetylglucosaminyltransferase, isozyme B" | 9.9313 | 7.0759 | 0.7125 | 0.0248 |
| Chmp1a | chromatin modifying protein 1A | 24.0315 | 17.1264 | 0.7127 | 0.0305 |
| FBRS | fibrosin | 8.3944 | 5.9938 | 0.7140 | 0.0300 |
| spen | "spen homolog, transcriptional regulator (Drosophila)" | 7.6682 | 5.4815 | 0.7148 | 0.0455 |
| ADRB1 | "adrenergic, beta-1-, receptor" | 6.1744 | 4.4151 | 0.7151 | 0.0278 |
| C14orf43 | chromosome 14 open reading frame 43 | 2.7837 | 1.9913 | 0.7153 | 0.0428 |
| trim8 | tripartite motif-containing 8 | 39.0551 | 27.9679 | 0.7161 | 0.0483 |
| GPR172A | G protein-coupled receptor 172A | 4.0416 | 2.8943 | 0.7161 | 0.0365 |
| PQLC2 | PQ loop repeat containing 2 | 5.0767 | 3.6364 | 0.7163 | 0.0085 |
| ZBTB48 | zinc finger and BTB domain containing 48 | 3.4105 | 2.4440 | 0.7166 | 0.0029 |
| Ndor1 | NADPH dependent diflavin oxidoreductase 1 | 6.3182 | 4.5281 | 0.7167 | 0.0482 |
| FAM57A | "family with sequence similarity 57, member A" | 2.8144 | 2.0183 | 0.7171 | 0.0450 |
| erF | Ets2 repressor factor | 11.4409 | 8.2080 | 0.7174 | 0.0411 |
| Pold1 | "polymerase (DNA directed), delta 1, catalytic subunit 125kDa" | 1.1904 | 0.8541 | 0.7175 | 0.0450 |
| NOL9 | nucleolar protein 9 | 3.6319 | 2.6083 | 0.7182 | 0.0189 |
| CSNK1D | "casein kinase 1, delta" | 12.3592 | 8.8811 | 0.7186 | 0.0422 |
| LMF1 | lipase maturation factor 1 | 3.1453 | 2.2615 | 0.7190 | 0.0166 |
| PLCG1 | "phospholipase C, gamma 1" | 15.7131 | 11.2990 | 0.7191 | 0.0113 |
| rnf166 | ring finger protein 166 | 3.2208 | 2.3166 | 0.7193 | 0.0426 |
| ARMC5 | armadillo repeat containing 5 | 3.4819 | 2.5067 | 0.7199 | 0.0418 |
| Fbxo46 | F-box protein 46 | 2.7126 | 1.9536 | 0.7202 | 0.0364 |
| ERC1 | ELKS/RAB6-interacting/CAST family member 1 | 3.6865 | 2.6571 | 0.7208 | 0.0488 |
| C20orf117 | chromosome 20 open reading frame 117 | 5.8702 | 4.2335 | 0.7212 | 0.0387 |
| MED12 | mediator complex subunit 12 | 4.1357 | 2.9871 | 0.7223 | 0.0058 |
| Cant1 | calcium activated nucleotidase 1 | 6.3520 | 4.5900 | 0.7226 | 0.0445 |
| Dcaf15 | chromosome 19 open reading frame 72 | 5.0849 | 3.6746 | 0.7226 | 0.0104 |
| wdr18 | WD repeat domain 18 | 10.5861 | 7.6517 | 0.7228 | 0.0187 |
| Hdac7 | histone deacetylase 7 | 3.4576 | 2.5007 | 0.7232 | 0.0154 |
| Sgta | "small glutamine-rich tetratricopeptide repeat (TPR)-containing, alpha" | 25.6054 | 18.5248 | 0.7235 | 0.0154 |
| Sf3a1 | "splicing factor 3a, subunit 1, 120kDa" | 15.9981 | 11.5789 | 0.7238 | 0.0332 |
| gcn1l1 | GCN1 general control of amino-acid synthesis 1-like 1 (yeast) | 6.1776 | 4.4746 | 0.7243 | 0.0270 |
| CLPTM1 | cleft lip and palate associated transmembrane protein 1 | 33.5949 | 24.3507 | 0.7248 | 0.0243 |
| rpap1 | RNA polymerase II associated protein 1 | 4.0509 | 2.9383 | 0.7254 | 0.0160 |
| Phf2 | PHD finger protein 2 | 9.1130 | 6.6197 | 0.7264 | 0.0281 |
| CD276 | CD276 molecule | 3.0379 | 2.2084 | 0.7269 | 0.0322 |
| Sema4a | "sema domain, immunoglobulin domain (Ig), transmembrane domain (TM) and short cytoplasmic domain, (semaphorin) 4A" | 5.6273 | 4.0908 | 0.7270 | 0.0432 |
| cad | "carbamoyl-phosphate synthetase 2, aspartate transcarbamylase, and dihydroorotase" | 3.1236 | 2.2738 | 0.7279 | 0.0271 |
| hectd3 | HECT domain containing 3 | 8.2178 | 5.9934 | 0.7293 | 0.0291 |
| MAML1 | mastermind-like 1 (Drosophila) | 2.7665 | 2.0178 | 0.7294 | 0.0068 |
| NCKAP5L | KIAA1602 | 4.2353 | 3.0959 | 0.7310 | 0.0345 |
| GPR137 | G protein-coupled receptor 137 | 13.7143 | 10.0259 | 0.7311 | 0.0413 |
| Ints5 | integrator complex subunit 5 | 4.5861 | 3.3622 | 0.7331 | 0.0311 |
| Fam160a2 | "family with sequence similarity 160, member A2" | 6.3684 | 4.6731 | 0.7338 | 0.0168 |
| CPNE6 | copine VI (neuronal) | 12.5595 | 9.2252 | 0.7345 | 0.0254 |
| gcc1 | GRIP and coiled-coil domain containing 1 | 2.4965 | 1.8350 | 0.7350 | 0.0002 |
| POFUT2 | protein O-fucosyltransferase 2 | 3.3400 | 2.4551 | 0.7351 | 0.0132 |
| Gramd1a | GRAM domain containing 1A | 7.6353 | 5.6141 | 0.7353 | 0.0452 |
| UNC45A | unc-45 homolog A (C. elegans) | 5.2850 | 3.8888 | 0.7358 | 0.0119 |
| WHSC2 | Wolf-Hirschhorn syndrome candidate 2 | 5.2137 | 3.8371 | 0.7360 | 0.0365 |
| Adora1 | adenosine A1 receptor | 23.5785 | 17.3529 | 0.7360 | 0.0401 |
| DVL3 | "dishevelled, dsh homolog 3 (Drosophila)" | 14.3087 | 10.5381 | 0.7365 | 0.0202 |
| SLC35C1 | "solute carrier family 35, member C1" | 3.2370 | 2.3841 | 0.7365 | 0.0490 |
| GNAO1 | "guanine nucleotide binding protein (G protein), alpha activating activity polypeptide O" | 52.0036 | 38.3213 | 0.7369 | 0.0153 |
| lpcat1 | lysophosphatidylcholine acyltransferase 1 | 8.2387 | 6.0733 | 0.7372 | 0.0219 |
| rab36 | "RAB36, member RAS oncogene family" | 4.6704 | 3.4434 | 0.7373 | 0.0489 |
| Inpp5d | "inositol polyphosphate-5-phosphatase, 145kDa" | 1.3028 | 0.9608 | 0.7375 | 0.0348 |
| ACO2 | "aconitase 2, mitochondrial" | 50.2094 | 37.0361 | 0.7376 | 0.0203 |
| ZNF319 | zinc finger protein 319 | 2.8998 | 2.1412 | 0.7384 | 0.0224 |
| SLITRK2 | "similar to CXorf2 protein; SLIT and NTRK-like family, member 2" | 7.0332 | 5.1945 | 0.7386 | 0.0272 |
| LOC100129095 | "similar to CXorf2 protein; SLIT and NTRK-like family, member 2" | 7.0332 | 5.1945 | 0.7386 | 0.0272 |
| AXIN1 | axin 1 | 4.0061 | 2.9601 | 0.7389 | 0.0351 |
| SLC25A23 | "solute carrier family 25 (mitochondrial carrier; phosphate carrier), member 23" | 89.9340 | 66.4531 | 0.7389 | 0.0270 |
| DOLK | dolichol kinase | 4.7724 | 3.5273 | 0.7391 | 0.0227 |
| C2orf18 | chromosome 2 open reading frame 18 | 7.4177 | 5.4826 | 0.7391 | 0.0022 |
| AGPAT3 | 1-acylglycerol-3-phosphate O-acyltransferase 3 | 14.6361 | 10.8232 | 0.7395 | 0.0417 |
| SNRNP200 | "similar to U5 snRNP-specific protein, 200 kDa; small nuclear ribonucleoprotein 200kDa (U5)" | 11.3146 | 8.3692 | 0.7397 | 0.0029 |
| LOC652147 | "similar to U5 snRNP-specific protein, 200 kDa; small nuclear ribonucleoprotein 200kDa (U5)" | 11.3146 | 8.3692 | 0.7397 | 0.0029 |
| heatr2 | HEAT repeat containing 2 | 1.6030 | 1.1858 | 0.7397 | 0.0151 |
| lmf2 | lipase maturation factor 2 | 13.5955 | 10.0586 | 0.7398 | 0.0388 |
| Dclk2 | doublecortin-like kinase 2 | 23.0555 | 17.0618 | 0.7400 | 0.0054 |
| LOC100133760 | "similar to Jumonji, AT rich interactive domain 1B (RBP2-like); lysine (K)-specific demethylase 5B" | 4.1368 | 3.0635 | 0.7405 | 0.0083 |
| KDM5B | "similar to Jumonji, AT rich interactive domain 1B (RBP2-like); lysine (K)-specific demethylase 5B" | 4.1368 | 3.0635 | 0.7405 | 0.0083 |
| Znfx1 | "zinc finger, NFX1-type containing 1" | 4.1211 | 3.0526 | 0.7407 | 0.0021 |
| ERGIC1 | endoplasmic reticulum-golgi intermediate compartment (ERGIC) 1 | 4.7017 | 3.4835 | 0.7409 | 0.0080 |
| Ptpn9 | "protein tyrosine phosphatase, non-receptor type 9" | 7.1816 | 5.3221 | 0.7411 | 0.0069 |
| GTF2IRD1 | GTF2I repeat domain containing 1 | 2.4445 | 1.8136 | 0.7419 | 0.0023 |
| USP2 | ubiquitin specific peptidase 2 | 7.4710 | 5.5433 | 0.7420 | 0.0279 |
| PCDHB15 | protocadherin beta 15 | 2.0040 | 1.4879 | 0.7425 | 0.0121 |
| SMG6 | "Smg-6 homolog, nonsense mediated mRNA decay factor (C. elegans)" | 4.8923 | 3.6333 | 0.7427 | 0.0315 |
| Dhx38 | DEAH (Asp-Glu-Ala-His) box polypeptide 38 | 8.1183 | 6.0349 | 0.7434 | 0.0247 |
| RAB1B | "RAB1B, member RAS oncogene family" | 26.9934 | 20.0741 | 0.7437 | 0.0301 |
| Add1 | adducin 1 (alpha) | 30.2131 | 22.5281 | 0.7456 | 0.0002 |
| ccdc97 | coiled-coil domain containing 97 | 5.8842 | 4.3917 | 0.7463 | 0.0145 |
| CTNND1 | "catenin (cadherin-associated protein), delta 1" | 12.9566 | 9.6726 | 0.7465 | 0.0025 |
| Stat3 | signal transducer and activator of transcription 3 (acute-phase response factor) | 8.1291 | 6.0734 | 0.7471 | 0.0077 |
| RIC8A | resistance to inhibitors of cholinesterase 8 homolog A (C. elegans) | 11.3085 | 8.4500 | 0.7472 | 0.0058 |
| pelp1 | "proline, glutamate and leucine rich protein 1" | 16.0526 | 12.0024 | 0.7477 | 0.0226 |
| BRF1 | "BRF1 homolog, subunit of RNA polymerase III transcription initiation factor IIIB (S. cerevisiae)" | 2.2645 | 1.6933 | 0.7478 | 0.0490 |
| AUTS2 | autism susceptibility candidate 2 | 3.9732 | 2.9718 | 0.7480 | 0.0158 |
| SLC16A2 | "solute carrier family 16, member 2 (monocarboxylic acid transporter 8)" | 5.8084 | 4.3459 | 0.7482 | 0.0286 |
| Nup214 | nucleoporin 214kDa | 2.8965 | 2.1705 | 0.7494 | 0.0010 |
| CTNND2 | "catenin (cadherin-associated protein), delta 2 (neural plakophilin-related arm-repeat protein)" | 55.4649 | 41.6140 | 0.7503 | 0.0247 |
| ANAPC2 | anaphase promoting complex subunit 2 | 10.3033 | 7.7318 | 0.7504 | 0.0462 |
| OS9 | "osteosarcoma amplified 9, endoplasmic reticulum associated protein" | 19.1711 | 14.3890 | 0.7506 | 0.0391 |
| aspscr1 | "alveolar soft part sarcoma chromosome region, candidate 1" | 2.7309 | 2.0501 | 0.7507 | 0.0364 |
| CSNK1E | "casein kinase 1, epsilon" | 20.9454 | 15.7266 | 0.7508 | 0.0412 |
| tbc1d9b | "TBC1 domain family, member 9B (with GRAM domain)" | 17.5545 | 13.1845 | 0.7511 | 0.0478 |
| Il17ra | interleukin 17 receptor A | 1.4649 | 1.1002 | 0.7511 | 0.0372 |
| C7orf26 | chromosome 7 open reading frame 26 | 9.2172 | 6.9230 | 0.7511 | 0.0458 |
| SYVN1 | "synovial apoptosis inhibitor 1, synoviolin" | 7.8234 | 5.8842 | 0.7521 | 0.0276 |
| LOC653375 | RCC1-like G exchanging factor-like; Williams-Beuren syndrome chromosome region 16 | 6.6684 | 5.0158 | 0.7522 | 0.0083 |
| WBSCR16 | RCC1-like G exchanging factor-like; Williams-Beuren syndrome chromosome region 16 | 6.6684 | 5.0158 | 0.7522 | 0.0083 |
| slc22a5 | "solute carrier family 22 (organic cation/carnitine transporter), member 5" | 1.0413 | 0.7836 | 0.7525 | 0.0234 |
| TRAK1 | "trafficking protein, kinesin binding 1" | 10.0774 | 7.5850 | 0.7527 | 0.0454 |
| UBOX5 | U-box domain containing 5 | 2.7346 | 2.0601 | 0.7533 | 0.0132 |
| SCMH1 | sex comb on midleg homolog 1 (Drosophila) | 7.1409 | 5.3820 | 0.7537 | 0.0351 |
| Atxn7l2 | ataxin 7-like 2 | 3.0653 | 2.3107 | 0.7538 | 0.0289 |
| Plxna3 | plexin A3 | 3.9919 | 3.0092 | 0.7538 | 0.0246 |
| wdr1 | WD repeat domain 1 | 10.5378 | 7.9469 | 0.7541 | 0.0195 |
| CNNM3 | cyclin M3 | 4.2047 | 3.1735 | 0.7547 | 0.0367 |
| Fbxo10 | F-box protein 10 | 2.3296 | 1.7584 | 0.7548 | 0.0446 |
| akt2 | v-akt murine thymoma viral oncogene homolog 2 | 6.3150 | 4.7699 | 0.7553 | 0.0429 |
| Cdan1 | "congenital dyserythropoietic anemia, type I" | 3.3567 | 2.5397 | 0.7566 | 0.0190 |
| Frmd4a | FERM domain containing 4A | 3.4255 | 2.5922 | 0.7567 | 0.0220 |
| LOC729799 | SEC14-like 1 (S. cerevisiae); SEC14-like 1 pseudogene | 10.5759 | 8.0047 | 0.7569 | 0.0030 |
| sec14l1 | SEC14-like 1 (S. cerevisiae); SEC14-like 1 pseudogene | 10.5759 | 8.0047 | 0.7569 | 0.0030 |
| ACCN2 | "amiloride-sensitive cation channel 2, neuronal" | 10.6198 | 8.0431 | 0.7574 | 0.0164 |
| VAC14 | Vac14 homolog (S. cerevisiae) | 4.7573 | 3.6116 | 0.7592 | 0.0431 |
| ZNF212 | zinc finger protein 212 | 3.2627 | 2.4780 | 0.7595 | 0.0154 |
| smcr7 | "Smith-Magenis syndrome chromosome region, candidate 7" | 3.9310 | 2.9898 | 0.7606 | 0.0184 |
| AGPAT1 | "1-acylglycerol-3-phosphate O-acyltransferase 1 (lysophosphatidic acid acyltransferase, alpha)" | 15.6525 | 11.9117 | 0.7610 | 0.0145 |
| AKAP1 | A kinase (PRKA) anchor protein 1 | 11.0159 | 8.4037 | 0.7629 | 0.0035 |
| Usp42 | ubiquitin specific peptidase 42 | 2.1415 | 1.6347 | 0.7633 | 0.0400 |
| OLFM1 | olfactomedin 1 | 140.4545 | 107.2560 | 0.7636 | 0.0412 |
| SLC25A15 | solute carrier family 25 (mitochondrial carrier; ornithine transporter) member 15 | 2.2050 | 1.6844 | 0.7639 | 0.0116 |
| PIAS3 | "protein inhibitor of activated STAT, 3" | 5.2348 | 4.0005 | 0.7642 | 0.0287 |
| TMEM132C | transmembrane protein 132C | 4.9229 | 3.7627 | 0.7643 | 0.0230 |
| OGG1 | 8-oxoguanine DNA glycosylase | 2.5927 | 1.9822 | 0.7645 | 0.0116 |
| SH2B3 | SH2B adaptor protein 3 | 3.4327 | 2.6263 | 0.7651 | 0.0454 |
| C17orf62 | chromosome 17 open reading frame 62 | 4.6373 | 3.5498 | 0.7655 | 0.0121 |
| MBD6 | methyl-CpG binding domain protein 6 | 4.9604 | 3.7983 | 0.7657 | 0.0188 |
| gpr173 | G protein-coupled receptor 173 | 5.9641 | 4.5715 | 0.7665 | 0.0284 |
| CSK | c-src tyrosine kinase | 5.9276 | 4.5438 | 0.7665 | 0.0490 |
| RILPL1 | Rab interacting lysosomal protein-like 1 | 6.2497 | 4.7951 | 0.7673 | 0.0443 |
| adcy8 | adenylate cyclase 8 (brain) | 2.7301 | 2.0966 | 0.7680 | 0.0334 |
| ZNF74 | zinc finger protein 74 | 2.8395 | 2.1807 | 0.7680 | 0.0442 |
| GRID1 | "glutamate receptor, ionotropic, delta 1" | 7.8316 | 6.0184 | 0.7685 | 0.0205 |
| DHX34 | DEAH (Asp-Glu-Ala-His) box polypeptide 34 | 3.6276 | 2.7902 | 0.7692 | 0.0398 |
| sumf2 | sulfatase modifying factor 2 | 13.3352 | 10.2628 | 0.7696 | 0.0122 |
| GGA1 | "golgi associated, gamma adaptin ear containing, ARF binding protein 1" | 6.3336 | 4.8744 | 0.7696 | 0.0311 |
| Wdr6 | WD repeat domain 6 | 15.0644 | 11.5974 | 0.7699 | 0.0099 |
| ADCY2 | adenylate cyclase 2 (brain) | 9.2131 | 7.0994 | 0.7706 | 0.0205 |
| pigo | "phosphatidylinositol glycan anchor biosynthesis, class O" | 2.2470 | 1.7318 | 0.7707 | 0.0225 |
| PARS2 | "prolyl-tRNA synthetase 2, mitochondrial (putative)" | 1.3621 | 1.0501 | 0.7710 | 0.0126 |
| WWP2 | WW domain containing E3 ubiquitin protein ligase 2 | 6.4327 | 4.9631 | 0.7715 | 0.0462 |
| MUL1 | mitochondrial E3 ubiquitin ligase 1 | 10.0322 | 7.7416 | 0.7717 | 0.0042 |
| atad3a | "ATPase family, AAA domain containing 3A" | 5.6091 | 4.3307 | 0.7721 | 0.0319 |
| ZNF784 | zinc finger protein 784 | 2.5150 | 1.9426 | 0.7724 | 0.0407 |
| C20orf4 | chromosome 20 open reading frame 4 | 4.7765 | 3.6909 | 0.7727 | 0.0316 |
| taf4 | "TAF4 RNA polymerase II, TATA box binding protein (TBP)-associated factor, 135kDa" | 2.5579 | 1.9784 | 0.7735 | 0.0047 |
| FZD4 | frizzled homolog 4 (Drosophila) | 2.0173 | 1.5616 | 0.7741 | 0.0154 |
| FAM20C | "family with sequence similarity 20, member C" | 6.7381 | 5.2256 | 0.7755 | 0.0427 |
| Sf1 | splicing factor 1 | 30.1204 | 23.3721 | 0.7760 | 0.0063 |
| Nkiras2 | NFKB inhibitor interacting Ras-like 2 | 2.4746 | 1.9223 | 0.7768 | 0.0112 |
| KDM3B | lysine (K)-specific demethylase 3B | 8.6393 | 6.7115 | 0.7769 | 0.0122 |
| RNF216L | ring finger protein 216-like | 1.8240 | 1.4178 | 0.7773 | 0.0048 |
| C4orf42 | chromosome 4 open reading frame 42 | 1.8446 | 1.4339 | 0.7773 | 0.0202 |
| jarid2 | "jumonji, AT rich interactive domain 2" | 3.9277 | 3.0567 | 0.7782 | 0.0296 |
| TMEM39B | transmembrane protein 39B | 3.5561 | 2.7677 | 0.7783 | 0.0035 |
| SRRM2 | serine/arginine repetitive matrix 2; hypothetical LOC100132779 | 33.0368 | 25.7140 | 0.7783 | 0.0475 |
| LOC100132779 | serine/arginine repetitive matrix 2; hypothetical LOC100132779 | 33.0368 | 25.7140 | 0.7783 | 0.0475 |
| UBTF | "upstream binding transcription factor, RNA polymerase I" | 13.9693 | 10.8746 | 0.7785 | 0.0077 |
| fam171a1 | "family with sequence similarity 171, member A1" | 42.1218 | 32.8081 | 0.7789 | 0.0478 |
| ELMO2 | engulfment and cell motility 2 | 13.5769 | 10.5764 | 0.7790 | 0.0021 |
| Mapkap1 | mitogen-activated protein kinase associated protein 1 | 12.3095 | 9.5923 | 0.7793 | 0.0481 |
| PCIF1 | PDX1 C-terminal inhibiting factor 1 | 12.0303 | 9.3807 | 0.7798 | 0.0245 |
| SURF4 | surfeit 4 | 24.5039 | 19.1205 | 0.7803 | 0.0034 |
| PGAP3 | post-GPI attachment to proteins 3 | 6.6053 | 5.1561 | 0.7806 | 0.0435 |
| DIS3L2 | DIS3 mitotic control homolog (S. cerevisiae)-like 2 | 1.8652 | 1.4560 | 0.7806 | 0.0349 |
| Lpcat3 | lysophosphatidylcholine acyltransferase 3 | 2.1228 | 1.6580 | 0.7811 | 0.0142 |
| Fam193a | chromosome 4 open reading frame 8 | 5.2720 | 4.1202 | 0.7815 | 0.0116 |
| Gorasp1 | "golgi reassembly stacking protein 1, 65kDa" | 8.6460 | 6.7617 | 0.7821 | 0.0113 |
| TXLNA | taxilin alpha | 8.2049 | 6.4189 | 0.7823 | 0.0133 |
| Rbm12 | RNA binding motif protein 12; copine I | 10.9789 | 8.5895 | 0.7824 | 0.0157 |
| CPNE1 | RNA binding motif protein 12; copine I | 10.9789 | 8.5895 | 0.7824 | 0.0157 |
| COLQ | collagen-like tail subunit (single strand of homotrimer) of asymmetric acetylcholinesterase | 2.4250 | 1.8974 | 0.7824 | 0.0482 |
| Zbtb9 | zinc finger and BTB domain containing 9 | 2.9240 | 2.2890 | 0.7828 | 0.0094 |
| UBE2J2 | "ubiquitin-conjugating enzyme E2, J2 (UBC6 homolog, yeast)" | 7.6021 | 5.9580 | 0.7837 | 0.0398 |
| Ino80 | INO80 homolog (S. cerevisiae) | 3.5608 | 2.7916 | 0.7840 | 0.0113 |
| dcaf7 | WD repeat domain 68 | 13.9802 | 10.9604 | 0.7840 | 0.0347 |
| SLC39A13 | "solute carrier family 39 (zinc transporter), member 13" | 9.0266 | 7.0789 | 0.7842 | 0.0356 |
| SMARCD1 | "SWI/SNF related, matrix associated, actin dependent regulator of chromatin, subfamily d, member 1" | 7.7733 | 6.1069 | 0.7856 | 0.0011 |
| SMAD7 | SMAD family member 7 | 6.0716 | 4.7738 | 0.7862 | 0.0474 |
| XRCC1 | X-ray repair complementing defective repair in Chinese hamster cells 1 | 5.2198 | 4.1046 | 0.7863 | 0.0498 |
| Baz2a | "bromodomain adjacent to zinc finger domain, 2A" | 6.7594 | 5.3180 | 0.7868 | 0.0409 |
| snx15 | sorting nexin 15 | 5.1705 | 4.0686 | 0.7869 | 0.0173 |
| ZDHHC18 | "zinc finger, DHHC-type containing 18" | 5.2989 | 4.1737 | 0.7877 | 0.0261 |
| N4BP1 | NEDD4 binding protein 1 | 4.6851 | 3.6904 | 0.7877 | 0.0239 |
| ZNF586 | zinc finger protein 586 | 1.0831 | 0.8533 | 0.7878 | 0.0428 |
| unk | unkempt homolog (Drosophila) | 3.4343 | 2.7100 | 0.7891 | 0.0206 |
| ZSWIM5 | "zinc finger, SWIM-type containing 5" | 1.8225 | 1.4392 | 0.7897 | 0.0065 |
| CHST10 | carbohydrate sulfotransferase 10 | 11.0981 | 8.7751 | 0.7907 | 0.0364 |
| thrA | "thyroid hormone receptor, alpha (erythroblastic leukemia viral (v-erb-a) oncogene homolog, avian)" | 43.0361 | 34.0557 | 0.7913 | 0.0486 |
| RMND5B | required for meiotic nuclear division 5 homolog B (S. cerevisiae) | 6.1302 | 4.8513 | 0.7914 | 0.0450 |
| CCDC123 | coiled-coil domain containing 123 | 1.6958 | 1.3424 | 0.7916 | 0.0314 |
| ilf3 | "interleukin enhancer binding factor 3, 90kDa" | 15.5324 | 12.2988 | 0.7918 | 0.0243 |
| TXNDC11 | thioredoxin domain containing 11 | 3.5952 | 2.8506 | 0.7929 | 0.0018 |
| DDX49 | DEAD (Asp-Glu-Ala-Asp) box polypeptide 49 | 10.2130 | 8.1041 | 0.7935 | 0.0493 |
| nadK | NAD kinase | 6.8709 | 5.4566 | 0.7942 | 0.0291 |
| ptpn1 | "protein tyrosine phosphatase, non-receptor type 1" | 7.7190 | 6.1357 | 0.7949 | 0.0127 |
| Man1b1 | "mannosidase, alpha, class 1B, member 1" | 7.1249 | 5.6654 | 0.7952 | 0.0364 |
| vopp1 | similar to EGFR-coamplified and overexpressed protein; EGFR-coamplified and overexpressed protein | 15.1525 | 12.0563 | 0.7957 | 0.0453 |
| LOC729086 | similar to EGFR-coamplified and overexpressed protein; EGFR-coamplified and overexpressed protein | 15.1525 | 12.0563 | 0.7957 | 0.0453 |
| DNAJB12 | "DnaJ (Hsp40) homolog, subfamily B, member 12" | 6.7716 | 5.3884 | 0.7957 | 0.0102 |
| abcb8 | "ATP-binding cassette, sub-family B (MDR/TAP), member 8" | 4.6360 | 3.6905 | 0.7960 | 0.0491 |
| LOC653303 | proprotein convertase subtilisin/kexin type 7 pseudogene; proprotein convertase subtilisin/kexin type 7 | 2.5786 | 2.0534 | 0.7963 | 0.0414 |
| pcsk7 | proprotein convertase subtilisin/kexin type 7 pseudogene; proprotein convertase subtilisin/kexin type 7 | 2.5786 | 2.0534 | 0.7963 | 0.0414 |
| PHF8 | PHD finger protein 8 | 2.4413 | 1.9459 | 0.7971 | 0.0304 |
| TFIP11 | tuftelin interacting protein 11 | 5.7776 | 4.6090 | 0.7977 | 0.0020 |
| TARS2 | "threonyl-tRNA synthetase 2, mitochondrial (putative)" | 7.8798 | 6.2912 | 0.7984 | 0.0341 |
| ZBTB49 | zinc finger protein 509 | 1.4947 | 1.1958 | 0.8000 | 0.0359 |
| DDB1 | "damage-specific DNA binding protein 1, 127kDa" | 15.2196 | 12.1907 | 0.8010 | 0.0442 |
| pfkfb3 | "6-phosphofructo-2-kinase/fructose-2,6-biphosphatase 3" | 21.7368 | 17.4299 | 0.8019 | 0.0204 |
| ITPK1 | "inositol 1,3,4-triphosphate 5/6 kinase" | 48.5985 | 39.0349 | 0.8032 | 0.0497 |
| PIGS | "phosphatidylinositol glycan anchor biosynthesis, class S" | 6.5087 | 5.2331 | 0.8040 | 0.0186 |
| POLDIP3 | "polymerase (DNA-directed), delta interacting protein 3" | 18.7054 | 15.0461 | 0.8044 | 0.0215 |
| Tyk2 | tyrosine kinase 2 | 7.6316 | 6.1411 | 0.8047 | 0.0398 |
| PES1 | "pescadillo homolog 1, containing BRCT domain (zebrafish)" | 8.2647 | 6.6579 | 0.8056 | 0.0307 |
| GLG1 | golgi apparatus protein 1 | 12.5262 | 10.1027 | 0.8065 | 0.0250 |
| CCNK | cyclin K | 3.0956 | 2.4975 | 0.8068 | 0.0457 |
| Recql5 | RecQ protein-like 5 | 2.6736 | 2.1596 | 0.8078 | 0.0046 |
| RBM28 | RNA binding motif protein 28 | 4.5738 | 3.6971 | 0.8083 | 0.0081 |
| Nfkbie | "nuclear factor of kappa light polypeptide gene enhancer in B-cells inhibitor, epsilon" | 3.2525 | 2.6314 | 0.8090 | 0.0481 |
| SPRY2 | sprouty homolog 2 (Drosophila) | 17.5777 | 14.2234 | 0.8092 | 0.0389 |
| ALG1 | "asparagine-linked glycosylation 1, beta-1,4-mannosyltransferase homolog (S. cerevisiae)" | 1.6328 | 1.3243 | 0.8111 | 0.0156 |
| SLC2A8 | "solute carrier family 2 (facilitated glucose transporter), member 8" | 5.2511 | 4.2647 | 0.8122 | 0.0408 |
| ATXN2 | ataxin 2 | 5.3826 | 4.3738 | 0.8126 | 0.0408 |
| IKBKB | "inhibitor of kappa light polypeptide gene enhancer in B-cells, kinase beta" | 2.2280 | 1.8122 | 0.8133 | 0.0348 |
| ARFGAP2 | ADP-ribosylation factor GTPase activating protein 2 | 17.9942 | 14.6851 | 0.8161 | 0.0372 |
| Tmem214 | transmembrane protein 214 | 7.1334 | 5.8226 | 0.8162 | 0.0331 |
| PTPRA | "protein tyrosine phosphatase, receptor type, A" | 45.5479 | 37.2446 | 0.8177 | 0.0006 |
| FAM193B | hypothetical protein FLJ10404 | 6.9542 | 5.6867 | 0.8177 | 0.0462 |
| ZSWIM1 | "zinc finger, SWIM-type containing 1" | 2.9985 | 2.4523 | 0.8179 | 0.0016 |
| DVL2 | "dishevelled, dsh homolog 2 (Drosophila)" | 4.6188 | 3.7792 | 0.8182 | 0.0487 |
| PMPCA | peptidase (mitochondrial processing) alpha | 9.2356 | 7.5584 | 0.8184 | 0.0292 |
| SIN3A | "SIN3 homolog A, transcription regulator (yeast)" | 2.7784 | 2.2796 | 0.8204 | 0.0007 |
| Setdb1 | "SET domain, bifurcated 1" | 2.1075 | 1.7296 | 0.8207 | 0.0394 |
| Cbx8 | "chromobox homolog 8 (Pc class homolog, Drosophila)" | 1.1400 | 0.9359 | 0.8210 | 0.0433 |
| PPP1R10 | "protein phosphatase 1, regulatory (inhibitor) subunit 10" | 9.0365 | 7.4368 | 0.8230 | 0.0349 |
| Setd8 | SET domain containing (lysine methyltransferase) 8 | 6.8334 | 5.6255 | 0.8232 | 0.0339 |
| DDX19A | DEAD (Asp-Glu-Ala-As) box polypeptide 19A | 3.7454 | 3.0905 | 0.8251 | 0.0086 |
| BRD1 | bromodomain containing 1 | 5.5031 | 4.5442 | 0.8257 | 0.0279 |
| tbc1d20 | "TBC1 domain family, member 20" | 9.6378 | 7.9610 | 0.8260 | 0.0310 |
| OSBPL2 | oxysterol binding protein-like 2 | 15.0828 | 12.4830 | 0.8276 | 0.0232 |
| sf3b3 | "splicing factor 3b, subunit 3, 130kDa" | 6.8275 | 5.6513 | 0.8277 | 0.0466 |
| Igf2r | insulin-like growth factor 2 receptor | 4.1334 | 3.4245 | 0.8285 | 0.0298 |
| TAF8 | "TAF8 RNA polymerase II, TATA box binding protein (TBP)-associated factor, 43kDa" | 1.8590 | 1.5415 | 0.8292 | 0.0369 |
| SPG7 | spastic paraplegia 7 (pure and complicated autosomal recessive) | 9.6957 | 8.0591 | 0.8312 | 0.0436 |
| DDX19B | DEAD (Asp-Glu-Ala-As) box polypeptide 19B | 1.5262 | 1.2698 | 0.8320 | 0.0203 |
| ACIN1 | apoptotic chromatin condensation inducer 1 | 11.3189 | 9.4284 | 0.8330 | 0.0396 |
| NRF1 | nuclear respiratory factor 1 | 4.1644 | 3.4850 | 0.8369 | 0.0275 |
| DCP1B | DCP1 decapping enzyme homolog B (S. cerevisiae) | 1.9460 | 1.6286 | 0.8369 | 0.0026 |
| USP4 | ubiquitin specific peptidase 4 (proto-oncogene) | 7.9340 | 6.6453 | 0.8376 | 0.0175 |
| LOC644006 | ring finger protein 4; hypothetical LOC644006 | 6.9665 | 5.8405 | 0.8384 | 0.0435 |
| Rnf4 | ring finger protein 4; hypothetical LOC644006 | 6.9665 | 5.8405 | 0.8384 | 0.0435 |
| CHD4 | chromodomain helicase DNA binding protein 4 | 15.0869 | 12.6487 | 0.8384 | 0.0038 |
| STAG3L3 | aminoacyl tRNA synthetase complex-interacting multifunctional protein 2; stromal antigen 3-like 3 | 3.0909 | 2.5961 | 0.8399 | 0.0434 |
| AIMP2 | aminoacyl tRNA synthetase complex-interacting multifunctional protein 2; stromal antigen 3-like 3 | 3.0909 | 2.5961 | 0.8399 | 0.0434 |
| BRD9 | bromodomain containing 9 | 3.9077 | 3.2956 | 0.8434 | 0.0338 |
| ISG20L2 | interferon stimulated exonuclease gene 20kDa-like 2 | 3.3740 | 2.8465 | 0.8436 | 0.0255 |
| URB2 | URB2 ribosome biogenesis 2 homolog (S. cerevisiae) | 1.2282 | 1.0371 | 0.8444 | 0.0156 |
| ZNF394 | zinc finger protein 394 | 2.4601 | 2.0775 | 0.8445 | 0.0150 |
| SDR39U1 | "short chain dehydrogenase/reductase family 39U, member 1" | 5.1554 | 4.3603 | 0.8458 | 0.0345 |
| rabl4 | "RAB, member of RAS oncogene family-like 4" | 4.0978 | 3.4659 | 0.8458 | 0.0377 |
| pdia6 | "protein disulfide isomerase family A, member 6" | 10.5822 | 8.9634 | 0.8470 | 0.0348 |
| Syt7 | synaptotagmin XVII; synaptotagmin VII | 3.7120 | 3.1533 | 0.8495 | 0.0121 |
| syt17 | synaptotagmin XVII; synaptotagmin VII | 3.7120 | 3.1533 | 0.8495 | 0.0121 |
| NUP188 | nucleoporin 188kDa | 3.0371 | 2.5808 | 0.8498 | 0.0031 |
| ZNF384 | zinc finger protein 384 | 5.5541 | 4.7207 | 0.8499 | 0.0105 |
| BCKDK | branched chain ketoacid dehydrogenase kinase | 7.4545 | 6.3374 | 0.8501 | 0.0406 |
| MYST4 | MYST histone acetyltransferase (monocytic leukemia) 4 | 2.9195 | 2.4820 | 0.8501 | 0.0473 |
| LCMT2 | leucine carboxyl methyltransferase 2 | 2.5098 | 2.1340 | 0.8503 | 0.0380 |
| GTF3C2 | "general transcription factor IIIC, polypeptide 2, beta 110kDa" | 3.8431 | 3.2732 | 0.8517 | 0.0484 |
| MTMR14 | myotubularin related protein 14 | 3.4407 | 2.9359 | 0.8533 | 0.0370 |
| EDC3 | enhancer of mRNA decapping 3 homolog (S. cerevisiae) | 4.6259 | 3.9493 | 0.8537 | 0.0185 |
| KIAA1539 | KIAA1539 | 5.0642 | 4.3264 | 0.8543 | 0.0325 |
| ZNF275 | zinc finger protein 275 | 3.6184 | 3.0919 | 0.8545 | 0.0086 |
| PI4KB | "phosphatidylinositol 4-kinase, catalytic, beta" | 13.1780 | 11.2703 | 0.8552 | 0.0410 |
| nonO | "non-POU domain containing, octamer-binding" | 18.0009 | 15.4396 | 0.8577 | 0.0418 |
| Tssc1 | tumor suppressing subtransferable candidate 1 | 5.4480 | 4.7119 | 0.8649 | 0.0412 |
| RPA1 | "replication protein A1, 70kDa" | 9.5789 | 8.3335 | 0.8700 | 0.0175 |
| sppl3 | signal peptide peptidase 3 | 9.1606 | 7.9940 | 0.8727 | 0.0281 |
| LRRC41 | leucine rich repeat containing 41 | 6.7931 | 5.9507 | 0.8760 | 0.0309 |
| THRAP3 | thyroid hormone receptor associated protein 3 | 20.5235 | 17.9823 | 0.8762 | 0.0168 |
| Tor1b | "torsin family 1, member B (torsin B)" | 5.5731 | 4.8925 | 0.8779 | 0.0228 |
| RNF185 | ring finger protein 185 | 8.6912 | 7.6557 | 0.8809 | 0.0451 |
| RAF1 | v-raf-1 murine leukemia viral oncogene homolog 1 | 7.5849 | 6.7339 | 0.8878 | 0.0486 |
| CPSF7 | "cleavage and polyadenylation specific factor 7, 59kDa" | 12.6494 | 11.2499 | 0.8894 | 0.0219 |
| SMARCAL1 | "SWI/SNF related, matrix associated, actin dependent regulator of chromatin, subfamily a-like 1" | 2.5828 | 2.2989 | 0.8901 | 0.0418 |
| nudt19 | nudix (nucleoside diphosphate linked moiety X)-type motif 19 | 1.8987 | 1.6912 | 0.8907 | 0.0472 |
| Tceb3 | "transcription elongation factor B (SIII), polypeptide 3 (110kDa, elongin A)" | 4.3390 | 3.9216 | 0.9038 | 0.0431 |
| RBM23 | RNA binding motif protein 23 | 6.8816 | 6.2844 | 0.9132 | 0.0428 |
| tbl2 | transducin (beta)-like 2 | 2.7838 | 2.5780 | 0.9261 | 0.0498 |
| fam192a | NEFA-interacting nuclear protein NIP30 | 21.6841 | 23.7124 | 1.0935 | 0.0290 |
| DRG1 | developmentally regulated GTP binding protein 1 | 15.6299 | 17.3940 | 1.1129 | 0.0351 |
| DKC1 | "dyskeratosis congenita 1, dyskerin" | 4.8087 | 5.3907 | 1.1210 | 0.0453 |
| RNF146 | ring finger protein 146 | 19.9319 | 22.4160 | 1.1246 | 0.0280 |
| MAGEF1 | "melanoma antigen family F, 1" | 25.9904 | 29.5570 | 1.1372 | 0.0204 |
| aldh3a2 | "aldehyde dehydrogenase 3 family, member A2" | 7.3440 | 8.3636 | 1.1388 | 0.0356 |
| Tmem111 | transmembrane protein 111 | 9.4315 | 10.8148 | 1.1467 | 0.0335 |
| Ttf1 | "transcription termination factor, RNA polymerase I" | 4.0148 | 4.6305 | 1.1533 | 0.0381 |
| DUSP12 | dual specificity phosphatase 12 | 5.4872 | 6.3297 | 1.1535 | 0.0411 |
| ube2d2 | "ubiquitin-conjugating enzyme E2D 2 (UBC4/5 homolog, yeast)" | 24.0383 | 27.8041 | 1.1567 | 0.0408 |
| trim69 | tripartite motif-containing 69 | 5.0354 | 5.8716 | 1.1661 | 0.0059 |
| CNIH4 | cornichon homolog 4 (Drosophila) | 4.3500 | 5.0856 | 1.1691 | 0.0144 |
| MRFAP1L1 | Morf4 family associated protein 1-like 1 | 36.7697 | 43.1531 | 1.1736 | 0.0465 |
| Khdrbs1 | "KH domain containing, RNA binding, signal transduction associated 1" | 20.6421 | 24.2845 | 1.1765 | 0.0278 |
| MTRF1L | mitochondrial translational release factor 1-like | 5.3067 | 6.2486 | 1.1775 | 0.0197 |
| ALG2 | "asparagine-linked glycosylation 2, alpha-1,3-mannosyltransferase homolog (S. cerevisiae)" | 4.6835 | 5.5258 | 1.1798 | 0.0400 |
| LOC645086 | chromosome 11 open reading frame 58 pseudogene; chromosome 11 open reading frame 58 | 12.3791 | 14.6211 | 1.1811 | 0.0284 |
| C11orf58 | chromosome 11 open reading frame 58 pseudogene; chromosome 11 open reading frame 58 | 12.3791 | 14.6211 | 1.1811 | 0.0284 |
| IFNGR2 | interferon gamma receptor 2 (interferon gamma transducer 1) | 6.5223 | 7.7048 | 1.1813 | 0.0424 |
| TPST1 | tyrosylprotein sulfotransferase 1 | 4.7324 | 5.5906 | 1.1813 | 0.0062 |
| LACTB | "lactamase, beta" | 2.1360 | 2.5256 | 1.1824 | 0.0460 |
| fam185a | "family with sequence similarity 185, member A; family with sequence similarity 185, member B pseudogene" | 1.8217 | 2.1651 | 1.1885 | 0.0193 |
| FAM185B | "family with sequence similarity 185, member A; family with sequence similarity 185, member B pseudogene" | 1.8217 | 2.1651 | 1.1885 | 0.0193 |
| Cops5 | COP9 constitutive photomorphogenic homolog subunit 5 (Arabidopsis) | 5.3848 | 6.4152 | 1.1914 | 0.0207 |
| tdrd7 | tudor domain containing 7 | 5.0575 | 6.0340 | 1.1931 | 0.0332 |
| PEBP1 | phosphatidylethanolamine binding protein 1 | 357.6730 | 428.5487 | 1.1982 | 0.0445 |
| msrA | methionine sulfoxide reductase A | 3.8232 | 4.5900 | 1.2006 | 0.0363 |
| mphosph10 | M-phase phosphoprotein 10 (U3 small nucleolar ribonucleoprotein) | 5.1196 | 6.1509 | 1.2014 | 0.0327 |
| trappc3 | trafficking protein particle complex 3 | 9.0146 | 10.8512 | 1.2037 | 0.0047 |
| RPGR | retinitis pigmentosa GTPase regulator | 1.0295 | 1.2408 | 1.2053 | 0.0389 |
| TP53RK | TP53 regulating kinase | 2.4041 | 2.8986 | 1.2057 | 0.0403 |
| CAMLG | calcium modulating ligand | 23.5954 | 28.5246 | 1.2089 | 0.0397 |
| Ccndbp1 | cyclin D-type binding-protein 1 | 8.5382 | 10.3262 | 1.2094 | 0.0337 |
| ZDHHC6 | "zinc finger, DHHC-type containing 6" | 8.1599 | 9.8961 | 1.2128 | 0.0358 |
| cct4 | "chaperonin containing TCP1, subunit 4 (delta)" | 26.5994 | 32.3136 | 1.2148 | 0.0498 |
| GGPS1 | geranylgeranyl diphosphate synthase 1 | 5.5477 | 6.7527 | 1.2172 | 0.0174 |
| Atp1b3 | "ATPase, Na+/K+ transporting, beta 3 polypeptide" | 23.3314 | 28.4238 | 1.2183 | 0.0341 |
| Dnaja1 | "DnaJ (Hsp40) homolog, subfamily A, member 1" | 80.1880 | 97.7276 | 1.2187 | 0.0464 |
| C9orf40 | chromosome 9 open reading frame 40 | 4.2906 | 5.2294 | 1.2188 | 0.0387 |
| nudt9 | nudix (nucleoside diphosphate linked moiety X)-type motif 9 | 9.6405 | 11.7644 | 1.2203 | 0.0406 |
| Ran | "RAN, member RAS oncogene family" | 31.2134 | 38.1510 | 1.2223 | 0.0447 |
| MED10 | mediator complex subunit 10 | 14.1460 | 17.3245 | 1.2247 | 0.0378 |
| FH | fumarate hydratase | 18.1925 | 22.2808 | 1.2247 | 0.0356 |
| NOP58 | NOP58 ribonucleoprotein homolog (yeast) | 7.7725 | 9.5283 | 1.2259 | 0.0353 |
| OAT | ornithine aminotransferase (gyrate atrophy) | 43.6686 | 53.6049 | 1.2275 | 0.0256 |
| Sumo1 | SMT3 suppressor of mif two 3 homolog 1 (S. cerevisiae); SUMO1 pseudogene 3 | 36.5042 | 44.8853 | 1.2296 | 0.0116 |
| SUMO1P3 | SMT3 suppressor of mif two 3 homolog 1 (S. cerevisiae); SUMO1 pseudogene 3 | 36.5042 | 44.8853 | 1.2296 | 0.0116 |
| ATP6V1G1 | "ATPase, H+ transporting, lysosomal 13kDa, V1 subunit G1" | 44.1393 | 54.3657 | 1.2317 | 0.0394 |
| PRNP | prion protein | 194.6768 | 239.9896 | 1.2328 | 0.0297 |
| MBD4 | methyl-CpG binding domain protein 4 | 6.8929 | 8.5125 | 1.2350 | 0.0185 |
| Chchd4 | coiled-coil-helix-coiled-coil-helix domain containing 4 | 7.2032 | 8.8984 | 1.2353 | 0.0273 |
| iscU | iron-sulfur cluster scaffold homolog (E. coli) | 25.6456 | 31.7298 | 1.2372 | 0.0201 |
| MED6 | mediator complex subunit 6 | 2.1993 | 2.7225 | 1.2379 | 0.0306 |
| LOC100132973 | "similar to elongin C; transcription elongation factor B (SIII), polypeptide 1 (15kDa, elongin C)" | 11.1647 | 13.8209 | 1.2379 | 0.0119 |
| TCEB1 | "similar to elongin C; transcription elongation factor B (SIII), polypeptide 1 (15kDa, elongin C)" | 11.1647 | 13.8209 | 1.2379 | 0.0119 |
| POLE3 | "polymerase (DNA directed), epsilon 3 (p17 subunit)" | 10.0650 | 12.4613 | 1.2381 | 0.0436 |
| C7orf44 | chromosome 7 open reading frame 44 | 2.4124 | 2.9871 | 1.2382 | 0.0093 |
| vdac2 | voltage-dependent anion channel 2 | 30.1831 | 37.4190 | 1.2397 | 0.0242 |
| c7orf30 | chromosome 7 open reading frame 30 | 2.9301 | 3.6361 | 1.2409 | 0.0325 |
| PSMD14 | "proteasome (prosome, macropain) 26S subunit, non-ATPase, 14" | 5.6621 | 7.0287 | 1.2414 | 0.0086 |
| Gtf2b | general transcription factor IIB | 5.9925 | 7.4504 | 1.2433 | 0.0058 |
| WDYHV1 | WDYHV motif containing 1 | 3.3396 | 4.1550 | 1.2441 | 0.0218 |
| LYRM4 | LYR motif containing 4 | 2.3992 | 2.9850 | 1.2441 | 0.0279 |
| Hexb | hexosaminidase B (beta polypeptide) | 4.8268 | 6.0067 | 1.2445 | 0.0237 |
| IQCB1 | IQ motif containing B1 | 4.6482 | 5.7895 | 1.2456 | 0.0445 |
| Psma4 | "proteasome (prosome, macropain) subunit, alpha type, 4" | 12.2197 | 15.2324 | 1.2465 | 0.0208 |
| HNRNPA1L2 | heterogeneous nuclear ribonucleoprotein A1-like 2 | 2.8500 | 3.5546 | 1.2472 | 0.0248 |
| Tmem81 | transmembrane protein 81 | 3.0012 | 3.7448 | 1.2478 | 0.0242 |
| MAP1LC3B | microtubule-associated protein 1 light chain 3 beta | 27.4360 | 34.2467 | 1.2482 | 0.0173 |
| c11orf57 | chromosome 11 open reading frame 57 | 4.7574 | 5.9479 | 1.2503 | 0.0387 |
| nfu1 | NFU1 iron-sulfur cluster scaffold homolog (S. cerevisiae) | 8.7201 | 10.9158 | 1.2518 | 0.0116 |
| Ttrap | TRAF and TNF receptor associated protein | 12.0318 | 15.0633 | 1.2520 | 0.0204 |
| C14orf142 | chromosome 14 open reading frame 142 | 4.8871 | 6.1192 | 1.2521 | 0.0401 |
| ptrh2 | peptidyl-tRNA hydrolase 2 | 2.0036 | 2.5148 | 1.2551 | 0.0163 |
| ENOPH1 | enolase-phosphatase 1 | 41.7421 | 52.4012 | 1.2554 | 0.0324 |
| RPL36AP51 | ribosomal protein L36a pseudogene 51; ribosomal protein L36a pseudogene 37; ribosomal protein L36a pseudogene 49; heterogeneous nuclear ribonucleoprotein H2 (H'); ribosomal protein L36a | 38.7841 | 48.7713 | 1.2575 | 0.0009 |
| Hnrnph2 | ribosomal protein L36a pseudogene 51; ribosomal protein L36a pseudogene 37; ribosomal protein L36a pseudogene 49; heterogeneous nuclear ribonucleoprotein H2 (H'); ribosomal protein L36a | 38.7841 | 48.7713 | 1.2575 | 0.0009 |
| RPL36A | ribosomal protein L36a pseudogene 51; ribosomal protein L36a pseudogene 37; ribosomal protein L36a pseudogene 49; heterogeneous nuclear ribonucleoprotein H2 (H'); ribosomal protein L36a | 38.7841 | 48.7713 | 1.2575 | 0.0009 |
| RPL36AP37 | ribosomal protein L36a pseudogene 51; ribosomal protein L36a pseudogene 37; ribosomal protein L36a pseudogene 49; heterogeneous nuclear ribonucleoprotein H2 (H'); ribosomal protein L36a | 38.7841 | 48.7713 | 1.2575 | 0.0009 |
| RPL36AP49 | ribosomal protein L36a pseudogene 51; ribosomal protein L36a pseudogene 37; ribosomal protein L36a pseudogene 49; heterogeneous nuclear ribonucleoprotein H2 (H'); ribosomal protein L36a | 38.7841 | 48.7713 | 1.2575 | 0.0009 |
| POLR2K | "polymerase (RNA) II (DNA directed) polypeptide K, 7.0kDa" | 27.2763 | 34.3029 | 1.2576 | 0.0103 |
| COQ3 | "coenzyme Q3 homolog, methyltransferase (S. cerevisiae)" | 5.9310 | 7.4665 | 1.2589 | 0.0178 |
| Gtf2e2 | "general transcription factor IIE, polypeptide 2, beta 34kDa" | 3.7030 | 4.6620 | 1.2590 | 0.0437 |
| set | SET nuclear oncogene; similar to SET translocation | 37.9024 | 47.8351 | 1.2621 | 0.0158 |
| LOC646817 | SET nuclear oncogene; similar to SET translocation | 37.9024 | 47.8351 | 1.2621 | 0.0158 |
| IGBP1 | chromosome 14 open reading frame 19; immunoglobulin (CD79A) binding protein 1 | 12.9580 | 16.3881 | 1.2647 | 0.0089 |
| C14orf19 | chromosome 14 open reading frame 19; immunoglobulin (CD79A) binding protein 1 | 12.9580 | 16.3881 | 1.2647 | 0.0089 |
| CLTA | "clathrin, light chain (Lca)" | 32.7494 | 41.4717 | 1.2663 | 0.0264 |
| KLHL7 | kelch-like 7 (Drosophila) | 6.6774 | 8.4567 | 1.2665 | 0.0397 |
| THYN1 | thymocyte nuclear protein 1 | 16.1817 | 20.5060 | 1.2672 | 0.0045 |
| RPL26L1 | ribosomal protein L26-like 1 | 7.1611 | 9.0771 | 1.2676 | 0.0457 |
| Exosc3 | exosome component 3 | 2.2130 | 2.8094 | 1.2695 | 0.0276 |
| znrf2 | zinc and ring finger 2 | 1.6570 | 2.1051 | 1.2704 | 0.0224 |
| MEAF6 | chromosome 1 open reading frame 149 | 19.0067 | 24.1795 | 1.2722 | 0.0417 |
| VPS29 | vacuolar protein sorting 29 homolog (S. cerevisiae) | 14.7041 | 18.7135 | 1.2727 | 0.0118 |
| fundc1 | FUN14 domain containing 1 | 14.4985 | 18.4653 | 1.2736 | 0.0373 |
| RfC4 | "replication factor C (activator 1) 4, 37kDa" | 1.9602 | 2.4995 | 1.2751 | 0.0314 |
| HDAC2 | histone deacetylase 2 | 2.9409 | 3.7506 | 1.2753 | 0.0475 |
| ENY2 | enhancer of yellow 2 homolog (Drosophila) | 4.5426 | 5.8035 | 1.2776 | 0.0085 |
| STARD3NL | STARD3 N-terminal like | 7.6498 | 9.7787 | 1.2783 | 0.0242 |
| c2orf47 | chromosome 2 open reading frame 47 | 3.8768 | 4.9558 | 1.2783 | 0.0295 |
| MRPL22 | mitochondrial ribosomal protein L22 | 2.1905 | 2.8016 | 1.2790 | 0.0359 |
| EIF3L | "eukaryotic translation initiation factor 3, subunit L" | 22.9869 | 29.5118 | 1.2839 | 0.0063 |
| CDKN1B | "cyclin-dependent kinase inhibitor 1B (p27, Kip1)" | 33.0760 | 42.5026 | 1.2850 | 0.0384 |
| EIF3J | "eukaryotic translation initiation factor 3, subunit J" | 15.8564 | 20.3812 | 1.2854 | 0.0412 |
| RSRC1 | arginine/serine-rich coiled-coil 1 | 4.4648 | 5.7417 | 1.2860 | 0.0400 |
| pdcd2l | programmed cell death 2-like | 1.4229 | 1.8298 | 1.2860 | 0.0261 |
| c7orf23 | chromosome 7 open reading frame 23 | 2.2121 | 2.8452 | 1.2862 | 0.0412 |
| ccdc99 | coiled-coil domain containing 99 | 1.3286 | 1.7090 | 1.2863 | 0.0427 |
| C9orf123 | chromosome 9 open reading frame 123 | 8.7170 | 11.2213 | 1.2873 | 0.0156 |
| FKBP3 | "FK506 binding protein 3, 25kDa" | 24.5721 | 31.6840 | 1.2894 | 0.0309 |
| MORF4 | mortality factor 4; mortality factor 4 like 1 | 55.3671 | 71.4091 | 1.2897 | 0.0023 |
| MORF4L1 | mortality factor 4; mortality factor 4 like 1 | 55.3671 | 71.4091 | 1.2897 | 0.0023 |
| ZFP28 | zinc finger protein 28 homolog (mouse) | 2.7257 | 3.5182 | 1.2908 | 0.0319 |
| NUDT5 | nudix (nucleoside diphosphate linked moiety X)-type motif 5 | 3.2763 | 4.2312 | 1.2915 | 0.0264 |
| PGRMC2 | progesterone receptor membrane component 2 | 9.8866 | 12.7719 | 1.2918 | 0.0280 |
| CCDC28A | coiled-coil domain containing 28A | 15.2136 | 19.6625 | 1.2924 | 0.0048 |
| ZC3H15 | zinc finger CCCH-type containing 15 | 19.7388 | 25.5187 | 1.2928 | 0.0349 |
| RWDD1L1 | RWD domain containing 1-like 1; RWD domain containing 1 | 7.0824 | 9.1722 | 1.2951 | 0.0225 |
| rwdd1 | RWD domain containing 1-like 1; RWD domain containing 1 | 7.0824 | 9.1722 | 1.2951 | 0.0225 |
| TAF9 | "TAF9 RNA polymerase II, TATA box binding protein (TBP)-associated factor, 32kDa" | 11.1702 | 14.4823 | 1.2965 | 0.0179 |
| MFF | mitochondrial fission factor | 11.3041 | 14.6560 | 1.2965 | 0.0387 |
| RAD51C | RAD51 homolog C (S. cerevisiae) | 4.3872 | 5.6970 | 1.2986 | 0.0453 |
| Zfp91-Cntf | zinc finger protein 91 homolog (mouse); ZFP91-CNTF readthrough transcript; ciliary neurotrophic factor | 10.7633 | 13.9897 | 1.2998 | 0.0383 |
| CNTF | zinc finger protein 91 homolog (mouse); ZFP91-CNTF readthrough transcript; ciliary neurotrophic factor | 10.7633 | 13.9897 | 1.2998 | 0.0383 |
| ZFP91 | zinc finger protein 91 homolog (mouse); ZFP91-CNTF readthrough transcript; ciliary neurotrophic factor | 10.7633 | 13.9897 | 1.2998 | 0.0383 |
| NOVA1 | neuro-oncological ventral antigen 1 | 8.6086 | 11.2011 | 1.3012 | 0.0446 |
| SNRPD1 | small nuclear ribonucleoprotein D1 polypeptide 16kDa; hypothetical protein LOC100129492 | 10.9566 | 14.2735 | 1.3027 | 0.0453 |
| LOC100129492 | small nuclear ribonucleoprotein D1 polypeptide 16kDa; hypothetical protein LOC100129492 | 10.9566 | 14.2735 | 1.3027 | 0.0453 |
| atp5h | "ATP synthase, H+ transporting, mitochondrial F0 complex, subunit d" | 20.0884 | 26.2424 | 1.3063 | 0.0238 |
| CENPJ | centromere protein J | 1.6887 | 2.2120 | 1.3099 | 0.0485 |
| Acn9 | ACN9 homolog (S. cerevisiae) | 3.1494 | 4.1265 | 1.3103 | 0.0238 |
| ap3s1 | "adaptor-related protein complex 3, sigma 1 subunit" | 16.9645 | 22.2451 | 1.3113 | 0.0376 |
| LOC440944 | hypothetical LOC440944 | 1.9936 | 2.6144 | 1.3114 | 0.0357 |
| Cox7a2l | cytochrome c oxidase subunit VIIa polypeptide 2 like | 13.8106 | 18.1211 | 1.3121 | 0.0018 |
| SMNDC1 | survival motor neuron domain containing 1 | 4.9125 | 6.4477 | 1.3125 | 0.0483 |
| HRASLS5 | "HRAS-like suppressor family, member 5" | 1.6557 | 2.1738 | 1.3130 | 0.0061 |
| c20orf30 | hypothetical LOC642975; chromosome 20 open reading frame 30 | 33.0314 | 43.4286 | 1.3148 | 0.0238 |
| LOC642975 | hypothetical LOC642975; chromosome 20 open reading frame 30 | 33.0314 | 43.4286 | 1.3148 | 0.0238 |
| Eapp | E2F-associated phosphoprotein | 9.7681 | 12.8449 | 1.3150 | 0.0255 |
| PIGH | "phosphatidylinositol glycan anchor biosynthesis, class H" | 4.0741 | 5.3604 | 1.3157 | 0.0007 |
| LYSMD2 | "LysM, putative peptidoglycan-binding, domain containing 2" | 13.3374 | 17.5498 | 1.3158 | 0.0067 |
| taf7 | "TAF7 RNA polymerase II, TATA box binding protein (TBP)-associated factor, 55kDa" | 23.3346 | 30.7051 | 1.3159 | 0.0373 |
| GTF3A | general transcription factor IIIA | 12.8450 | 16.9064 | 1.3162 | 0.0219 |
| FAM98B | "family with sequence similarity 98, member B" | 6.2005 | 8.1657 | 1.3169 | 0.0301 |
| UBXN8 | UBX domain protein 8 | 2.8277 | 3.7334 | 1.3203 | 0.0211 |
| ebag9 | "estrogen receptor binding site associated, antigen, 9" | 4.8201 | 6.3660 | 1.3207 | 0.0265 |
| PSMD10 | "proteasome (prosome, macropain) 26S subunit, non-ATPase, 10" | 14.8677 | 19.6457 | 1.3214 | 0.0295 |
| Atpif1 | ATPase inhibitory factor 1 | 33.8713 | 44.7751 | 1.3219 | 0.0235 |
| RGS2 | "regulator of G-protein signaling 2, 24kDa" | 8.0952 | 10.7064 | 1.3226 | 0.0346 |
| LOC550643 | hypothetical LOC550643 | 11.4796 | 15.1851 | 1.3228 | 0.0291 |
| C3orf26 | chromosome 3 open reading frame 26 | 4.5547 | 6.0319 | 1.3243 | 0.0062 |
| Gtpbp8 | GTP-binding protein 8 (putative) | 1.5963 | 2.1169 | 1.3261 | 0.0266 |
| DDX18 | DEAD (Asp-Glu-Ala-Asp) box polypeptide 18 | 4.4999 | 5.9708 | 1.3269 | 0.0159 |
| Psmc6 | "proteasome (prosome, macropain) 26S subunit, ATPase, 6" | 7.5535 | 10.0237 | 1.3270 | 0.0434 |
| vapa | "VAMP (vesicle-associated membrane protein)-associated protein A, 33kDa" | 11.5534 | 15.3528 | 1.3288 | 0.0345 |
| ociad2 | OCIA domain containing 2 | 6.4820 | 8.6228 | 1.3303 | 0.0113 |
| CACYBP | similar to calcyclin binding protein; calcyclin binding protein | 25.4883 | 33.9270 | 1.3311 | 0.0299 |
| LOC644877 | similar to calcyclin binding protein; calcyclin binding protein | 25.4883 | 33.9270 | 1.3311 | 0.0299 |
| IFIT1 | interferon-induced protein with tetratricopeptide repeats 1 | 11.8324 | 15.7505 | 1.3311 | 0.0011 |
| C6orf162 | chromosome 6 open reading frame 162 | 4.6516 | 6.1956 | 1.3319 | 0.0314 |
| SEC62 | SEC62 homolog (S. cerevisiae) | 26.2413 | 34.9884 | 1.3333 | 0.0314 |
| TRIAP1 | TP53 regulated inhibitor of apoptosis 1 | 6.3389 | 8.4600 | 1.3346 | 0.0457 |
| SPIN2B | "spindlin family, member 2B" | 3.5256 | 4.7058 | 1.3347 | 0.0205 |
| EIF1B | eukaryotic translation initiation factor 1B | 34.8068 | 46.4903 | 1.3357 | 0.0026 |
| itgb1bp1 | integrin beta 1 binding protein 1 | 10.7695 | 14.3956 | 1.3367 | 0.0061 |
| SFRS11 | "splicing factor, arginine/serine-rich 11" | 15.5581 | 20.8024 | 1.3371 | 0.0431 |
| CRBN | cereblon | 6.4397 | 8.6217 | 1.3388 | 0.0332 |
| PLEKHA3 | "pleckstrin homology domain containing, family A (phosphoinositide binding specific) member 3" | 6.2329 | 8.3522 | 1.3400 | 0.0295 |
| apoo | apolipoprotein O | 18.0476 | 24.2051 | 1.3412 | 0.0262 |
| CYCS | "cytochrome c, somatic" | 42.2950 | 56.7287 | 1.3413 | 0.0075 |
| SERINC1 | serine incorporator 1 | 197.3312 | 264.7223 | 1.3415 | 0.0287 |
| HSP90AA2 | "heat shock protein 90kDa alpha (cytosolic), class A member 2; heat shock protein 90kDa alpha (cytosolic), class A member 1" | 247.4170 | 332.9340 | 1.3456 | 0.0041 |
| HSP90AA1 | "heat shock protein 90kDa alpha (cytosolic), class A member 2; heat shock protein 90kDa alpha (cytosolic), class A member 1" | 247.4170 | 332.9340 | 1.3456 | 0.0041 |
| MTX2 | metaxin 2 | 18.7274 | 25.2039 | 1.3458 | 0.0190 |
| hnrpll | heterogeneous nuclear ribonucleoprotein L-like | 4.8379 | 6.5156 | 1.3468 | 0.0390 |
| RRP15 | ribosomal RNA processing 15 homolog (S. cerevisiae) | 1.5114 | 2.0379 | 1.3483 | 0.0402 |
| Tceal1 | transcription elongation factor A (SII)-like 1 | 17.9147 | 24.1871 | 1.3501 | 0.0151 |
| TCEAL8 | transcription elongation factor A (SII)-like 8 | 27.9782 | 37.7829 | 1.3504 | 0.0117 |
| Chmp5 | chromatin modifying protein 5 | 31.7762 | 42.9833 | 1.3527 | 0.0134 |
| UBE2F | ubiquitin-conjugating enzyme E2F (putative) | 2.5284 | 3.4227 | 1.3537 | 0.0044 |
| arsk | "arylsulfatase family, member K" | 1.2789 | 1.7314 | 1.3538 | 0.0134 |
| C12orf73 | chromosome 12 open reading frame 73 | 2.3931 | 3.2401 | 1.3539 | 0.0074 |
| C6orf130 | chromosome 6 open reading frame 130 | 2.5405 | 3.4404 | 1.3542 | 0.0489 |
| dynll1 | "dynein, light chain, LC8-type 1" | 70.1253 | 95.0437 | 1.3553 | 0.0371 |
| NARS2 | "asparaginyl-tRNA synthetase 2, mitochondrial (putative)" | 2.3820 | 3.2292 | 1.3557 | 0.0061 |
| PDCD10 | programmed cell death 10 | 6.6312 | 9.0087 | 1.3585 | 0.0362 |
| WBP4 | WW domain binding protein 4 (formin binding protein 21) | 9.3188 | 12.6628 | 1.3588 | 0.0049 |
| frg1 | FSHD region gene 1 | 8.5888 | 11.6713 | 1.3589 | 0.0184 |
| SFRS2B | "splicing factor, arginine/serine-rich 2B" | 3.9455 | 5.3640 | 1.3595 | 0.0468 |
| MRPL50 | mitochondrial ribosomal protein L50 | 6.8447 | 9.3139 | 1.3607 | 0.0199 |
| GLRX | glutaredoxin (thioltransferase) | 2.7564 | 3.7571 | 1.3630 | 0.0445 |
| RPL15P22 | ribosomal protein L15 pseudogene 22; ribosomal protein L15 pseudogene 18; ribosomal protein L15 pseudogene 17; ribosomal protein L15 pseudogene 3; ribosomal protein L15 pseudogene 7; ribosomal protein L15 | 62.5651 | 85.4534 | 1.3658 | 0.0204 |
| RPL15P18 | ribosomal protein L15 pseudogene 22; ribosomal protein L15 pseudogene 18; ribosomal protein L15 pseudogene 17; ribosomal protein L15 pseudogene 3; ribosomal protein L15 pseudogene 7; ribosomal protein L15 | 62.5651 | 85.4534 | 1.3658 | 0.0204 |
| RPL15P7 | ribosomal protein L15 pseudogene 22; ribosomal protein L15 pseudogene 18; ribosomal protein L15 pseudogene 17; ribosomal protein L15 pseudogene 3; ribosomal protein L15 pseudogene 7; ribosomal protein L15 | 62.5651 | 85.4534 | 1.3658 | 0.0204 |
| RPL15 | ribosomal protein L15 pseudogene 22; ribosomal protein L15 pseudogene 18; ribosomal protein L15 pseudogene 17; ribosomal protein L15 pseudogene 3; ribosomal protein L15 pseudogene 7; ribosomal protein L15 | 62.5651 | 85.4534 | 1.3658 | 0.0204 |
| RPL15P3 | ribosomal protein L15 pseudogene 22; ribosomal protein L15 pseudogene 18; ribosomal protein L15 pseudogene 17; ribosomal protein L15 pseudogene 3; ribosomal protein L15 pseudogene 7; ribosomal protein L15 | 62.5651 | 85.4534 | 1.3658 | 0.0204 |
| RPL15P17 | ribosomal protein L15 pseudogene 22; ribosomal protein L15 pseudogene 18; ribosomal protein L15 pseudogene 17; ribosomal protein L15 pseudogene 3; ribosomal protein L15 pseudogene 7; ribosomal protein L15 | 62.5651 | 85.4534 | 1.3658 | 0.0204 |
| NUDT2 | nudix (nucleoside diphosphate linked moiety X)-type motif 2 | 9.7999 | 13.3864 | 1.3660 | 0.0434 |
| TMEM126B | transmembrane protein 126B | 14.0413 | 19.1815 | 1.3661 | 0.0436 |
| TATDN1 | TatD DNase domain containing 1 | 1.9748 | 2.6993 | 1.3668 | 0.0432 |
| PGRMC1 | progesterone receptor membrane component 1 | 67.8598 | 92.7585 | 1.3669 | 0.0102 |
| LOC644589 | "similar to translocase of the inner mitochondrial membrane 14; DnaJ (Hsp40) homolog, subfamily C, member 19" | 7.0575 | 9.6474 | 1.3670 | 0.0160 |
| DNAJC19 | "similar to translocase of the inner mitochondrial membrane 14; DnaJ (Hsp40) homolog, subfamily C, member 19" | 7.0575 | 9.6474 | 1.3670 | 0.0160 |
| mRpS33 | mitochondrial ribosomal protein S33 | 9.3822 | 12.8441 | 1.3690 | 0.0068 |
| UBE2D1 | "ubiquitin-conjugating enzyme E2D 1 (UBC4/5 homolog, yeast)" | 5.1393 | 7.0421 | 1.3703 | 0.0414 |
| ACTR6 | ARP6 actin-related protein 6 homolog (yeast) | 8.2699 | 11.3455 | 1.3719 | 0.0439 |
| SNORA25 | "TATA box binding protein (TBP)-associated factor, RNA polymerase I, D, 41kDa; small nucleolar RNA, H/ACA box 32; small nucleolar RNA, H/ACA box 25" | 4.7451 | 6.5162 | 1.3733 | 0.0499 |
| TAF1D | "TATA box binding protein (TBP)-associated factor, RNA polymerase I, D, 41kDa; small nucleolar RNA, H/ACA box 32; small nucleolar RNA, H/ACA box 25" | 4.7451 | 6.5162 | 1.3733 | 0.0499 |
| SNORA32 | "TATA box binding protein (TBP)-associated factor, RNA polymerase I, D, 41kDa; small nucleolar RNA, H/ACA box 32; small nucleolar RNA, H/ACA box 25" | 4.7451 | 6.5162 | 1.3733 | 0.0499 |
| NDUFB6 | "NADH dehydrogenase (ubiquinone) 1 beta subcomplex, 6, 17kDa" | 20.2531 | 27.8342 | 1.3743 | 0.0215 |
| Rnf219 | ring finger protein 219 | 2.4505 | 3.3685 | 1.3746 | 0.0433 |
| fam188a | chromosome 10 open reading frame 97 | 6.8889 | 9.4725 | 1.3750 | 0.0142 |
| FAM92A2 | "family with sequence similarity 92, member A2; family with sequence similarity 92, member A1" | 2.9529 | 4.0605 | 1.3751 | 0.0369 |
| FAM92A1 | "family with sequence similarity 92, member A2; family with sequence similarity 92, member A1" | 2.9529 | 4.0605 | 1.3751 | 0.0369 |
| MRPL1 | mitochondrial ribosomal protein L1 | 7.3925 | 10.1724 | 1.3760 | 0.0090 |
| C4orf43 | chromosome 4 open reading frame 43 | 1.8688 | 2.5716 | 1.3761 | 0.0166 |
| HIGD1A | "similar to HIG1 domain family, member 1A; HIG1 hypoxia inducible domain family, member 1A; HIG1 hypoxia inducible domain family, member 1D" | 92.5548 | 127.4069 | 1.3766 | 0.0479 |
| HIGD1D | "similar to HIG1 domain family, member 1A; HIG1 hypoxia inducible domain family, member 1A; HIG1 hypoxia inducible domain family, member 1D" | 92.5548 | 127.4069 | 1.3766 | 0.0479 |
| LOC100130383 | "similar to HIG1 domain family, member 1A; HIG1 hypoxia inducible domain family, member 1A; HIG1 hypoxia inducible domain family, member 1D" | 92.5548 | 127.4069 | 1.3766 | 0.0479 |
| vbp1 | von Hippel-Lindau binding protein 1 | 20.0886 | 27.6534 | 1.3766 | 0.0315 |
| GSTO1 | glutathione S-transferase omega 1 | 27.4511 | 37.7916 | 1.3767 | 0.0038 |
| MAT2B | "methionine adenosyltransferase II, beta" | 10.9946 | 15.1370 | 1.3768 | 0.0167 |
| BRP44 | brain protein 44 | 18.1134 | 24.9669 | 1.3784 | 0.0155 |
| dnajb9 | "DnaJ (Hsp40) homolog, subfamily B, member 9" | 10.9062 | 15.0836 | 1.3830 | 0.0463 |
| Ptges3 | prostaglandin E synthase 3 (cytosolic) | 48.1904 | 66.6912 | 1.3839 | 0.0158 |
| CCNB1IP1 | cyclin B1 interacting protein 1 | 4.3061 | 5.9732 | 1.3871 | 0.0396 |
| MRPS35 | mitochondrial ribosomal protein S35 | 13.4211 | 18.6218 | 1.3875 | 0.0248 |
| cir1 | corepressor interacting with RBPJ | 8.3451 | 11.5811 | 1.3878 | 0.0364 |
| DSN1 | "DSN1, MIND kinetochore complex component, homolog (S. cerevisiae)" | 1.9331 | 2.6916 | 1.3924 | 0.0335 |
| KCNMB4 | "potassium large conductance calcium-activated channel, subfamily M, beta member 4" | 13.7836 | 19.1966 | 1.3927 | 0.0174 |
| mettl5 | methyltransferase like 5 | 5.1308 | 7.1494 | 1.3934 | 0.0056 |
| polB | "polymerase (DNA directed), beta" | 2.8000 | 3.9075 | 1.3955 | 0.0239 |
| CALM3 | "calmodulin 3 (phosphorylase kinase, delta); calmodulin 2 (phosphorylase kinase, delta); calmodulin 1 (phosphorylase kinase, delta)" | 117.7632 | 164.4545 | 1.3965 | 0.0239 |
| CALM2 | "calmodulin 3 (phosphorylase kinase, delta); calmodulin 2 (phosphorylase kinase, delta); calmodulin 1 (phosphorylase kinase, delta)" | 117.7632 | 164.4545 | 1.3965 | 0.0239 |
| CALM1 | "calmodulin 3 (phosphorylase kinase, delta); calmodulin 2 (phosphorylase kinase, delta); calmodulin 1 (phosphorylase kinase, delta)" | 117.7632 | 164.4545 | 1.3965 | 0.0239 |
| NIPSNAP3B | nipsnap homolog 3B (C. elegans) | 3.0899 | 4.3179 | 1.3974 | 0.0310 |
| RPL23AP82 | ribosomal protein L23a pseudogene 25; ribosomal protein L23a pseudogene 82 | 3.9491 | 5.5189 | 1.3975 | 0.0028 |
| RPL23AP25 | ribosomal protein L23a pseudogene 25; ribosomal protein L23a pseudogene 82 | 3.9491 | 5.5189 | 1.3975 | 0.0028 |
| C12orf76 | chromosome 12 open reading frame 76 | 9.9003 | 13.8400 | 1.3979 | 0.0300 |
| MTERFD1 | MTERF domain containing 1 | 4.4714 | 6.2509 | 1.3980 | 0.0167 |
| LOC729852 | hypothetical protein LOC729852 | 3.9589 | 5.5359 | 1.3983 | 0.0235 |
| LOC439953 | similar to TRIMCyp; peptidylprolyl isomerase A (cyclophilin A); peptidylprolyl isomerase A (cyclophilin A)-like 3 | 104.0579 | 145.8011 | 1.4012 | 0.0147 |
| ppiA | similar to TRIMCyp; peptidylprolyl isomerase A (cyclophilin A); peptidylprolyl isomerase A (cyclophilin A)-like 3 | 104.0579 | 145.8011 | 1.4012 | 0.0147 |
| PPIAL3 | similar to TRIMCyp; peptidylprolyl isomerase A (cyclophilin A); peptidylprolyl isomerase A (cyclophilin A)-like 3 | 104.0579 | 145.8011 | 1.4012 | 0.0147 |
| MKKS | McKusick-Kaufman syndrome | 16.5876 | 23.2656 | 1.4026 | 0.0249 |
| TMEM70 | transmembrane protein 70 | 7.3298 | 10.2856 | 1.4033 | 0.0216 |
| pts | 6-pyruvoyltetrahydropterin synthase | 5.7343 | 8.0619 | 1.4059 | 0.0160 |
| NSMCE2 | "non-SMC element 2, MMS21 homolog (S. cerevisiae)" | 2.3162 | 3.2571 | 1.4062 | 0.0287 |
| Zfand1 | "zinc finger, AN1-type domain 1" | 5.2923 | 7.4458 | 1.4069 | 0.0316 |
| Cdkl3 | cyclin-dependent kinase-like 3 | 1.1079 | 1.5595 | 1.4076 | 0.0329 |
| cisd2 | CDGSH iron sulfur domain 2 | 9.2833 | 13.0681 | 1.4077 | 0.0386 |
| PTPLA | "protein tyrosine phosphatase-like (proline instead of catalytic arginine), member A" | 1.3431 | 1.8925 | 1.4091 | 0.0497 |
| GGH | "gamma-glutamyl hydrolase (conjugase, folylpolygammaglutamyl hydrolase)" | 2.9818 | 4.2131 | 1.4130 | 0.0208 |
| LOC644101 | "similar to chromobox homolog 3; chromobox homolog 3 (HP1 gamma homolog, Drosophila)" | 13.1369 | 18.5679 | 1.4134 | 0.0235 |
| CBX3 | "similar to chromobox homolog 3; chromobox homolog 3 (HP1 gamma homolog, Drosophila)" | 13.1369 | 18.5679 | 1.4134 | 0.0235 |
| SPDYA | "protein phosphatase 1, catalytic subunit, beta isoform; speedy homolog A (Xenopus laevis)" | 26.6592 | 37.6903 | 1.4138 | 0.0301 |
| PPP1CB | "protein phosphatase 1, catalytic subunit, beta isoform; speedy homolog A (Xenopus laevis)" | 26.6592 | 37.6903 | 1.4138 | 0.0301 |
| cfdp1 | craniofacial development protein 1 | 8.1872 | 11.5855 | 1.4151 | 0.0337 |
| ccdc104 | coiled-coil domain containing 104 | 16.9421 | 24.0584 | 1.4200 | 0.0118 |
| CHORDC1 | cysteine and histidine-rich domain (CHORD)-containing 1; cysteine and histidine-rich domain (CHORD)-containing 1 pseudogene | 3.5675 | 5.0811 | 1.4243 | 0.0094 |
| LOC727896 | cysteine and histidine-rich domain (CHORD)-containing 1; cysteine and histidine-rich domain (CHORD)-containing 1 pseudogene | 3.5675 | 5.0811 | 1.4243 | 0.0094 |
| ube2b | ubiquitin-conjugating enzyme E2B (RAD6 homolog) | 19.0737 | 27.1937 | 1.4257 | 0.0160 |
| NDUFS4 | "NADH dehydrogenase (ubiquinone) Fe-S protein 4, 18kDa (NADH-coenzyme Q reductase)" | 17.1034 | 24.3978 | 1.4265 | 0.0259 |
| ECT2 | epithelial cell transforming sequence 2 oncogene | 1.5120 | 2.1578 | 1.4271 | 0.0447 |
| ARL6IP5 | ADP-ribosylation-like factor 6 interacting protein 5 | 52.4406 | 74.9334 | 1.4289 | 0.0137 |
| H2AFZ | "H2A histone family, member Z" | 22.3048 | 31.8777 | 1.4292 | 0.0010 |
| Ap1s2 | "adaptor-related protein complex 1, sigma 2 subunit pseudogene; adaptor-related protein complex 1, sigma 2 subunit" | 8.2103 | 11.7758 | 1.4343 | 0.0262 |
| LOC653653 | "adaptor-related protein complex 1, sigma 2 subunit pseudogene; adaptor-related protein complex 1, sigma 2 subunit" | 8.2103 | 11.7758 | 1.4343 | 0.0262 |
| STAG3L4 | stromal antigen 3-like 4 | 2.5337 | 3.6368 | 1.4354 | 0.0027 |
| Tomm22 | translocase of outer mitochondrial membrane 22 homolog (yeast) | 17.0106 | 24.4361 | 1.4365 | 0.0048 |
| BNIP3L | BCL2/adenovirus E1B 19kDa interacting protein 3-like | 28.8241 | 41.4724 | 1.4388 | 0.0404 |
| Upf3b | UPF3 regulator of nonsense transcripts homolog B (yeast) | 5.6284 | 8.0989 | 1.4389 | 0.0027 |
| AMD1 | adenosylmethionine decarboxylase 1 | 26.4339 | 38.0613 | 1.4399 | 0.0440 |
| smpdl3a | "sphingomyelin phosphodiesterase, acid-like 3A" | 2.8974 | 4.1774 | 1.4418 | 0.0311 |
| MAPKSP1 | MAPK scaffold protein 1 | 9.3612 | 13.5059 | 1.4427 | 0.0444 |
| Paip2 | poly(A) binding protein interacting protein 2 | 42.5660 | 61.4954 | 1.4447 | 0.0016 |
| FGFR1OP2 | FGFR1 oncogene partner 2 | 12.8738 | 18.6004 | 1.4448 | 0.0470 |
| USP45 | ubiquitin specific peptidase 45 | 1.2211 | 1.7645 | 1.4451 | 0.0420 |
| LOC375190 | chromosome 2 open reading frame 84; hypothetical protein LOC375190 | 1.9136 | 2.7656 | 1.4453 | 0.0127 |
| C2orf84 | chromosome 2 open reading frame 84; hypothetical protein LOC375190 | 1.9136 | 2.7656 | 1.4453 | 0.0127 |
| PIGP | "phosphatidylinositol glycan anchor biosynthesis, class P" | 1.9147 | 2.7698 | 1.4466 | 0.0032 |
| thoc7 | THO complex 7 homolog (Drosophila) | 16.0911 | 23.3263 | 1.4496 | 0.0247 |
| dut | deoxyuridine triphosphatase | 8.6941 | 12.6169 | 1.4512 | 0.0340 |
| ARMC1 | armadillo repeat containing 1 | 11.2898 | 16.3885 | 1.4516 | 0.0374 |
| Asnsd1 | asparagine synthetase domain containing 1 | 11.9077 | 17.2955 | 1.4525 | 0.0045 |
| THAP10 | THAP domain containing 10 | 3.1620 | 4.5945 | 1.4530 | 0.0105 |
| GLRX2 | glutaredoxin 2 | 6.4374 | 9.3573 | 1.4536 | 0.0165 |
| SNRNP27 | small nuclear ribonucleoprotein 27kDa (U4/U6.U5) | 8.6598 | 12.6073 | 1.4558 | 0.0478 |
| BLOC1S2 | "biogenesis of lysosomal organelles complex-1, subunit 2" | 19.0141 | 27.6886 | 1.4562 | 0.0014 |
| LOC727947 | similar to ubiquinol-cytochrome c reductase binding protein; ubiquinol-cytochrome c reductase binding protein pseudogene; ubiquinol-cytochrome c reductase binding protein | 15.4931 | 22.5819 | 1.4575 | 0.0234 |
| LOC442454 | similar to ubiquinol-cytochrome c reductase binding protein; ubiquinol-cytochrome c reductase binding protein pseudogene; ubiquinol-cytochrome c reductase binding protein | 15.4931 | 22.5819 | 1.4575 | 0.0234 |
| UQCRB | similar to ubiquinol-cytochrome c reductase binding protein; ubiquinol-cytochrome c reductase binding protein pseudogene; ubiquinol-cytochrome c reductase binding protein | 15.4931 | 22.5819 | 1.4575 | 0.0234 |
| NAP1L1 | nucleosome assembly protein 1-like 1 | 26.8676 | 39.1823 | 1.4584 | 0.0021 |
| ZNF302 | zinc finger protein 302 | 7.4659 | 10.8958 | 1.4594 | 0.0260 |
| C4orf27 | chromosome 4 open reading frame 27 | 11.9951 | 17.5098 | 1.4598 | 0.0278 |
| atp5l | "ATP synthase, H+ transporting, mitochondrial F0 complex, subunit G" | 14.1866 | 20.7133 | 1.4601 | 0.0144 |
| BTF3L4 | basic transcription factor 3-like 4; similar to hCG2008008 | 7.2300 | 10.5597 | 1.4605 | 0.0233 |
| LOC100131401 | basic transcription factor 3-like 4; similar to hCG2008008 | 7.2300 | 10.5597 | 1.4605 | 0.0233 |
| TMEM14A | transmembrane protein 14A | 44.0368 | 64.3190 | 1.4606 | 0.0032 |
| Ndufa4 | "NADH dehydrogenase (ubiquinone) 1 alpha subcomplex, 4, 9kDa" | 69.8566 | 102.0613 | 1.4610 | 0.0147 |
| BMI1 | BMI1 polycomb ring finger oncogene | 8.3221 | 12.1822 | 1.4638 | 0.0326 |
| PPA1 | pyrophosphatase (inorganic) 1 | 43.9257 | 64.3727 | 1.4655 | 0.0115 |
| c6orf211 | chromosome 6 open reading frame 211 | 6.0706 | 8.8976 | 1.4657 | 0.0180 |
| MED21 | mediator complex subunit 21 | 6.3349 | 9.3154 | 1.4705 | 0.0422 |
| NDUFAB1 | "NADH dehydrogenase (ubiquinone) 1, alpha/beta subcomplex, 1, 8kDa" | 22.6193 | 33.2708 | 1.4709 | 0.0003 |
| PCNP | PEST proteolytic signal containing nuclear protein | 39.5107 | 58.1978 | 1.4730 | 0.0390 |
| CCDC91 | coiled-coil domain containing 91 | 5.4896 | 8.0875 | 1.4732 | 0.0084 |
| BAG2 | BCL2-associated athanogene 2 | 1.6198 | 2.3866 | 1.4734 | 0.0306 |
| UQCRHL | ubiquinol-cytochrome c reductase hinge protein-like; ubiquinol-cytochrome c reductase hinge protein | 75.8500 | 112.1360 | 1.4784 | 0.0077 |
| Uqcrh | ubiquinol-cytochrome c reductase hinge protein-like; ubiquinol-cytochrome c reductase hinge protein | 75.8500 | 112.1360 | 1.4784 | 0.0077 |
| ripply2 | chromosome 6 open reading frame 59; ripply2 homolog (zebrafish) | 6.2534 | 9.2459 | 1.4785 | 0.0071 |
| C6orf59 | chromosome 6 open reading frame 59; ripply2 homolog (zebrafish) | 6.2534 | 9.2459 | 1.4785 | 0.0071 |
| C14orf2 | chromosome 14 open reading frame 2 | 17.1543 | 25.4054 | 1.4810 | 0.0103 |
| arl6ip1 | ADP-ribosylation factor-like 6 interacting protein 1 | 73.9868 | 109.6755 | 1.4824 | 0.0144 |
| ACYP1 | "acylphosphatase 1, erythrocyte (common) type" | 5.9461 | 8.8340 | 1.4857 | 0.0040 |
| PIGK | "phosphatidylinositol glycan anchor biosynthesis, class K" | 5.0884 | 7.5965 | 1.4929 | 0.0369 |
| COPS2 | COP9 constitutive photomorphogenic homolog subunit 2 (Arabidopsis) | 9.5241 | 14.2532 | 1.4966 | 0.0394 |
| sub1 | SUB1 homolog (S. cerevisiae) | 16.3449 | 24.4620 | 1.4966 | 0.0374 |
| c11orf74 | chromosome 11 open reading frame 74 | 4.8907 | 7.3378 | 1.5003 | 0.0037 |
| SH3GL3 | SH3-domain GRB2-like 3 | 14.8748 | 22.4269 | 1.5077 | 0.0372 |
| IMPA1 | inositol(myo)-1(or 4)-monophosphatase 1 | 8.1077 | 12.2320 | 1.5087 | 0.0447 |
| DSTN | destrin (actin depolymerizing factor) | 95.8469 | 144.6122 | 1.5088 | 0.0084 |
| PFDN4 | prefoldin subunit 4 | 9.0119 | 13.6626 | 1.5161 | 0.0072 |
| Ankrd46 | ankyrin repeat domain 46 | 11.3100 | 17.1730 | 1.5184 | 0.0403 |
| Yeats4 | YEATS domain containing 4 | 4.4571 | 6.7813 | 1.5215 | 0.0208 |
| AASDHPPT | aminoadipate-semialdehyde dehydrogenase-phosphopantetheinyl transferase | 12.0176 | 18.4405 | 1.5345 | 0.0392 |
| C3orf14 | chromosome 3 open reading frame 14 | 7.1947 | 11.0410 | 1.5346 | 0.0302 |
| GUSBP1 | "glucuronidase, beta-like 2; glucuronidase, beta pseudogene" | 1.1156 | 1.7125 | 1.5351 | 0.0138 |
| GUSBL2 | "glucuronidase, beta-like 2; glucuronidase, beta pseudogene" | 1.1156 | 1.7125 | 1.5351 | 0.0138 |
| Rpa3 | "replication protein A3, 14kDa" | 2.5117 | 3.8598 | 1.5367 | 0.0009 |
| SKP1 | S-phase kinase-associated protein 1 | 20.8248 | 32.0719 | 1.5401 | 0.0081 |
| C1orf151 | chromosome 1 open reading frame 151 | 3.1729 | 4.8871 | 1.5403 | 0.0058 |
| CCDC126 | coiled-coil domain containing 126 | 1.8664 | 2.8790 | 1.5425 | 0.0407 |
| C12orf62 | chromosome 12 open reading frame 62 | 11.3039 | 17.4419 | 1.5430 | 0.0283 |
| txndc9 | thioredoxin domain containing 9 | 3.5308 | 5.4571 | 1.5456 | 0.0343 |
| LRRC39 | leucine rich repeat containing 39 | 1.4252 | 2.2134 | 1.5530 | 0.0043 |
| Tceal7 | transcription elongation factor A (SII)-like 7 | 53.8349 | 83.7410 | 1.5555 | 0.0073 |
| SNX16 | sorting nexin 16 | 1.8465 | 2.8847 | 1.5622 | 0.0311 |
| COX6C | cytochrome c oxidase subunit VIc | 30.6973 | 48.2072 | 1.5704 | 0.0259 |
| C6orf115 | chromosome 6 open reading frame 115 | 6.8260 | 10.7973 | 1.5818 | 0.0041 |
| HINT1 | histidine triad nucleotide binding protein 1 | 55.0249 | 87.0891 | 1.5827 | 0.0013 |
| Tmem55a | transmembrane protein 55A | 8.6923 | 13.7606 | 1.5831 | 0.0091 |
| Mad2l1 | MAD2 mitotic arrest deficient-like 1 (yeast) | 1.8193 | 2.8884 | 1.5876 | 0.0311 |
| CISD1 | CDGSH iron sulfur domain 1 | 11.2123 | 17.8173 | 1.5891 | 0.0174 |
| GOLGA8A | "golgi autoantigen, golgin subfamily a, 8B; golgi autoantigen, golgin subfamily a, 8A" | 5.3398 | 8.5141 | 1.5945 | 0.0345 |
| GOLGA8B | "golgi autoantigen, golgin subfamily a, 8B; golgi autoantigen, golgin subfamily a, 8A" | 5.3398 | 8.5141 | 1.5945 | 0.0345 |
| EMB | embigin homolog (mouse) | 1.0999 | 1.7689 | 1.6082 | 0.0469 |
| Cox7b | cytochrome c oxidase subunit VIIb | 20.1009 | 32.3836 | 1.6111 | 0.0096 |
| sec61g | Sec61 gamma subunit | 7.4511 | 12.1191 | 1.6265 | 0.0319 |
| rbm11 | RNA binding motif protein 11 | 2.5681 | 4.1902 | 1.6316 | 0.0190 |
| POLR3G | polymerase (RNA) III (DNA directed) polypeptide G (32kD) | 1.2128 | 1.9803 | 1.6329 | 0.0384 |
| CCDC34 | coiled-coil domain containing 34 | 2.2126 | 3.6615 | 1.6549 | 0.0043 |
| TMSL2 | "thymosin-like 2 (pseudogene); thymosin-like 1 (pseudogene); thymosin beta 4, X-linked" | 275.7028 | 460.1926 | 1.6692 | 0.0245 |
| TMSL1 | "thymosin-like 2 (pseudogene); thymosin-like 1 (pseudogene); thymosin beta 4, X-linked" | 275.7028 | 460.1926 | 1.6692 | 0.0245 |
| TMSB4X | "thymosin-like 2 (pseudogene); thymosin-like 1 (pseudogene); thymosin beta 4, X-linked" | 275.7028 | 460.1926 | 1.6692 | 0.0245 |
| DYNLT3 | "dynein, light chain, Tctex-type 3" | 19.7038 | 33.4056 | 1.6954 | 0.0367 |
| Tctex1d1 | Tctex1 domain containing 1 | 1.0619 | 1.8061 | 1.7008 | 0.0058 |
| SVIP | small VCP/p97-interacting protein | 8.7777 | 14.9348 | 1.7014 | 0.0014 |
| Hist4h4 | "histone cluster 1, H4l; histone cluster 1, H4k; histone cluster 4, H4; histone cluster 1, H4h; histone cluster 1, H4j; histone cluster 1, H4i; histone cluster 1, H4d; histone cluster 1, H4c; histone cluster 1, H4f; histone cluster 1, H4e; histone cluster | 1.0957 | 1.8968 | 1.7311 | 0.0128 |
| Hist1h4j | "histone cluster 1, H4l; histone cluster 1, H4k; histone cluster 4, H4; histone cluster 1, H4h; histone cluster 1, H4j; histone cluster 1, H4i; histone cluster 1, H4d; histone cluster 1, H4c; histone cluster 1, H4f; histone cluster 1, H4e; histone cluster | 1.0957 | 1.8968 | 1.7311 | 0.0128 |
| Hist1h4b | "histone cluster 1, H4l; histone cluster 1, H4k; histone cluster 4, H4; histone cluster 1, H4h; histone cluster 1, H4j; histone cluster 1, H4i; histone cluster 1, H4d; histone cluster 1, H4c; histone cluster 1, H4f; histone cluster 1, H4e; histone cluster | 1.0957 | 1.8968 | 1.7311 | 0.0128 |
| HIST2H4B | "histone cluster 1, H4l; histone cluster 1, H4k; histone cluster 4, H4; histone cluster 1, H4h; histone cluster 1, H4j; histone cluster 1, H4i; histone cluster 1, H4d; histone cluster 1, H4c; histone cluster 1, H4f; histone cluster 1, H4e; histone cluster | 1.0957 | 1.8968 | 1.7311 | 0.0128 |
| Hist1h4a | "histone cluster 1, H4l; histone cluster 1, H4k; histone cluster 4, H4; histone cluster 1, H4h; histone cluster 1, H4j; histone cluster 1, H4i; histone cluster 1, H4d; histone cluster 1, H4c; histone cluster 1, H4f; histone cluster 1, H4e; histone cluster | 1.0957 | 1.8968 | 1.7311 | 0.0128 |
| HIST2H4A | "histone cluster 1, H4l; histone cluster 1, H4k; histone cluster 4, H4; histone cluster 1, H4h; histone cluster 1, H4j; histone cluster 1, H4i; histone cluster 1, H4d; histone cluster 1, H4c; histone cluster 1, H4f; histone cluster 1, H4e; histone cluster | 1.0957 | 1.8968 | 1.7311 | 0.0128 |
| Hist1h4i | "histone cluster 1, H4l; histone cluster 1, H4k; histone cluster 4, H4; histone cluster 1, H4h; histone cluster 1, H4j; histone cluster 1, H4i; histone cluster 1, H4d; histone cluster 1, H4c; histone cluster 1, H4f; histone cluster 1, H4e; histone cluster | 1.0957 | 1.8968 | 1.7311 | 0.0128 |
| Hist1h4h | "histone cluster 1, H4l; histone cluster 1, H4k; histone cluster 4, H4; histone cluster 1, H4h; histone cluster 1, H4j; histone cluster 1, H4i; histone cluster 1, H4d; histone cluster 1, H4c; histone cluster 1, H4f; histone cluster 1, H4e; histone cluster | 1.0957 | 1.8968 | 1.7311 | 0.0128 |
| Hist1h4f | "histone cluster 1, H4l; histone cluster 1, H4k; histone cluster 4, H4; histone cluster 1, H4h; histone cluster 1, H4j; histone cluster 1, H4i; histone cluster 1, H4d; histone cluster 1, H4c; histone cluster 1, H4f; histone cluster 1, H4e; histone cluster | 1.0957 | 1.8968 | 1.7311 | 0.0128 |
| Hist1h4c | "histone cluster 1, H4l; histone cluster 1, H4k; histone cluster 4, H4; histone cluster 1, H4h; histone cluster 1, H4j; histone cluster 1, H4i; histone cluster 1, H4d; histone cluster 1, H4c; histone cluster 1, H4f; histone cluster 1, H4e; histone cluster | 1.0957 | 1.8968 | 1.7311 | 0.0128 |
| hist1h4l | "histone cluster 1, H4l; histone cluster 1, H4k; histone cluster 4, H4; histone cluster 1, H4h; histone cluster 1, H4j; histone cluster 1, H4i; histone cluster 1, H4d; histone cluster 1, H4c; histone cluster 1, H4f; histone cluster 1, H4e; histone cluster | 1.0957 | 1.8968 | 1.7311 | 0.0128 |
| HIST1H4E | "histone cluster 1, H4l; histone cluster 1, H4k; histone cluster 4, H4; histone cluster 1, H4h; histone cluster 1, H4j; histone cluster 1, H4i; histone cluster 1, H4d; histone cluster 1, H4c; histone cluster 1, H4f; histone cluster 1, H4e; histone cluster | 1.0957 | 1.8968 | 1.7311 | 0.0128 |
| Hist1h4k | "histone cluster 1, H4l; histone cluster 1, H4k; histone cluster 4, H4; histone cluster 1, H4h; histone cluster 1, H4j; histone cluster 1, H4i; histone cluster 1, H4d; histone cluster 1, H4c; histone cluster 1, H4f; histone cluster 1, H4e; histone cluster | 1.0957 | 1.8968 | 1.7311 | 0.0128 |
| Hist1h4d | "histone cluster 1, H4l; histone cluster 1, H4k; histone cluster 4, H4; histone cluster 1, H4h; histone cluster 1, H4j; histone cluster 1, H4i; histone cluster 1, H4d; histone cluster 1, H4c; histone cluster 1, H4f; histone cluster 1, H4e; histone cluster | 1.0957 | 1.8968 | 1.7311 | 0.0128 |
| dnajc12 | "DnaJ (Hsp40) homolog, subfamily C, member 12" | 6.5600 | 11.4277 | 1.7420 | 0.0031 |
| C13orf27 | chromosome 13 open reading frame 27 | 2.2629 | 3.9721 | 1.7553 | 0.0014 |
| FAM133A | "family with sequence similarity 133, member A" | 1.5817 | 2.7784 | 1.7566 | 0.0274 |
| EEF1E1 | eukaryotic translation elongation factor 1 epsilon 1 | 2.1839 | 3.8576 | 1.7663 | 0.0234 |
| NECAB1 | N-terminal EF-hand calcium binding protein 1 | 25.7199 | 45.6002 | 1.7730 | 0.0411 |
| Gca | "grancalcin, EF-hand calcium binding protein" | 6.1870 | 11.0068 | 1.7790 | 0.0421 |
| Scoc | short coiled-coil protein | 35.9567 | 64.3714 | 1.7902 | 0.0199 |
| Kctd4 | potassium channel tetramerisation domain containing 4 | 3.1576 | 5.6729 | 1.7966 | 0.0378 |
| IFT80 | intraflagellar transport 80 homolog (Chlamydomonas) | 3.7537 | 6.9170 | 1.8427 | 0.0210 |
| LOC439953 | similar to TRIMCyp; peptidylprolyl isomerase A (cyclophilin A); peptidylprolyl isomerase A (cyclophilin A)-like 3 | 3.4614 | 6.6154 | 1.9112 | 0.0270 |
| ppiA | similar to TRIMCyp; peptidylprolyl isomerase A (cyclophilin A); peptidylprolyl isomerase A (cyclophilin A)-like 3 | 3.4614 | 6.6154 | 1.9112 | 0.0270 |
| PPIAL3 | similar to TRIMCyp; peptidylprolyl isomerase A (cyclophilin A); peptidylprolyl isomerase A (cyclophilin A)-like 3 | 3.4614 | 6.6154 | 1.9112 | 0.0270 |
| LOC728554 | similar to THO complex 3; THO complex 3 | 1.0830 | 2.1223 | 1.9597 | 0.0390 |
| THOC3 | similar to THO complex 3; THO complex 3 | 1.0830 | 2.1223 | 1.9597 | 0.0390 |
| SOSTDC1 | sclerostin domain containing 1 | 1.4245 | 2.8289 | 1.9859 | 0.0033 |
| RNU6-2 | "RNA, U6 small nuclear 2; RNA, U6 small nuclear 1" | 1.7841 | 3.6298 | 2.0345 | 0.0500 |
| RNU6-1 | "RNA, U6 small nuclear 2; RNA, U6 small nuclear 1" | 1.7841 | 3.6298 | 2.0345 | 0.0500 |
| RNU6-2 | "RNA, U6 small nuclear 2; RNA, U6 small nuclear 1" | 1.4456 | 3.0918 | 2.1388 | 0.0358 |
| RNU6-1 | "RNA, U6 small nuclear 2; RNA, U6 small nuclear 1" | 1.4456 | 3.0918 | 2.1388 | 0.0358 |
| GREM1 | "gremlin 1, cysteine knot superfamily, homolog (Xenopus laevis)" | 2.7284 | 6.9845 | 2.5599 | 0.0059 |
| RNU6-2 | "RNA, U6 small nuclear 2; RNA, U6 small nuclear 1" | 1.5332 | 4.0299 | 2.6284 | 0.0070 |
| RNU6-1 | "RNA, U6 small nuclear 2; RNA, U6 small nuclear 1" | 1.5332 | 4.0299 | 2.6284 | 0.0070 |
| **Gene symbol** | **Gene Name** | **adult** | **old** | **ratio** | **ttest** |
| LOC100132153 | similar to tubulin T beta15 | 10.4454 | 2.5743 | 0.2465 | 0.0315 |
| WNT7B | "wingless-type MMTV integration site family, member 7B" | 2.5660 | 0.6690 | 0.2607 | 0.0188 |
| PCDHGA12 | "protocadherin gamma subfamily C, 3; protocadherin gamma subfamily C, 5; protocadherin gamma subfamily C, 4; protocadherin gamma subfamily A, 12" | 1.0359 | 0.3321 | 0.3206 | 0.0003 |
| PCDHGC5 | "protocadherin gamma subfamily C, 3; protocadherin gamma subfamily C, 5; protocadherin gamma subfamily C, 4; protocadherin gamma subfamily A, 12" | 1.0359 | 0.3321 | 0.3206 | 0.0003 |
| PCDHGC3 | "protocadherin gamma subfamily C, 3; protocadherin gamma subfamily C, 5; protocadherin gamma subfamily C, 4; protocadherin gamma subfamily A, 12" | 1.0359 | 0.3321 | 0.3206 | 0.0003 |
| PCDHGC4 | "protocadherin gamma subfamily C, 3; protocadherin gamma subfamily C, 5; protocadherin gamma subfamily C, 4; protocadherin gamma subfamily A, 12" | 1.0359 | 0.3321 | 0.3206 | 0.0003 |
| SLC6A11 | "solute carrier family 6 (neurotransmitter transporter, GABA), member 11" | 7.8033 | 3.0209 | 0.3871 | 0.0423 |
| SLC29A4 | "solute carrier family 29 (nucleoside transporters), member 4; similar to solute carrier family 29 (nucleoside transporters), member 4" | 12.4424 | 4.8762 | 0.3919 | 0.0233 |
| LOC100132308 | "solute carrier family 29 (nucleoside transporters), member 4; similar to solute carrier family 29 (nucleoside transporters), member 4" | 12.4424 | 4.8762 | 0.3919 | 0.0233 |
| LOC402509 | "solute carrier family 29 (nucleoside transporters), member 4; similar to solute carrier family 29 (nucleoside transporters), member 4" | 12.4424 | 4.8762 | 0.3919 | 0.0233 |
| RNU6-2 | "RNA, U6 small nuclear 2; RNA, U6 small nuclear 1" | 2.2388 | 0.8852 | 0.3954 | 0.0145 |
| RNU6-1 | "RNA, U6 small nuclear 2; RNA, U6 small nuclear 1" | 2.2388 | 0.8852 | 0.3954 | 0.0145 |
| P2RY1 | "purinergic receptor P2Y, G-protein coupled, 1" | 1.4092 | 0.5625 | 0.3991 | 0.0274 |
| NCAN | neurocan | 38.4210 | 15.4501 | 0.4021 | 0.0045 |
| SHISA6 | FLJ45455 protein | 1.1456 | 0.4684 | 0.4089 | 0.0025 |
| Sorcs2 | sortilin-related VPS10 domain containing receptor 2 | 8.4566 | 3.5354 | 0.4181 | 0.0005 |
| rhpn2 | "rhophilin, Rho GTPase binding protein 2; similar to rhophilin, Rho GTPase binding protein 2" | 2.0030 | 0.8628 | 0.4307 | 0.0471 |
| RHPN2P1 | "rhophilin, Rho GTPase binding protein 2; similar to rhophilin, Rho GTPase binding protein 2" | 2.0030 | 0.8628 | 0.4307 | 0.0471 |
| Fat1 | FAT tumor suppressor homolog 1 (Drosophila) | 3.7743 | 1.6331 | 0.4327 | 0.0194 |
| rgma | "RGM domain family, member A" | 17.9974 | 7.9215 | 0.4401 | 0.0089 |
| Plcxd1 | "phosphatidylinositol-specific phospholipase C, X domain containing 1" | 6.9548 | 3.1017 | 0.4460 | 0.0472 |
| ephb1 | EPH receptor B1 | 4.4325 | 1.9781 | 0.4463 | 0.0066 |
| GRIN2D | "glutamate receptor, ionotropic, N-methyl D-aspartate 2D" | 2.1841 | 1.0176 | 0.4659 | 0.0101 |
| Prdm16 | PR domain containing 16 | 2.0348 | 0.9714 | 0.4774 | 0.0423 |
| sdc3 | syndecan 3 | 49.1572 | 23.7673 | 0.4835 | 0.0128 |
| RADIL | Ras association and DIL domains | 1.4385 | 0.7022 | 0.4881 | 0.0440 |
| Ttyh3 | tweety homolog 3 (Drosophila) | 33.0055 | 16.1513 | 0.4894 | 0.0162 |
| LRP5 | low density lipoprotein receptor-related protein 5 | 2.3584 | 1.1611 | 0.4923 | 0.0316 |
| mir126 | microRNA 126 | 1.3909 | 0.6855 | 0.4929 | 0.0276 |
| DCHS1 | dachsous 1 (Drosophila) | 2.5035 | 1.2351 | 0.4934 | 0.0055 |
| ZNF710 | zinc finger protein 710 | 5.9894 | 2.9916 | 0.4995 | 0.0046 |
| SEPN1 | "selenoprotein N, 1" | 14.7191 | 7.3847 | 0.5017 | 0.0078 |
| Ptchd2 | patched domain containing 2 | 2.0549 | 1.0310 | 0.5018 | 0.0032 |
| BAI1 | brain-specific angiogenesis inhibitor 1 | 31.3867 | 15.9550 | 0.5083 | 0.0122 |
| BCL9L | B-cell CLL/lymphoma 9-like | 2.9379 | 1.5002 | 0.5106 | 0.0279 |
| MAPK4 | mitogen-activated protein kinase 4 | 15.2305 | 7.7987 | 0.5120 | 0.0081 |
| LRRC8A | "leucine rich repeat containing 8 family, member A" | 29.6998 | 15.2697 | 0.5141 | 0.0279 |
| Sprn | shadow of prion protein homolog (zebrafish) | 15.7481 | 8.1602 | 0.5182 | 0.0406 |
| KIAA0195 | KIAA0195 | 17.1415 | 8.9091 | 0.5197 | 0.0144 |
| APC2 | adenomatosis polyposis coli 2 | 29.8771 | 15.5930 | 0.5219 | 0.0312 |
| Fam83h | "family with sequence similarity 83, member H" | 2.8450 | 1.4903 | 0.5238 | 0.0252 |
| lrp1 | low density lipoprotein-related protein 1 (alpha-2-macroglobulin receptor) | 23.0753 | 12.1032 | 0.5245 | 0.0062 |
| Slc12a4 | "solute carrier family 12 (potassium/chloride transporters), member 4" | 3.1270 | 1.6413 | 0.5249 | 0.0490 |
| Mir324 | microRNA 324 | 2.4629 | 1.2945 | 0.5256 | 0.0350 |
| DAG1 | dystroglycan 1 (dystrophin-associated glycoprotein 1) | 11.9971 | 6.3648 | 0.5305 | 0.0112 |
| TNRC18B | trinucleotide repeat containing 18B | 9.8015 | 5.2050 | 0.5310 | 0.0484 |
| ECE1 | endothelin converting enzyme 1 | 8.3630 | 4.4485 | 0.5319 | 0.0115 |
| wfs1 | Wolfram syndrome 1 (wolframin) | 18.1636 | 9.7767 | 0.5383 | 0.0185 |
| RN5S17 | "RNA, 5S ribosomal 9; RNA, 5S ribosomal 13; RNA, 5S ribosomal 12; RNA, 5S ribosomal 11; RNA, 5S ribosomal 10; RNA, 5S ribosomal 17; RNA, 5S ribosomal 16; RNA, 5S ribosomal 15; RNA, 5S ribosomal 14; RNA, 5S ribosomal 1; RNA, 5S ribosomal 2; RNA, 5S ribosom | 3.4166 | 1.8427 | 0.5393 | 0.0303 |
| RN5S16 | "RNA, 5S ribosomal 9; RNA, 5S ribosomal 13; RNA, 5S ribosomal 12; RNA, 5S ribosomal 11; RNA, 5S ribosomal 10; RNA, 5S ribosomal 17; RNA, 5S ribosomal 16; RNA, 5S ribosomal 15; RNA, 5S ribosomal 14; RNA, 5S ribosomal 1; RNA, 5S ribosomal 2; RNA, 5S ribosom | 3.4166 | 1.8427 | 0.5393 | 0.0303 |
| RN5S6 | "RNA, 5S ribosomal 9; RNA, 5S ribosomal 13; RNA, 5S ribosomal 12; RNA, 5S ribosomal 11; RNA, 5S ribosomal 10; RNA, 5S ribosomal 17; RNA, 5S ribosomal 16; RNA, 5S ribosomal 15; RNA, 5S ribosomal 14; RNA, 5S ribosomal 1; RNA, 5S ribosomal 2; RNA, 5S ribosom | 3.4166 | 1.8427 | 0.5393 | 0.0303 |
| RN5S7 | "RNA, 5S ribosomal 9; RNA, 5S ribosomal 13; RNA, 5S ribosomal 12; RNA, 5S ribosomal 11; RNA, 5S ribosomal 10; RNA, 5S ribosomal 17; RNA, 5S ribosomal 16; RNA, 5S ribosomal 15; RNA, 5S ribosomal 14; RNA, 5S ribosomal 1; RNA, 5S ribosomal 2; RNA, 5S ribosom | 3.4166 | 1.8427 | 0.5393 | 0.0303 |
| RN5S9 | "RNA, 5S ribosomal 9; RNA, 5S ribosomal 13; RNA, 5S ribosomal 12; RNA, 5S ribosomal 11; RNA, 5S ribosomal 10; RNA, 5S ribosomal 17; RNA, 5S ribosomal 16; RNA, 5S ribosomal 15; RNA, 5S ribosomal 14; RNA, 5S ribosomal 1; RNA, 5S ribosomal 2; RNA, 5S ribosom | 3.4166 | 1.8427 | 0.5393 | 0.0303 |
| RN5S3 | "RNA, 5S ribosomal 9; RNA, 5S ribosomal 13; RNA, 5S ribosomal 12; RNA, 5S ribosomal 11; RNA, 5S ribosomal 10; RNA, 5S ribosomal 17; RNA, 5S ribosomal 16; RNA, 5S ribosomal 15; RNA, 5S ribosomal 14; RNA, 5S ribosomal 1; RNA, 5S ribosomal 2; RNA, 5S ribosom | 3.4166 | 1.8427 | 0.5393 | 0.0303 |
| RN5S4 | "RNA, 5S ribosomal 9; RNA, 5S ribosomal 13; RNA, 5S ribosomal 12; RNA, 5S ribosomal 11; RNA, 5S ribosomal 10; RNA, 5S ribosomal 17; RNA, 5S ribosomal 16; RNA, 5S ribosomal 15; RNA, 5S ribosomal 14; RNA, 5S ribosomal 1; RNA, 5S ribosomal 2; RNA, 5S ribosom | 3.4166 | 1.8427 | 0.5393 | 0.0303 |
| RN5S1 | "RNA, 5S ribosomal 9; RNA, 5S ribosomal 13; RNA, 5S ribosomal 12; RNA, 5S ribosomal 11; RNA, 5S ribosomal 10; RNA, 5S ribosomal 17; RNA, 5S ribosomal 16; RNA, 5S ribosomal 15; RNA, 5S ribosomal 14; RNA, 5S ribosomal 1; RNA, 5S ribosomal 2; RNA, 5S ribosom | 3.4166 | 1.8427 | 0.5393 | 0.0303 |
| RN5S15 | "RNA, 5S ribosomal 9; RNA, 5S ribosomal 13; RNA, 5S ribosomal 12; RNA, 5S ribosomal 11; RNA, 5S ribosomal 10; RNA, 5S ribosomal 17; RNA, 5S ribosomal 16; RNA, 5S ribosomal 15; RNA, 5S ribosomal 14; RNA, 5S ribosomal 1; RNA, 5S ribosomal 2; RNA, 5S ribosom | 3.4166 | 1.8427 | 0.5393 | 0.0303 |
| RN5S8 | "RNA, 5S ribosomal 9; RNA, 5S ribosomal 13; RNA, 5S ribosomal 12; RNA, 5S ribosomal 11; RNA, 5S ribosomal 10; RNA, 5S ribosomal 17; RNA, 5S ribosomal 16; RNA, 5S ribosomal 15; RNA, 5S ribosomal 14; RNA, 5S ribosomal 1; RNA, 5S ribosomal 2; RNA, 5S ribosom | 3.4166 | 1.8427 | 0.5393 | 0.0303 |
| RN5S5 | "RNA, 5S ribosomal 9; RNA, 5S ribosomal 13; RNA, 5S ribosomal 12; RNA, 5S ribosomal 11; RNA, 5S ribosomal 10; RNA, 5S ribosomal 17; RNA, 5S ribosomal 16; RNA, 5S ribosomal 15; RNA, 5S ribosomal 14; RNA, 5S ribosomal 1; RNA, 5S ribosomal 2; RNA, 5S ribosom | 3.4166 | 1.8427 | 0.5393 | 0.0303 |
| RN5S13 | "RNA, 5S ribosomal 9; RNA, 5S ribosomal 13; RNA, 5S ribosomal 12; RNA, 5S ribosomal 11; RNA, 5S ribosomal 10; RNA, 5S ribosomal 17; RNA, 5S ribosomal 16; RNA, 5S ribosomal 15; RNA, 5S ribosomal 14; RNA, 5S ribosomal 1; RNA, 5S ribosomal 2; RNA, 5S ribosom | 3.4166 | 1.8427 | 0.5393 | 0.0303 |
| RN5S12 | "RNA, 5S ribosomal 9; RNA, 5S ribosomal 13; RNA, 5S ribosomal 12; RNA, 5S ribosomal 11; RNA, 5S ribosomal 10; RNA, 5S ribosomal 17; RNA, 5S ribosomal 16; RNA, 5S ribosomal 15; RNA, 5S ribosomal 14; RNA, 5S ribosomal 1; RNA, 5S ribosomal 2; RNA, 5S ribosom | 3.4166 | 1.8427 | 0.5393 | 0.0303 |
| RN5S11 | "RNA, 5S ribosomal 9; RNA, 5S ribosomal 13; RNA, 5S ribosomal 12; RNA, 5S ribosomal 11; RNA, 5S ribosomal 10; RNA, 5S ribosomal 17; RNA, 5S ribosomal 16; RNA, 5S ribosomal 15; RNA, 5S ribosomal 14; RNA, 5S ribosomal 1; RNA, 5S ribosomal 2; RNA, 5S ribosom | 3.4166 | 1.8427 | 0.5393 | 0.0303 |
| RN5S2 | "RNA, 5S ribosomal 9; RNA, 5S ribosomal 13; RNA, 5S ribosomal 12; RNA, 5S ribosomal 11; RNA, 5S ribosomal 10; RNA, 5S ribosomal 17; RNA, 5S ribosomal 16; RNA, 5S ribosomal 15; RNA, 5S ribosomal 14; RNA, 5S ribosomal 1; RNA, 5S ribosomal 2; RNA, 5S ribosom | 3.4166 | 1.8427 | 0.5393 | 0.0303 |
| RN5S10 | "RNA, 5S ribosomal 9; RNA, 5S ribosomal 13; RNA, 5S ribosomal 12; RNA, 5S ribosomal 11; RNA, 5S ribosomal 10; RNA, 5S ribosomal 17; RNA, 5S ribosomal 16; RNA, 5S ribosomal 15; RNA, 5S ribosomal 14; RNA, 5S ribosomal 1; RNA, 5S ribosomal 2; RNA, 5S ribosom | 3.4166 | 1.8427 | 0.5393 | 0.0303 |
| RN5S14 | "RNA, 5S ribosomal 9; RNA, 5S ribosomal 13; RNA, 5S ribosomal 12; RNA, 5S ribosomal 11; RNA, 5S ribosomal 10; RNA, 5S ribosomal 17; RNA, 5S ribosomal 16; RNA, 5S ribosomal 15; RNA, 5S ribosomal 14; RNA, 5S ribosomal 1; RNA, 5S ribosomal 2; RNA, 5S ribosom | 3.4166 | 1.8427 | 0.5393 | 0.0303 |
| caskin2 | CASK interacting protein 2 | 8.3258 | 4.5285 | 0.5439 | 0.0119 |
| Cacng4 | "calcium channel, voltage-dependent, gamma subunit 4" | 5.8744 | 3.1963 | 0.5441 | 0.0322 |
| Gpr75 | G protein-coupled receptor 75 | 3.7713 | 2.0579 | 0.5457 | 0.0106 |
| tp53i11 | tumor protein p53 inducible protein 11 | 5.3294 | 2.9133 | 0.5466 | 0.0157 |
| Gnl3l | guanine nucleotide binding protein-like 3 (nucleolar)-like | 2.0849 | 1.1472 | 0.5502 | 0.0130 |
| CCDC85C | coiled-coil domain containing 85C | 4.3245 | 2.3940 | 0.5536 | 0.0401 |
| Pskh1 | protein serine kinase H1 | 4.1123 | 2.2917 | 0.5573 | 0.0500 |
| mn1 | meningioma (disrupted in balanced translocation) 1 | 2.8469 | 1.5917 | 0.5591 | 0.0035 |
| FAM38A | "family with sequence similarity 38, member A" | 2.6074 | 1.4603 | 0.5601 | 0.0385 |
| TMEM132A | transmembrane protein 132A | 29.8723 | 16.7974 | 0.5623 | 0.0266 |
| WDR86 | WD repeat domain 86 | 1.9269 | 1.0890 | 0.5652 | 0.0454 |
| Tpcn1 | two pore segment channel 1 | 10.8609 | 6.1412 | 0.5654 | 0.0165 |
| GATA2 | GATA binding protein 2 | 1.0725 | 0.6092 | 0.5680 | 0.0469 |
| SLC7A5 | "solute carrier family 7 (cationic amino acid transporter, y+ system), member 5" | 23.4529 | 13.3761 | 0.5703 | 0.0115 |
| TMEM104 | transmembrane protein 104 | 3.6113 | 2.0602 | 0.5705 | 0.0163 |
| ZNF628 | zinc finger protein 628 | 1.7187 | 0.9826 | 0.5717 | 0.0185 |
| Per1 | period homolog 1 (Drosophila) | 10.2926 | 5.8948 | 0.5727 | 0.0210 |
| JMJD8 | jumonji domain containing 8 | 4.6810 | 2.6853 | 0.5737 | 0.0231 |
| plxnb2 | plexin B2 | 9.6950 | 5.5651 | 0.5740 | 0.0252 |
| RAVER1 | "ribonucleoprotein, PTB-binding 1" | 1.9665 | 1.1367 | 0.5781 | 0.0068 |
| SHROOM3 | shroom family member 3 | 1.5664 | 0.9065 | 0.5787 | 0.0170 |
| notch1 | "Notch homolog 1, translocation-associated (Drosophila)" | 5.1118 | 2.9665 | 0.5803 | 0.0188 |
| CHPF | chondroitin polymerizing factor | 21.1567 | 12.2989 | 0.5813 | 0.0043 |
| Tie1 | tyrosine kinase with immunoglobulin-like and EGF-like domains 1 | 2.8841 | 1.6920 | 0.5867 | 0.0011 |
| BOC | Boc homolog (mouse) | 1.0452 | 0.6146 | 0.5881 | 0.0252 |
| C2orf85 | chromosome 2 open reading frame 85 | 5.9640 | 3.5077 | 0.5882 | 0.0245 |
| kiaa0664 | KIAA0664 | 13.6272 | 8.0371 | 0.5898 | 0.0254 |
| sufU | suppressor of fused homolog (Drosophila) | 2.6063 | 1.5378 | 0.5900 | 0.0039 |
| AGRN | agrin | 17.7114 | 10.4845 | 0.5920 | 0.0137 |
| Hcfc1 | host cell factor C1 (VP16-accessory protein) | 10.1541 | 6.0227 | 0.5931 | 0.0411 |
| rgs12 | regulator of G-protein signaling 12 | 7.7069 | 4.5719 | 0.5932 | 0.0220 |
| PLEC | "similar to Plectin 1 (PLTN) (PCN) (Hemidesmosomal protein 1) (HD1); plectin 1, intermediate filament binding protein 500kDa" | 21.2382 | 12.5996 | 0.5933 | 0.0269 |
| LOC652460 | "similar to Plectin 1 (PLTN) (PCN) (Hemidesmosomal protein 1) (HD1); plectin 1, intermediate filament binding protein 500kDa" | 21.2382 | 12.5996 | 0.5933 | 0.0269 |
| KCNN3 | "potassium intermediate/small conductance calcium-activated channel, subfamily N, member 3" | 1.6254 | 0.9663 | 0.5945 | 0.0441 |
| MIDN | midnolin | 10.0463 | 5.9727 | 0.5945 | 0.0241 |
| LRFN3 | leucine rich repeat and fibronectin type III domain containing 3 | 6.7473 | 4.0120 | 0.5946 | 0.0440 |
| Grik5 | "glutamate receptor, ionotropic, kainate 5" | 15.3848 | 9.1520 | 0.5949 | 0.0389 |
| mmp15 | matrix metallopeptidase 15 (membrane-inserted) | 3.4520 | 2.0562 | 0.5956 | 0.0144 |
| Klhl26 | kelch-like 26 (Drosophila) | 10.4873 | 6.2515 | 0.5961 | 0.0066 |
| ST5 | suppression of tumorigenicity 5 | 2.0260 | 1.2086 | 0.5966 | 0.0170 |
| TNKS1BP1 | "tankyrase 1 binding protein 1, 182kDa" | 8.6296 | 5.1509 | 0.5969 | 0.0048 |
| TLE3 | "transducin-like enhancer of split 3 (E(sp1) homolog, Drosophila)" | 2.6320 | 1.5723 | 0.5974 | 0.0201 |
| FKBP9 | "FK506 binding protein 9, 63 kDa" | 10.4672 | 6.2542 | 0.5975 | 0.0136 |
| Foxk1 | forkhead box K1 | 8.0211 | 4.7941 | 0.5977 | 0.0468 |
| SPATA2L | spermatogenesis associated 2-like | 7.6727 | 4.5873 | 0.5979 | 0.0315 |
| GAS2L1 | growth arrest-specific 2 like 1 | 20.0175 | 12.0066 | 0.5998 | 0.0474 |
| Ell | elongation factor RNA polymerase II | 1.9753 | 1.1858 | 0.6003 | 0.0235 |
| WDR81 | WD repeat domain 81 | 2.8008 | 1.6864 | 0.6021 | 0.0059 |
| SLC24A3 | "solute carrier family 24 (sodium/potassium/calcium exchanger), member 3" | 5.0675 | 3.0570 | 0.6033 | 0.0071 |
| tmem198 | transmembrane protein 198 | 8.7396 | 5.2979 | 0.6062 | 0.0239 |
| SLC27A1 | "solute carrier family 27 (fatty acid transporter), member 1" | 7.5732 | 4.5938 | 0.6066 | 0.0417 |
| ATP10A | "ATPase, class V, type 10A" | 1.9660 | 1.1938 | 0.6072 | 0.0126 |
| TMEM164 | transmembrane protein 164 | 3.0995 | 1.8883 | 0.6092 | 0.0431 |
| NPRL3 | chromosome 16 open reading frame 35 | 7.1767 | 4.3850 | 0.6110 | 0.0032 |
| SALL2 | sal-like 2 (Drosophila) | 8.5473 | 5.2303 | 0.6119 | 0.0145 |
| Abcd1 | "ATP-binding cassette, sub-family D (ALD), member 1" | 1.3126 | 0.8038 | 0.6124 | 0.0165 |
| NEURL1B | neuralized homolog 1B (Drosophila) | 4.5540 | 2.7895 | 0.6125 | 0.0393 |
| SLC6A1 | "solute carrier family 6 (neurotransmitter transporter, GABA), member 1" | 81.2451 | 49.8214 | 0.6132 | 0.0053 |
| clpB | ClpB caseinolytic peptidase B homolog (E. coli) | 5.5668 | 3.4167 | 0.6138 | 0.0431 |
| znf687 | zinc finger protein 687 | 4.8208 | 2.9644 | 0.6149 | 0.0020 |
| NLGN3 | neuroligin 3 | 27.4399 | 16.9052 | 0.6161 | 0.0023 |
| Med16 | mediator complex subunit 16 | 14.1849 | 8.7639 | 0.6178 | 0.0227 |
| MDGA1 | MAM domain containing glycosylphosphatidylinositol anchor 1 | 1.8634 | 1.1531 | 0.6188 | 0.0159 |
| AP1B1 | "adaptor-related protein complex 1, beta 1 subunit" | 21.0360 | 13.0316 | 0.6195 | 0.0136 |
| Atg2a | ATG2 autophagy related 2 homolog A (S. cerevisiae) | 6.0947 | 3.7759 | 0.6195 | 0.0155 |
| LRP3 | low density lipoprotein receptor-related protein 3 | 39.1814 | 24.2818 | 0.6197 | 0.0379 |
| Col5a1 | "collagen, type V, alpha 1" | 1.0236 | 0.6346 | 0.6200 | 0.0257 |
| sdk2 | sidekick homolog 2 (chicken) | 1.4975 | 0.9299 | 0.6210 | 0.0436 |
| sema3f | "sema domain, immunoglobulin domain (Ig), short basic domain, secreted, (semaphorin) 3F" | 1.1414 | 0.7104 | 0.6224 | 0.0407 |
| Zc3h4 | zinc finger CCCH-type containing 4 | 6.2162 | 3.8701 | 0.6226 | 0.0090 |
| RXRA | "retinoid X receptor, alpha" | 7.1832 | 4.4792 | 0.6236 | 0.0377 |
| CDH4 | "cadherin 4, type 1, R-cadherin (retinal)" | 6.5634 | 4.0928 | 0.6236 | 0.0274 |
| CSDC2 | "cold shock domain containing C2, RNA binding" | 11.6521 | 7.2747 | 0.6243 | 0.0246 |
| bcr | breakpoint cluster region | 12.6294 | 7.9011 | 0.6256 | 0.0198 |
| ATN1 | atrophin 1 | 82.8166 | 51.8512 | 0.6261 | 0.0465 |
| Sel1l3 | KIAA0746 protein | 3.5851 | 2.2502 | 0.6277 | 0.0339 |
| vps37b | vacuolar protein sorting 37 homolog B (S. cerevisiae) | 4.2051 | 2.6423 | 0.6284 | 0.0171 |
| WIZ | widely interspaced zinc finger motifs | 8.0758 | 5.0746 | 0.6284 | 0.0262 |
| BEGAIN | brain-enriched guanylate kinase-associated homolog (rat) | 8.7029 | 5.4768 | 0.6293 | 0.0233 |
| CCDC9 | coiled-coil domain containing 9 | 6.6880 | 4.2100 | 0.6295 | 0.0405 |
| WWC1 | WW and C2 domain containing 1 | 9.0736 | 5.7350 | 0.6321 | 0.0432 |
| ZNF70 | zinc finger protein 70 | 1.1551 | 0.7314 | 0.6332 | 0.0473 |
| Fam5b | "family with sequence similarity 5, member B" | 19.8083 | 12.5476 | 0.6334 | 0.0021 |
| LRRC56 | leucine rich repeat containing 56 | 1.7978 | 1.1394 | 0.6338 | 0.0309 |
| sox1 | SRY (sex determining region Y)-box 1 | 1.4607 | 0.9268 | 0.6345 | 0.0191 |
| speG | SPEG complex locus | 5.3228 | 3.3804 | 0.6351 | 0.0324 |
| GRAMD4 | "TSC22 domain family, member 3; GRAM domain containing 4" | 11.9012 | 7.5684 | 0.6359 | 0.0164 |
| TSC22D3 | "TSC22 domain family, member 3; GRAM domain containing 4" | 11.9012 | 7.5684 | 0.6359 | 0.0164 |
| Znf516 | zinc finger protein 516 | 1.7427 | 1.1094 | 0.6366 | 0.0429 |
| slc1a4 | "solute carrier family 1 (glutamate/neutral amino acid transporter), member 4" | 14.4544 | 9.2020 | 0.6366 | 0.0276 |
| MAVS | mitochondrial antiviral signaling protein | 4.2655 | 2.7159 | 0.6367 | 0.0338 |
| Nlgn2 | neuroligin 2 | 39.0978 | 24.9020 | 0.6369 | 0.0376 |
| PCNXL3 | pecanex-like 3 (Drosophila) | 5.4681 | 3.4946 | 0.6391 | 0.0039 |
| ZNF282 | zinc finger protein 282 | 6.2300 | 3.9819 | 0.6392 | 0.0106 |
| CREB3L1 | cAMP responsive element binding protein 3-like 1 | 2.7431 | 1.7539 | 0.6394 | 0.0171 |
| Srcap | Snf2-related CREBBP activator protein | 9.3053 | 5.9587 | 0.6404 | 0.0343 |
| CREBBP | CREB binding protein | 6.6923 | 4.2879 | 0.6407 | 0.0372 |
| ZBTB45 | zinc finger and BTB domain containing 45 | 7.2703 | 4.6644 | 0.6416 | 0.0484 |
| Nacc1 | "nucleus accumbens associated 1, BEN and BTB (POZ) domain containing" | 21.5839 | 13.8551 | 0.6419 | 0.0275 |
| CABIN1 | calcineurin binding protein 1 | 10.7648 | 6.9103 | 0.6419 | 0.0395 |
| C15orf59 | chromosome 15 open reading frame 59 | 41.8528 | 26.8811 | 0.6423 | 0.0418 |
| KIAA0649 | KIAA0649 | 11.3902 | 7.3183 | 0.6425 | 0.0180 |
| Hyal1 | hyaluronoglucosaminidase 1 | 1.1700 | 0.7527 | 0.6433 | 0.0113 |
| ptch1 | patched homolog 1 (Drosophila) | 2.0593 | 1.3250 | 0.6434 | 0.0313 |
| EHD1 | EH-domain containing 1 | 5.9121 | 3.8057 | 0.6437 | 0.0138 |
| SH3GL1 | SH3-domain GRB2-like 1 | 16.4607 | 10.5986 | 0.6439 | 0.0266 |
| Meis3 | Meis homeobox 3; Meis homeobox 3 pseudogene 2 | 20.1277 | 12.9769 | 0.6447 | 0.0396 |
| MEIS3P2 | Meis homeobox 3; Meis homeobox 3 pseudogene 2 | 20.1277 | 12.9769 | 0.6447 | 0.0396 |
| Pc | pyruvate carboxylase | 17.7166 | 11.4315 | 0.6452 | 0.0361 |
| MYH9 | "myosin, heavy chain 9, non-muscle" | 23.9754 | 15.4855 | 0.6459 | 0.0217 |
| SCAP | SREBF chaperone | 15.2742 | 9.8832 | 0.6471 | 0.0053 |
| b4galt2 | "UDP-Gal:betaGlcNAc beta 1,4- galactosyltransferase, polypeptide 2" | 23.5979 | 15.2731 | 0.6472 | 0.0075 |
| PRKD2 | protein kinase D2 | 2.2135 | 1.4346 | 0.6481 | 0.0345 |
| CRB2 | crumbs homolog 2 (Drosophila) | 4.1039 | 2.6610 | 0.6484 | 0.0464 |
| mfi2 | antigen p97 (melanoma associated) identified by monoclonal antibodies 133.2 and 96.5 | 1.5728 | 1.0212 | 0.6493 | 0.0380 |
| RGS9 | regulator of G-protein signaling 9 | 1.1230 | 0.7294 | 0.6495 | 0.0494 |
| ARHGEF10L | Rho guanine nucleotide exchange factor (GEF) 10-like | 5.7591 | 3.7423 | 0.6498 | 0.0265 |
| SETD1A | SET domain containing 1A | 5.0950 | 3.3194 | 0.6515 | 0.0358 |
| Crat | carnitine acetyltransferase | 15.7618 | 10.2966 | 0.6533 | 0.0207 |
| Ptbp1 | polypyrimidine tract binding protein 1 | 5.9044 | 3.8591 | 0.6536 | 0.0457 |
| Alg12 | "asparagine-linked glycosylation 12, alpha-1,6-mannosyltransferase homolog (S. cerevisiae)" | 4.9756 | 3.2574 | 0.6547 | 0.0082 |
| Fjx1 | four jointed box 1 (Drosophila) | 7.8300 | 5.1273 | 0.6548 | 0.0349 |
| NLRX1 | NLR family member X1 | 1.9300 | 1.2646 | 0.6552 | 0.0203 |
| MINK1 | misshapen-like kinase 1 (zebrafish) | 33.8796 | 22.2199 | 0.6558 | 0.0436 |
| Mier2 | "mesoderm induction early response 1, family member 2" | 5.2335 | 3.4334 | 0.6560 | 0.0288 |
| ZNF777 | zinc finger protein 777 | 6.8514 | 4.4994 | 0.6567 | 0.0382 |
| Plxnd1 | plexin D1 | 8.3438 | 5.4822 | 0.6570 | 0.0080 |
| GALNT2 | UDP-N-acetyl-alpha-D-galactosamine:polypeptide N-acetylgalactosaminyltransferase 2 (GalNAc-T2) | 6.5009 | 4.2751 | 0.6576 | 0.0074 |
| Ldlr | low density lipoprotein receptor | 3.0424 | 2.0028 | 0.6583 | 0.0274 |
| Mapk7 | mitogen-activated protein kinase 7 | 2.1983 | 1.4484 | 0.6589 | 0.0205 |
| ZNF362 | zinc finger protein 362 | 6.1694 | 4.0677 | 0.6593 | 0.0396 |
| Ehd4 | EH-domain containing 4 | 1.4515 | 0.9577 | 0.6598 | 0.0387 |
| actn4 | "actinin, alpha 4" | 40.2279 | 26.5645 | 0.6604 | 0.0101 |
| cyp46a1 | "cytochrome P450, family 46, subfamily A, polypeptide 1" | 13.6795 | 9.0371 | 0.6606 | 0.0270 |
| ABR | active BCR-related gene | 56.4049 | 37.2734 | 0.6608 | 0.0498 |
| ARHGAP33 | sorting nexin 26 | 18.4753 | 12.2420 | 0.6626 | 0.0465 |
| ephb2 | EPH receptor B2 | 2.7745 | 1.8390 | 0.6628 | 0.0293 |
| ncln | nicalin homolog (zebrafish) | 11.8861 | 7.8812 | 0.6631 | 0.0417 |
| SNX8 | sorting nexin 8 | 6.4651 | 4.2904 | 0.6636 | 0.0434 |
| PFKL | "phosphofructokinase, liver" | 24.3672 | 16.1929 | 0.6645 | 0.0055 |
| zbtb46 | zinc finger and BTB domain containing 46 | 2.9935 | 1.9909 | 0.6651 | 0.0405 |
| GYS1 | glycogen synthase 1 (muscle) | 7.2942 | 4.8544 | 0.6655 | 0.0088 |
| LPPR2 | lipid phosphate phosphatase-related protein type 2 | 36.7002 | 24.4556 | 0.6664 | 0.0368 |
| GABBR1 | "gamma-aminobutyric acid (GABA) B receptor, 1" | 76.1743 | 50.8778 | 0.6679 | 0.0143 |
| TMEM8B | transmembrane protein 8B | 10.0936 | 6.7505 | 0.6688 | 0.0223 |
| plk3 | polo-like kinase 3 (Drosophila) | 1.8518 | 1.2386 | 0.6688 | 0.0168 |
| calhm2 | calcium homeostasis modulator 2 | 2.9240 | 1.9598 | 0.6703 | 0.0313 |
| akt1 | v-akt murine thymoma viral oncogene homolog 1 | 7.0057 | 4.6971 | 0.6705 | 0.0273 |
| STRN4 | "striatin, calmodulin binding protein 4" | 12.4723 | 8.3639 | 0.6706 | 0.0377 |
| TRPM4 | "transient receptor potential cation channel, subfamily M, member 4" | 2.0168 | 1.3536 | 0.6712 | 0.0476 |
| TRAF7 | TNF receptor-associated factor 7 | 8.8980 | 5.9746 | 0.6714 | 0.0451 |
| ANO8 | anoctamin 8 | 10.8354 | 7.2820 | 0.6721 | 0.0158 |
| Arvcf | armadillo repeat gene deletes in velocardiofacial syndrome | 6.5503 | 4.4027 | 0.6721 | 0.0252 |
| Rasip1 | Ras interacting protein 1 | 2.4521 | 1.6483 | 0.6722 | 0.0466 |
| STAG3L1 | stromal antigen 3-like 2; stromal antigen 3-like 1 | 2.0466 | 1.3770 | 0.6728 | 0.0365 |
| STAG3L2 | stromal antigen 3-like 2; stromal antigen 3-like 1 | 2.0466 | 1.3770 | 0.6728 | 0.0365 |
| Tab1 | mitogen-activated protein kinase kinase kinase 7 interacting protein 1 | 9.9706 | 6.7174 | 0.6737 | 0.0058 |
| Prkca | "protein kinase C, alpha" | 18.9411 | 12.7663 | 0.6740 | 0.0137 |
| PTK7 | PTK7 protein tyrosine kinase 7 | 1.1310 | 0.7638 | 0.6753 | 0.0124 |
| RNF40 | ring finger protein 40 | 12.8496 | 8.6799 | 0.6755 | 0.0172 |
| ltbp3 | latent transforming growth factor beta binding protein 3 | 13.6522 | 9.2256 | 0.6758 | 0.0374 |
| pygb | "phosphorylase, glycogen; brain" | 52.3591 | 35.3825 | 0.6758 | 0.0191 |
| SCARB1 | "scavenger receptor class B, member 1" | 7.0364 | 4.7550 | 0.6758 | 0.0113 |
| Mta1 | metastasis associated 1 | 7.6375 | 5.1615 | 0.6758 | 0.0257 |
| Slc43a2 | "solute carrier family 43, member 2" | 7.2461 | 4.8996 | 0.6762 | 0.0489 |
| xrcc3 | X-ray repair complementing defective repair in Chinese hamster cells 3 | 1.4821 | 1.0029 | 0.6767 | 0.0469 |
| ZC3H3 | zinc finger CCCH-type containing 3 | 5.0437 | 3.4140 | 0.6769 | 0.0184 |
| EPHB3 | EPH receptor B3 | 3.0338 | 2.0538 | 0.6770 | 0.0092 |
| PAK4 | p21 protein (Cdc42/Rac)-activated kinase 4 | 3.6253 | 2.4547 | 0.6771 | 0.0161 |
| GREB1 | GREB1 protein | 2.7210 | 1.8452 | 0.6781 | 0.0136 |
| Zmiz1 | "zinc finger, MIZ-type containing 1" | 13.3092 | 9.0503 | 0.6800 | 0.0490 |
| Ptpn23 | "protein tyrosine phosphatase, non-receptor type 23" | 13.2609 | 9.0245 | 0.6805 | 0.0299 |
| Ogdh | oxoglutarate (alpha-ketoglutarate) dehydrogenase (lipoamide) | 31.7670 | 21.6295 | 0.6809 | 0.0158 |
| Stk32c | serine/threonine kinase 32C | 18.9868 | 12.9388 | 0.6815 | 0.0340 |
| Tcf7l2 | "transcription factor 7-like 2 (T-cell specific, HMG-box)" | 4.7032 | 3.2059 | 0.6816 | 0.0256 |
| FOXRED2 | FAD-dependent oxidoreductase domain containing 2 | 10.6099 | 7.2334 | 0.6818 | 0.0186 |
| TMEM184B | transmembrane protein 184B | 18.9301 | 12.9089 | 0.6819 | 0.0043 |
| SIDT2 | "SID1 transmembrane family, member 2" | 6.4622 | 4.4076 | 0.6821 | 0.0045 |
| MOBKL2A | "MOB1, Mps One Binder kinase activator-like 2A (yeast)" | 4.6356 | 3.1620 | 0.6821 | 0.0326 |
| LETM1 | leucine zipper-EF-hand containing transmembrane protein 1 | 7.4379 | 5.0776 | 0.6827 | 0.0073 |
| VPS18 | vacuolar protein sorting 18 homolog (S. cerevisiae) | 9.6564 | 6.5968 | 0.6832 | 0.0472 |
| OSBPL5 | oxysterol binding protein-like 5 | 4.0906 | 2.7957 | 0.6834 | 0.0363 |
| ercc2 | "excision repair cross-complementing rodent repair deficiency, complementation group 2" | 4.6399 | 3.1718 | 0.6836 | 0.0213 |
| nfix | nuclear factor I/X (CCAAT-binding transcription factor) | 55.7459 | 38.1076 | 0.6836 | 0.0427 |
| por | P450 (cytochrome) oxidoreductase | 7.7847 | 5.3227 | 0.6837 | 0.0121 |
| UPF1 | UPF1 regulator of nonsense transcripts homolog (yeast) | 10.8764 | 7.4384 | 0.6839 | 0.0254 |
| dtx1 | deltex homolog 1 (Drosophila) | 11.6639 | 7.9898 | 0.6850 | 0.0241 |
| PXDN | peroxidasin homolog (Drosophila) | 1.3189 | 0.9050 | 0.6862 | 0.0271 |
| Xylt2 | xylosyltransferase II | 4.0958 | 2.8122 | 0.6866 | 0.0194 |
| Pofut1 | protein O-fucosyltransferase 1 | 2.7134 | 1.8643 | 0.6871 | 0.0194 |
| GTPBP1 | GTP binding protein 1 | 5.5833 | 3.8378 | 0.6874 | 0.0078 |
| MYH14 | "myosin, heavy chain 14" | 7.8348 | 5.3856 | 0.6874 | 0.0307 |
| tcf3 | transcription factor 3 (E2A immunoglobulin enhancer binding factors E12/E47) | 3.7732 | 2.5937 | 0.6874 | 0.0395 |
| SEMA4G | "sema domain, immunoglobulin domain (Ig), transmembrane domain (TM) and short cytoplasmic domain, (semaphorin) 4G" | 1.9902 | 1.3683 | 0.6875 | 0.0194 |
| EHMT1 | euchromatic histone-lysine N-methyltransferase 1 | 2.5419 | 1.7477 | 0.6876 | 0.0165 |
| C17orf103 | chromosome 17 open reading frame 103 | 2.5344 | 1.7433 | 0.6879 | 0.0085 |
| PNPLA7 | patatin-like phospholipase domain containing 7 | 4.1373 | 2.8460 | 0.6879 | 0.0397 |
| NXPH3 | neurexophilin 3 | 7.1953 | 4.9518 | 0.6882 | 0.0406 |
| khsrp | KH-type splicing regulatory protein | 28.9518 | 19.9442 | 0.6889 | 0.0367 |
| PDZRN3 | PDZ domain containing ring finger 3 | 5.8300 | 4.0172 | 0.6891 | 0.0087 |
| Trim65 | tripartite motif-containing 65 | 5.1876 | 3.5754 | 0.6892 | 0.0158 |
| tmem8a | transmembrane protein 8A | 6.5148 | 4.4912 | 0.6894 | 0.0470 |
| ARID1A | AT rich interactive domain 1A (SWI-like) | 8.6099 | 5.9365 | 0.6895 | 0.0255 |
| c19orf6 | chromosome 19 open reading frame 6 | 34.2619 | 23.6315 | 0.6897 | 0.0487 |
| LOC152845 | pleiomorphic adenoma gene-like 2; similar to pleiomorphic adenoma gene-like 2 | 1.9426 | 1.3414 | 0.6905 | 0.0016 |
| PLAGL2 | pleiomorphic adenoma gene-like 2; similar to pleiomorphic adenoma gene-like 2 | 1.9426 | 1.3414 | 0.6905 | 0.0016 |
| BRPF1 | "bromodomain and PHD finger containing, 1" | 3.3802 | 2.3369 | 0.6914 | 0.0105 |
| MEX3B | mex-3 homolog B (C. elegans) | 1.6832 | 1.1645 | 0.6919 | 0.0099 |
| fgd1 | "FYVE, RhoGEF and PH domain containing 1" | 5.5612 | 3.8492 | 0.6922 | 0.0465 |
| YBX2 | Y box binding protein 2 | 1.0651 | 0.7374 | 0.6923 | 0.0442 |
| Dot1l | "DOT1-like, histone H3 methyltransferase (S. cerevisiae)" | 2.1378 | 1.4811 | 0.6928 | 0.0493 |
| TBC1D14 | "TBC1 domain family, member 14" | 5.8234 | 4.0369 | 0.6932 | 0.0477 |
| SLC41A1 | "solute carrier family 41, member 1" | 8.9258 | 6.1884 | 0.6933 | 0.0125 |
| RFX1 | "regulatory factor X, 1 (influences HLA class II expression)" | 2.4034 | 1.6664 | 0.6934 | 0.0331 |
| BCL9 | B-cell CLL/lymphoma 9 | 5.0097 | 3.4748 | 0.6936 | 0.0173 |
| BAHD1 | bromo adjacent homology domain containing 1 | 5.8269 | 4.0521 | 0.6954 | 0.0231 |
| CCDC120 | coiled-coil domain containing 120 | 1.7626 | 1.2261 | 0.6956 | 0.0458 |
| TOM1L2 | target of myb1-like 2 (chicken) | 36.9437 | 25.7301 | 0.6965 | 0.0196 |
| GMEB2 | glucocorticoid modulatory element binding protein 2 | 4.0831 | 2.8440 | 0.6965 | 0.0271 |
| KDM4B | lysine (K)-specific demethylase 4B | 4.6069 | 3.2093 | 0.6966 | 0.0094 |
| FBXW8 | F-box and WD repeat domain containing 8 | 1.6581 | 1.1557 | 0.6970 | 0.0093 |
| TECPR2 | tectonin beta-propeller repeat containing 2 | 10.6621 | 7.4375 | 0.6976 | 0.0114 |
| PXN | paxillin | 2.8819 | 2.0106 | 0.6977 | 0.0314 |
| DNAJB5 | "DnaJ (Hsp40) homolog, subfamily B, member 5" | 5.5846 | 3.8991 | 0.6982 | 0.0055 |
| C17orf70 | chromosome 17 open reading frame 70 | 4.7205 | 3.2985 | 0.6988 | 0.0235 |
| IFT140 | intraflagellar transport 140 homolog (Chlamydomonas) | 3.8782 | 2.7114 | 0.6992 | 0.0318 |
| Ctdp1 | "CTD (carboxy-terminal domain, RNA polymerase II, polypeptide A) phosphatase, subunit 1" | 3.1220 | 2.1881 | 0.7009 | 0.0279 |
| MDC1 | mediator of DNA-damage checkpoint 1 | 3.6798 | 2.5804 | 0.7012 | 0.0104 |
| PAQR7 | progestin and adipoQ receptor family member VII | 3.4291 | 2.4078 | 0.7022 | 0.0109 |
| CYFIP1 | cytoplasmic FMR1 interacting protein 1 | 4.8136 | 3.3813 | 0.7025 | 0.0081 |
| Axin2 | axin 2 | 3.4034 | 2.3909 | 0.7025 | 0.0336 |
| Eif4g1 | "eukaryotic translation initiation factor 4 gamma, 1" | 24.2225 | 17.0172 | 0.7025 | 0.0135 |
| pprc1 | "peroxisome proliferator-activated receptor gamma, coactivator-related 1" | 3.9640 | 2.7855 | 0.7027 | 0.0445 |
| GTF3C1 | "general transcription factor IIIC, polypeptide 1, alpha 220kDa" | 8.3648 | 5.8799 | 0.7029 | 0.0446 |
| PHF12 | PHD finger protein 12 | 3.1612 | 2.2222 | 0.7030 | 0.0007 |
| SMAD3 | SMAD family member 3 | 6.7728 | 4.7634 | 0.7033 | 0.0278 |
| Acox3 | "acyl-Coenzyme A oxidase 3, pristanoyl" | 3.1833 | 2.2394 | 0.7035 | 0.0006 |
| TMEM201 | transmembrane protein 201 | 3.4430 | 2.4221 | 0.7035 | 0.0225 |
| Tbcd | tubulin folding cofactor D | 7.7094 | 5.4278 | 0.7041 | 0.0360 |
| Sirpa | signal-regulatory protein alpha | 109.7370 | 77.2938 | 0.7044 | 0.0383 |
| FBXW5 | F-box and WD repeat domain containing 5 | 34.5686 | 24.3556 | 0.7046 | 0.0415 |
| TSPAN14 | tetraspanin 14 | 7.5003 | 5.2896 | 0.7053 | 0.0099 |
| ZNF408 | zinc finger protein 408 | 2.4910 | 1.7585 | 0.7060 | 0.0172 |
| RASSF7 | Ras association (RalGDS/AF-6) domain family (N-terminal) member 7 | 3.6305 | 2.5644 | 0.7064 | 0.0386 |
| RBMS2P1 | "RNA binding motif, single stranded interacting protein 2; RNA binding motif, single stranded interacting protein 2 pseudogene" | 1.9836 | 1.4014 | 0.7065 | 0.0491 |
| rbms2 | "RNA binding motif, single stranded interacting protein 2; RNA binding motif, single stranded interacting protein 2 pseudogene" | 1.9836 | 1.4014 | 0.7065 | 0.0491 |
| GTPBP5 | GTP binding protein 5 (putative) | 4.8850 | 3.4561 | 0.7075 | 0.0292 |
| kiaa0556 | KIAA0556 | 2.7289 | 1.9319 | 0.7080 | 0.0047 |
| MVD | mevalonate (diphospho) decarboxylase | 13.4266 | 9.5141 | 0.7086 | 0.0304 |
| Kifc3 | kinesin family member C3 | 6.7475 | 4.7816 | 0.7086 | 0.0248 |
| ZNF646 | zinc finger protein 646 | 2.3409 | 1.6595 | 0.7089 | 0.0310 |
| mtmr3 | myotubularin related protein 3 | 6.0156 | 4.2666 | 0.7093 | 0.0076 |
| sp2 | Sp2 transcription factor | 7.4173 | 5.2627 | 0.7095 | 0.0150 |
| p4hb | "prolyl 4-hydroxylase, beta polypeptide" | 17.4134 | 12.3594 | 0.7098 | 0.0040 |
| PDK2 | "pyruvate dehydrogenase kinase, isozyme 2" | 10.5770 | 7.5126 | 0.7103 | 0.0323 |
| fes | feline sarcoma oncogene | 2.0138 | 1.4316 | 0.7109 | 0.0381 |
| EP300 | E1A binding protein p300 | 7.7356 | 5.4992 | 0.7109 | 0.0257 |
| c19orf22 | chromosome 19 open reading frame 22 | 14.0561 | 9.9981 | 0.7113 | 0.0300 |
| NAV2 | neuron navigator 2 | 2.9725 | 2.1153 | 0.7116 | 0.0064 |
| ZNF764 | zinc finger protein 764 | 2.0308 | 1.4460 | 0.7120 | 0.0068 |
| traF2 | TNF receptor-associated factor 2 | 3.5728 | 2.5448 | 0.7123 | 0.0308 |
| MGAT4B | "mannosyl (alpha-1,3-)-glycoprotein beta-1,4-N-acetylglucosaminyltransferase, isozyme B" | 9.9313 | 7.0759 | 0.7125 | 0.0248 |
| Chmp1a | chromatin modifying protein 1A | 24.0315 | 17.1264 | 0.7127 | 0.0305 |
| FBRS | fibrosin | 8.3944 | 5.9938 | 0.7140 | 0.0300 |
| spen | "spen homolog, transcriptional regulator (Drosophila)" | 7.6682 | 5.4815 | 0.7148 | 0.0455 |
| ADRB1 | "adrenergic, beta-1-, receptor" | 6.1744 | 4.4151 | 0.7151 | 0.0278 |
| C14orf43 | chromosome 14 open reading frame 43 | 2.7837 | 1.9913 | 0.7153 | 0.0428 |
| trim8 | tripartite motif-containing 8 | 39.0551 | 27.9679 | 0.7161 | 0.0483 |
| GPR172A | G protein-coupled receptor 172A | 4.0416 | 2.8943 | 0.7161 | 0.0365 |
| PQLC2 | PQ loop repeat containing 2 | 5.0767 | 3.6364 | 0.7163 | 0.0085 |
| ZBTB48 | zinc finger and BTB domain containing 48 | 3.4105 | 2.4440 | 0.7166 | 0.0029 |
| Ndor1 | NADPH dependent diflavin oxidoreductase 1 | 6.3182 | 4.5281 | 0.7167 | 0.0482 |
| FAM57A | "family with sequence similarity 57, member A" | 2.8144 | 2.0183 | 0.7171 | 0.0450 |
| erF | Ets2 repressor factor | 11.4409 | 8.2080 | 0.7174 | 0.0411 |
| Pold1 | "polymerase (DNA directed), delta 1, catalytic subunit 125kDa" | 1.1904 | 0.8541 | 0.7175 | 0.0450 |
| NOL9 | nucleolar protein 9 | 3.6319 | 2.6083 | 0.7182 | 0.0189 |
| CSNK1D | "casein kinase 1, delta" | 12.3592 | 8.8811 | 0.7186 | 0.0422 |
| LMF1 | lipase maturation factor 1 | 3.1453 | 2.2615 | 0.7190 | 0.0166 |
| PLCG1 | "phospholipase C, gamma 1" | 15.7131 | 11.2990 | 0.7191 | 0.0113 |
| rnf166 | ring finger protein 166 | 3.2208 | 2.3166 | 0.7193 | 0.0426 |
| ARMC5 | armadillo repeat containing 5 | 3.4819 | 2.5067 | 0.7199 | 0.0418 |
| Fbxo46 | F-box protein 46 | 2.7126 | 1.9536 | 0.7202 | 0.0364 |
| ERC1 | ELKS/RAB6-interacting/CAST family member 1 | 3.6865 | 2.6571 | 0.7208 | 0.0488 |
| C20orf117 | chromosome 20 open reading frame 117 | 5.8702 | 4.2335 | 0.7212 | 0.0387 |
| MED12 | mediator complex subunit 12 | 4.1357 | 2.9871 | 0.7223 | 0.0058 |
| Cant1 | calcium activated nucleotidase 1 | 6.3520 | 4.5900 | 0.7226 | 0.0445 |
| Dcaf15 | chromosome 19 open reading frame 72 | 5.0849 | 3.6746 | 0.7226 | 0.0104 |
| wdr18 | WD repeat domain 18 | 10.5861 | 7.6517 | 0.7228 | 0.0187 |
| Hdac7 | histone deacetylase 7 | 3.4576 | 2.5007 | 0.7232 | 0.0154 |
| Sgta | "small glutamine-rich tetratricopeptide repeat (TPR)-containing, alpha" | 25.6054 | 18.5248 | 0.7235 | 0.0154 |
| Sf3a1 | "splicing factor 3a, subunit 1, 120kDa" | 15.9981 | 11.5789 | 0.7238 | 0.0332 |
| gcn1l1 | GCN1 general control of amino-acid synthesis 1-like 1 (yeast) | 6.1776 | 4.4746 | 0.7243 | 0.0270 |
| CLPTM1 | cleft lip and palate associated transmembrane protein 1 | 33.5949 | 24.3507 | 0.7248 | 0.0243 |
| rpap1 | RNA polymerase II associated protein 1 | 4.0509 | 2.9383 | 0.7254 | 0.0160 |
| Phf2 | PHD finger protein 2 | 9.1130 | 6.6197 | 0.7264 | 0.0281 |
| CD276 | CD276 molecule | 3.0379 | 2.2084 | 0.7269 | 0.0322 |
| Sema4a | "sema domain, immunoglobulin domain (Ig), transmembrane domain (TM) and short cytoplasmic domain, (semaphorin) 4A" | 5.6273 | 4.0908 | 0.7270 | 0.0432 |
| cad | "carbamoyl-phosphate synthetase 2, aspartate transcarbamylase, and dihydroorotase" | 3.1236 | 2.2738 | 0.7279 | 0.0271 |
| hectd3 | HECT domain containing 3 | 8.2178 | 5.9934 | 0.7293 | 0.0291 |
| MAML1 | mastermind-like 1 (Drosophila) | 2.7665 | 2.0178 | 0.7294 | 0.0068 |
| NCKAP5L | KIAA1602 | 4.2353 | 3.0959 | 0.7310 | 0.0345 |
| GPR137 | G protein-coupled receptor 137 | 13.7143 | 10.0259 | 0.7311 | 0.0413 |
| Ints5 | integrator complex subunit 5 | 4.5861 | 3.3622 | 0.7331 | 0.0311 |
| Fam160a2 | "family with sequence similarity 160, member A2" | 6.3684 | 4.6731 | 0.7338 | 0.0168 |
| CPNE6 | copine VI (neuronal) | 12.5595 | 9.2252 | 0.7345 | 0.0254 |
| gcc1 | GRIP and coiled-coil domain containing 1 | 2.4965 | 1.8350 | 0.7350 | 0.0002 |
| POFUT2 | protein O-fucosyltransferase 2 | 3.3400 | 2.4551 | 0.7351 | 0.0132 |
| Gramd1a | GRAM domain containing 1A | 7.6353 | 5.6141 | 0.7353 | 0.0452 |
| UNC45A | unc-45 homolog A (C. elegans) | 5.2850 | 3.8888 | 0.7358 | 0.0119 |
| WHSC2 | Wolf-Hirschhorn syndrome candidate 2 | 5.2137 | 3.8371 | 0.7360 | 0.0365 |
| Adora1 | adenosine A1 receptor | 23.5785 | 17.3529 | 0.7360 | 0.0401 |
| DVL3 | "dishevelled, dsh homolog 3 (Drosophila)" | 14.3087 | 10.5381 | 0.7365 | 0.0202 |
| SLC35C1 | "solute carrier family 35, member C1" | 3.2370 | 2.3841 | 0.7365 | 0.0490 |
| GNAO1 | "guanine nucleotide binding protein (G protein), alpha activating activity polypeptide O" | 52.0036 | 38.3213 | 0.7369 | 0.0153 |
| lpcat1 | lysophosphatidylcholine acyltransferase 1 | 8.2387 | 6.0733 | 0.7372 | 0.0219 |
| rab36 | "RAB36, member RAS oncogene family" | 4.6704 | 3.4434 | 0.7373 | 0.0489 |
| Inpp5d | "inositol polyphosphate-5-phosphatase, 145kDa" | 1.3028 | 0.9608 | 0.7375 | 0.0348 |
| ACO2 | "aconitase 2, mitochondrial" | 50.2094 | 37.0361 | 0.7376 | 0.0203 |
| ZNF319 | zinc finger protein 319 | 2.8998 | 2.1412 | 0.7384 | 0.0224 |
| SLITRK2 | "similar to CXorf2 protein; SLIT and NTRK-like family, member 2" | 7.0332 | 5.1945 | 0.7386 | 0.0272 |
| LOC100129095 | "similar to CXorf2 protein; SLIT and NTRK-like family, member 2" | 7.0332 | 5.1945 | 0.7386 | 0.0272 |
| AXIN1 | axin 1 | 4.0061 | 2.9601 | 0.7389 | 0.0351 |
| SLC25A23 | "solute carrier family 25 (mitochondrial carrier; phosphate carrier), member 23" | 89.9340 | 66.4531 | 0.7389 | 0.0270 |
| DOLK | dolichol kinase | 4.7724 | 3.5273 | 0.7391 | 0.0227 |
| C2orf18 | chromosome 2 open reading frame 18 | 7.4177 | 5.4826 | 0.7391 | 0.0022 |
| AGPAT3 | 1-acylglycerol-3-phosphate O-acyltransferase 3 | 14.6361 | 10.8232 | 0.7395 | 0.0417 |
| SNRNP200 | "similar to U5 snRNP-specific protein, 200 kDa; small nuclear ribonucleoprotein 200kDa (U5)" | 11.3146 | 8.3692 | 0.7397 | 0.0029 |
| LOC652147 | "similar to U5 snRNP-specific protein, 200 kDa; small nuclear ribonucleoprotein 200kDa (U5)" | 11.3146 | 8.3692 | 0.7397 | 0.0029 |
| heatr2 | HEAT repeat containing 2 | 1.6030 | 1.1858 | 0.7397 | 0.0151 |
| lmf2 | lipase maturation factor 2 | 13.5955 | 10.0586 | 0.7398 | 0.0388 |
| Dclk2 | doublecortin-like kinase 2 | 23.0555 | 17.0618 | 0.7400 | 0.0054 |
| LOC100133760 | "similar to Jumonji, AT rich interactive domain 1B (RBP2-like); lysine (K)-specific demethylase 5B" | 4.1368 | 3.0635 | 0.7405 | 0.0083 |
| KDM5B | "similar to Jumonji, AT rich interactive domain 1B (RBP2-like); lysine (K)-specific demethylase 5B" | 4.1368 | 3.0635 | 0.7405 | 0.0083 |
| Znfx1 | "zinc finger, NFX1-type containing 1" | 4.1211 | 3.0526 | 0.7407 | 0.0021 |
| ERGIC1 | endoplasmic reticulum-golgi intermediate compartment (ERGIC) 1 | 4.7017 | 3.4835 | 0.7409 | 0.0080 |
| Ptpn9 | "protein tyrosine phosphatase, non-receptor type 9" | 7.1816 | 5.3221 | 0.7411 | 0.0069 |
| GTF2IRD1 | GTF2I repeat domain containing 1 | 2.4445 | 1.8136 | 0.7419 | 0.0023 |
| USP2 | ubiquitin specific peptidase 2 | 7.4710 | 5.5433 | 0.7420 | 0.0279 |
| PCDHB15 | protocadherin beta 15 | 2.0040 | 1.4879 | 0.7425 | 0.0121 |
| SMG6 | "Smg-6 homolog, nonsense mediated mRNA decay factor (C. elegans)" | 4.8923 | 3.6333 | 0.7427 | 0.0315 |
| Dhx38 | DEAH (Asp-Glu-Ala-His) box polypeptide 38 | 8.1183 | 6.0349 | 0.7434 | 0.0247 |
| RAB1B | "RAB1B, member RAS oncogene family" | 26.9934 | 20.0741 | 0.7437 | 0.0301 |
| Add1 | adducin 1 (alpha) | 30.2131 | 22.5281 | 0.7456 | 0.0002 |
| ccdc97 | coiled-coil domain containing 97 | 5.8842 | 4.3917 | 0.7463 | 0.0145 |
| CTNND1 | "catenin (cadherin-associated protein), delta 1" | 12.9566 | 9.6726 | 0.7465 | 0.0025 |
| Stat3 | signal transducer and activator of transcription 3 (acute-phase response factor) | 8.1291 | 6.0734 | 0.7471 | 0.0077 |
| RIC8A | resistance to inhibitors of cholinesterase 8 homolog A (C. elegans) | 11.3085 | 8.4500 | 0.7472 | 0.0058 |
| pelp1 | "proline, glutamate and leucine rich protein 1" | 16.0526 | 12.0024 | 0.7477 | 0.0226 |
| BRF1 | "BRF1 homolog, subunit of RNA polymerase III transcription initiation factor IIIB (S. cerevisiae)" | 2.2645 | 1.6933 | 0.7478 | 0.0490 |
| AUTS2 | autism susceptibility candidate 2 | 3.9732 | 2.9718 | 0.7480 | 0.0158 |
| SLC16A2 | "solute carrier family 16, member 2 (monocarboxylic acid transporter 8)" | 5.8084 | 4.3459 | 0.7482 | 0.0286 |
| Nup214 | nucleoporin 214kDa | 2.8965 | 2.1705 | 0.7494 | 0.0010 |
| CTNND2 | "catenin (cadherin-associated protein), delta 2 (neural plakophilin-related arm-repeat protein)" | 55.4649 | 41.6140 | 0.7503 | 0.0247 |
| ANAPC2 | anaphase promoting complex subunit 2 | 10.3033 | 7.7318 | 0.7504 | 0.0462 |
| OS9 | "osteosarcoma amplified 9, endoplasmic reticulum associated protein" | 19.1711 | 14.3890 | 0.7506 | 0.0391 |
| aspscr1 | "alveolar soft part sarcoma chromosome region, candidate 1" | 2.7309 | 2.0501 | 0.7507 | 0.0364 |
| CSNK1E | "casein kinase 1, epsilon" | 20.9454 | 15.7266 | 0.7508 | 0.0412 |
| tbc1d9b | "TBC1 domain family, member 9B (with GRAM domain)" | 17.5545 | 13.1845 | 0.7511 | 0.0478 |
| Il17ra | interleukin 17 receptor A | 1.4649 | 1.1002 | 0.7511 | 0.0372 |
| C7orf26 | chromosome 7 open reading frame 26 | 9.2172 | 6.9230 | 0.7511 | 0.0458 |
| SYVN1 | "synovial apoptosis inhibitor 1, synoviolin" | 7.8234 | 5.8842 | 0.7521 | 0.0276 |
| LOC653375 | RCC1-like G exchanging factor-like; Williams-Beuren syndrome chromosome region 16 | 6.6684 | 5.0158 | 0.7522 | 0.0083 |
| WBSCR16 | RCC1-like G exchanging factor-like; Williams-Beuren syndrome chromosome region 16 | 6.6684 | 5.0158 | 0.7522 | 0.0083 |
| slc22a5 | "solute carrier family 22 (organic cation/carnitine transporter), member 5" | 1.0413 | 0.7836 | 0.7525 | 0.0234 |
| TRAK1 | "trafficking protein, kinesin binding 1" | 10.0774 | 7.5850 | 0.7527 | 0.0454 |
| UBOX5 | U-box domain containing 5 | 2.7346 | 2.0601 | 0.7533 | 0.0132 |
| SCMH1 | sex comb on midleg homolog 1 (Drosophila) | 7.1409 | 5.3820 | 0.7537 | 0.0351 |
| Atxn7l2 | ataxin 7-like 2 | 3.0653 | 2.3107 | 0.7538 | 0.0289 |
| Plxna3 | plexin A3 | 3.9919 | 3.0092 | 0.7538 | 0.0246 |
| wdr1 | WD repeat domain 1 | 10.5378 | 7.9469 | 0.7541 | 0.0195 |
| CNNM3 | cyclin M3 | 4.2047 | 3.1735 | 0.7547 | 0.0367 |
| Fbxo10 | F-box protein 10 | 2.3296 | 1.7584 | 0.7548 | 0.0446 |
| akt2 | v-akt murine thymoma viral oncogene homolog 2 | 6.3150 | 4.7699 | 0.7553 | 0.0429 |
| Cdan1 | "congenital dyserythropoietic anemia, type I" | 3.3567 | 2.5397 | 0.7566 | 0.0190 |
| Frmd4a | FERM domain containing 4A | 3.4255 | 2.5922 | 0.7567 | 0.0220 |
| LOC729799 | SEC14-like 1 (S. cerevisiae); SEC14-like 1 pseudogene | 10.5759 | 8.0047 | 0.7569 | 0.0030 |
| sec14l1 | SEC14-like 1 (S. cerevisiae); SEC14-like 1 pseudogene | 10.5759 | 8.0047 | 0.7569 | 0.0030 |
| ACCN2 | "amiloride-sensitive cation channel 2, neuronal" | 10.6198 | 8.0431 | 0.7574 | 0.0164 |
| VAC14 | Vac14 homolog (S. cerevisiae) | 4.7573 | 3.6116 | 0.7592 | 0.0431 |
| ZNF212 | zinc finger protein 212 | 3.2627 | 2.4780 | 0.7595 | 0.0154 |
| smcr7 | "Smith-Magenis syndrome chromosome region, candidate 7" | 3.9310 | 2.9898 | 0.7606 | 0.0184 |
| AGPAT1 | "1-acylglycerol-3-phosphate O-acyltransferase 1 (lysophosphatidic acid acyltransferase, alpha)" | 15.6525 | 11.9117 | 0.7610 | 0.0145 |
| AKAP1 | A kinase (PRKA) anchor protein 1 | 11.0159 | 8.4037 | 0.7629 | 0.0035 |
| Usp42 | ubiquitin specific peptidase 42 | 2.1415 | 1.6347 | 0.7633 | 0.0400 |
| OLFM1 | olfactomedin 1 | 140.4545 | 107.2560 | 0.7636 | 0.0412 |
| SLC25A15 | solute carrier family 25 (mitochondrial carrier; ornithine transporter) member 15 | 2.2050 | 1.6844 | 0.7639 | 0.0116 |
| PIAS3 | "protein inhibitor of activated STAT, 3" | 5.2348 | 4.0005 | 0.7642 | 0.0287 |
| TMEM132C | transmembrane protein 132C | 4.9229 | 3.7627 | 0.7643 | 0.0230 |
| OGG1 | 8-oxoguanine DNA glycosylase | 2.5927 | 1.9822 | 0.7645 | 0.0116 |
| SH2B3 | SH2B adaptor protein 3 | 3.4327 | 2.6263 | 0.7651 | 0.0454 |
| C17orf62 | chromosome 17 open reading frame 62 | 4.6373 | 3.5498 | 0.7655 | 0.0121 |
| MBD6 | methyl-CpG binding domain protein 6 | 4.9604 | 3.7983 | 0.7657 | 0.0188 |
| gpr173 | G protein-coupled receptor 173 | 5.9641 | 4.5715 | 0.7665 | 0.0284 |
| CSK | c-src tyrosine kinase | 5.9276 | 4.5438 | 0.7665 | 0.0490 |
| RILPL1 | Rab interacting lysosomal protein-like 1 | 6.2497 | 4.7951 | 0.7673 | 0.0443 |
| adcy8 | adenylate cyclase 8 (brain) | 2.7301 | 2.0966 | 0.7680 | 0.0334 |
| ZNF74 | zinc finger protein 74 | 2.8395 | 2.1807 | 0.7680 | 0.0442 |
| GRID1 | "glutamate receptor, ionotropic, delta 1" | 7.8316 | 6.0184 | 0.7685 | 0.0205 |
| DHX34 | DEAH (Asp-Glu-Ala-His) box polypeptide 34 | 3.6276 | 2.7902 | 0.7692 | 0.0398 |
| sumf2 | sulfatase modifying factor 2 | 13.3352 | 10.2628 | 0.7696 | 0.0122 |
| GGA1 | "golgi associated, gamma adaptin ear containing, ARF binding protein 1" | 6.3336 | 4.8744 | 0.7696 | 0.0311 |
| Wdr6 | WD repeat domain 6 | 15.0644 | 11.5974 | 0.7699 | 0.0099 |
| ADCY2 | adenylate cyclase 2 (brain) | 9.2131 | 7.0994 | 0.7706 | 0.0205 |
| pigo | "phosphatidylinositol glycan anchor biosynthesis, class O" | 2.2470 | 1.7318 | 0.7707 | 0.0225 |
| PARS2 | "prolyl-tRNA synthetase 2, mitochondrial (putative)" | 1.3621 | 1.0501 | 0.7710 | 0.0126 |
| WWP2 | WW domain containing E3 ubiquitin protein ligase 2 | 6.4327 | 4.9631 | 0.7715 | 0.0462 |
| MUL1 | mitochondrial E3 ubiquitin ligase 1 | 10.0322 | 7.7416 | 0.7717 | 0.0042 |
| atad3a | "ATPase family, AAA domain containing 3A" | 5.6091 | 4.3307 | 0.7721 | 0.0319 |
| ZNF784 | zinc finger protein 784 | 2.5150 | 1.9426 | 0.7724 | 0.0407 |
| C20orf4 | chromosome 20 open reading frame 4 | 4.7765 | 3.6909 | 0.7727 | 0.0316 |
| taf4 | "TAF4 RNA polymerase II, TATA box binding protein (TBP)-associated factor, 135kDa" | 2.5579 | 1.9784 | 0.7735 | 0.0047 |
| FZD4 | frizzled homolog 4 (Drosophila) | 2.0173 | 1.5616 | 0.7741 | 0.0154 |
| FAM20C | "family with sequence similarity 20, member C" | 6.7381 | 5.2256 | 0.7755 | 0.0427 |
| Sf1 | splicing factor 1 | 30.1204 | 23.3721 | 0.7760 | 0.0063 |
| Nkiras2 | NFKB inhibitor interacting Ras-like 2 | 2.4746 | 1.9223 | 0.7768 | 0.0112 |
| KDM3B | lysine (K)-specific demethylase 3B | 8.6393 | 6.7115 | 0.7769 | 0.0122 |
| RNF216L | ring finger protein 216-like | 1.8240 | 1.4178 | 0.7773 | 0.0048 |
| C4orf42 | chromosome 4 open reading frame 42 | 1.8446 | 1.4339 | 0.7773 | 0.0202 |
| jarid2 | "jumonji, AT rich interactive domain 2" | 3.9277 | 3.0567 | 0.7782 | 0.0296 |
| TMEM39B | transmembrane protein 39B | 3.5561 | 2.7677 | 0.7783 | 0.0035 |
| SRRM2 | serine/arginine repetitive matrix 2; hypothetical LOC100132779 | 33.0368 | 25.7140 | 0.7783 | 0.0475 |
| LOC100132779 | serine/arginine repetitive matrix 2; hypothetical LOC100132779 | 33.0368 | 25.7140 | 0.7783 | 0.0475 |
| UBTF | "upstream binding transcription factor, RNA polymerase I" | 13.9693 | 10.8746 | 0.7785 | 0.0077 |
| fam171a1 | "family with sequence similarity 171, member A1" | 42.1218 | 32.8081 | 0.7789 | 0.0478 |
| ELMO2 | engulfment and cell motility 2 | 13.5769 | 10.5764 | 0.7790 | 0.0021 |
| Mapkap1 | mitogen-activated protein kinase associated protein 1 | 12.3095 | 9.5923 | 0.7793 | 0.0481 |
| PCIF1 | PDX1 C-terminal inhibiting factor 1 | 12.0303 | 9.3807 | 0.7798 | 0.0245 |
| SURF4 | surfeit 4 | 24.5039 | 19.1205 | 0.7803 | 0.0034 |
| PGAP3 | post-GPI attachment to proteins 3 | 6.6053 | 5.1561 | 0.7806 | 0.0435 |
| DIS3L2 | DIS3 mitotic control homolog (S. cerevisiae)-like 2 | 1.8652 | 1.4560 | 0.7806 | 0.0349 |
| Lpcat3 | lysophosphatidylcholine acyltransferase 3 | 2.1228 | 1.6580 | 0.7811 | 0.0142 |
| Fam193a | chromosome 4 open reading frame 8 | 5.2720 | 4.1202 | 0.7815 | 0.0116 |
| Gorasp1 | "golgi reassembly stacking protein 1, 65kDa" | 8.6460 | 6.7617 | 0.7821 | 0.0113 |
| TXLNA | taxilin alpha | 8.2049 | 6.4189 | 0.7823 | 0.0133 |
| Rbm12 | RNA binding motif protein 12; copine I | 10.9789 | 8.5895 | 0.7824 | 0.0157 |
| CPNE1 | RNA binding motif protein 12; copine I | 10.9789 | 8.5895 | 0.7824 | 0.0157 |
| COLQ | collagen-like tail subunit (single strand of homotrimer) of asymmetric acetylcholinesterase | 2.4250 | 1.8974 | 0.7824 | 0.0482 |
| Zbtb9 | zinc finger and BTB domain containing 9 | 2.9240 | 2.2890 | 0.7828 | 0.0094 |
| UBE2J2 | "ubiquitin-conjugating enzyme E2, J2 (UBC6 homolog, yeast)" | 7.6021 | 5.9580 | 0.7837 | 0.0398 |
| Ino80 | INO80 homolog (S. cerevisiae) | 3.5608 | 2.7916 | 0.7840 | 0.0113 |
| dcaf7 | WD repeat domain 68 | 13.9802 | 10.9604 | 0.7840 | 0.0347 |
| SLC39A13 | "solute carrier family 39 (zinc transporter), member 13" | 9.0266 | 7.0789 | 0.7842 | 0.0356 |
| SMARCD1 | "SWI/SNF related, matrix associated, actin dependent regulator of chromatin, subfamily d, member 1" | 7.7733 | 6.1069 | 0.7856 | 0.0011 |
| SMAD7 | SMAD family member 7 | 6.0716 | 4.7738 | 0.7862 | 0.0474 |
| XRCC1 | X-ray repair complementing defective repair in Chinese hamster cells 1 | 5.2198 | 4.1046 | 0.7863 | 0.0498 |
| Baz2a | "bromodomain adjacent to zinc finger domain, 2A" | 6.7594 | 5.3180 | 0.7868 | 0.0409 |
| snx15 | sorting nexin 15 | 5.1705 | 4.0686 | 0.7869 | 0.0173 |
| ZDHHC18 | "zinc finger, DHHC-type containing 18" | 5.2989 | 4.1737 | 0.7877 | 0.0261 |
| N4BP1 | NEDD4 binding protein 1 | 4.6851 | 3.6904 | 0.7877 | 0.0239 |
| ZNF586 | zinc finger protein 586 | 1.0831 | 0.8533 | 0.7878 | 0.0428 |
| unk | unkempt homolog (Drosophila) | 3.4343 | 2.7100 | 0.7891 | 0.0206 |
| ZSWIM5 | "zinc finger, SWIM-type containing 5" | 1.8225 | 1.4392 | 0.7897 | 0.0065 |
| CHST10 | carbohydrate sulfotransferase 10 | 11.0981 | 8.7751 | 0.7907 | 0.0364 |
| thrA | "thyroid hormone receptor, alpha (erythroblastic leukemia viral (v-erb-a) oncogene homolog, avian)" | 43.0361 | 34.0557 | 0.7913 | 0.0486 |
| RMND5B | required for meiotic nuclear division 5 homolog B (S. cerevisiae) | 6.1302 | 4.8513 | 0.7914 | 0.0450 |
| CCDC123 | coiled-coil domain containing 123 | 1.6958 | 1.3424 | 0.7916 | 0.0314 |
| ilf3 | "interleukin enhancer binding factor 3, 90kDa" | 15.5324 | 12.2988 | 0.7918 | 0.0243 |
| TXNDC11 | thioredoxin domain containing 11 | 3.5952 | 2.8506 | 0.7929 | 0.0018 |
| DDX49 | DEAD (Asp-Glu-Ala-Asp) box polypeptide 49 | 10.2130 | 8.1041 | 0.7935 | 0.0493 |
| nadK | NAD kinase | 6.8709 | 5.4566 | 0.7942 | 0.0291 |
| ptpn1 | "protein tyrosine phosphatase, non-receptor type 1" | 7.7190 | 6.1357 | 0.7949 | 0.0127 |
| Man1b1 | "mannosidase, alpha, class 1B, member 1" | 7.1249 | 5.6654 | 0.7952 | 0.0364 |
| vopp1 | similar to EGFR-coamplified and overexpressed protein; EGFR-coamplified and overexpressed protein | 15.1525 | 12.0563 | 0.7957 | 0.0453 |
| LOC729086 | similar to EGFR-coamplified and overexpressed protein; EGFR-coamplified and overexpressed protein | 15.1525 | 12.0563 | 0.7957 | 0.0453 |
| DNAJB12 | "DnaJ (Hsp40) homolog, subfamily B, member 12" | 6.7716 | 5.3884 | 0.7957 | 0.0102 |
| abcb8 | "ATP-binding cassette, sub-family B (MDR/TAP), member 8" | 4.6360 | 3.6905 | 0.7960 | 0.0491 |
| LOC653303 | proprotein convertase subtilisin/kexin type 7 pseudogene; proprotein convertase subtilisin/kexin type 7 | 2.5786 | 2.0534 | 0.7963 | 0.0414 |
| pcsk7 | proprotein convertase subtilisin/kexin type 7 pseudogene; proprotein convertase subtilisin/kexin type 7 | 2.5786 | 2.0534 | 0.7963 | 0.0414 |
| PHF8 | PHD finger protein 8 | 2.4413 | 1.9459 | 0.7971 | 0.0304 |
| TFIP11 | tuftelin interacting protein 11 | 5.7776 | 4.6090 | 0.7977 | 0.0020 |
| TARS2 | "threonyl-tRNA synthetase 2, mitochondrial (putative)" | 7.8798 | 6.2912 | 0.7984 | 0.0341 |
| ZBTB49 | zinc finger protein 509 | 1.4947 | 1.1958 | 0.8000 | 0.0359 |
| DDB1 | "damage-specific DNA binding protein 1, 127kDa" | 15.2196 | 12.1907 | 0.8010 | 0.0442 |
| pfkfb3 | "6-phosphofructo-2-kinase/fructose-2,6-biphosphatase 3" | 21.7368 | 17.4299 | 0.8019 | 0.0204 |
| ITPK1 | "inositol 1,3,4-triphosphate 5/6 kinase" | 48.5985 | 39.0349 | 0.8032 | 0.0497 |
| PIGS | "phosphatidylinositol glycan anchor biosynthesis, class S" | 6.5087 | 5.2331 | 0.8040 | 0.0186 |
| POLDIP3 | "polymerase (DNA-directed), delta interacting protein 3" | 18.7054 | 15.0461 | 0.8044 | 0.0215 |
| Tyk2 | tyrosine kinase 2 | 7.6316 | 6.1411 | 0.8047 | 0.0398 |
| PES1 | "pescadillo homolog 1, containing BRCT domain (zebrafish)" | 8.2647 | 6.6579 | 0.8056 | 0.0307 |
| GLG1 | golgi apparatus protein 1 | 12.5262 | 10.1027 | 0.8065 | 0.0250 |
| CCNK | cyclin K | 3.0956 | 2.4975 | 0.8068 | 0.0457 |
| Recql5 | RecQ protein-like 5 | 2.6736 | 2.1596 | 0.8078 | 0.0046 |
| RBM28 | RNA binding motif protein 28 | 4.5738 | 3.6971 | 0.8083 | 0.0081 |
| Nfkbie | "nuclear factor of kappa light polypeptide gene enhancer in B-cells inhibitor, epsilon" | 3.2525 | 2.6314 | 0.8090 | 0.0481 |
| SPRY2 | sprouty homolog 2 (Drosophila) | 17.5777 | 14.2234 | 0.8092 | 0.0389 |
| ALG1 | "asparagine-linked glycosylation 1, beta-1,4-mannosyltransferase homolog (S. cerevisiae)" | 1.6328 | 1.3243 | 0.8111 | 0.0156 |
| SLC2A8 | "solute carrier family 2 (facilitated glucose transporter), member 8" | 5.2511 | 4.2647 | 0.8122 | 0.0408 |
| ATXN2 | ataxin 2 | 5.3826 | 4.3738 | 0.8126 | 0.0408 |
| IKBKB | "inhibitor of kappa light polypeptide gene enhancer in B-cells, kinase beta" | 2.2280 | 1.8122 | 0.8133 | 0.0348 |
| ARFGAP2 | ADP-ribosylation factor GTPase activating protein 2 | 17.9942 | 14.6851 | 0.8161 | 0.0372 |
| Tmem214 | transmembrane protein 214 | 7.1334 | 5.8226 | 0.8162 | 0.0331 |
| PTPRA | "protein tyrosine phosphatase, receptor type, A" | 45.5479 | 37.2446 | 0.8177 | 0.0006 |
| FAM193B | hypothetical protein FLJ10404 | 6.9542 | 5.6867 | 0.8177 | 0.0462 |
| ZSWIM1 | "zinc finger, SWIM-type containing 1" | 2.9985 | 2.4523 | 0.8179 | 0.0016 |
| DVL2 | "dishevelled, dsh homolog 2 (Drosophila)" | 4.6188 | 3.7792 | 0.8182 | 0.0487 |
| PMPCA | peptidase (mitochondrial processing) alpha | 9.2356 | 7.5584 | 0.8184 | 0.0292 |
| SIN3A | "SIN3 homolog A, transcription regulator (yeast)" | 2.7784 | 2.2796 | 0.8204 | 0.0007 |
| Setdb1 | "SET domain, bifurcated 1" | 2.1075 | 1.7296 | 0.8207 | 0.0394 |
| Cbx8 | "chromobox homolog 8 (Pc class homolog, Drosophila)" | 1.1400 | 0.9359 | 0.8210 | 0.0433 |
| PPP1R10 | "protein phosphatase 1, regulatory (inhibitor) subunit 10" | 9.0365 | 7.4368 | 0.8230 | 0.0349 |
| Setd8 | SET domain containing (lysine methyltransferase) 8 | 6.8334 | 5.6255 | 0.8232 | 0.0339 |
| DDX19A | DEAD (Asp-Glu-Ala-As) box polypeptide 19A | 3.7454 | 3.0905 | 0.8251 | 0.0086 |
| BRD1 | bromodomain containing 1 | 5.5031 | 4.5442 | 0.8257 | 0.0279 |
| tbc1d20 | "TBC1 domain family, member 20" | 9.6378 | 7.9610 | 0.8260 | 0.0310 |
| OSBPL2 | oxysterol binding protein-like 2 | 15.0828 | 12.4830 | 0.8276 | 0.0232 |
| sf3b3 | "splicing factor 3b, subunit 3, 130kDa" | 6.8275 | 5.6513 | 0.8277 | 0.0466 |
| Igf2r | insulin-like growth factor 2 receptor | 4.1334 | 3.4245 | 0.8285 | 0.0298 |
| TAF8 | "TAF8 RNA polymerase II, TATA box binding protein (TBP)-associated factor, 43kDa" | 1.8590 | 1.5415 | 0.8292 | 0.0369 |
| SPG7 | spastic paraplegia 7 (pure and complicated autosomal recessive) | 9.6957 | 8.0591 | 0.8312 | 0.0436 |
| DDX19B | DEAD (Asp-Glu-Ala-As) box polypeptide 19B | 1.5262 | 1.2698 | 0.8320 | 0.0203 |
| ACIN1 | apoptotic chromatin condensation inducer 1 | 11.3189 | 9.4284 | 0.8330 | 0.0396 |
| NRF1 | nuclear respiratory factor 1 | 4.1644 | 3.4850 | 0.8369 | 0.0275 |
| DCP1B | DCP1 decapping enzyme homolog B (S. cerevisiae) | 1.9460 | 1.6286 | 0.8369 | 0.0026 |
| USP4 | ubiquitin specific peptidase 4 (proto-oncogene) | 7.9340 | 6.6453 | 0.8376 | 0.0175 |
| LOC644006 | ring finger protein 4; hypothetical LOC644006 | 6.9665 | 5.8405 | 0.8384 | 0.0435 |
| Rnf4 | ring finger protein 4; hypothetical LOC644006 | 6.9665 | 5.8405 | 0.8384 | 0.0435 |
| CHD4 | chromodomain helicase DNA binding protein 4 | 15.0869 | 12.6487 | 0.8384 | 0.0038 |
| STAG3L3 | aminoacyl tRNA synthetase complex-interacting multifunctional protein 2; stromal antigen 3-like 3 | 3.0909 | 2.5961 | 0.8399 | 0.0434 |
| AIMP2 | aminoacyl tRNA synthetase complex-interacting multifunctional protein 2; stromal antigen 3-like 3 | 3.0909 | 2.5961 | 0.8399 | 0.0434 |
| BRD9 | bromodomain containing 9 | 3.9077 | 3.2956 | 0.8434 | 0.0338 |
| ISG20L2 | interferon stimulated exonuclease gene 20kDa-like 2 | 3.3740 | 2.8465 | 0.8436 | 0.0255 |
| URB2 | URB2 ribosome biogenesis 2 homolog (S. cerevisiae) | 1.2282 | 1.0371 | 0.8444 | 0.0156 |
| ZNF394 | zinc finger protein 394 | 2.4601 | 2.0775 | 0.8445 | 0.0150 |
| SDR39U1 | "short chain dehydrogenase/reductase family 39U, member 1" | 5.1554 | 4.3603 | 0.8458 | 0.0345 |
| rabl4 | "RAB, member of RAS oncogene family-like 4" | 4.0978 | 3.4659 | 0.8458 | 0.0377 |
| pdia6 | "protein disulfide isomerase family A, member 6" | 10.5822 | 8.9634 | 0.8470 | 0.0348 |
| Syt7 | synaptotagmin XVII; synaptotagmin VII | 3.7120 | 3.1533 | 0.8495 | 0.0121 |
| syt17 | synaptotagmin XVII; synaptotagmin VII | 3.7120 | 3.1533 | 0.8495 | 0.0121 |
| NUP188 | nucleoporin 188kDa | 3.0371 | 2.5808 | 0.8498 | 0.0031 |
| ZNF384 | zinc finger protein 384 | 5.5541 | 4.7207 | 0.8499 | 0.0105 |
| BCKDK | branched chain ketoacid dehydrogenase kinase | 7.4545 | 6.3374 | 0.8501 | 0.0406 |
| MYST4 | MYST histone acetyltransferase (monocytic leukemia) 4 | 2.9195 | 2.4820 | 0.8501 | 0.0473 |
| LCMT2 | leucine carboxyl methyltransferase 2 | 2.5098 | 2.1340 | 0.8503 | 0.0380 |
| GTF3C2 | "general transcription factor IIIC, polypeptide 2, beta 110kDa" | 3.8431 | 3.2732 | 0.8517 | 0.0484 |
| MTMR14 | myotubularin related protein 14 | 3.4407 | 2.9359 | 0.8533 | 0.0370 |
| EDC3 | enhancer of mRNA decapping 3 homolog (S. cerevisiae) | 4.6259 | 3.9493 | 0.8537 | 0.0185 |
| KIAA1539 | KIAA1539 | 5.0642 | 4.3264 | 0.8543 | 0.0325 |
| ZNF275 | zinc finger protein 275 | 3.6184 | 3.0919 | 0.8545 | 0.0086 |
| PI4KB | "phosphatidylinositol 4-kinase, catalytic, beta" | 13.1780 | 11.2703 | 0.8552 | 0.0410 |
| nonO | "non-POU domain containing, octamer-binding" | 18.0009 | 15.4396 | 0.8577 | 0.0418 |
| Tssc1 | tumor suppressing subtransferable candidate 1 | 5.4480 | 4.7119 | 0.8649 | 0.0412 |
| RPA1 | "replication protein A1, 70kDa" | 9.5789 | 8.3335 | 0.8700 | 0.0175 |
| sppl3 | signal peptide peptidase 3 | 9.1606 | 7.9940 | 0.8727 | 0.0281 |
| LRRC41 | leucine rich repeat containing 41 | 6.7931 | 5.9507 | 0.8760 | 0.0309 |
| THRAP3 | thyroid hormone receptor associated protein 3 | 20.5235 | 17.9823 | 0.8762 | 0.0168 |
| Tor1b | "torsin family 1, member B (torsin B)" | 5.5731 | 4.8925 | 0.8779 | 0.0228 |
| RNF185 | ring finger protein 185 | 8.6912 | 7.6557 | 0.8809 | 0.0451 |
| RAF1 | v-raf-1 murine leukemia viral oncogene homolog 1 | 7.5849 | 6.7339 | 0.8878 | 0.0486 |
| CPSF7 | "cleavage and polyadenylation specific factor 7, 59kDa" | 12.6494 | 11.2499 | 0.8894 | 0.0219 |
| SMARCAL1 | "SWI/SNF related, matrix associated, actin dependent regulator of chromatin, subfamily a-like 1" | 2.5828 | 2.2989 | 0.8901 | 0.0418 |
| nudt19 | nudix (nucleoside diphosphate linked moiety X)-type motif 19 | 1.8987 | 1.6912 | 0.8907 | 0.0472 |
| Tceb3 | "transcription elongation factor B (SIII), polypeptide 3 (110kDa, elongin A)" | 4.3390 | 3.9216 | 0.9038 | 0.0431 |
| RBM23 | RNA binding motif protein 23 | 6.8816 | 6.2844 | 0.9132 | 0.0428 |
| tbl2 | transducin (beta)-like 2 | 2.7838 | 2.5780 | 0.9261 | 0.0498 |
| fam192a | NEFA-interacting nuclear protein NIP30 | 21.6841 | 23.7124 | 1.0935 | 0.0290 |
| DRG1 | developmentally regulated GTP binding protein 1 | 15.6299 | 17.3940 | 1.1129 | 0.0351 |
| DKC1 | "dyskeratosis congenita 1, dyskerin" | 4.8087 | 5.3907 | 1.1210 | 0.0453 |
| RNF146 | ring finger protein 146 | 19.9319 | 22.4160 | 1.1246 | 0.0280 |
| MAGEF1 | "melanoma antigen family F, 1" | 25.9904 | 29.5570 | 1.1372 | 0.0204 |
| aldh3a2 | "aldehyde dehydrogenase 3 family, member A2" | 7.3440 | 8.3636 | 1.1388 | 0.0356 |
| Tmem111 | transmembrane protein 111 | 9.4315 | 10.8148 | 1.1467 | 0.0335 |
| Ttf1 | "transcription termination factor, RNA polymerase I" | 4.0148 | 4.6305 | 1.1533 | 0.0381 |
| DUSP12 | dual specificity phosphatase 12 | 5.4872 | 6.3297 | 1.1535 | 0.0411 |
| ube2d2 | "ubiquitin-conjugating enzyme E2D 2 (UBC4/5 homolog, yeast)" | 24.0383 | 27.8041 | 1.1567 | 0.0408 |
| trim69 | tripartite motif-containing 69 | 5.0354 | 5.8716 | 1.1661 | 0.0059 |
| CNIH4 | cornichon homolog 4 (Drosophila) | 4.3500 | 5.0856 | 1.1691 | 0.0144 |
| MRFAP1L1 | Morf4 family associated protein 1-like 1 | 36.7697 | 43.1531 | 1.1736 | 0.0465 |
| Khdrbs1 | "KH domain containing, RNA binding, signal transduction associated 1" | 20.6421 | 24.2845 | 1.1765 | 0.0278 |
| MTRF1L | mitochondrial translational release factor 1-like | 5.3067 | 6.2486 | 1.1775 | 0.0197 |
| ALG2 | "asparagine-linked glycosylation 2, alpha-1,3-mannosyltransferase homolog (S. cerevisiae)" | 4.6835 | 5.5258 | 1.1798 | 0.0400 |
| LOC645086 | chromosome 11 open reading frame 58 pseudogene; chromosome 11 open reading frame 58 | 12.3791 | 14.6211 | 1.1811 | 0.0284 |
| C11orf58 | chromosome 11 open reading frame 58 pseudogene; chromosome 11 open reading frame 58 | 12.3791 | 14.6211 | 1.1811 | 0.0284 |
| IFNGR2 | interferon gamma receptor 2 (interferon gamma transducer 1) | 6.5223 | 7.7048 | 1.1813 | 0.0424 |
| TPST1 | tyrosylprotein sulfotransferase 1 | 4.7324 | 5.5906 | 1.1813 | 0.0062 |
| LACTB | "lactamase, beta" | 2.1360 | 2.5256 | 1.1824 | 0.0460 |
| fam185a | "family with sequence similarity 185, member A; family with sequence similarity 185, member B pseudogene" | 1.8217 | 2.1651 | 1.1885 | 0.0193 |
| FAM185B | "family with sequence similarity 185, member A; family with sequence similarity 185, member B pseudogene" | 1.8217 | 2.1651 | 1.1885 | 0.0193 |
| Cops5 | COP9 constitutive photomorphogenic homolog subunit 5 (Arabidopsis) | 5.3848 | 6.4152 | 1.1914 | 0.0207 |
| tdrd7 | tudor domain containing 7 | 5.0575 | 6.0340 | 1.1931 | 0.0332 |
| PEBP1 | phosphatidylethanolamine binding protein 1 | 357.6730 | 428.5487 | 1.1982 | 0.0445 |
| msrA | methionine sulfoxide reductase A | 3.8232 | 4.5900 | 1.2006 | 0.0363 |
| mphosph10 | M-phase phosphoprotein 10 (U3 small nucleolar ribonucleoprotein) | 5.1196 | 6.1509 | 1.2014 | 0.0327 |
| trappc3 | trafficking protein particle complex 3 | 9.0146 | 10.8512 | 1.2037 | 0.0047 |
| RPGR | retinitis pigmentosa GTPase regulator | 1.0295 | 1.2408 | 1.2053 | 0.0389 |
| TP53RK | TP53 regulating kinase | 2.4041 | 2.8986 | 1.2057 | 0.0403 |
| CAMLG | calcium modulating ligand | 23.5954 | 28.5246 | 1.2089 | 0.0397 |
| Ccndbp1 | cyclin D-type binding-protein 1 | 8.5382 | 10.3262 | 1.2094 | 0.0337 |
| ZDHHC6 | "zinc finger, DHHC-type containing 6" | 8.1599 | 9.8961 | 1.2128 | 0.0358 |
| cct4 | "chaperonin containing TCP1, subunit 4 (delta)" | 26.5994 | 32.3136 | 1.2148 | 0.0498 |
| GGPS1 | geranylgeranyl diphosphate synthase 1 | 5.5477 | 6.7527 | 1.2172 | 0.0174 |
| Atp1b3 | "ATPase, Na+/K+ transporting, beta 3 polypeptide" | 23.3314 | 28.4238 | 1.2183 | 0.0341 |
| Dnaja1 | "DnaJ (Hsp40) homolog, subfamily A, member 1" | 80.1880 | 97.7276 | 1.2187 | 0.0464 |
| C9orf40 | chromosome 9 open reading frame 40 | 4.2906 | 5.2294 | 1.2188 | 0.0387 |
| nudt9 | nudix (nucleoside diphosphate linked moiety X)-type motif 9 | 9.6405 | 11.7644 | 1.2203 | 0.0406 |
| Ran | "RAN, member RAS oncogene family" | 31.2134 | 38.1510 | 1.2223 | 0.0447 |
| MED10 | mediator complex subunit 10 | 14.1460 | 17.3245 | 1.2247 | 0.0378 |
| FH | fumarate hydratase | 18.1925 | 22.2808 | 1.2247 | 0.0356 |
| NOP58 | NOP58 ribonucleoprotein homolog (yeast) | 7.7725 | 9.5283 | 1.2259 | 0.0353 |
| OAT | ornithine aminotransferase (gyrate atrophy) | 43.6686 | 53.6049 | 1.2275 | 0.0256 |
| Sumo1 | SMT3 suppressor of mif two 3 homolog 1 (S. cerevisiae); SUMO1 pseudogene 3 | 36.5042 | 44.8853 | 1.2296 | 0.0116 |
| SUMO1P3 | SMT3 suppressor of mif two 3 homolog 1 (S. cerevisiae); SUMO1 pseudogene 3 | 36.5042 | 44.8853 | 1.2296 | 0.0116 |
| ATP6V1G1 | "ATPase, H+ transporting, lysosomal 13kDa, V1 subunit G1" | 44.1393 | 54.3657 | 1.2317 | 0.0394 |
| PRNP | prion protein | 194.6768 | 239.9896 | 1.2328 | 0.0297 |
| MBD4 | methyl-CpG binding domain protein 4 | 6.8929 | 8.5125 | 1.2350 | 0.0185 |
| Chchd4 | coiled-coil-helix-coiled-coil-helix domain containing 4 | 7.2032 | 8.8984 | 1.2353 | 0.0273 |
| iscU | iron-sulfur cluster scaffold homolog (E. coli) | 25.6456 | 31.7298 | 1.2372 | 0.0201 |
| MED6 | mediator complex subunit 6 | 2.1993 | 2.7225 | 1.2379 | 0.0306 |
| LOC100132973 | "similar to elongin C; transcription elongation factor B (SIII), polypeptide 1 (15kDa, elongin C)" | 11.1647 | 13.8209 | 1.2379 | 0.0119 |
| TCEB1 | "similar to elongin C; transcription elongation factor B (SIII), polypeptide 1 (15kDa, elongin C)" | 11.1647 | 13.8209 | 1.2379 | 0.0119 |
| POLE3 | "polymerase (DNA directed), epsilon 3 (p17 subunit)" | 10.0650 | 12.4613 | 1.2381 | 0.0436 |
| C7orf44 | chromosome 7 open reading frame 44 | 2.4124 | 2.9871 | 1.2382 | 0.0093 |
| vdac2 | voltage-dependent anion channel 2 | 30.1831 | 37.4190 | 1.2397 | 0.0242 |
| c7orf30 | chromosome 7 open reading frame 30 | 2.9301 | 3.6361 | 1.2409 | 0.0325 |
| PSMD14 | "proteasome (prosome, macropain) 26S subunit, non-ATPase, 14" | 5.6621 | 7.0287 | 1.2414 | 0.0086 |
| Gtf2b | general transcription factor IIB | 5.9925 | 7.4504 | 1.2433 | 0.0058 |
| WDYHV1 | WDYHV motif containing 1 | 3.3396 | 4.1550 | 1.2441 | 0.0218 |
| LYRM4 | LYR motif containing 4 | 2.3992 | 2.9850 | 1.2441 | 0.0279 |
| Hexb | hexosaminidase B (beta polypeptide) | 4.8268 | 6.0067 | 1.2445 | 0.0237 |
| IQCB1 | IQ motif containing B1 | 4.6482 | 5.7895 | 1.2456 | 0.0445 |
| Psma4 | "proteasome (prosome, macropain) subunit, alpha type, 4" | 12.2197 | 15.2324 | 1.2465 | 0.0208 |
| HNRNPA1L2 | heterogeneous nuclear ribonucleoprotein A1-like 2 | 2.8500 | 3.5546 | 1.2472 | 0.0248 |
| Tmem81 | transmembrane protein 81 | 3.0012 | 3.7448 | 1.2478 | 0.0242 |
| MAP1LC3B | microtubule-associated protein 1 light chain 3 beta | 27.4360 | 34.2467 | 1.2482 | 0.0173 |
| c11orf57 | chromosome 11 open reading frame 57 | 4.7574 | 5.9479 | 1.2503 | 0.0387 |
| nfu1 | NFU1 iron-sulfur cluster scaffold homolog (S. cerevisiae) | 8.7201 | 10.9158 | 1.2518 | 0.0116 |
| Ttrap | TRAF and TNF receptor associated protein | 12.0318 | 15.0633 | 1.2520 | 0.0204 |
| C14orf142 | chromosome 14 open reading frame 142 | 4.8871 | 6.1192 | 1.2521 | 0.0401 |
| ptrh2 | peptidyl-tRNA hydrolase 2 | 2.0036 | 2.5148 | 1.2551 | 0.0163 |
| ENOPH1 | enolase-phosphatase 1 | 41.7421 | 52.4012 | 1.2554 | 0.0324 |
| RPL36AP51 | ribosomal protein L36a pseudogene 51; ribosomal protein L36a pseudogene 37; ribosomal protein L36a pseudogene 49; heterogeneous nuclear ribonucleoprotein H2 (H'); ribosomal protein L36a | 38.7841 | 48.7713 | 1.2575 | 0.0009 |
| Hnrnph2 | ribosomal protein L36a pseudogene 51; ribosomal protein L36a pseudogene 37; ribosomal protein L36a pseudogene 49; heterogeneous nuclear ribonucleoprotein H2 (H'); ribosomal protein L36a | 38.7841 | 48.7713 | 1.2575 | 0.0009 |
| RPL36A | ribosomal protein L36a pseudogene 51; ribosomal protein L36a pseudogene 37; ribosomal protein L36a pseudogene 49; heterogeneous nuclear ribonucleoprotein H2 (H'); ribosomal protein L36a | 38.7841 | 48.7713 | 1.2575 | 0.0009 |
| RPL36AP37 | ribosomal protein L36a pseudogene 51; ribosomal protein L36a pseudogene 37; ribosomal protein L36a pseudogene 49; heterogeneous nuclear ribonucleoprotein H2 (H'); ribosomal protein L36a | 38.7841 | 48.7713 | 1.2575 | 0.0009 |
| RPL36AP49 | ribosomal protein L36a pseudogene 51; ribosomal protein L36a pseudogene 37; ribosomal protein L36a pseudogene 49; heterogeneous nuclear ribonucleoprotein H2 (H'); ribosomal protein L36a | 38.7841 | 48.7713 | 1.2575 | 0.0009 |
| POLR2K | "polymerase (RNA) II (DNA directed) polypeptide K, 7.0kDa" | 27.2763 | 34.3029 | 1.2576 | 0.0103 |
| COQ3 | "coenzyme Q3 homolog, methyltransferase (S. cerevisiae)" | 5.9310 | 7.4665 | 1.2589 | 0.0178 |
| Gtf2e2 | "general transcription factor IIE, polypeptide 2, beta 34kDa" | 3.7030 | 4.6620 | 1.2590 | 0.0437 |
| set | SET nuclear oncogene; similar to SET translocation | 37.9024 | 47.8351 | 1.2621 | 0.0158 |
| LOC646817 | SET nuclear oncogene; similar to SET translocation | 37.9024 | 47.8351 | 1.2621 | 0.0158 |
| IGBP1 | chromosome 14 open reading frame 19; immunoglobulin (CD79A) binding protein 1 | 12.9580 | 16.3881 | 1.2647 | 0.0089 |
| C14orf19 | chromosome 14 open reading frame 19; immunoglobulin (CD79A) binding protein 1 | 12.9580 | 16.3881 | 1.2647 | 0.0089 |
| CLTA | "clathrin, light chain (Lca)" | 32.7494 | 41.4717 | 1.2663 | 0.0264 |
| KLHL7 | kelch-like 7 (Drosophila) | 6.6774 | 8.4567 | 1.2665 | 0.0397 |
| THYN1 | thymocyte nuclear protein 1 | 16.1817 | 20.5060 | 1.2672 | 0.0045 |
| RPL26L1 | ribosomal protein L26-like 1 | 7.1611 | 9.0771 | 1.2676 | 0.0457 |
| Exosc3 | exosome component 3 | 2.2130 | 2.8094 | 1.2695 | 0.0276 |
| znrf2 | zinc and ring finger 2 | 1.6570 | 2.1051 | 1.2704 | 0.0224 |
| MEAF6 | chromosome 1 open reading frame 149 | 19.0067 | 24.1795 | 1.2722 | 0.0417 |
| VPS29 | vacuolar protein sorting 29 homolog (S. cerevisiae) | 14.7041 | 18.7135 | 1.2727 | 0.0118 |
| fundc1 | FUN14 domain containing 1 | 14.4985 | 18.4653 | 1.2736 | 0.0373 |
| RfC4 | "replication factor C (activator 1) 4, 37kDa" | 1.9602 | 2.4995 | 1.2751 | 0.0314 |
| HDAC2 | histone deacetylase 2 | 2.9409 | 3.7506 | 1.2753 | 0.0475 |
| ENY2 | enhancer of yellow 2 homolog (Drosophila) | 4.5426 | 5.8035 | 1.2776 | 0.0085 |
| STARD3NL | STARD3 N-terminal like | 7.6498 | 9.7787 | 1.2783 | 0.0242 |
| c2orf47 | chromosome 2 open reading frame 47 | 3.8768 | 4.9558 | 1.2783 | 0.0295 |
| MRPL22 | mitochondrial ribosomal protein L22 | 2.1905 | 2.8016 | 1.2790 | 0.0359 |
| EIF3L | "eukaryotic translation initiation factor 3, subunit L" | 22.9869 | 29.5118 | 1.2839 | 0.0063 |
| CDKN1B | "cyclin-dependent kinase inhibitor 1B (p27, Kip1)" | 33.0760 | 42.5026 | 1.2850 | 0.0384 |
| EIF3J | "eukaryotic translation initiation factor 3, subunit J" | 15.8564 | 20.3812 | 1.2854 | 0.0412 |
| RSRC1 | arginine/serine-rich coiled-coil 1 | 4.4648 | 5.7417 | 1.2860 | 0.0400 |
| pdcd2l | programmed cell death 2-like | 1.4229 | 1.8298 | 1.2860 | 0.0261 |
| c7orf23 | chromosome 7 open reading frame 23 | 2.2121 | 2.8452 | 1.2862 | 0.0412 |
| ccdc99 | coiled-coil domain containing 99 | 1.3286 | 1.7090 | 1.2863 | 0.0427 |
| C9orf123 | chromosome 9 open reading frame 123 | 8.7170 | 11.2213 | 1.2873 | 0.0156 |
| FKBP3 | "FK506 binding protein 3, 25kDa" | 24.5721 | 31.6840 | 1.2894 | 0.0309 |
| MORF4 | mortality factor 4; mortality factor 4 like 1 | 55.3671 | 71.4091 | 1.2897 | 0.0023 |
| MORF4L1 | mortality factor 4; mortality factor 4 like 1 | 55.3671 | 71.4091 | 1.2897 | 0.0023 |
| ZFP28 | zinc finger protein 28 homolog (mouse) | 2.7257 | 3.5182 | 1.2908 | 0.0319 |
| NUDT5 | nudix (nucleoside diphosphate linked moiety X)-type motif 5 | 3.2763 | 4.2312 | 1.2915 | 0.0264 |
| PGRMC2 | progesterone receptor membrane component 2 | 9.8866 | 12.7719 | 1.2918 | 0.0280 |
| CCDC28A | coiled-coil domain containing 28A | 15.2136 | 19.6625 | 1.2924 | 0.0048 |
| ZC3H15 | zinc finger CCCH-type containing 15 | 19.7388 | 25.5187 | 1.2928 | 0.0349 |
| RWDD1L1 | RWD domain containing 1-like 1; RWD domain containing 1 | 7.0824 | 9.1722 | 1.2951 | 0.0225 |
| rwdd1 | RWD domain containing 1-like 1; RWD domain containing 1 | 7.0824 | 9.1722 | 1.2951 | 0.0225 |
| TAF9 | "TAF9 RNA polymerase II, TATA box binding protein (TBP)-associated factor, 32kDa" | 11.1702 | 14.4823 | 1.2965 | 0.0179 |
| MFF | mitochondrial fission factor | 11.3041 | 14.6560 | 1.2965 | 0.0387 |
| RAD51C | RAD51 homolog C (S. cerevisiae) | 4.3872 | 5.6970 | 1.2986 | 0.0453 |
| Zfp91-Cntf | zinc finger protein 91 homolog (mouse); ZFP91-CNTF readthrough transcript; ciliary neurotrophic factor | 10.7633 | 13.9897 | 1.2998 | 0.0383 |
| CNTF | zinc finger protein 91 homolog (mouse); ZFP91-CNTF readthrough transcript; ciliary neurotrophic factor | 10.7633 | 13.9897 | 1.2998 | 0.0383 |
| ZFP91 | zinc finger protein 91 homolog (mouse); ZFP91-CNTF readthrough transcript; ciliary neurotrophic factor | 10.7633 | 13.9897 | 1.2998 | 0.0383 |
| NOVA1 | neuro-oncological ventral antigen 1 | 8.6086 | 11.2011 | 1.3012 | 0.0446 |
| SNRPD1 | small nuclear ribonucleoprotein D1 polypeptide 16kDa; hypothetical protein LOC100129492 | 10.9566 | 14.2735 | 1.3027 | 0.0453 |
| LOC100129492 | small nuclear ribonucleoprotein D1 polypeptide 16kDa; hypothetical protein LOC100129492 | 10.9566 | 14.2735 | 1.3027 | 0.0453 |
| atp5h | "ATP synthase, H+ transporting, mitochondrial F0 complex, subunit d" | 20.0884 | 26.2424 | 1.3063 | 0.0238 |
| CENPJ | centromere protein J | 1.6887 | 2.2120 | 1.3099 | 0.0485 |
| Acn9 | ACN9 homolog (S. cerevisiae) | 3.1494 | 4.1265 | 1.3103 | 0.0238 |
| ap3s1 | "adaptor-related protein complex 3, sigma 1 subunit" | 16.9645 | 22.2451 | 1.3113 | 0.0376 |
| LOC440944 | hypothetical LOC440944 | 1.9936 | 2.6144 | 1.3114 | 0.0357 |
| Cox7a2l | cytochrome c oxidase subunit VIIa polypeptide 2 like | 13.8106 | 18.1211 | 1.3121 | 0.0018 |
| SMNDC1 | survival motor neuron domain containing 1 | 4.9125 | 6.4477 | 1.3125 | 0.0483 |
| HRASLS5 | "HRAS-like suppressor family, member 5" | 1.6557 | 2.1738 | 1.3130 | 0.0061 |
| c20orf30 | hypothetical LOC642975; chromosome 20 open reading frame 30 | 33.0314 | 43.4286 | 1.3148 | 0.0238 |
| LOC642975 | hypothetical LOC642975; chromosome 20 open reading frame 30 | 33.0314 | 43.4286 | 1.3148 | 0.0238 |
| Eapp | E2F-associated phosphoprotein | 9.7681 | 12.8449 | 1.3150 | 0.0255 |
| PIGH | "phosphatidylinositol glycan anchor biosynthesis, class H" | 4.0741 | 5.3604 | 1.3157 | 0.0007 |
| LYSMD2 | "LysM, putative peptidoglycan-binding, domain containing 2" | 13.3374 | 17.5498 | 1.3158 | 0.0067 |
| taf7 | "TAF7 RNA polymerase II, TATA box binding protein (TBP)-associated factor, 55kDa" | 23.3346 | 30.7051 | 1.3159 | 0.0373 |
| GTF3A | general transcription factor IIIA | 12.8450 | 16.9064 | 1.3162 | 0.0219 |
| FAM98B | "family with sequence similarity 98, member B" | 6.2005 | 8.1657 | 1.3169 | 0.0301 |
| UBXN8 | UBX domain protein 8 | 2.8277 | 3.7334 | 1.3203 | 0.0211 |
| ebag9 | "estrogen receptor binding site associated, antigen, 9" | 4.8201 | 6.3660 | 1.3207 | 0.0265 |
| PSMD10 | "proteasome (prosome, macropain) 26S subunit, non-ATPase, 10" | 14.8677 | 19.6457 | 1.3214 | 0.0295 |
| Atpif1 | ATPase inhibitory factor 1 | 33.8713 | 44.7751 | 1.3219 | 0.0235 |
| RGS2 | "regulator of G-protein signaling 2, 24kDa" | 8.0952 | 10.7064 | 1.3226 | 0.0346 |
| LOC550643 | hypothetical LOC550643 | 11.4796 | 15.1851 | 1.3228 | 0.0291 |
| C3orf26 | chromosome 3 open reading frame 26 | 4.5547 | 6.0319 | 1.3243 | 0.0062 |
| Gtpbp8 | GTP-binding protein 8 (putative) | 1.5963 | 2.1169 | 1.3261 | 0.0266 |
| DDX18 | DEAD (Asp-Glu-Ala-Asp) box polypeptide 18 | 4.4999 | 5.9708 | 1.3269 | 0.0159 |
| Psmc6 | "proteasome (prosome, macropain) 26S subunit, ATPase, 6" | 7.5535 | 10.0237 | 1.3270 | 0.0434 |
| vapa | "VAMP (vesicle-associated membrane protein)-associated protein A, 33kDa" | 11.5534 | 15.3528 | 1.3288 | 0.0345 |
| ociad2 | OCIA domain containing 2 | 6.4820 | 8.6228 | 1.3303 | 0.0113 |
| CACYBP | similar to calcyclin binding protein; calcyclin binding protein | 25.4883 | 33.9270 | 1.3311 | 0.0299 |
| LOC644877 | similar to calcyclin binding protein; calcyclin binding protein | 25.4883 | 33.9270 | 1.3311 | 0.0299 |
| IFIT1 | interferon-induced protein with tetratricopeptide repeats 1 | 11.8324 | 15.7505 | 1.3311 | 0.0011 |
| C6orf162 | chromosome 6 open reading frame 162 | 4.6516 | 6.1956 | 1.3319 | 0.0314 |
| SEC62 | SEC62 homolog (S. cerevisiae) | 26.2413 | 34.9884 | 1.3333 | 0.0314 |
| TRIAP1 | TP53 regulated inhibitor of apoptosis 1 | 6.3389 | 8.4600 | 1.3346 | 0.0457 |
| SPIN2B | "spindlin family, member 2B" | 3.5256 | 4.7058 | 1.3347 | 0.0205 |
| EIF1B | eukaryotic translation initiation factor 1B | 34.8068 | 46.4903 | 1.3357 | 0.0026 |
| itgb1bp1 | integrin beta 1 binding protein 1 | 10.7695 | 14.3956 | 1.3367 | 0.0061 |
| SFRS11 | "splicing factor, arginine/serine-rich 11" | 15.5581 | 20.8024 | 1.3371 | 0.0431 |
| CRBN | cereblon | 6.4397 | 8.6217 | 1.3388 | 0.0332 |
| PLEKHA3 | "pleckstrin homology domain containing, family A (phosphoinositide binding specific) member 3" | 6.2329 | 8.3522 | 1.3400 | 0.0295 |
| apoo | apolipoprotein O | 18.0476 | 24.2051 | 1.3412 | 0.0262 |
| CYCS | "cytochrome c, somatic" | 42.2950 | 56.7287 | 1.3413 | 0.0075 |
| SERINC1 | serine incorporator 1 | 197.3312 | 264.7223 | 1.3415 | 0.0287 |
| HSP90AA2 | "heat shock protein 90kDa alpha (cytosolic), class A member 2; heat shock protein 90kDa alpha (cytosolic), class A member 1" | 247.4170 | 332.9340 | 1.3456 | 0.0041 |
| HSP90AA1 | "heat shock protein 90kDa alpha (cytosolic), class A member 2; heat shock protein 90kDa alpha (cytosolic), class A member 1" | 247.4170 | 332.9340 | 1.3456 | 0.0041 |
| MTX2 | metaxin 2 | 18.7274 | 25.2039 | 1.3458 | 0.0190 |
| hnrpll | heterogeneous nuclear ribonucleoprotein L-like | 4.8379 | 6.5156 | 1.3468 | 0.0390 |
| RRP15 | ribosomal RNA processing 15 homolog (S. cerevisiae) | 1.5114 | 2.0379 | 1.3483 | 0.0402 |
| Tceal1 | transcription elongation factor A (SII)-like 1 | 17.9147 | 24.1871 | 1.3501 | 0.0151 |
| TCEAL8 | transcription elongation factor A (SII)-like 8 | 27.9782 | 37.7829 | 1.3504 | 0.0117 |
| Chmp5 | chromatin modifying protein 5 | 31.7762 | 42.9833 | 1.3527 | 0.0134 |
| UBE2F | ubiquitin-conjugating enzyme E2F (putative) | 2.5284 | 3.4227 | 1.3537 | 0.0044 |
| arsk | "arylsulfatase family, member K" | 1.2789 | 1.7314 | 1.3538 | 0.0134 |
| C12orf73 | chromosome 12 open reading frame 73 | 2.3931 | 3.2401 | 1.3539 | 0.0074 |
| C6orf130 | chromosome 6 open reading frame 130 | 2.5405 | 3.4404 | 1.3542 | 0.0489 |
| dynll1 | "dynein, light chain, LC8-type 1" | 70.1253 | 95.0437 | 1.3553 | 0.0371 |
| NARS2 | "asparaginyl-tRNA synthetase 2, mitochondrial (putative)" | 2.3820 | 3.2292 | 1.3557 | 0.0061 |
| PDCD10 | programmed cell death 10 | 6.6312 | 9.0087 | 1.3585 | 0.0362 |
| WBP4 | WW domain binding protein 4 (formin binding protein 21) | 9.3188 | 12.6628 | 1.3588 | 0.0049 |
| frg1 | FSHD region gene 1 | 8.5888 | 11.6713 | 1.3589 | 0.0184 |
| SFRS2B | "splicing factor, arginine/serine-rich 2B" | 3.9455 | 5.3640 | 1.3595 | 0.0468 |
| MRPL50 | mitochondrial ribosomal protein L50 | 6.8447 | 9.3139 | 1.3607 | 0.0199 |
| GLRX | glutaredoxin (thioltransferase) | 2.7564 | 3.7571 | 1.3630 | 0.0445 |
| RPL15P22 | ribosomal protein L15 pseudogene 22; ribosomal protein L15 pseudogene 18; ribosomal protein L15 pseudogene 17; ribosomal protein L15 pseudogene 3; ribosomal protein L15 pseudogene 7; ribosomal protein L15 | 62.5651 | 85.4534 | 1.3658 | 0.0204 |
| RPL15P18 | ribosomal protein L15 pseudogene 22; ribosomal protein L15 pseudogene 18; ribosomal protein L15 pseudogene 17; ribosomal protein L15 pseudogene 3; ribosomal protein L15 pseudogene 7; ribosomal protein L15 | 62.5651 | 85.4534 | 1.3658 | 0.0204 |
| RPL15P7 | ribosomal protein L15 pseudogene 22; ribosomal protein L15 pseudogene 18; ribosomal protein L15 pseudogene 17; ribosomal protein L15 pseudogene 3; ribosomal protein L15 pseudogene 7; ribosomal protein L15 | 62.5651 | 85.4534 | 1.3658 | 0.0204 |
| RPL15 | ribosomal protein L15 pseudogene 22; ribosomal protein L15 pseudogene 18; ribosomal protein L15 pseudogene 17; ribosomal protein L15 pseudogene 3; ribosomal protein L15 pseudogene 7; ribosomal protein L15 | 62.5651 | 85.4534 | 1.3658 | 0.0204 |
| RPL15P3 | ribosomal protein L15 pseudogene 22; ribosomal protein L15 pseudogene 18; ribosomal protein L15 pseudogene 17; ribosomal protein L15 pseudogene 3; ribosomal protein L15 pseudogene 7; ribosomal protein L15 | 62.5651 | 85.4534 | 1.3658 | 0.0204 |
| RPL15P17 | ribosomal protein L15 pseudogene 22; ribosomal protein L15 pseudogene 18; ribosomal protein L15 pseudogene 17; ribosomal protein L15 pseudogene 3; ribosomal protein L15 pseudogene 7; ribosomal protein L15 | 62.5651 | 85.4534 | 1.3658 | 0.0204 |
| NUDT2 | nudix (nucleoside diphosphate linked moiety X)-type motif 2 | 9.7999 | 13.3864 | 1.3660 | 0.0434 |
| TMEM126B | transmembrane protein 126B | 14.0413 | 19.1815 | 1.3661 | 0.0436 |
| TATDN1 | TatD DNase domain containing 1 | 1.9748 | 2.6993 | 1.3668 | 0.0432 |
| PGRMC1 | progesterone receptor membrane component 1 | 67.8598 | 92.7585 | 1.3669 | 0.0102 |
| LOC644589 | "similar to translocase of the inner mitochondrial membrane 14; DnaJ (Hsp40) homolog, subfamily C, member 19" | 7.0575 | 9.6474 | 1.3670 | 0.0160 |
| DNAJC19 | "similar to translocase of the inner mitochondrial membrane 14; DnaJ (Hsp40) homolog, subfamily C, member 19" | 7.0575 | 9.6474 | 1.3670 | 0.0160 |
| mRpS33 | mitochondrial ribosomal protein S33 | 9.3822 | 12.8441 | 1.3690 | 0.0068 |
| UBE2D1 | "ubiquitin-conjugating enzyme E2D 1 (UBC4/5 homolog, yeast)" | 5.1393 | 7.0421 | 1.3703 | 0.0414 |
| ACTR6 | ARP6 actin-related protein 6 homolog (yeast) | 8.2699 | 11.3455 | 1.3719 | 0.0439 |
| SNORA25 | "TATA box binding protein (TBP)-associated factor, RNA polymerase I, D, 41kDa; small nucleolar RNA, H/ACA box 32; small nucleolar RNA, H/ACA box 25" | 4.7451 | 6.5162 | 1.3733 | 0.0499 |
| TAF1D | "TATA box binding protein (TBP)-associated factor, RNA polymerase I, D, 41kDa; small nucleolar RNA, H/ACA box 32; small nucleolar RNA, H/ACA box 25" | 4.7451 | 6.5162 | 1.3733 | 0.0499 |
| SNORA32 | "TATA box binding protein (TBP)-associated factor, RNA polymerase I, D, 41kDa; small nucleolar RNA, H/ACA box 32; small nucleolar RNA, H/ACA box 25" | 4.7451 | 6.5162 | 1.3733 | 0.0499 |
| NDUFB6 | "NADH dehydrogenase (ubiquinone) 1 beta subcomplex, 6, 17kDa" | 20.2531 | 27.8342 | 1.3743 | 0.0215 |
| Rnf219 | ring finger protein 219 | 2.4505 | 3.3685 | 1.3746 | 0.0433 |
| fam188a | chromosome 10 open reading frame 97 | 6.8889 | 9.4725 | 1.3750 | 0.0142 |
| FAM92A2 | "family with sequence similarity 92, member A2; family with sequence similarity 92, member A1" | 2.9529 | 4.0605 | 1.3751 | 0.0369 |
| FAM92A1 | "family with sequence similarity 92, member A2; family with sequence similarity 92, member A1" | 2.9529 | 4.0605 | 1.3751 | 0.0369 |
| MRPL1 | mitochondrial ribosomal protein L1 | 7.3925 | 10.1724 | 1.3760 | 0.0090 |
| C4orf43 | chromosome 4 open reading frame 43 | 1.8688 | 2.5716 | 1.3761 | 0.0166 |
| HIGD1A | "similar to HIG1 domain family, member 1A; HIG1 hypoxia inducible domain family, member 1A; HIG1 hypoxia inducible domain family, member 1D" | 92.5548 | 127.4069 | 1.3766 | 0.0479 |
| HIGD1D | "similar to HIG1 domain family, member 1A; HIG1 hypoxia inducible domain family, member 1A; HIG1 hypoxia inducible domain family, member 1D" | 92.5548 | 127.4069 | 1.3766 | 0.0479 |
| LOC100130383 | "similar to HIG1 domain family, member 1A; HIG1 hypoxia inducible domain family, member 1A; HIG1 hypoxia inducible domain family, member 1D" | 92.5548 | 127.4069 | 1.3766 | 0.0479 |
| vbp1 | von Hippel-Lindau binding protein 1 | 20.0886 | 27.6534 | 1.3766 | 0.0315 |
| GSTO1 | glutathione S-transferase omega 1 | 27.4511 | 37.7916 | 1.3767 | 0.0038 |
| MAT2B | "methionine adenosyltransferase II, beta" | 10.9946 | 15.1370 | 1.3768 | 0.0167 |
| BRP44 | brain protein 44 | 18.1134 | 24.9669 | 1.3784 | 0.0155 |
| dnajb9 | "DnaJ (Hsp40) homolog, subfamily B, member 9" | 10.9062 | 15.0836 | 1.3830 | 0.0463 |
| Ptges3 | prostaglandin E synthase 3 (cytosolic) | 48.1904 | 66.6912 | 1.3839 | 0.0158 |
| CCNB1IP1 | cyclin B1 interacting protein 1 | 4.3061 | 5.9732 | 1.3871 | 0.0396 |
| MRPS35 | mitochondrial ribosomal protein S35 | 13.4211 | 18.6218 | 1.3875 | 0.0248 |
| cir1 | corepressor interacting with RBPJ | 8.3451 | 11.5811 | 1.3878 | 0.0364 |
| DSN1 | "DSN1, MIND kinetochore complex component, homolog (S. cerevisiae)" | 1.9331 | 2.6916 | 1.3924 | 0.0335 |
| KCNMB4 | "potassium large conductance calcium-activated channel, subfamily M, beta member 4" | 13.7836 | 19.1966 | 1.3927 | 0.0174 |
| mettl5 | methyltransferase like 5 | 5.1308 | 7.1494 | 1.3934 | 0.0056 |
| polB | "polymerase (DNA directed), beta" | 2.8000 | 3.9075 | 1.3955 | 0.0239 |
| CALM3 | "calmodulin 3 (phosphorylase kinase, delta); calmodulin 2 (phosphorylase kinase, delta); calmodulin 1 (phosphorylase kinase, delta)" | 117.7632 | 164.4545 | 1.3965 | 0.0239 |
| CALM2 | "calmodulin 3 (phosphorylase kinase, delta); calmodulin 2 (phosphorylase kinase, delta); calmodulin 1 (phosphorylase kinase, delta)" | 117.7632 | 164.4545 | 1.3965 | 0.0239 |
| CALM1 | "calmodulin 3 (phosphorylase kinase, delta); calmodulin 2 (phosphorylase kinase, delta); calmodulin 1 (phosphorylase kinase, delta)" | 117.7632 | 164.4545 | 1.3965 | 0.0239 |
| NIPSNAP3B | nipsnap homolog 3B (C. elegans) | 3.0899 | 4.3179 | 1.3974 | 0.0310 |
| RPL23AP82 | ribosomal protein L23a pseudogene 25; ribosomal protein L23a pseudogene 82 | 3.9491 | 5.5189 | 1.3975 | 0.0028 |
| RPL23AP25 | ribosomal protein L23a pseudogene 25; ribosomal protein L23a pseudogene 82 | 3.9491 | 5.5189 | 1.3975 | 0.0028 |
| C12orf76 | chromosome 12 open reading frame 76 | 9.9003 | 13.8400 | 1.3979 | 0.0300 |
| MTERFD1 | MTERF domain containing 1 | 4.4714 | 6.2509 | 1.3980 | 0.0167 |
| LOC729852 | hypothetical protein LOC729852 | 3.9589 | 5.5359 | 1.3983 | 0.0235 |
| LOC439953 | similar to TRIMCyp; peptidylprolyl isomerase A (cyclophilin A); peptidylprolyl isomerase A (cyclophilin A)-like 3 | 104.0579 | 145.8011 | 1.4012 | 0.0147 |
| ppiA | similar to TRIMCyp; peptidylprolyl isomerase A (cyclophilin A); peptidylprolyl isomerase A (cyclophilin A)-like 3 | 104.0579 | 145.8011 | 1.4012 | 0.0147 |
| PPIAL3 | similar to TRIMCyp; peptidylprolyl isomerase A (cyclophilin A); peptidylprolyl isomerase A (cyclophilin A)-like 3 | 104.0579 | 145.8011 | 1.4012 | 0.0147 |
| MKKS | McKusick-Kaufman syndrome | 16.5876 | 23.2656 | 1.4026 | 0.0249 |
| TMEM70 | transmembrane protein 70 | 7.3298 | 10.2856 | 1.4033 | 0.0216 |
| pts | 6-pyruvoyltetrahydropterin synthase | 5.7343 | 8.0619 | 1.4059 | 0.0160 |
| NSMCE2 | "non-SMC element 2, MMS21 homolog (S. cerevisiae)" | 2.3162 | 3.2571 | 1.4062 | 0.0287 |
| Zfand1 | "zinc finger, AN1-type domain 1" | 5.2923 | 7.4458 | 1.4069 | 0.0316 |
| Cdkl3 | cyclin-dependent kinase-like 3 | 1.1079 | 1.5595 | 1.4076 | 0.0329 |
| cisd2 | CDGSH iron sulfur domain 2 | 9.2833 | 13.0681 | 1.4077 | 0.0386 |
| PTPLA | "protein tyrosine phosphatase-like (proline instead of catalytic arginine), member A" | 1.3431 | 1.8925 | 1.4091 | 0.0497 |
| GGH | "gamma-glutamyl hydrolase (conjugase, folylpolygammaglutamyl hydrolase)" | 2.9818 | 4.2131 | 1.4130 | 0.0208 |
| LOC644101 | "similar to chromobox homolog 3; chromobox homolog 3 (HP1 gamma homolog, Drosophila)" | 13.1369 | 18.5679 | 1.4134 | 0.0235 |
| CBX3 | "similar to chromobox homolog 3; chromobox homolog 3 (HP1 gamma homolog, Drosophila)" | 13.1369 | 18.5679 | 1.4134 | 0.0235 |
| SPDYA | "protein phosphatase 1, catalytic subunit, beta isoform; speedy homolog A (Xenopus laevis)" | 26.6592 | 37.6903 | 1.4138 | 0.0301 |
| PPP1CB | "protein phosphatase 1, catalytic subunit, beta isoform; speedy homolog A (Xenopus laevis)" | 26.6592 | 37.6903 | 1.4138 | 0.0301 |
| cfdp1 | craniofacial development protein 1 | 8.1872 | 11.5855 | 1.4151 | 0.0337 |
| ccdc104 | coiled-coil domain containing 104 | 16.9421 | 24.0584 | 1.4200 | 0.0118 |
| CHORDC1 | cysteine and histidine-rich domain (CHORD)-containing 1; cysteine and histidine-rich domain (CHORD)-containing 1 pseudogene | 3.5675 | 5.0811 | 1.4243 | 0.0094 |
| LOC727896 | cysteine and histidine-rich domain (CHORD)-containing 1; cysteine and histidine-rich domain (CHORD)-containing 1 pseudogene | 3.5675 | 5.0811 | 1.4243 | 0.0094 |
| ube2b | ubiquitin-conjugating enzyme E2B (RAD6 homolog) | 19.0737 | 27.1937 | 1.4257 | 0.0160 |
| NDUFS4 | "NADH dehydrogenase (ubiquinone) Fe-S protein 4, 18kDa (NADH-coenzyme Q reductase)" | 17.1034 | 24.3978 | 1.4265 | 0.0259 |
| ECT2 | epithelial cell transforming sequence 2 oncogene | 1.5120 | 2.1578 | 1.4271 | 0.0447 |
| ARL6IP5 | ADP-ribosylation-like factor 6 interacting protein 5 | 52.4406 | 74.9334 | 1.4289 | 0.0137 |
| H2AFZ | "H2A histone family, member Z" | 22.3048 | 31.8777 | 1.4292 | 0.0010 |
| Ap1s2 | "adaptor-related protein complex 1, sigma 2 subunit pseudogene; adaptor-related protein complex 1, sigma 2 subunit" | 8.2103 | 11.7758 | 1.4343 | 0.0262 |
| LOC653653 | "adaptor-related protein complex 1, sigma 2 subunit pseudogene; adaptor-related protein complex 1, sigma 2 subunit" | 8.2103 | 11.7758 | 1.4343 | 0.0262 |
| STAG3L4 | stromal antigen 3-like 4 | 2.5337 | 3.6368 | 1.4354 | 0.0027 |
| Tomm22 | translocase of outer mitochondrial membrane 22 homolog (yeast) | 17.0106 | 24.4361 | 1.4365 | 0.0048 |
| BNIP3L | BCL2/adenovirus E1B 19kDa interacting protein 3-like | 28.8241 | 41.4724 | 1.4388 | 0.0404 |
| Upf3b | UPF3 regulator of nonsense transcripts homolog B (yeast) | 5.6284 | 8.0989 | 1.4389 | 0.0027 |
| AMD1 | adenosylmethionine decarboxylase 1 | 26.4339 | 38.0613 | 1.4399 | 0.0440 |
| smpdl3a | "sphingomyelin phosphodiesterase, acid-like 3A" | 2.8974 | 4.1774 | 1.4418 | 0.0311 |
| MAPKSP1 | MAPK scaffold protein 1 | 9.3612 | 13.5059 | 1.4427 | 0.0444 |
| Paip2 | poly(A) binding protein interacting protein 2 | 42.5660 | 61.4954 | 1.4447 | 0.0016 |
| FGFR1OP2 | FGFR1 oncogene partner 2 | 12.8738 | 18.6004 | 1.4448 | 0.0470 |
| USP45 | ubiquitin specific peptidase 45 | 1.2211 | 1.7645 | 1.4451 | 0.0420 |
| LOC375190 | chromosome 2 open reading frame 84; hypothetical protein LOC375190 | 1.9136 | 2.7656 | 1.4453 | 0.0127 |
| C2orf84 | chromosome 2 open reading frame 84; hypothetical protein LOC375190 | 1.9136 | 2.7656 | 1.4453 | 0.0127 |
| PIGP | "phosphatidylinositol glycan anchor biosynthesis, class P" | 1.9147 | 2.7698 | 1.4466 | 0.0032 |
| thoc7 | THO complex 7 homolog (Drosophila) | 16.0911 | 23.3263 | 1.4496 | 0.0247 |
| dut | deoxyuridine triphosphatase | 8.6941 | 12.6169 | 1.4512 | 0.0340 |
| ARMC1 | armadillo repeat containing 1 | 11.2898 | 16.3885 | 1.4516 | 0.0374 |
| Asnsd1 | asparagine synthetase domain containing 1 | 11.9077 | 17.2955 | 1.4525 | 0.0045 |
| THAP10 | THAP domain containing 10 | 3.1620 | 4.5945 | 1.4530 | 0.0105 |
| GLRX2 | glutaredoxin 2 | 6.4374 | 9.3573 | 1.4536 | 0.0165 |
| SNRNP27 | small nuclear ribonucleoprotein 27kDa (U4/U6.U5) | 8.6598 | 12.6073 | 1.4558 | 0.0478 |
| BLOC1S2 | "biogenesis of lysosomal organelles complex-1, subunit 2" | 19.0141 | 27.6886 | 1.4562 | 0.0014 |
| LOC727947 | similar to ubiquinol-cytochrome c reductase binding protein; ubiquinol-cytochrome c reductase binding protein pseudogene; ubiquinol-cytochrome c reductase binding protein | 15.4931 | 22.5819 | 1.4575 | 0.0234 |
| LOC442454 | similar to ubiquinol-cytochrome c reductase binding protein; ubiquinol-cytochrome c reductase binding protein pseudogene; ubiquinol-cytochrome c reductase binding protein | 15.4931 | 22.5819 | 1.4575 | 0.0234 |
| UQCRB | similar to ubiquinol-cytochrome c reductase binding protein; ubiquinol-cytochrome c reductase binding protein pseudogene; ubiquinol-cytochrome c reductase binding protein | 15.4931 | 22.5819 | 1.4575 | 0.0234 |
| NAP1L1 | nucleosome assembly protein 1-like 1 | 26.8676 | 39.1823 | 1.4584 | 0.0021 |
| ZNF302 | zinc finger protein 302 | 7.4659 | 10.8958 | 1.4594 | 0.0260 |
| C4orf27 | chromosome 4 open reading frame 27 | 11.9951 | 17.5098 | 1.4598 | 0.0278 |
| atp5l | "ATP synthase, H+ transporting, mitochondrial F0 complex, subunit G" | 14.1866 | 20.7133 | 1.4601 | 0.0144 |
| BTF3L4 | basic transcription factor 3-like 4; similar to hCG2008008 | 7.2300 | 10.5597 | 1.4605 | 0.0233 |
| LOC100131401 | basic transcription factor 3-like 4; similar to hCG2008008 | 7.2300 | 10.5597 | 1.4605 | 0.0233 |
| TMEM14A | transmembrane protein 14A | 44.0368 | 64.3190 | 1.4606 | 0.0032 |
| Ndufa4 | "NADH dehydrogenase (ubiquinone) 1 alpha subcomplex, 4, 9kDa" | 69.8566 | 102.0613 | 1.4610 | 0.0147 |
| BMI1 | BMI1 polycomb ring finger oncogene | 8.3221 | 12.1822 | 1.4638 | 0.0326 |
| PPA1 | pyrophosphatase (inorganic) 1 | 43.9257 | 64.3727 | 1.4655 | 0.0115 |
| c6orf211 | chromosome 6 open reading frame 211 | 6.0706 | 8.8976 | 1.4657 | 0.0180 |
| MED21 | mediator complex subunit 21 | 6.3349 | 9.3154 | 1.4705 | 0.0422 |
| NDUFAB1 | "NADH dehydrogenase (ubiquinone) 1, alpha/beta subcomplex, 1, 8kDa" | 22.6193 | 33.2708 | 1.4709 | 0.0003 |
| PCNP | PEST proteolytic signal containing nuclear protein | 39.5107 | 58.1978 | 1.4730 | 0.0390 |
| CCDC91 | coiled-coil domain containing 91 | 5.4896 | 8.0875 | 1.4732 | 0.0084 |
| BAG2 | BCL2-associated athanogene 2 | 1.6198 | 2.3866 | 1.4734 | 0.0306 |
| UQCRHL | ubiquinol-cytochrome c reductase hinge protein-like; ubiquinol-cytochrome c reductase hinge protein | 75.8500 | 112.1360 | 1.4784 | 0.0077 |
| Uqcrh | ubiquinol-cytochrome c reductase hinge protein-like; ubiquinol-cytochrome c reductase hinge protein | 75.8500 | 112.1360 | 1.4784 | 0.0077 |
| ripply2 | chromosome 6 open reading frame 59; ripply2 homolog (zebrafish) | 6.2534 | 9.2459 | 1.4785 | 0.0071 |
| C6orf59 | chromosome 6 open reading frame 59; ripply2 homolog (zebrafish) | 6.2534 | 9.2459 | 1.4785 | 0.0071 |
| C14orf2 | chromosome 14 open reading frame 2 | 17.1543 | 25.4054 | 1.4810 | 0.0103 |
| arl6ip1 | ADP-ribosylation factor-like 6 interacting protein 1 | 73.9868 | 109.6755 | 1.4824 | 0.0144 |
| ACYP1 | "acylphosphatase 1, erythrocyte (common) type" | 5.9461 | 8.8340 | 1.4857 | 0.0040 |
| PIGK | "phosphatidylinositol glycan anchor biosynthesis, class K" | 5.0884 | 7.5965 | 1.4929 | 0.0369 |
| COPS2 | COP9 constitutive photomorphogenic homolog subunit 2 (Arabidopsis) | 9.5241 | 14.2532 | 1.4966 | 0.0394 |
| sub1 | SUB1 homolog (S. cerevisiae) | 16.3449 | 24.4620 | 1.4966 | 0.0374 |
| c11orf74 | chromosome 11 open reading frame 74 | 4.8907 | 7.3378 | 1.5003 | 0.0037 |
| SH3GL3 | SH3-domain GRB2-like 3 | 14.8748 | 22.4269 | 1.5077 | 0.0372 |
| IMPA1 | inositol(myo)-1(or 4)-monophosphatase 1 | 8.1077 | 12.2320 | 1.5087 | 0.0447 |
| DSTN | destrin (actin depolymerizing factor) | 95.8469 | 144.6122 | 1.5088 | 0.0084 |
| PFDN4 | prefoldin subunit 4 | 9.0119 | 13.6626 | 1.5161 | 0.0072 |
| Ankrd46 | ankyrin repeat domain 46 | 11.3100 | 17.1730 | 1.5184 | 0.0403 |
| Yeats4 | YEATS domain containing 4 | 4.4571 | 6.7813 | 1.5215 | 0.0208 |
| AASDHPPT | aminoadipate-semialdehyde dehydrogenase-phosphopantetheinyl transferase | 12.0176 | 18.4405 | 1.5345 | 0.0392 |
| C3orf14 | chromosome 3 open reading frame 14 | 7.1947 | 11.0410 | 1.5346 | 0.0302 |
| GUSBP1 | "glucuronidase, beta-like 2; glucuronidase, beta pseudogene" | 1.1156 | 1.7125 | 1.5351 | 0.0138 |
| GUSBL2 | "glucuronidase, beta-like 2; glucuronidase, beta pseudogene" | 1.1156 | 1.7125 | 1.5351 | 0.0138 |
| Rpa3 | "replication protein A3, 14kDa" | 2.5117 | 3.8598 | 1.5367 | 0.0009 |
| SKP1 | S-phase kinase-associated protein 1 | 20.8248 | 32.0719 | 1.5401 | 0.0081 |
| C1orf151 | chromosome 1 open reading frame 151 | 3.1729 | 4.8871 | 1.5403 | 0.0058 |
| CCDC126 | coiled-coil domain containing 126 | 1.8664 | 2.8790 | 1.5425 | 0.0407 |
| C12orf62 | chromosome 12 open reading frame 62 | 11.3039 | 17.4419 | 1.5430 | 0.0283 |
| txndc9 | thioredoxin domain containing 9 | 3.5308 | 5.4571 | 1.5456 | 0.0343 |
| LRRC39 | leucine rich repeat containing 39 | 1.4252 | 2.2134 | 1.5530 | 0.0043 |
| Tceal7 | transcription elongation factor A (SII)-like 7 | 53.8349 | 83.7410 | 1.5555 | 0.0073 |
| SNX16 | sorting nexin 16 | 1.8465 | 2.8847 | 1.5622 | 0.0311 |
| COX6C | cytochrome c oxidase subunit VIc | 30.6973 | 48.2072 | 1.5704 | 0.0259 |
| C6orf115 | chromosome 6 open reading frame 115 | 6.8260 | 10.7973 | 1.5818 | 0.0041 |
| HINT1 | histidine triad nucleotide binding protein 1 | 55.0249 | 87.0891 | 1.5827 | 0.0013 |
| Tmem55a | transmembrane protein 55A | 8.6923 | 13.7606 | 1.5831 | 0.0091 |
| Mad2l1 | MAD2 mitotic arrest deficient-like 1 (yeast) | 1.8193 | 2.8884 | 1.5876 | 0.0311 |
| CISD1 | CDGSH iron sulfur domain 1 | 11.2123 | 17.8173 | 1.5891 | 0.0174 |
| GOLGA8A | "golgi autoantigen, golgin subfamily a, 8B; golgi autoantigen, golgin subfamily a, 8A" | 5.3398 | 8.5141 | 1.5945 | 0.0345 |
| GOLGA8B | "golgi autoantigen, golgin subfamily a, 8B; golgi autoantigen, golgin subfamily a, 8A" | 5.3398 | 8.5141 | 1.5945 | 0.0345 |
| EMB | embigin homolog (mouse) | 1.0999 | 1.7689 | 1.6082 | 0.0469 |
| Cox7b | cytochrome c oxidase subunit VIIb | 20.1009 | 32.3836 | 1.6111 | 0.0096 |
| sec61g | Sec61 gamma subunit | 7.4511 | 12.1191 | 1.6265 | 0.0319 |
| rbm11 | RNA binding motif protein 11 | 2.5681 | 4.1902 | 1.6316 | 0.0190 |
| POLR3G | polymerase (RNA) III (DNA directed) polypeptide G (32kD) | 1.2128 | 1.9803 | 1.6329 | 0.0384 |
| CCDC34 | coiled-coil domain containing 34 | 2.2126 | 3.6615 | 1.6549 | 0.0043 |
| TMSL2 | "thymosin-like 2 (pseudogene); thymosin-like 1 (pseudogene); thymosin beta 4, X-linked" | 275.7028 | 460.1926 | 1.6692 | 0.0245 |
| TMSL1 | "thymosin-like 2 (pseudogene); thymosin-like 1 (pseudogene); thymosin beta 4, X-linked" | 275.7028 | 460.1926 | 1.6692 | 0.0245 |
| TMSB4X | "thymosin-like 2 (pseudogene); thymosin-like 1 (pseudogene); thymosin beta 4, X-linked" | 275.7028 | 460.1926 | 1.6692 | 0.0245 |
| DYNLT3 | "dynein, light chain, Tctex-type 3" | 19.7038 | 33.4056 | 1.6954 | 0.0367 |
| Tctex1d1 | Tctex1 domain containing 1 | 1.0619 | 1.8061 | 1.7008 | 0.0058 |
| SVIP | small VCP/p97-interacting protein | 8.7777 | 14.9348 | 1.7014 | 0.0014 |
| Hist4h4 | "histone cluster 1, H4l; histone cluster 1, H4k; histone cluster 4, H4; histone cluster 1, H4h; histone cluster 1, H4j; histone cluster 1, H4i; histone cluster 1, H4d; histone cluster 1, H4c; histone cluster 1, H4f; histone cluster 1, H4e; histone cluster | 1.0957 | 1.8968 | 1.7311 | 0.0128 |
| Hist1h4j | "histone cluster 1, H4l; histone cluster 1, H4k; histone cluster 4, H4; histone cluster 1, H4h; histone cluster 1, H4j; histone cluster 1, H4i; histone cluster 1, H4d; histone cluster 1, H4c; histone cluster 1, H4f; histone cluster 1, H4e; histone cluster | 1.0957 | 1.8968 | 1.7311 | 0.0128 |
| Hist1h4b | "histone cluster 1, H4l; histone cluster 1, H4k; histone cluster 4, H4; histone cluster 1, H4h; histone cluster 1, H4j; histone cluster 1, H4i; histone cluster 1, H4d; histone cluster 1, H4c; histone cluster 1, H4f; histone cluster 1, H4e; histone cluster | 1.0957 | 1.8968 | 1.7311 | 0.0128 |
| HIST2H4B | "histone cluster 1, H4l; histone cluster 1, H4k; histone cluster 4, H4; histone cluster 1, H4h; histone cluster 1, H4j; histone cluster 1, H4i; histone cluster 1, H4d; histone cluster 1, H4c; histone cluster 1, H4f; histone cluster 1, H4e; histone cluster | 1.0957 | 1.8968 | 1.7311 | 0.0128 |
| Hist1h4a | "histone cluster 1, H4l; histone cluster 1, H4k; histone cluster 4, H4; histone cluster 1, H4h; histone cluster 1, H4j; histone cluster 1, H4i; histone cluster 1, H4d; histone cluster 1, H4c; histone cluster 1, H4f; histone cluster 1, H4e; histone cluster | 1.0957 | 1.8968 | 1.7311 | 0.0128 |
| HIST2H4A | "histone cluster 1, H4l; histone cluster 1, H4k; histone cluster 4, H4; histone cluster 1, H4h; histone cluster 1, H4j; histone cluster 1, H4i; histone cluster 1, H4d; histone cluster 1, H4c; histone cluster 1, H4f; histone cluster 1, H4e; histone cluster | 1.0957 | 1.8968 | 1.7311 | 0.0128 |
| Hist1h4i | "histone cluster 1, H4l; histone cluster 1, H4k; histone cluster 4, H4; histone cluster 1, H4h; histone cluster 1, H4j; histone cluster 1, H4i; histone cluster 1, H4d; histone cluster 1, H4c; histone cluster 1, H4f; histone cluster 1, H4e; histone cluster | 1.0957 | 1.8968 | 1.7311 | 0.0128 |
| Hist1h4h | "histone cluster 1, H4l; histone cluster 1, H4k; histone cluster 4, H4; histone cluster 1, H4h; histone cluster 1, H4j; histone cluster 1, H4i; histone cluster 1, H4d; histone cluster 1, H4c; histone cluster 1, H4f; histone cluster 1, H4e; histone cluster | 1.0957 | 1.8968 | 1.7311 | 0.0128 |
| Hist1h4f | "histone cluster 1, H4l; histone cluster 1, H4k; histone cluster 4, H4; histone cluster 1, H4h; histone cluster 1, H4j; histone cluster 1, H4i; histone cluster 1, H4d; histone cluster 1, H4c; histone cluster 1, H4f; histone cluster 1, H4e; histone cluster | 1.0957 | 1.8968 | 1.7311 | 0.0128 |
| Hist1h4c | "histone cluster 1, H4l; histone cluster 1, H4k; histone cluster 4, H4; histone cluster 1, H4h; histone cluster 1, H4j; histone cluster 1, H4i; histone cluster 1, H4d; histone cluster 1, H4c; histone cluster 1, H4f; histone cluster 1, H4e; histone cluster | 1.0957 | 1.8968 | 1.7311 | 0.0128 |
| hist1h4l | "histone cluster 1, H4l; histone cluster 1, H4k; histone cluster 4, H4; histone cluster 1, H4h; histone cluster 1, H4j; histone cluster 1, H4i; histone cluster 1, H4d; histone cluster 1, H4c; histone cluster 1, H4f; histone cluster 1, H4e; histone cluster | 1.0957 | 1.8968 | 1.7311 | 0.0128 |
| HIST1H4E | "histone cluster 1, H4l; histone cluster 1, H4k; histone cluster 4, H4; histone cluster 1, H4h; histone cluster 1, H4j; histone cluster 1, H4i; histone cluster 1, H4d; histone cluster 1, H4c; histone cluster 1, H4f; histone cluster 1, H4e; histone cluster | 1.0957 | 1.8968 | 1.7311 | 0.0128 |
| Hist1h4k | "histone cluster 1, H4l; histone cluster 1, H4k; histone cluster 4, H4; histone cluster 1, H4h; histone cluster 1, H4j; histone cluster 1, H4i; histone cluster 1, H4d; histone cluster 1, H4c; histone cluster 1, H4f; histone cluster 1, H4e; histone cluster | 1.0957 | 1.8968 | 1.7311 | 0.0128 |
| Hist1h4d | "histone cluster 1, H4l; histone cluster 1, H4k; histone cluster 4, H4; histone cluster 1, H4h; histone cluster 1, H4j; histone cluster 1, H4i; histone cluster 1, H4d; histone cluster 1, H4c; histone cluster 1, H4f; histone cluster 1, H4e; histone cluster | 1.0957 | 1.8968 | 1.7311 | 0.0128 |
| dnajc12 | "DnaJ (Hsp40) homolog, subfamily C, member 12" | 6.5600 | 11.4277 | 1.7420 | 0.0031 |
| C13orf27 | chromosome 13 open reading frame 27 | 2.2629 | 3.9721 | 1.7553 | 0.0014 |
| FAM133A | "family with sequence similarity 133, member A" | 1.5817 | 2.7784 | 1.7566 | 0.0274 |
| EEF1E1 | eukaryotic translation elongation factor 1 epsilon 1 | 2.1839 | 3.8576 | 1.7663 | 0.0234 |
| NECAB1 | N-terminal EF-hand calcium binding protein 1 | 25.7199 | 45.6002 | 1.7730 | 0.0411 |
| Gca | "grancalcin, EF-hand calcium binding protein" | 6.1870 | 11.0068 | 1.7790 | 0.0421 |
| Scoc | short coiled-coil protein | 35.9567 | 64.3714 | 1.7902 | 0.0199 |
| Kctd4 | potassium channel tetramerisation domain containing 4 | 3.1576 | 5.6729 | 1.7966 | 0.0378 |
| IFT80 | intraflagellar transport 80 homolog (Chlamydomonas) | 3.7537 | 6.9170 | 1.8427 | 0.0210 |
| LOC439953 | similar to TRIMCyp; peptidylprolyl isomerase A (cyclophilin A); peptidylprolyl isomerase A (cyclophilin A)-like 3 | 3.4614 | 6.6154 | 1.9112 | 0.0270 |
| ppiA | similar to TRIMCyp; peptidylprolyl isomerase A (cyclophilin A); peptidylprolyl isomerase A (cyclophilin A)-like 3 | 3.4614 | 6.6154 | 1.9112 | 0.0270 |
| PPIAL3 | similar to TRIMCyp; peptidylprolyl isomerase A (cyclophilin A); peptidylprolyl isomerase A (cyclophilin A)-like 3 | 3.4614 | 6.6154 | 1.9112 | 0.0270 |
| LOC728554 | similar to THO complex 3; THO complex 3 | 1.0830 | 2.1223 | 1.9597 | 0.0390 |
| THOC3 | similar to THO complex 3; THO complex 3 | 1.0830 | 2.1223 | 1.9597 | 0.0390 |
| SOSTDC1 | sclerostin domain containing 1 | 1.4245 | 2.8289 | 1.9859 | 0.0033 |
| RNU6-2 | "RNA, U6 small nuclear 2; RNA, U6 small nuclear 1" | 1.7841 | 3.6298 | 2.0345 | 0.0500 |
| RNU6-1 | "RNA, U6 small nuclear 2; RNA, U6 small nuclear 1" | 1.7841 | 3.6298 | 2.0345 | 0.0500 |
| RNU6-2 | "RNA, U6 small nuclear 2; RNA, U6 small nuclear 1" | 1.4456 | 3.0918 | 2.1388 | 0.0358 |
| RNU6-1 | "RNA, U6 small nuclear 2; RNA, U6 small nuclear 1" | 1.4456 | 3.0918 | 2.1388 | 0.0358 |
| GREM1 | "gremlin 1, cysteine knot superfamily, homolog (Xenopus laevis)" | 2.7284 | 6.9845 | 2.5599 | 0.0059 |
| RNU6-2 | "RNA, U6 small nuclear 2; RNA, U6 small nuclear 1" | 1.5332 | 4.0299 | 2.6284 | 0.0070 |
| RNU6-1 | "RNA, U6 small nuclear 2; RNA, U6 small nuclear 1" | 1.5332 | 4.0299 | 2.6284 | 0.0070 |

**Supplementary table 2.** Cytoscape functional pathway analysis of significant DEGs during human prefrontal cortex aging

| **GOTerm** | **Associated Genes Found** | **Nr. Genes** | **% Associated Genes** | **Term PValue** | **Term PValue Corrected with Bonferroni step down** |
| --- | --- | --- | --- | --- | --- |
| Oxidative phosphorylation | [ATP6V1G1, COX6C, NDUFAB1, NDUFB6, NDUFS4, PPA1, UQCRB, UQCRHL] | 8 | 6.0150 | 0.0595 | 0.7740 |
| Ribosome biogenesis in eukaryotes | [DKC1, NOP58, RBM28, RN5S1, RN5S10, RN5S11, RN5S12, RN5S13, RN5S14, RN5S15, RN5S16, RN5S17, RN5S2, RN5S3, RN5S4, RN5S5, RN5S6, RN5S7, RN5S8, RN5S9] | 20 | 18.8679 | 0.0000 | 0.0000 |
| Ribosome | [MRPL1, MRPL22, RN5S1, RN5S10, RN5S11, RN5S12, RN5S13, RN5S14, RN5S15, RN5S16, RN5S17, RN5S2, RN5S3, RN5S4, RN5S5, RN5S6, RN5S7, RN5S8, RN5S9, RPL15, RPL26L1, RPL36A] | 22 | 14.2857 | 0.0000 | 0.0000 |
| RNA transport | [ACIN1, CYFIP1, EIF1B, EIF3J, NUP188, RNU6-1, THOC3, UPF1] | 8 | 4.6784 | 0.1739 | 1.0000 |
| Spliceosome | [ACIN1, HNRNPA1L2, RNU6-1, SMNDC1, SNRNP200, SNRNP27, SNRPD1, SRSF8, THOC3] | 9 | 6.7164 | 0.0259 | 0.4655 |
| Rap1 signaling pathway | [ADCY2, CALM1, CALM2, CALM3, CTNND1, GNAO1, P2RY1, PLCG1, PRKD2, RAF1] | 10 | 4.7619 | 0.1285 | 1.0000 |
| cGMP-PKG signaling pathway | [ADCY2, ADRB1, CALM1, CALM2, CALM3, CREB3L1, GTF2IRD1, KCNMB4, PPP1CB, RAF1, RGS2] | 11 | 6.7485 | 0.0141 | 0.3516 |
| cAMP signaling pathway | [ADCY2, ADRB1, CALM1, CALM2, CALM3, CREB3L1, CREBBP, EP300, GABBR1, GRIN2D, PPP1CB, RAF1] | 12 | 6.0606 | 0.0229 | 0.4804 |
| Phosphatidylinositol signaling system | [CALM1, CALM2, CALM3, IMPA1, ITPK1, MTMR14, PI4KB, PLCG1] | 8 | 8.2474 | 0.0114 | 0.2967 |
| Oocyte meiosis | [ADCY2, ANAPC2, CALM1, CALM2, CALM3, PPP1CB, SKP1, SPDYA] | 8 | 6.4516 | 0.0424 | 0.6361 |
| Ubiquitin mediated proteolysis | [ANAPC2, DDB1, FBXW8, PIAS3, SKP1, SYVN1, TCEB1, UBE2D1, UBE2F, UBE2J2, UBOX5, WWP2] | 12 | 8.7591 | 0.0013 | 0.0406 |
| Protein processing in endoplasmic reticulum | [BAG2, DNAJB12, HSP90AA1, OS9, RNF185, SEC62, SKP1, SVIP, SYVN1, UBE2D1, UBE2J2] | 11 | 6.6265 | 0.0159 | 0.3665 |
| Endocytosis | [ADRB1, ARFGAP2, CLTA, EHD1, SH3GL1, SH3GL3, SMAD3, VPS29] | 8 | 3.0769 | 0.5834 | 0.5834 |
| mTOR signaling pathway | [ATP6V1G1, DVL2, DVL3, FZD4, IKBKB, LAMTOR3, LRP5, NPRL3, RAF1, SLC7A5, WNT7B] | 11 | 7.2848 | 0.0082 | 0.2294 |
| Wnt signaling pathway | [APC2, AXIN1, CACYBP, CREBBP, CSNK1E, DVL2, DVL3, EP300, FZD4, LRP5, PORCN, SKP1, SMAD3, WNT7B] | 14 | 9.7902 | 0.0002 | 0.0057 |
| Hippo signaling pathway | [APC2, AXIN1, CRB2, CSNK1D, CSNK1E, DVL2, DVL3, FZD4, PPP1CB, SMAD3, SMAD7, WNT7B, WWC1] | 13 | 8.4416 | 0.0011 | 0.0385 |
| Signaling pathways regulating pluripotency of stem cells | [APC2, AXIN1, BMI1, DVL2, DVL3, FZD4, RAF1, SMAD3, WNT7B] | 9 | 6.4748 | 0.0318 | 0.5400 |
| Long-term potentiation | [CALM1, CALM2, CALM3, CREBBP, EP300, GRIN2D, PPP1CB, RAF1] | 8 | 11.9403 | 0.0012 | 0.0381 |
| Regulation of actin cytoskeleton | [APC2, CYFIP1, MYH14, MYH9, PAK4, PPP1CB, PXN, RAF1, TMSB4X] | 9 | 4.2453 | 0.2282 | 0.6846 |
| Estrogen signaling pathway | [ADCY2, CALM1, CALM2, CALM3, CREB3L1, GABBR1, GNAO1, HSP90AA1, RAF1] | 9 | 9.1837 | 0.0037 | 0.1144 |
| Melanogenesis | [ADCY2, CALM1, CALM2, CALM3, CREB3L1, CREBBP, DVL2, DVL3, EP300, FZD4, GNAO1, RAF1, WNT7B] | 13 | 12.8713 | 0.0000 | 0.0006 |
| Thyroid hormone signaling pathway | [CREBBP, EP300, HDAC2, MED12, PLCG1, RAF1, RXRA, SIN3A, SLC16A2] | 9 | 7.7586 | 0.0110 | 0.2963 |
| Oxytocin signaling pathway | [ADCY2, CALM1, CALM2, CALM3, GNAO1, PPP1CB, RAF1, RGS2] | 8 | 5.2288 | 0.1114 | 1.0000 |
| Glucagon signaling pathway | [ADCY2, CALM1, CALM2, CALM3, CREB3L1, CREBBP, EP300, GYS1, PFKL] | 9 | 8.7379 | 0.0051 | 0.1490 |
| Non-alcoholic fatty liver disease (NAFLD) | [COX6C, CYCS, IKBKB, NDUFAB1, NDUFB6, NDUFS4, RXRA, UQCRB, UQCRHL] | 9 | 6.0403 | 0.0463 | 0.6478 |
| Alzheimer's disease | [CALM1, CALM2, CALM3, COX6C, CYCS, GRIN2D, NDUFAB1, NDUFB6, NDUFS4, UQCRB, UQCRHL] | 11 | 6.4327 | 0.0195 | 0.4282 |
| Parkinson's disease | [COX6C, CYCS, NDUFAB1, NDUFB6, NDUFS4, UBE2J2, UQCRB, UQCRHL] | 8 | 5.6338 | 0.0805 | 0.8855 |
| Huntington's disease | [CLTA, COX6C, CREB3L1, CREBBP, CYCS, EP300, HDAC2, NDUFAB1, NDUFB6, NDUFS4, NRF1, POLR2K, SIN3A, UQCRB, UQCRHL] | 15 | 7.7720 | 0.0011 | 0.0395 |
| Alcoholism | [CALM1, CALM2, CALM3, CREB3L1, GNAO1, GRIN2D, H2AFZ, HDAC2, HIST1H4E, HIST2H4A, HIST2H4B, PPP1CB, RAF1] | 13 | 7.2222 | 0.0045 | 0.1354 |
| Tuberculosis | [CALM1, CALM2, CALM3, CREBBP, CYCS, EP300, IFNGR2, RAF1] | 8 | 4.4693 | 0.2058 | 0.8232 |
| Hepatitis B | [CDKN1B, CREB3L1, CREBBP, CYCS, DDB1, EP300, IKBKB, MAVS, RAF1, SMAD3] | 10 | 6.9444 | 0.0157 | 0.3760 |
| Influenza A | [CREBBP, CYCS, EP300, IFNGR2, IKBKB, MAVS, NLRX1, RAF1] | 8 | 4.6243 | 0.1816 | 1.0000 |
| HTLV-I infection | [ADCY2, ANAPC2, APC2, CREBBP, DVL2, DVL3, EP300, FZD4, IKBKB, POLE3, SMAD3, VAC14, WNT7B] | 13 | 5.0781 | 0.0619 | 0.7430 |
| Herpes simplex infection | [CREBBP, CYCS, EP300, GTF2IRD1, IFIT1, IFNGR2, IKBKB, MAVS, PPP1CB, SKP1, SRSF8] | 11 | 5.9459 | 0.0324 | 0.5183 |
| Epstein-Barr virus infection | [CDKN1B, CREBBP, EP300, HDAC2, IKBKB, PLCG1, POLR2K, POLR3G, PSMD14] | 9 | 4.4335 | 0.1933 | 0.9667 |
| Pathways in cancer | [ADCY2, APC2, AXIN1, CDKN1B, CREBBP, CYCS, DVL2, DVL3, EP300, FH, FZD4, HDAC2, HSP90AA1, IKBKB, PLCG1, RAF1, RXRA, SMAD3, TCEB1, WNT7B] | 20 | 5.0633 | 0.0247 | 0.4933 |
| Viral carcinogenesis | [CDKN1B, CHD4, CREB3L1, CREBBP, DDB1, EP300, HDAC2, HIST1H4E, HIST2H4A, HIST2H4B, PXN, VAC14] | 12 | 5.9701 | 0.0254 | 0.4820 |
| MicroRNAs in cancer | [APC2, BMI1, CDKN1B, CREBBP, EP300, IKBKB, PAK4, PLCG1, RAF1, SPRY2] | 10 | 3.3445 | 0.4747 | 0.9493 |
| Breast cancer | [APC2, AXIN1, DVL2, DVL3, FZD4, LRP5, RAF1, WNT7B] | 8 | 5.5556 | 0.0857 | 0.8569 |

**Supplementary table 3.** Aging-related gene prioritization by Endeavor using a PD-associated training gene set and the mRNA expression fold changes of each gene in the cortex and ventral midbrains of 8-month-old versus 34-month-old mice

| **#** | **Gene** | **Description** | **Human PFC (FC, old/adult)** | **Mouse CTX (FC, long-lived/adult)** | **Mouse VM (FC, long-lived/adult)** |
| --- | --- | --- | --- | --- | --- |
| 1 | EIF4G1 | Eukaryotic translation initiation factor 4 gamma, 1 | 0.70 | 0.68 | 0.76 |
| 2 | AIMP2 | Aminoacyl tRNA synthetase complex-interacting multifunctional protein 2 | 0.84 | 0.98 | 2.22 |
| 3 | FBXW5 | F-box and WD repeat domain containing 5 | 0.70 | 0.92 | 1.04 |
| 4 | ATXN2 | Ataxin 2 | 0.81 | 0.94 | 1.16 |
| 5 | AKT1 | v-akt murine thymoma viral oncogene homolog 1 | 0.67 | 1.12 | 1.58 |
| 6 | RAF1 | v-raf-1 murine leukemia viral oncogene homolog 1 | 0.89 | 0.84 | 1.15 |
| 7 | CREBBP | CREB binding protein | 0.64 | 0.96 | 1.38 |
| 8 | DNAJB9 | DnaJ (Hsp40) homolog, subfamily B, member 9 | 1.38 | 0.98 | 1.51 |
| 9 | BRF1 | BRF1 homolog | 0.75 | 1.10 | 1.05 |
| 10 | CYCS | cytochrome c, somatic | 1.34 | 1.01 | 2.27 |
| 11 | GTF2B | general transcription factor IIB | 1.24 | 0.82 | 2.99 |
| 12 | CDKN1B | cyclin-dependent kinase inhibitor 1B | 1.28 | 0.78 | 2.63 |
| 13 | AXIN1 | axin 1 | 0.74 | 0.92 | 1.66 |
| 14 | WWP2 | WW domain containing E3 ubiquitin protein ligase 2 | 0.77 | 0.97 | 1.60 |
| 15 | AKT2 | v-akt murine thymoma viral oncogene homolog 2 | 0.76 | 0.86 | 2.19 |
| 16 | BAG2 | BCL2-associated athanogene 2 | 1.47 | 1.15 | 1.25 |
| 17 | TCF7L2 | transcription factor 7-like 2 | 0.68 | 0.51 | 4.40 |
| 18 | UBE2D1 | ubiquitin-conjugating enzyme E2D 1 | 1.37 | 1.09 | 2.29 |
| 19 | TRAF7 | TNF receptor-associated factor 7 | 0.67 | 0.82 | 1.28 |
| 20 | HEXB | hexosaminidase B | 1.24 | 0.87 | 2.03 |
| 21 | NDOR1 | NADPH dependent diflavin oxidoreductase 1 | 0.72 | 0.96 | 1.01 |
| 22 | ADRB1 | adrenergic, beta-1-, receptor | 0.72 | 0.73 | 1.25 |
| 23 | NOTCH1 | Notch homolog 1 | 0.58 | 0.74 | 1.43 |
| 24 | UBE2J2 | ubiquitin-conjugating enzyme E2, J | 0.78 | 0.97 | 0.98 |
| 25 | *LRP1* | low density lipoprotein-related protein 1 | 0.52 | 1.13 | 1.22 |
| 26 | POR | P450 (cytochrome) oxidoreductase | 0.68 | 1.31 | 1.57 |
| 27 | LDLR | low density lipoprotein receptor | 0.66 | 1.11 | 1.24 |
| 28 | SLC6A1 | solute carrier family 6, member 1 | 0.61 | 0.73 | 1.65 |
| 29 | SP2 | Sp2 transcription factor | 0.71 | 0.83 | 1.57 |
| 30 | CACYBP | similar to calcyclin binding protein; calcyclin binding protein | 1.33 | 1.48 | 1.77 |
| 31 | TBCD | tubulin folding cofactor D | 0.70 | 0.79 | 0.41 |
| 32 | GTF3C2 | general transcription factor IIIC, polypeptide 2, beta | 0.85 | 0.91 | 1.06 |
| 33 | NRF1 | nuclear respiratory factor 1 | 0.84 | 0.85 | 2.02 |

**Supplementary Table 4.** Oligonucleotide primers used for the amplification of the indicated mouse genes

| **#** | **Gene** | **Forward (5’–3’)** | **Reverse (5’–3’)** |
| --- | --- | --- | --- |
| 1 | *eif4g1* | GGTGGTGTTTAGCACGCCT | CAGCAGGGTAGACATGGGG |
| 2 | *aimp2* | AACGCTTGTATGAGTTGAAGGC | TCTGGGGTGTGAATCATCTTTG |
| 3 | *fbxw5* | GAGTTCCGGCGGCTCTATG | AGATGGTCAGGTCGTTGTTCC |
| 4 | *atxn2* | GCTCTCAGCGCAAAGGTGA | GGACACCACACCATAATTCTCTT |
| 5 | *akt1* | CCTTTATTGGCTACAAGGAACGG | GAAGGTGCGCTCAATGACTG |
| 6 | *raf1* | TGGAATGAGCTTACATGACTGC | GTTGTGAGTGGAACATGATCCAA |
| 7 | *crebbp* | TTCTCCGCGAATGACAACACA | CCTGGGTTGATGCTAGAGCC |
| 8 | *dnajb9* | CTCCACAGTCAGTTTTCGTCTT | GGCCTTTTTGATTTGTCGCTC |
| 9 | *brf1* | GGGAAGACGTTCCTGCTCTTG | GTGCATCCAATCCCTTTTCATTC |
| 10 | *cycs* | CCAAATCTCCACGGTCTGTTC | ATCAGGGTATCCTCTCCCCAG |
| 11 | *gtf2b* | TCCCGAATGTGGCCTAGTTG | TTGCTCGATCAGAGCTACTCA |
| 12 | *cdkn1b* | GGGCAGATACGAGTGGCAG | TGAGACCCAATTAAAGGCACC |
| 13 | *axin1* | CATTGTGTCCAGACAAACCAAGC | GGAAGGGTAGGTATTCTCCTCCA |
| 14 | *wwp2* | TTTGAGAAGTCCCAGCTTACCC | CTCCAGACCTTCAGATCCAAATG |
| 15 | *akt2* | GGCCCCTGACCAGACCTTA | GATAGCCCGCATCCACTCTTC |
| 16 | *bag2* | AGACGCAGCTACTGCTGTTG | CGGATCGTTTCCACCGAGAC |
| 17 | *tcf7l2* | CACCGACAGTCAAGCAGGAAT | CCACCTTCGCTCTCATCTCTTT |
| 18 | *ube2d1* | CCCGTGGGAGATGACTTGTTC | GGATAGTCTGTCGGAAAGTGGA |
| 19 | *traf7* | CAGCCCTCGGTAAAGCTGTG | ACTGCAATGTTGTTCACCACTA |
| 20 | *hexb* | CTGGTGTCGCTAGTGTCGC | CAGGGCCATGATGTCTCTTG |
| 21 | *ndor1* | TCTTGGAGGTGCTATGCGACT | GGGGCTCCTTCAGACGAGT |
| 22 | *adrb1* | CTCATCGTGGTGGGTAACGTG | ACACACAGCACATCTACCGAA |
| 23 | *notch1* | CCCTTGCTCTGCCTAACGC | GGAGTCCTGGCATCGTTGG |
| 24 | *ube2j2* | CTTGAATGGCATTATGTTGTCCG | CAGCCTTGTGTTGCACTTAAATC |
| 25 | *lrp1* | CAGAGATGCCCGCCAAATGA | CGTCAAAATCTTTGCACGTCTTG |
| 26 | *por* | ATGGGGGACTCTCACGAAGAC | TCTTGCTGAACTCCGGTATCTC |
| 27 | *ldlr* | GAAGGCAGCTACAAGTGTGAG | GGGGAGCAGACTGGTGTACT |
| 28 | *slc6a1* | GAAAGCTGTCTGATTCTGAGGTG | AGCAAACGATGATGGAGTCCC |
| 29 | *sp2* | CCAGCCTACCCCAAGGAAAC | GGGAGCCCTGAATCTGAAGTAT |
| 30 | *cacybp* | ATGGGACTACTTAACACAGGTGG | GGTTCGCTTCATATCATCGTCTC |
| 31 | *tbcd* | ATGGTACTGAGCAATGAGCCG | GTTCATCATCCACTCAAGGTGT |
| 32 | *gtf3c2* | CTCCCCCTCCACAACAATTCC | AGCAATTTGACTCCCAAGCCT |
| 33 | *nrf1* | AGCACGGAGTGACCCAAAC | AGGATGTCCGAGTCATCATAAGA |
| 34 | *gapdh* | AACTTTGGCATTGTGGAAGG | ACACATTGGGGGTAGGAACA |
